# Impact of vitamin C supplementation on placental DNA methylation changes related to maternal smoking: association with gene expression and respiratory outcomes

**DOI:** 10.1186/s13148-021-01161-y

**Published:** 2021-09-19

**Authors:** Lyndsey E. Shorey-Kendrick, Cindy T. McEvoy, Shannon M. O’Sullivan, Kristin Milner, Brittany Vuylsteke, Robert S. Tepper, David M. Haas, Byung Park, Lina Gao, Annette Vu, Cynthia D. Morris, Eliot R. Spindel

**Affiliations:** 1 Division of Neuroscience, Oregon National Primate Research Center, Oregon Health and Science University, 505 NW 185th Ave, Beaverton, OR 97006 USA; 2 Department of Pediatrics, Oregon Health and Science University, Portland, OR USA; 3 Department of Pediatrics, Herman B Wells Center for Pediatric Research, Indiana University School of Medicine, Indianapolis, IN USA; 4 Department of Obstetrics and Gynecology, Indiana University School of Medicine, Indianapolis, IN USA; 5 Biostatistics Shared Resources, Knight Cancer Institute, Oregon Health and Science University, Portland, OR USA; 6 Bioinformatics and Biostatistics Core, Oregon National Primate Research Center, Oregon Health and Science University, Portland, OR USA; 7 School of Public Health, Oregon Health and Science University-Portland State University, Portland, OR USA; 8 Department of Medical Informatics and Clinical Epidemiology, Oregon Health and Science University, Portland, OR USA; 9 Oregon Clinical and Translational Research Institute, Oregon Health and Science, Portland, OR USA

**Keywords:** MSDP: maternal smoking during pregnancy, Vitamin C, RCT: randomized clinical trial, DNA methylation, MethylationEPIC, Nicotine

## Abstract

**Background:**

Maternal smoking during pregnancy (MSDP) affects development of multiple organ systems including the placenta, lung, brain, and vasculature. In particular, children exposed to MSDP show lifelong deficits in pulmonary function and increased risk of asthma and wheeze. Our laboratory has previously shown that vitamin C supplementation during pregnancy prevents some of the adverse effects of MSDP on offspring respiratory outcomes. Epigenetic modifications, including DNA methylation (DNAm), are a likely link between in utero exposures and adverse health outcomes, and MSDP has previously been associated with DNAm changes in blood, placenta, and buccal epithelium. Analysis of placental DNAm may reveal critical targets of MSDP and vitamin C relevant to respiratory health outcomes.

**Results:**

DNAm was measured in placentas obtained from 72 smokers enrolled in the VCSIP RCT: NCT03203603 (37 supplemented with vitamin C, 35 with placebo) and 24 never-smokers for reference. Methylation at one CpG, cg20790161, reached Bonferroni significance and was hypomethylated in vitamin C supplemented smokers versus placebo. Analysis of spatially related CpGs identified 93 candidate differentially methylated regions (DMRs) between treatment groups, including loci known to be associated with lung function, oxidative stress, fetal development and growth, and angiogenesis. Overlap of nominally significant differentially methylated CpGs (DMCs) in never-smokers versus placebo with nominally significant DMCs in vitamin C versus placebo identified 9059 candidate “restored CpGs” for association with placental transcript expression and respiratory outcomes. Methylation at 274 restored candidate CpG sites was associated with expression of 259 genes (FDR < 0.05). We further identified candidate CpGs associated with infant lung function (34 CpGs) and composite wheeze (1 CpG) at 12 months of age (FDR < 0.05). Increased methylation in the *DIP2C*, *APOH/PRKCA*, and additional candidate gene regions was associated with improved lung function and decreased wheeze in offspring of vitamin C-treated smokers.

**Conclusions:**

Vitamin C supplementation to pregnant smokers ameliorates changes associated with maternal smoking in placental DNA methylation and gene expression in pathways potentially linked to improved placental function and offspring respiratory health. Further work is necessary to validate candidate loci and elucidate the causal pathway between placental methylation changes and outcomes of offspring exposed to MSDP.

*Clinical trial registration* ClinicalTrials.gov, NCT01723696. Registered November 6, 2012. https://clinicaltrials.gov/ct2/show/record/NCT01723696.

**Supplementary Information:**

The online version contains supplementary material available at 10.1186/s13148-021-01161-y.

## Background

Maternal smoking during pregnancy (MSDP) is the leading preventable cause of prematurity, intrauterine growth restriction (IUGR), and perinatal death [1–4]; however, despite smoking cessation efforts, over 50% of smokers will continue to smoke during pregnancy [5, 6]. MSDP affects development of multiple organ systems including placenta, lung, brain, and vasculature [7–12] and is associated with altered DNA methylation in placenta, blood, and buccal epithelium [13–24]. Perhaps the best-characterized effects of MSDP on long-term offspring health are respiratory outcomes. Offspring exposed to MSDP exhibit lifetime decreases in airway function and increased risk of wheeze and asthma [12, 25, 26]. Mechanisms of MSDP on fetal lung development include both direct effects of nicotine, nicotine metabolites, and other harmful components in cigarette smoke on the developing fetus [12], and indirect effects on the feto-placental unit [27].

The placenta plays a key role in the overall health and development of the fetus through the supply of oxygen and nutrients, gas and waste exchange, and regulation of fetal growth in response to fetal endocrine signals [28]. Compromised placental function is associated with increased risk of cardiometabolic diseases in later life [29, 30], and smoking during pregnancy is specifically associated with impairment in both placental structure and function. For instance, multiple Doppler-ultrasound (Doppler-US) studies have shown that smoking during pregnancy decreases placental blood flow and increases umbilical artery pulsatility index [11, 31, 32]. Placental pathologies in smokers include increased thickness of the villous membrane, increased collagen deposition, decreased vascularization of the placental bed, as well as reduced intervillous area and capillary volume [27, 33].

DNA methylation (DNAm) is the covalent addition of a methyl group, found primarily at cytosine–guanine (CpG) dinucleotides, and can act to regulate gene expression at local and distant loci through modification of chromatin state and transcription factor binding [34]. DNA methylation regulates processes critical to placental development [35] and may provide a mechanistic link between in utero exposures and future health outcomes [36–39]. Placental DNAm is dysregulated with in utero exposure to heavy metals, alcohol, and air pollution [40–42], with some of the largest effect sizes reported in association with MSDP [15, 43–49]. Previous studies have reported global and gene-specific changes in placental DNAm in response to MSDP [19, 42, 50], and some of these changes are proposed to mediate the effect of MSDP on health outcomes, such as infant birth weight and psychiatric morbidity [13, 23, 51].

We have previously demonstrated that vitamin C supplementation (500 mg/day) to pregnant smokers unable to quit smoking significantly increased newborn lung function and infant airway function through one year of age in two double-blind, randomized control trials [52–54]. In our pilot clinical trial population, we demonstrated using targeted bisulfite sequencing that vitamin C supplementation during pregnancy could restore levels of DNAm in candidate genes in placenta, in parallel with improved lung function, as well as in cord blood and childhood buccal DNA [55]. We also recently demonstrated that vitamin C supplementation improved placental hemodynamics in an ancillary study of Doppler-US in a subset of participants in the second VCSIP RCT [56]. Therefore, improved lung function in offspring from pregnancies supplemented by vitamin C may parallel improved placental structure and function. We hypothesize that epigenome-wide analysis of placental DNAm in this cohort may reveal loci dysregulated with MSDP that are improved or protected by vitamin C supplementation, and that these changes may also relate to improved infant lung function.

To test this hypothesis, we measured placental DNAm genome-wide using the Illumina MethylationEPIC array platform in a subset of placentas collected at delivery from participants in the VCSIP RCT. We used a concept-driven analysis approach (Additional file 1: Figure S1) to identify candidate differentially methylated CpGs (DMCs) potentially relevant to lung function. We also performed differentially methylated region (DMR) analysis, which may provide greater evidence of a functional impact on gene expression than individual CpGs [57]. We conducted enrichment analyses within candidate overlapping (i.e., “normalized”) DMCs and DMRs to identify biological pathways and processes which vitamin C supplementation may protect, and expression quantitative trait methylation (eQTM) analysis to identify genes with mRNA correlated with methylation changes. Lastly, we examined association of candidate CpGs with infant lung function and composite wheeze measured at 12 months of age.

## Results

### Baseline characteristics

We measured epigenome-wide DNA methylation in placentas obtained at delivery from 72 smoking participants from the “Vitamin C to Decrease the Effects of Smoking in Pregnancy on Infant Lung Function” (VCSIP) multi-center, double-blind RCT (35 placebo and 37 vitamin C supplemented) and from 24 never-smokers for reference [53, 54, 58]. Additional file 2: Table S1 presents the maternal and infant demographics and birth statistics per group (never-smokers, placebo smokers, vitamin C smokers) for the 96 subjects with placental epigenome-wide DNAm measurements. There were no significant differences in baseline characteristics between the vitamin C-supplemented smokers and placebo-supplemented smokers included on the MethylationEPIC arrays. Gene expression (total RNA-sequencing) was also available in 71 of the placental samples (26 placebo, 27 vitamin C, and 18 never-smokers; Additional file 1: Figure S2) [56].

### Differentially methylated loci between treatment groups

**Fig. 1:**
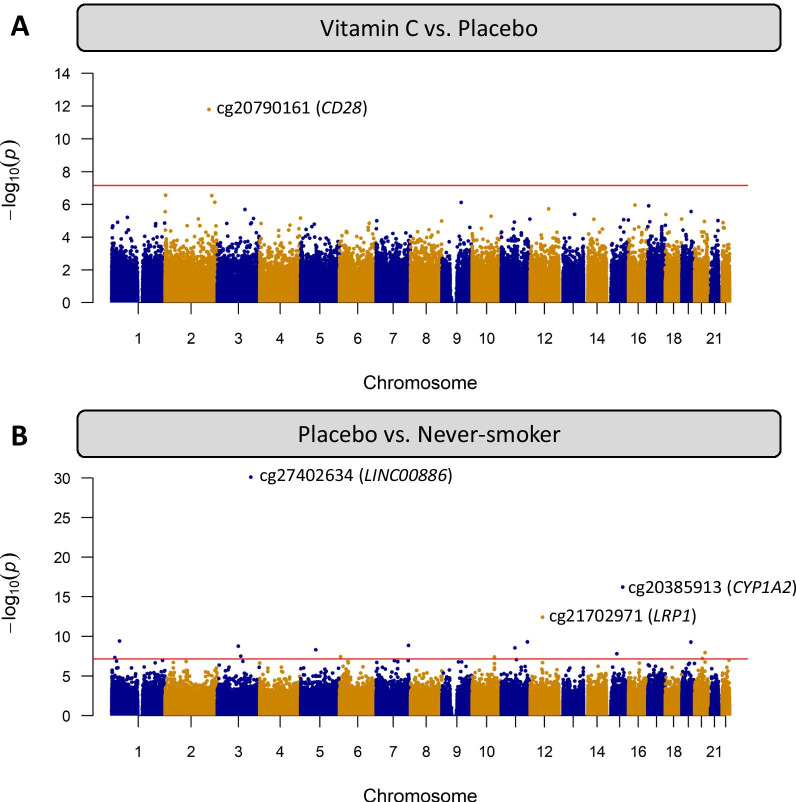
Manhattan plots of differential placental DNAm between **a** placebo and vitamin C smokers and **b** placebo smokers and never-smokers. The horizontal red line marks Bonferroni adjusted significance (unadjusted *p* < 0.05/714666 CpGs = 6.99 e−08). The top 3 Bonferroni significant CpGs per comparison are annotated to the nearest proximal gene

**Fig. 2:**
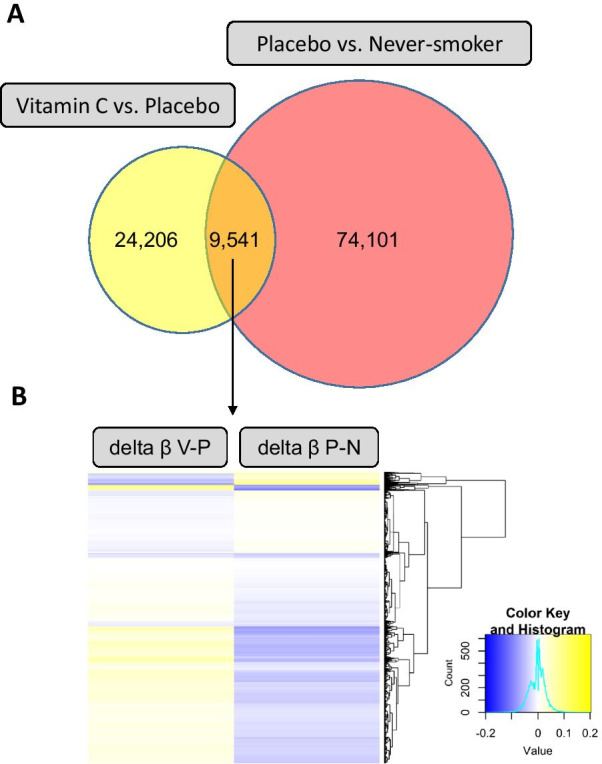
Selection of candidate CpG sites used for functional enrichment analysis, eQTM analysis, and association with FEF_75_ and wheeze at 12 months of age. **a** Venn diagram showing overlap of nominally significant DMCs between groups (*n* = 9541 CpGs overlapping). **b** Heatmap showing the magnitude and direction of methylation change (delta beta) between sample groups (P–N: placebo vs never-smoker; V–P: vitamin C vs placebo). Only the CpGs with a reversal in the direction of methylation change with vitamin C supplementation (*n* = 9059 CpGs “partially restored”) were considered in downstream analyses

We performed epigenome-wide robust linear regression analysis to measure differential methylation in placentas from smokers randomized to placebo versus vitamin C. We also recruited pregnant never-smokers, as a reference group, to identify loci differentially methylated with MSDP and potentially normalized with vitamin C. All models were adjusted for cellular heterogeneity, infant sex, and gestational age at delivery. Comparison of vitamin C supplemented smokers and placebo smokers identified a single Bonferroni significant CpG (cg20790161; chr2:204553666), located in an intergenic region 17.5 Kbp upstream of *CD28* (Fig. 1a; Table 1), which was also the only FDR-DMC between RCT groups. The majority (61%) of nominally significant CpGs between RCT groups had increased methylation in placentas from vitamin C smokers versus placebo. Comparison of never-smokers to placebo supplemented smokers identified 17 CpGs at Bonferroni significance (Fig. 1b; Table 1), 7 of which have been previously reported as differentially methylated with MSDP in placenta with consistent direction and magnitude of effect size and 726 CpGs with FDR adjusted *p* < 0.05. Results from sensitivity analyses adding ethnicity (self-reported), or removing adjustments for cell heterogeneity, infant sex, or gestational age, were not observably different from the original model (Additional File 1: Figure S3). The top CpG associated with maternal smoking status was cg27402634 (chr3:156536860), with a striking decrease of 31.4% in DNAm in placentas from placebo smokers versus never-smokers (beta scale SE = 0.018; *p* = 3.88E−31), and was not restored with vitamin C. The majority (82%) of MSDP associated loci (placebo-smokers compared with never-smokers) were hypomethylated. Novel CpGs differentially methylated in placebo versus never-smokers at Bonferroni significance mapped to 10 unique genes (*MIR100HG, SSPO, ST3GAL6-AS1, MARK2, BDP1, SLC9A8, NECTIN3-AS1, PPP1R3G, PRDM2,* and *SRC*) (Fig. 2).

**Table 1:** Bonferroni significant DMCs in vitamin C smokers versus placebo (VP) and in placebo versus never-smokers (PN)

Probe	Comparison	Location	Nearest gene	Distance to nearest gene (Kbp)	Bonferroni significant EWAS results*		
					Delta beta	*P* value	Bonferroni adj. *P* value
cg20790161	VP	chr2:204553666	*CD28*	17.5	− 0.16	1.63E−12	1.16E−06
–	–	–	–	–	–	–	–
cg27402634	PN	chr3:156536860	*LINC00886*	2.0	− 0.31	7.53E−31	5.38E−25
cg20385913	PN	chr15:75054510	*CYP1A2*	13.3	0.08	5.91E−17	4.22E−11
cg21702971	PN	chr12:57588243	*LRP1*	66.0	0.03	3.66E−13	2.61E−07
cg04895640	PN	chr1:36807418	*STK40*	19.5	0.02	3.85E−10	2.75E−04
cg12026561	PN	chr11:122238873	*MIR100HG*	12.9	− 0.04	4.78E−10	3.42E−04
cg03313447	PN	chr19:41829042	*CCDC97*	0.4	− 0.05	5.14E−10	3.68E−04
cg25639387	PN	chr7:149503093	*SSPO*	30.0	− 0.04	1.32E−09	9.47E−04
cg03256631	PN	chr3:98445144	*ST3GAL6-AS1*	6.4	− 0.07	1.64E−09	1.17E−03
cg21362410	PN	chr11:63650618	*MARK2*	44.2	0.04	2.59E−09	1.85E−03
cg07752120	PN	chr5:70746259	*BDP1*	5.2	− 0.02	4.67E−09	3.34E−03
cg24797066	PN	chr20:48407084	*SLC9A8*	22.2	− 0.07	1.08E−08	7.73E−03
cg24372191	PN	chr15:48116584	*SEMA6D*	105.9	0.06	1.50E−08	1.07E−02
cg04572941	PN	chr3:110033667	*NECTIN3-AS1*	755.1	− 0.04	3.06E−08	2.18E−02
cg10563109	PN	chr6:5087749	*PPP1R3G*	2.0	0.02	3.49E−08	2.49E−02
cg14015502	PN	chr10:104535020	*WBP1L*	31.3	− 0.08	3.77E−08	2.69E−02
cg00376466	PN	chr1:14463622	*PRDM2*	387.8	− 0.08	4.39E−08	3.14E−02
cg24034752	PN	chr20:36023689	*SRC*	49.1	− 0.04	5.95E−08	4.25E−02

Comparison: VP = Vitamin C versus Placebo, PN = Placebo versus Never-smokers; Nearest gene = nearest proximal gene annotated using the GenomicRanges package in R; Delta beta = coefficient from beta-scale robust linear regression

^*^Adjusted for infant sex, gestational age at delivery, and estimated cellular heterogeneity

**Fig. 3:**
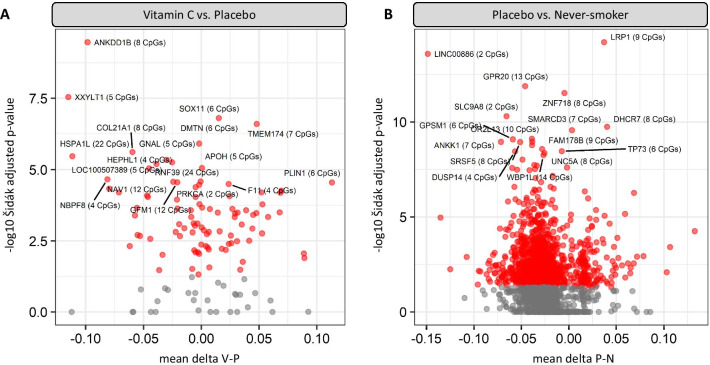
Volcano plots of differentially methylated regions (DMRs) in placenta between **a** placebo and vitamin C smokers and **b** placebo smokers and never-smokers. DMRs with a Šidák adjusted *p *value < 0.05 are shown in red. The top 20 DMRs per comparison are annotated with the nearest gene and number of CpGs. The x-axis denotes the average delta-beta between sample groups across the DMR region

We next used comb-p to identify differentially methylated regions (DMRs) [59], a method that combines adjacent *p* values in sliding windows, and required a *p *value of 0.05 to start a region and extended the region if another significant *p *value was within 500 base pairs [60]. We identified 93 DMRs between randomized treatment groups (vitamin C-smokers vs placebo-smokers; VP-DMRs; Fig. 3a; Additional file 2: Table S3) that spanned 548 individual CpGs (278 hypomethylated in placebo/ 270 hypermethylated) and 82 unique genes. Visual inspection of individual DMR beta values and genomic locations confirmed consistent patterns of DNAm within loci and proximity to critical regulatory features (i.e., CpG islands, transcription factor binding sites, DNAse hypersensitivity sites, etc.) for the majority of DMRs (Additional file 3). The top significant PV-DMR (mean ∆*β* *P*–*V* = 9.85%; Šidák adjusted *p* = 3.45E−10) covered a CpG island and shore across the transcription start site (TSS) for *ANKDD1B* (chr5:74907152–74908171). Further, we identified 3 PV-DMR loci with a mean DNA methylation difference greater than 10% (*PLIN1, HSPA1A/1L, and XXYTL1*). In the comparison of never-smokers versus placebo-supplemented smokers, we identified 1359 Šidák significant NP-DMRs (Fig. 3b; Additional file 2: Table S4), with the top 3 significant DMRs annotated to *LRP1, LINC00886, and GPR20*. Out of the 93 PV-DMR loci between randomized treatment groups, 25 overlapped with NP-DMRs, proximal to 16 unique genes (Table 2). The top significant restored DMR (mean ∆*β* *V*–*P* = 4.8%; mean ∆*β* *P*–*N* = −2.3%; Šidák adjusted *p* = 2.52E−7) was located in an intergenic region (chr5:72596701–72597716) located between *TMEM174* and *FOXD1*. Additionally, multiple restored DMRs mapped to *PRKCA* (3 DMRs) and *DIP2C* (2 DMRs).

**Table 2:** Restored DMRs in vitamin C supplemented smokers versus placebo

Chr	Start	End	Nearest genes	n CpGs	Šidák adjusted *p *value	Genomic Region	mean ∆*β* VP	mean ∆*β* PN	% Restored
5	72596701	72597716	*TMEM174/FOXD1*	7	2.52E−07	IGR-shore	4.8%	− 2.3%	207%
17	64225346	64226104	*APOH*	5	8.85E−06	Body-opensea	0.1%	− 0.2%	33%
17	64652223	64652404	*PRKCA*	2	2.63E−05	Body-opensea	− 0.1%	0.8%	7%
7	150951482	150952100	*SMARCD3*	7	9.14E−05	Body-shelf	− 0.5%	0.3%	143%
10	743286	743974	*DIP2C*	6	1.65E−04	IGR-island	− 0.4%	0.1%	654%
14	64760831	64761520	*ESR2*	8	2.16E−04	1stExon-shore	0.1%	− 0.9%	8%
14	100203730	100204259	*EML1*	8	2.45E−04	IGR-shore	0.7%	− 1.7%	43%
17	81015941	81016295	*B3GNTL1*	4	2.70E−04	IGR-shore	− 0.9%	0.2%	403%
11	31832710	31833310	*PAX6*	13	4.10E−04	5'UTR-island	− 5.7%	1.4%	419%
7	149472934	149473464	*SSPO*	4	7.88E−04	TSS200-shore	− 0.3%	0.2%	167%
9	140193776	140193935	*NRARP*	3	8.37E−04	IGR-shore	− 1.9%	1.6%	117%
4	42399115	42400465	*SHISA3*	9	9.46E−04	TSS1500-shore	− 0.6%	1.3%	42%
5	176810555	176811258	*SLC34A1*	7	1.36E−03	TSS1500-opensea	0.2%	− 0.2%	114%
18	12727080	12727198	*PSMG2*	2	4.26E−03	IGR-opensea	0.0%	0.3%	2%
17	64636808	64636928	*PRKCA*	2	5.36E−03	Body-opensea	0.6%	− 0.3%	185%
2	103041487	103041922	*IL18RAP*	2	6.31E−03	Body-opensea	0.6%	− 1.2%	54%
17	64499496	64499742	*PRKCA*	2	7.98E−03	Body-opensea	− 0.3%	0.3%	102%
10	740024	740191	*DIP2C*	2	8.97E−03	IGR-shelf	− 1.0%	0.4%	240%
7	150896413	150896490	*ABCF2*	3	3.07E−02	IGR-opensea	− 0.8%	0.4%	195%

Chr = Chromosome; Start/End = hg19 base pair positions; Nearest gene = nearest proximal gene using the chromosome and positions (GrCh37/hg19) provided for each probe in the Illumina HumanMethylationEPIC annotation file, matched to the nearest gene symbol using the GenomicRanges package in R; n CpGs = number of CpGs in DMR; ∆β VP = delta beta vitamin C versus placebo smokers; ∆β PN = delta beta placebo versus nonsmokers; percent restored = (− ∆*β* VP/∆*β* PN) * 100

For downstream analyses, we focused on “candidate restored DMCs” based on the overlap of nominally significant CpGs between the two comparisons (PN: placebo vs never-smoker; VP: vitamin C vs placebo; *n* = 9541 CpGs; Fig. 2a) and showing any restoration with vitamin C treatment of the methylation change caused by maternal smoking. In 9059 out of 9541 overlapping CpGs (95%), the average mean difference in methylation between vitamin C smokers and placebo smokers was in the opposite direction of the effect between placebo smokers and never-smokers (Fig. 2b; Additional file 2: Table S2), consistent with partial restoration.

### Association of candidate DMCs with infant lung function and wheeze

**Fig. 4:**
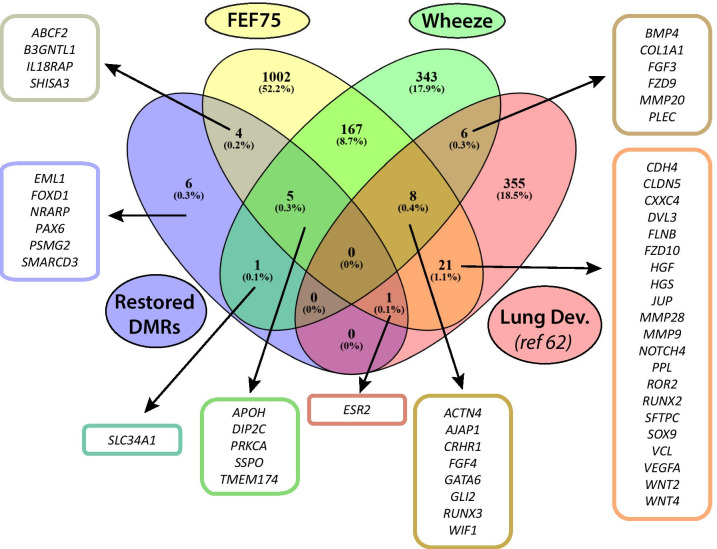
Overlap of genes containing restored DMRs and candidate loci nominally associated with respiratory outcomes (12 month FEF_75_ and/or composite wheeze score) and candidate lung development genes [61]

**Fig. 5:**
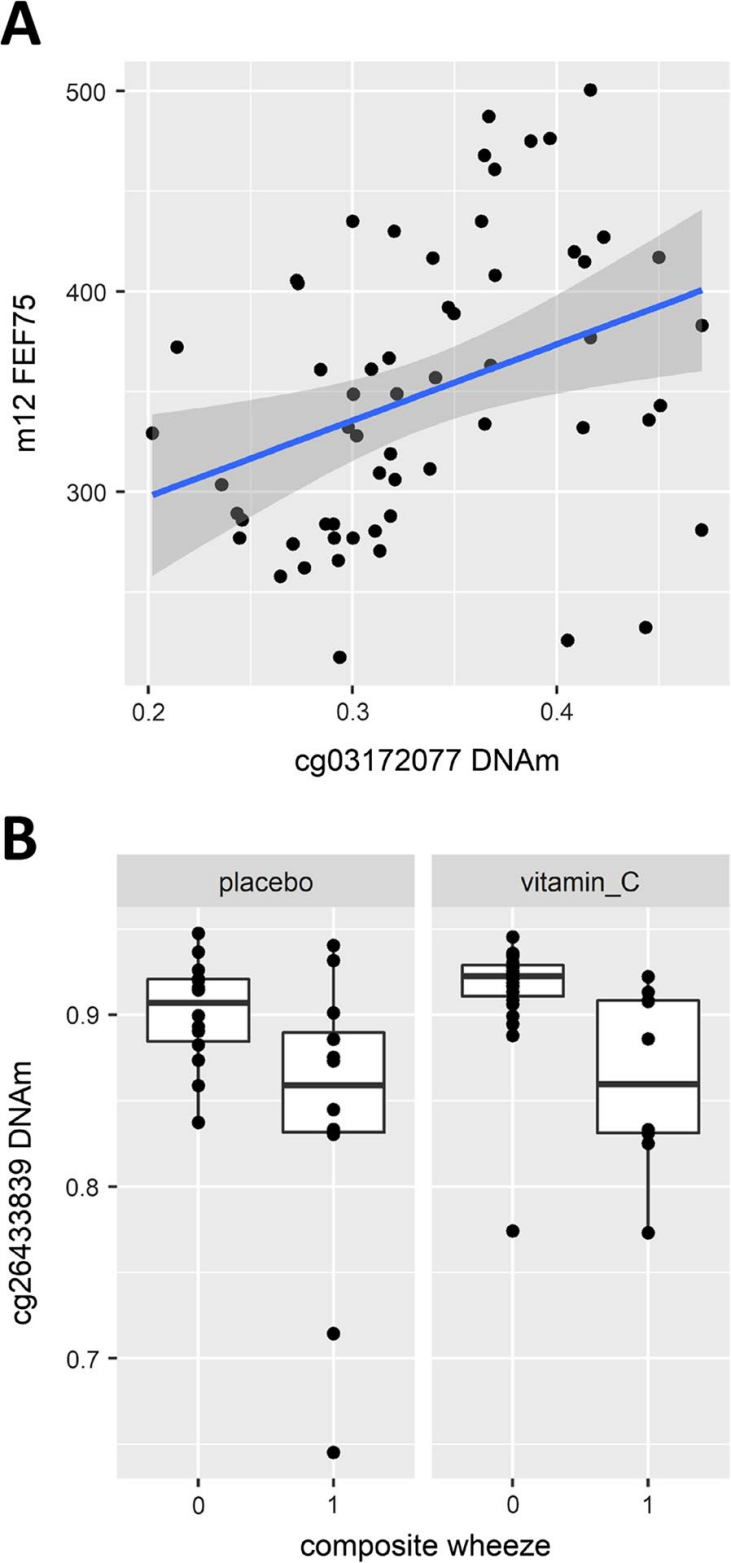
Top CpGs associated with **a** 12 month FEF_75_ and **b** composite wheeze score

We next examined the relationship between DNAm at candidate CpGs with infant lung function (FEF_75;_ the measurement of forced expiratory flows (FEF) at 75% of the expired volume) and wheeze assessed at 12 months of age. Due to a smaller sample size, we considered nominal *p* < 0.05 significant within the candidate CpGs tested for association with outcome. Out of 9059 candidates partially “normalized” with vitamin C supplementation, 1584 CpGs (annotated to 1208 unique genes) were nominally associated (and 34 FDR significant; Additional file 1: Figures S6–S7) with infant lung function after adjustment for infant length at PFT, infant sex, and GA at delivery. Of note, 52 candidate CpGs associated with lung function annotated to *PRKCA,* 18 CpGs annotated to *ADAMTS2*, and 10 CpGs annotated to *FOXP4* were positively associated with FEF_75_ (Additional file 2: Table S2)_._ We also identified 30 genes annotated to CpGs associated with FEF_75_ with known roles in lung development, including 6 genes (*ROR2, SOX9, GATA6, RUNX2, RUNX3,* and *ACTN4*) previously associated with adult lung function (Fig. 4; Additional file 2: Table S2) [61]. The top CpG among candidates associated with FEF_75_ was cg03172077, annotated to *TOP3B* (Fig. 5a).

We also examined association of candidate restored CpGs with composite wheeze as defined by any of the following: parental report of wheeze, healthcare provider diagnosis of wheeze, any bronchodilator or steroids use. A total of 620 candidate CpGs (annotated to 530 unique genes) were nominally associated with wheeze after adjustment for infant length at 12 months of age, infant sex, and GA at delivery (Additional file 2: Table S2). Eighteen wheeze associated CpGs in placenta annotated to 14 unique genes with known roles in lung development (Fig. 4; Additional file 2: Table S2) [61]. The top (and only FDR significant) CpG associated with wheeze was cg26433839, annotated to *PRKCA,* in addition to 47 *PRKCA* CpGs and 6 CpGs annotated to *APOH* (directly upstream of *PRKCA)* found to have nominally significant lower methylation associated with wheeze (Fig. 5b). The majority (42/53, 79%) of wheeze associated CpGs annotated to *APOH/ PRKCA* were also positively associated with FEF_75_ at 12 months of age.

### Replication look-up

Prenatal exposure to MSDP (never-smokers vs placebo-smokers: NP) was associated with placental DNAm at 17 Bonferroni significant CpGs and 726 FDR-significant DMCs (Table 1; Fig. 1b). We compared our results with those of two previous EWAS of MSDP in placenta [13, 62]. In lookup study 1, we focused on the overlap of our results with DMCs associated with sustained MSDP (Everson et al.: Table S6 [62]), since the majority of smokers in our study were persistent smokers throughout pregnancy. Out of all 19,219 CpGs sustained-smoking associated CpGs (FDR < 0.05) identified by Everson et al. [62], 17,039 were represented by probes on the EPIC platform and 4034 were also nominally significant in our comparison of placebo smokers to never-smokers on the beta scale (Additional file 2: Table S5). Within those 4034 CpGs, 3964 (98%) showed a consistent direction of change between smokers and non-smokers in the two datasets (overall correlation = 0.88; *p* < 2.2e−16) and 131 CpGs were nominally restored with vitamin C treatment. Out of 726 FDR NP-DMCs in our study, 105 were FDR significant in the PACE study [62] (correlation = 0.95; *p* < 2.2e−16; Additional file 1: Figure S4A). The second lookup study was from the Gen3G cohort which used the same Illumina MethylationEPIC platform as this study [13]. We replicated 62/71 Gen3G DMCs at nominal significance and 20/71 at FDR significance between placebo smokers and never-smokers with consistent direction and magnitude of effect sizes (overall correlation = 0.99; *p* < 2.2e−16; Additional file 1: Figure S4B).

### Functional enrichment within candidate loci

We next performed enrichment analyses in the 9059 candidate restored CpGs (Fig. 2) to identify biological functions and processes dysregulated with MSDP and improved with vitamin C supplementation. Analysis for enrichment using ConsensusPathDB of the 5613 unique genes annotated nearest to candidate restored CpGs identified enrichment of 156 pathways (*q *value < 0.05; Additional file 2: Table S6) and 461 GO-terms (*q *value < 0.05; Additional file 2: Table S7). The top enriched pathway and GO-terms were “neuronal system” (*q *value = 2.3E−08) and “nervous system development” (*q *value = 6.8E−36), respectively. Other significant pathways were related to cell signaling (i.e., *PI3K-AKT-mTOR, Wnt, VEGFA-VEGFR2, Hippo, GPCR, ERBB2, MAPK1/MAPK3*), differentiation (ectoderm, neural crest), insulin secretion and Type II diabetes, growth factors (i.e., *VEGFs*, *FGFs*, *EGFR1*), calcium regulation in the cardiac cell, extracellular matrix, and many others. Overrepresented GO-terms were related to development, morphogenesis, and embryogenesis, as well as growth factor signaling. For context, we also performed CPDB pathway analysis using the “Top 9059 NP DMCs,” regardless of whether they were restored with vitamin C. The top smoking associated pathways in this study included “nervous system development,” “axon guidance,” and “neuronal system” (Additional file 2: Table S6). We next used IPA software to identify upstream regulators enriched among candidate CpGs associated with MSDP and blunted by vitamin C supplementation. Top upstream regulators of candidate restored CpGs included *ESR1* (*p* = 1.1E−11; 447 target molecules in dataset), *CREBBP* (*p* = 3.85E−10; 150 targets), and *TGFB1* (*p* = 1.35E−09; 567 downstream targets; Additional file 2: Table S8).

### Expression quantitative trait methylation (eQTM) at differentially methylated loci

We investigated the possible impact of DNA methylation changes on the expression of mRNA using RNA-seq data available from 71 placentas used in EPIC methylation analysis. To this end, we calculated the residuals for both the CpG beta-values and logcpm mRNA expression regression analyses adjusted for infant sex, cell type composition, gestational age at delivery, and RNA batch. We then tested the association between methylation residuals of candidate CpGs (described in methods) with flanking (± 250 kb) mRNA residuals. Out of 10,010 candidate CpGs tested, we identified 432 total significant associations with at least one mRNA transcript (at FDR < 0.05). The 432 FDR significant eQTM associations included 357 unique CpGs within 250 kb of transcripts annotated to 268 unique gene regions (Additional file 2: Table S9). The top significant eQTM in candidate restored CpGs was cg02283691 at chr19:33182526, annotated to *NUDT19*, and was negatively associated with the expression of *NUDT19* (*p* = 1.34E−19; *β* = − 3.41; SE = 0.27; Additional file 1: Figure S5A)*.* Notably, we identified several non-DMR genes from our candidate list of restored CpGs with multiple significant eQTM CpGs including 8 CpGs annotated to *LOXL2* and positively associated with *ENTPD4* mRNA. Within the 93 VP-DMRs we identified 89 eQTM CpGs, annotated to 21 unique gene regions (FDR *p* < 0.05; Table 3). Nine of the VP-DMRs contained more than one significant eQTM CpG inversely associated with one or more mRNA transcripts including *PCK2 (13 CpGs), IRF7 (11 CpGs), IVD (11 CpGs), ZNF214/ZNF215 (11 CpGs), LINC00526/LINC00667 (7 CPGs), LRRC4 (5 CpGs), ZNF85 (4 CpGs), AL161785.1 (3 CpGs),* and *ETF1P1 (2 CpGs)*. Further, four PV-DMR regions contained more than one positive eQTM association located nearest *COL21A1 (8 CpGs), ZFP57 (4 CpGs), RNF39 (2 CpGs)*, and *TMEM105* (2 CpGs).

**Table 3:** Vitamin C versus Placebo DMCs located within DMRs and associated with expression levels of mRNA

Probe ID and location					eQTM						Mean across DMR		
Probe	Nearest gene	Chr	Start	Genomic region	Ensembl GeneId	RNA-seq GeneName	Coeff	SE	*P* value	FDR adj.P.Val	∆*β* PN	∆*β* VP	% Restored
cg08757348	*ZNF214*	11	7041565	5'UTR-island	ENSG00000149054	*ZNF215*	− 50.90	6.34	1.77E−11	8.12E−08	0.00	0.00	− 274.61
cg10885961	*LINC00667*	18	5238589	Body-island	ENSG00000263753	*LINC00667*	− 2.20	0.33	3.56E−09	5.44E−06	0.01	− 0.08	542.67
cg00684824	*COL21A1*	6	56112024	5'UTR-shore	ENSG00000124749	*COL21A1*	2.89	0.46	2.70E−08	3.10E−05	0.02	− 0.05	255.48
cg00433866	*MOG*	6	29623646	TSS1500-opensea	ENSG00000204644	*ZFP57*	15.20	2.47	4.14E−08	3.76E−05	− 0.04	0.06	136.70
cg04395593	*C9orf106*	9	132044732	Body-opensea	ENSG00000224307	*AL161785.1*	− 2.44	0.41	7.18E−08	4.12E−05	0.04	− 0.08	180.49
cg21175940	*RNF39*	6	30038910	Body-island	ENSG00000204618	*RNF39*	23.58	4.28	5.72E−07	1.88E−04	0.00	− 0.01	− 230.29
cg14948785	*NLRP14*	11	7041870	1stExon-island	ENSG00000149054	*ZNF215*	− 47.35	8.76	8.81E−07	2.70E−04	0.00	0.00	− 192.77
cg24384195	*PIK3CG*	7	106505370	TSS1500-shelf	ENSG00000105851	*PIK3CG*	− 9.05	1.68	9.86E−07	2.83E−04	0.00	0.01	− 146.10
cg22384902	*SND1*	7	127671261	TSS1500-island	ENSG00000128594	*LRRC4*	− 5.64	1.08	1.66E−06	4.23E−04	0.02	− 0.02	84.88
cg07652350	*IRF7*	11	616009	TSS1500-island	ENSG00000185507	*IRF7*	− 1.53	0.30	2.92E−06	6.30E−04	0.03	− 0.06	196.36
cg02378269	*IVD*	15	40697591	TSS200-shore	ENSG00000128928	*IVD*	− 0.97	0.20	7.74E−06	1.27E−03	− 0.05	0.05	100.42
cg04328729	*DIP2C*	10	521830	Body-shelf	ENSG00000151240	*DIP2C*	2.06	0.44	1.12E−05	1.56E−03	− 0.01	− 0.03	− 294.15
cg08201311	*OBSL1*	2	220435003	1stExon-island	ENSG00000124006	*OBSL1*	− 1.83	0.40	2.31E−05	2.74E−03	− 0.01	− 0.06	− 526.37
cg11479689	*NRL*	14	24563183	TSS1500-shore	ENSG00000100889	*PCK2*	− 1.10	0.25	3.04E−05	3.37E−03	− 0.09	0.06	68.11
cg15467148	*PCK2*	14	24563578	1stExon-island	ENSG00000100889	*PCK2*	− 1.26	0.28	3.26E−05	3.49E−03	− 0.08	0.06	82.03
cg06173720	*LRRC4*	7	127670993	5'UTR-shore	ENSG00000128594	*LRRC4*	− 6.02	1.40	5.26E−05	4.66E−03	0.01	− 0.01	87.33
cg23370394	*BAHCC1*	17	79410470	Body-shore	ENSG00000185332	*TMEM105*	6.19	1.57	1.85E−04	1.21E−02	− 0.02	0.02	101.82
cg16316672	*LINC00526*	18	5237581	TSS1500-shore	ENSG00000264575	*LINC00526*	− 3.04	0.80	3.29E−04	2.07E−02	0.01	− 0.01	101.38
cg25464210	*ZNF85*	19	21106002	TSS200-opensea	ENSG00000105750	*ZNF85*	− 0.88	0.23	3.56E−04	2.18E−02	− 0.07	0.07	106.16
cg15676302	*DMTN*	8	21909754	TSS1500-shelf	ENSG00000208037	*MIR320A*	3.95	1.07	4.64E−04	2.68E−02	− 0.03	0.02	82.05
cg08092680	*TCL1A*	14	96181044	TSS1500-shore	ENSG00000182512	*GLRX5*	2.66	0.77	9.56E−04	4.93E−02	0.01	− 0.01	122.95

Shown here is the top FDR significant CpG per DMR associated with expression, sorted by *p *value

eQTM = expression quantitative trait loci. Nearest gene = nearest proximal gene using the chromosome and positions (GrCh37/hg19) provided for each probe in the Illumina HumanMethylationEPIC annotation file, matched to the nearest gene symbol using the GenomicRanges package in R; Chr = Chromosome; Start = Position (hg19); Ensembl GeneId = associated transcript within ± 250 kb of the CpG site; RNA-seq GeneName = annotated to Ensembl with Biomart; Coeff = coefficient from eQTM association; SE = standard error; ∆*β* PN = delta beta placebo versus nonsmokers; ∆*β* VP = delta beta vitamin C versus placebo smokers; percent restored = (− ∆*β* VP/∆*β* PN) * 100

Among the 17 CpGs associated with MSDP at FDR significance in placebo versus never-smokers, 3 were eQTMs after FDR multiple testing correction. Notably, the top eQTM associated with MSDP was at cg03313447, annotated to *CCDC97,* and was strongly associated with decreased expression of nearby *TGFB1* (*β* = − 11.0; FDR adjusted *p* = 6.94E−08; Additional file 1: Figure S5B). The top CpG associated with MSDP, cg27402634, was not significantly associated with expression of flanking genes including *LEKR1* (*β* = − 0.41; FDR adjusted *p* = 0.65; Additional file 1: Figure S5C); however, cg20385913 located in the body of *CYP1A2* was associated with expression of *CYP1A1* mRNA (*β* = 35.7; FDR adjusted *p* < 0.01); Additional file 1: Figure S5D).

## Discussion

This study of placental DNA methylation nested within the VCSIP RCT [53] provides suggestive evidence for partial or full restoration of 9059 CpGs associated with MSDP in placentas from smokers randomized to vitamin C versus placebo. We observed consistently lower placental DNAm among smokers supplemented to placebo versus never-smokers and versus vitamin C supplemented smokers (6691 CpGs hypomethylated, 2368 CpGs hypermethylated) and identified 21 candidate restored DMRs in addition to 268 unique DMC genes associated with mRNA expression using eQTM analysis. Importantly, a subset of candidate CpGs dysregulated with MSDP and normalized with vitamin C were associated with FEF_75_ and/or composite wheeze, assessed only in offspring born to pregnant smokers at 12 months of age.

Only one CpG, cg20790161, located in an intergenic region 17.5 Kbp upstream of *CD28* reached epigenome-wide significance in the comparison of randomized treatment groups. However, there was no significant difference between placebo smokers and non-smokers, and we found no previous reports for differential methylation or functional relevance at this locus. Therefore, we have focused our discussion on candidate restored DMCs and DMRs nearest genes with multiple significant associations in downstream analyses (Fig. 4; Additional file 2: Table S2), with known association with lung development and function [61], and/or literature connections to biological processes and pathways enriched among candidate loci. The first gene candidate supported by multiple lines of evidence is *DIP2C* (disco-interacting protein 2 homolog C). We identified 2 DMRs hypomethylated in placebo vs never-smokers and restored with vitamin C located across the intergenic CpG island and shore regions upstream of *DIP2C*. Additionally, we identified 6 restored candidate DMCs nominally associated with lung function, and 1 restored DMC (cg27315601) associated with both wheeze and lung function. Furthermore, 4 DMCs located in the body of *DIP2C* were significantly associated with *DIP2C* expression (Additional file 1: Figure S8).

The DIP2 family members (*DIP2A*, *DIP2B*, and *DIP2C*) are highly conserved, and DIP2B and DIP2C are both expressed in human lung and placenta (Human Protein Atlas available from http://www.proteinatlas.org) [63]. Transcriptome profiling in lungs from Dip2a^−/−^ versus wild-type mice revealed dysregulation of genes critical to vasculogenesis, alveologenesis, and branching morphogenesis [64], while loss of Dip2b in mice results in embryonic lethality due to abnormal lung development [65, 66], suggesting a likely role for *DIP2* members in human lung development. Further, an EWAS of lung function in a Korean COPD cohort identified one significant DMC in *DIP2C* (cg03559389) associated with FEV1/FVC ratio, strengthening the potential relevance of this gene in lung development and function [67].

We also identified six DMRs restored with vitamin C in gene regions associated with both lung function and wheeze that were not associated with changes in mRNA expression in the placenta (3 DMRs in *PRKCA,* 1 DMR in *APOH, TMEM174/FOXD1,* and *SSPO*). Within *PRKCA* (Protein kinase C alpha type) we identified 3 restored DMRs and 41 candidate CpGs that were nominally associated with both lung function and wheeze. *PRKCA* is involved in the regulation of critical pulmonary and cardiovascular processes including angiogenesis [68], vascular endothelial barrier function [69], platelet function [70], arterial blood flow [71], cardiac hypertrophy [72], and endothelial cell migration and adhesion [73]. Increased expression of *PRKCA* in pulmonary artery smooth muscle cells from smokers is associated with increased pulmonary artery wall thickness [74]. Moreover, *PRKCA* has been suggested as a positional candidate for the shared genetic predisposition to asthma and obesity [75], and in utero exposure to polycyclic aromatic hydrocarbons from both ambient sources and MSDP has been previously reported as a risk factor for both asthma and obesity in early life [76].

Directly upstream of *PRKCA* is the *APOH* (Apolipoprotein H) gene, also known as beta-2-glycoprotein I (β2GPI). We identified one DMR (6 CpGs) annotated to *APOH,* similarly hypomethylated in placentas from placebo-smokers compared to placentas from both never-smokers and vitamin C-smokers. Interestingly, β2GPI is expressed in placental syncytiotrophoblasts and extravillous trophoblasts, and one of the key targets for antiphospholipid antibodies (aPL) that are associated with adverse pregnancy outcomes such as intrauterine growth restriction (IUGR), preeclampsia, and recurrent miscarriage [77]. Additionally, β2GPI is associated with hypoxia in endothelial cells, and β2GPI-derived peptides have been tested for therapeutic potential in limiting tumor growth by regulating angiogenesis [78].

*FOXD1* (forkhead box D1) belongs to the forkhead family of transcription factors and regulates gene expression in a wide variety of biological processes including kidney morphogenesis and retinal development. Moreover, Foxd1 expressing progenitor cells play a role in lung development and lung fibrosis [79]. *FOXD1* mutations have been implicated in obstetric complications including preeclampsia, IUGR, repeated implantation failure, and recurrent pregnancy loss through regulation of endometrial and placental genes [80]. We identified a restored DMR located in the intergenic region between *TMEM174* and *FOXD1* that spanned ENCODE regulatory motifs, in addition to candidate restored DMCs outside the DMR positively associated with respiratory outcomes (Additional file 3; Additional file 1: Table S2). One CpG was correlated with *FOXD1* mRNA expression (*r* = 0.244; *p* = 0.039) in crude analysis but did not reach FDR significance in the adjusted eQTM analysis. In all, these findings suggest that DNA methylation loci dysregulated by MSDP in placenta and restored with vitamin C supplementation are involved in biological processes critical to angiogenesis and embryonic morphogenesis that are highly relevant to both placental function and lung development.

We examined the consistency of our results with those from two previous EWAS of MSDP in placenta (Additional file 2: Table S5). The top CpG associated with MSDP in our study, cg27402634, was also the top DMC in previous reports [13, 21] and we observed a similar large magnitude of difference between placebo-smokers and never-smokers (− 31 ± 2%). Contrary to previous reports, we did not observe increased expression of *LEKR1* mRNA associated with cg27402634 (*r* = − 0.19; *p* = 0.09). The absolute value of effect sizes for MSDP were, on average, greater in our cohort than in a meta-analysis of sustained MSDP in placental DNAm [62], suggesting potentially increased duration and magnitude of exposure in our population. This is not surprising given that our randomized clinical trial consisted of pregnant smokers unable to quit smoking, compared with prospective birth cohorts with lower prevalence of MSDP and potentially a lower proportion of heavy smokers. Other possible explanations for differences in effect size include differences in sample size, probe efficiency between the MethylationEPIC and 450 K platforms, maternal demographics (i.e., race and age), and overall health status.

We further identified novel loci in the comparison of placebo-treated smokers to never-smokers and in the candidate restored CpGs, possibly due to differences in overall health status and prevalence of smoking in our clinical trial population compared to population-based studies, with limited information on smoke exposure level and vitamin C status. Across both novel and replicated CpGs, the majority were hypomethylated in smokers versus non-smokers. A large proportion of replicated loci associated with MSDP and not reversed by vitamin C were dose-dependently associated with level of exposure, based on maternal cotinine measurements performed throughout pregnancy [81].

Enrichment analysis of genes nearest candidate “normalized CpGs” identified many of the same pathways previously reported to be associated with sustained MSDP [62] (Additional file 2: Table S6). The top three pathways associated with sustained MSDP [62] and normalized or improved with vitamin C included “calcium regulation in the cardiac cell,” “VEGFA-VEGFR2 signaling,” and “Wnt signaling”. Placental vasculogenesis is regulated by a number of growth factors and aberrations in this process are a common theme identified in pregnancy complications such as intrauterine growth restriction and preeclampsia [82]. Dysregulated angiogenesis is also associated with pulmonary complications such as broncho-pulmonary dysplasia, pulmonary hypertension, and COPD [83]. Therefore, vitamin C supplementation to pregnant smokers may restore the balance of angiogenic factors in the placenta, in parallel with changes in the developing lung, in order to elicit the measured effects on lung function in our RCT.

To confirm the potential importance of these methylation changes on lung development and function, we examined our results for genes with known roles in lung development, based on a list of 391 genes compiled by Portas et al. Out of 391 known lung development genes, 126 were listed among our candidate restored DMCs (Additional file 2: Table S2), and 36 were associated with infant lung function and/or wheeze within RCT participants (Fig. 4) [61]. We speculate that although DNA methylation profiles are largely tissue specific at the CpG level, maternal smoking during pregnancy disrupts an overlapping set of critical developmental and homeostatic pathways across fetal and placental tissues through epigenetic mechanisms. This is supported by a previous study showing overlap of nicotine associated methylation changes between placenta and fetal lung collected in early development [84]. Future studies are necessary to validate these findings in additional populations and in animal models, and to identify the specific mechanism(s) whereby altered DNA methylation by vitamin C in the placenta influences infant lung growth and development.

Our findings in this study, combined with a previous study of placental hemodynamics and transcriptome analysis in this cohort [56], suggest that altered placental methylation and gene expression may mediate changes in vasculature and angiogenic signaling in response to MSDP and that these changes may be blunted by supplemental vitamin C during pregnancy. Our results are supported by the overall consistency of our findings with previous EWAS studies of MSDP and by histological studies in placentas from smokers which report increased collagen deposition, increased thickness of the villous membrane, vascular remodeling, as well as reduced intervillous area and capillary volume [27]. Importantly, as these reported histological changes in the placenta are similar to changes observed in blood vessels from offspring of smokers [85, 86], the mechanism by which vitamin C improves placental blood flow in smokers may parallel the widespread effects of MSDP on pulmonary and cardiovascular development.

Our study is the first to use the MethylationEPIC array (over 850,000 CpGs) for association with placental DNAm in a majority smoking (and RCT) population with extensive exposure measurements collected at multiple time-points throughout pregnancy. To our knowledge, the only previous EWAS of placental DNAm with MSDP measured on this platform was from the Genetics of Glucose Regulation in Gestation and Growth Study (Gen3G), which included 403 non-smokers and 38 participants with self-reported smoking during pregnancy [13]. We are also the first to combine epigenome-wide placental DNAm association with MSDP and direct measurements of childhood lung function.

The primary limitation to our study is our available sample size, which was underpowered to detect small differences in methylation at the majority of loci [87]. As we anticipated that our sample size may be insufficient to detect epigenome-wide significance between placebo and vitamin C treated smokers, we used a concept-driven analysis approach with the overall goal to identify candidate loci that may be “normalized” with vitamin C supplementation toward the level of never-smokers. We also performed DMR analysis using comb-p for consistency with previous studies [55]. However, comb-p has been recently reported to have greater Type 1 error, also known as “higher false positives,” than other DMR calling methods, and therefore, these candidate regions require further validation. An additional limitation is that these results represent only a subset of placentas available from the parent RCT. In the parent RCT, we collected 210 placentas from pregnancies of smokers at delivery and selected a subset of available placentas using a blocked- randomization design after exclusion of placentas from pregnancies complicated by preeclampsia, preterm delivery, gestational hypertension, and placentas collected outside the 3-h window post-delivery (Additional file 1: Figure S2). However, our previous transcriptome-wide analysis of 80 placentas (60 RCT smokers and 20 never-smokers for reference) also suggested activation of vasculogenesis, endothelial tissue development, and response to growth factors and we confirmed expression of genes critical to these processes by RT-qPCR in the larger RCT population [56]. Although these findings are suggestive and require replication, given that we focused on the overlap of nominally significant CpGs and a less stringent DMR method, our results are supported by consistency with prior studies of smoking-associated placental DNA methylation and downstream analyses demonstrating association of DNAm with placental gene expression and infant lung function.

## Conclusions

This epigenome-wide analysis of placental DNA methylation within a randomized clinical trial population of pregnant smokers identified candidate loci associated with vitamin C supplementation. Critically, some of the treatment associated CpGs were also associated with infant lung function and wheeze measured at 12 months of age. These findings suggest the potential for vitamin C supplementation to mitigate negative consequences of MSDP on placental gene expression through epigenetic mechanisms.

## Methods

### Study design

This study was nested within a multi-center, double-blind RCT that demonstrated improved airway function at 3 and 12 months of age in offspring whose mothers were randomized to supplemental vitamin C (500 mg/day) versus placebo [53, 54, 58]. For the current study, we analyzed 96 placentas and prioritized samples used previously in transcriptome analysis [56]. We excluded placentas from subjects with gestational hypertension, preeclampsia, and preterm delivery (< 37 weeks), and placentas sampled more than 3 h after delivery (Additional file 1: Figure S2).

### Study population

The parent RCT recruited women with singleton pregnancies (≥ 15 years old; < 23 weeks gestation) with a history of current smoking and documented refusal/inability to quit. Women were randomized to receive vitamin C versus placebo after a successful run-in trial for medication compliance that required 75% adherence and return for follow-up within 7–21 days. Randomization to vitamin C or placebo was blocked in rotations of two and four subjects, and stratified by gestational age at randomization (≤ 18 vs > 18 weeks) and site (Oregon Health & Science University [OHSU], Portland, Oregon; PeaceHealth Southwest Washington Medical Center [SWW], Vancouver, Washington; Indiana University [IU], Indianapolis, Indiana). A total of 252 pregnant smokers were randomized and 243 infants were available for study at delivery. The RCT was approved by each site’s Institutional Review Board and monitored by an NIH appointed Data Safety Monitoring Board. A group of 33 pregnant never-smokers were enrolled toward the end of the RCT as a reference group for an ancillary study of placental blood flow, histology, and molecular biomarkers (Additional File 1: Figure S2). We obtained written informed consent from all subjects prior to enrollment [58].

### Statistical analysis of patient demographics

Normally distributed variables are expressed as mean and standard error and compared for group differences using an unadjusted F-test. Non-normally distributed variables are expressed as median and interquartile range (25th–75th percentile), and the Wilcoxon rank sum test was used to compare groups. Chi-square test was used to compare categorical variables. Significance was defined as *p* < 0.05.

### Placental DNA methylation (DNAm) acquisition and pre-processing

Epigenome-wide placental DNAm was measured with the Infinium MethylationEPIC BeadChip (Illumina, San Diego, California) at the Fred Hutchinson Cancer Genomics Resource (Seattle, WA). See Supplemental Methods for details of placental collection, DNA extraction and DNA methylation acquisition (Additional file 1). Data normalization and QC were performed using ChAMP: non-CpG probes, probes with a beadcount < 3 in at least 5% of samples, probes annotated to SNPs [88], probes with a detection *p *value > 0.01 in one or more samples, cross-hybridizing probes [89], and probes on X/Y chromosomes were removed and remaining probes (*n* = 714,666) were normalized via functional normalization.

### Estimate of placental cellular heterogeneity

We used the RefFreeEWAS package to estimate proportions of cell types in placental samples. We used the top 10,000 most variable CpGs from our dataset in the bootstrap to determine the optimal number of cell types. The optimal k for this dataset (*k* = 2) was selected based on the minimal deviance metric. The entire set of filtered CpGs (*n* = 714,666) was then used to estimate the proportions of each cell type.

### CpG annotation to nearest gene

For downstream enrichment analysis we annotated all intergenic CpGs to the nearest proximal gene using the chromosome and positions (GrCh37/hg19) provided for each probe in the Illumina HumanMethylationEPIC annotation file, matched to the nearest gene symbol using the *GenomicRanges* package in R.

### Analysis of differentially methylated CpGs (DMCs) and regions (DMRs)

We performed differential methylation analysis using the lmFit (method = “robust”), contrasts.fit and eBayes functions in limma with methylation for each CpG site as the response variable on the M-scale (logit2 beta) and randomization group as the predictor. Covariates were selected from a list of potential a priori confounders assessed for variance contribution using champ.SVD. We adjusted models for infant sex, gestational age at delivery, and estimated cellular heterogeneity. We used quantile–quantile plots of *p *values to visualize genomic inflation between unadjusted and adjusted models (Additional file 1: Figure S3). We computed estimated coefficients and standard errors for each contrast of interest (never-smoker vs placebo smoker) and (placebo smoker vs vitamin C smoker). We also calculated the coefficients and standard error from *β* value regression for more intuitive biological interpretation and comparison to previous studies. To identify differentially methylated regions (DMRs) we used comb-p [59] using the results from each fully adjusted limma model as input and the following parameters to initiate and extend a region: –seed 0.05 –dist 500. A DMR was considered significant when it included at least 2 probes within a window of 500 bp and the Šidák corrected *p *value was < 0.05.

### Association of candidate DMCs with infant lung function and wheeze

We measured airway function in infants born to smoking participants in the RCT as described previously [53]. Briefly, FEFs were obtained from forced expiratory flow volume curves using the raised volume rapid thoracic compression technique following the American Thoracic Society/ European Respiratory Society criteria for performance and acceptance [90]. The measurement of FEF at 75% of the expired volume (FEF_75_) was defined a priori as the primary outcome in our RCT and was measured at 3 and 12 months of age. A modified form of the International Study of Asthma and Allergies in Childhood (ISAAC) respiratory questionnaire [91] was administered at least quarterly to the infant’s caretaker. Composite wheeze was defined as a positive response to any of the following questions: parental report of wheeze, healthcare provider diagnosis of wheeze or any bronchodilator or steroids use. We used robust linear regression analysis adjusted for infant length at PFT, infant sex, and gestational age (GA) at birth to check for association between candidate loci and lung function or wheeze assessed in infants at 12 months of age. Due to our small sample size, CpGs with unadjusted *p* < 0.05 were considered significant for discussion relevant to lung function or wheeze.

### Replication look-up in previous EWAS

We compared our results with previous findings from two previous EWAS of MSDP associated methylation changes in placenta: (1) a meta-analysis for the association between sustained MSDP and placental DNAm measured on the Illumina HumanMethylation450 BeadChip (Additional file 2: Table S6 [62]), and (2) results from the Gen3G study (Web Table 3 [13]) measured using the same Illumina MethylationEPIC platform as this study. For our comparisons, we utilized the nominally significant results from the contrast of never-smokers versus smokers randomized to placebo and compared beta-value scale coefficients to the beta-coefficients reported previously for MSDP.

### Enrichment analysis of biological pathways and gene ontology (GO) terms

Functional enrichment analyses were performed at the gene level using ConsensusPathDB [92] and Ingenuity Pathway Analysis (Qiagen Inc., MD, USA) [93]. We focused our enrichment analysis on genes annotated nearest to candidate CpGs partially restored in the overlap of nominal *p *values (Fig. 2). ConsensusPathDB performs enrichment analysis using a hypergeometric test, and we report significant pathway and GO_term results after multiple testing correction with FDR < 5%. In IPA, we used the “Core Analysis” pipeline with default settings to test for enriched canonical pathways, upstream regulators, diseases, and functions.

### Expression quantitative trait methylation (eQTM) loci

We performed expression quantitative trait methylation (eQTM) analysis using the *MEAL* package [94] for correlation of expression and methylation. Genome-wide RNA-sequencing was available for 71 placentas (26 placebo, 27 vitamin C, and 18 never-smokers) with MethylationEPIC data (Additional file 1: Figure S2; Additional file 1: Supplemental Methods). We focused our eQTM analysis on CpGs partially restored in the overlap of nominal p-values (Fig. 2), CpGs located in DMRs between placebo and vitamin C (Additional file 2: Table S3), and FDR significant CpGs between placebo and never-smokers (Table 1). Combined, these three sets of candidate CpGs included 10,010 unique CpGs for eQTM analysis. We used the default flanking parameter to identify mRNA transcripts with a transcription start site (TSS) located within 250 kb of each candidate CpG. We calculated the residuals for both the CpG beta-values and logcpm mRNA expression regression analyses adjusted for infant sex, cell type composition, and gestational age at delivery. The association between methylation and expression residuals was performed in 55825 CpG-mRNA pairs and we report statistically significant eQTM with a FDR < 5% (Additional file 2: Table S9).

## Supplementary Information

**Additional file 1. Figures:** Figure S1. Flowchart of the analysis steps; Figure S2. Consort diagram of samples used in analysis; Figure S3. QQ-plot for unadjusted vs adjusted models; Figure S4. Comparison of FDR significant smoking DMCs with previous studies; Figure S5. Top eQTMs associated with vitamin C vs placebo DMRs; Figure S6. Top DMCs associated with m12 FEF75; Figure S7. Heatmap of FDR DMCs associated with m12 FEF75; Figure S8. Visual summary of DIP2C findings. Supplemental Methods.

**Additional file 2. Tables:** Table S1. Population characteristics; Table S2. Candidate restored DMCs and association with outcomes; Table S3. VP-DMRs; Table S4. PN-DMRs; Table S5. Lookup analysis; Table S6. CPDB Pathway; Table S7. CPDB GO terms; Table S8. IPA Top regulators; Table S9. eQTMs.

**Additional file 3.** Gviz plots of significant DMRs between vitamin-C supplemented smokers and placebo. All 93 DMRs are plotted and arranged in alphabetical order based on the nearest annotated gene. Each page represents a single DMR and is organized into multiple tracks showing genomic ranges, CpG island locations, DMR location, and CpG level data tracks for each comparison (placebo vs non-smoker and vitamin C vs placebo).

## Data Availability

The raw and processed DNAm data are publically accessible through NCBI Gene Expression Omnibus (GEO) via accession series  GSE169598.

## Abbreviations

MSDP: Maternal smoking during pregnancy
CpG: Cytosine–guanine dinucleotide
DNAm: DNA methylation
DMC: Differentially methylated CpG
DMR: Differentially methylated region
Doppler-US: Doppler ultrasound
EWAS: Epigenome-wide association study
eQTM: Expression quantitative trait methylation
RCT: Randomized clinical trial
IUGR: Intra-uterine growth restriction
TSS: Transcription start site
FDR: False discovery rate
FEF: Forced expiratory flow
FEF_75_: Forced expiratory flow at 75% of vital capacity
PN: Placebo versus non-smoker
VP: Vitamin C versus placebo
Gen3G: Genetics of Glucose regulation in Gestation and Growth study
PACE: Pregnancy And Childhood Epigenetics
