# Supplementary material for: Impact of vitamin C supplementation on placental DNA methylation changes related to maternal smoking: association with gene expression and respiratory outcomes
Source: Clin Epigenetics. 2021 Sep 19;13:177. doi: 10.1186/s13148-021-01161-y (PMC8451157; doi:10.1186/s13148-021-01161-y)
Supplement: Supplementary file 1 — Additional file 1. Figures: Figure S1. Flowchart of the analysis steps; Figure S2. Consort diagram of samples used in analysis; Figure S3. QQ-plot for unadjusted vs adjusted models; Figure S4. Comparison of FDR significant smoking DMCs with previous studies; Figure S5. Top eQTMs associated with vitamin C vs placebo DMRs; Figure S6. Top DMCs associated with m12 FEF75; Figure S7. Heatmap of FDR DMCs associated with m12 FEF75; Figure S8. Visual summary of DIP2C findings. Supplemental Methods. [file 13148_2021_1161_MOESM1_ESM.pdf]

Figure S1. Flowchart of the analysis steps and summary of results filtering.

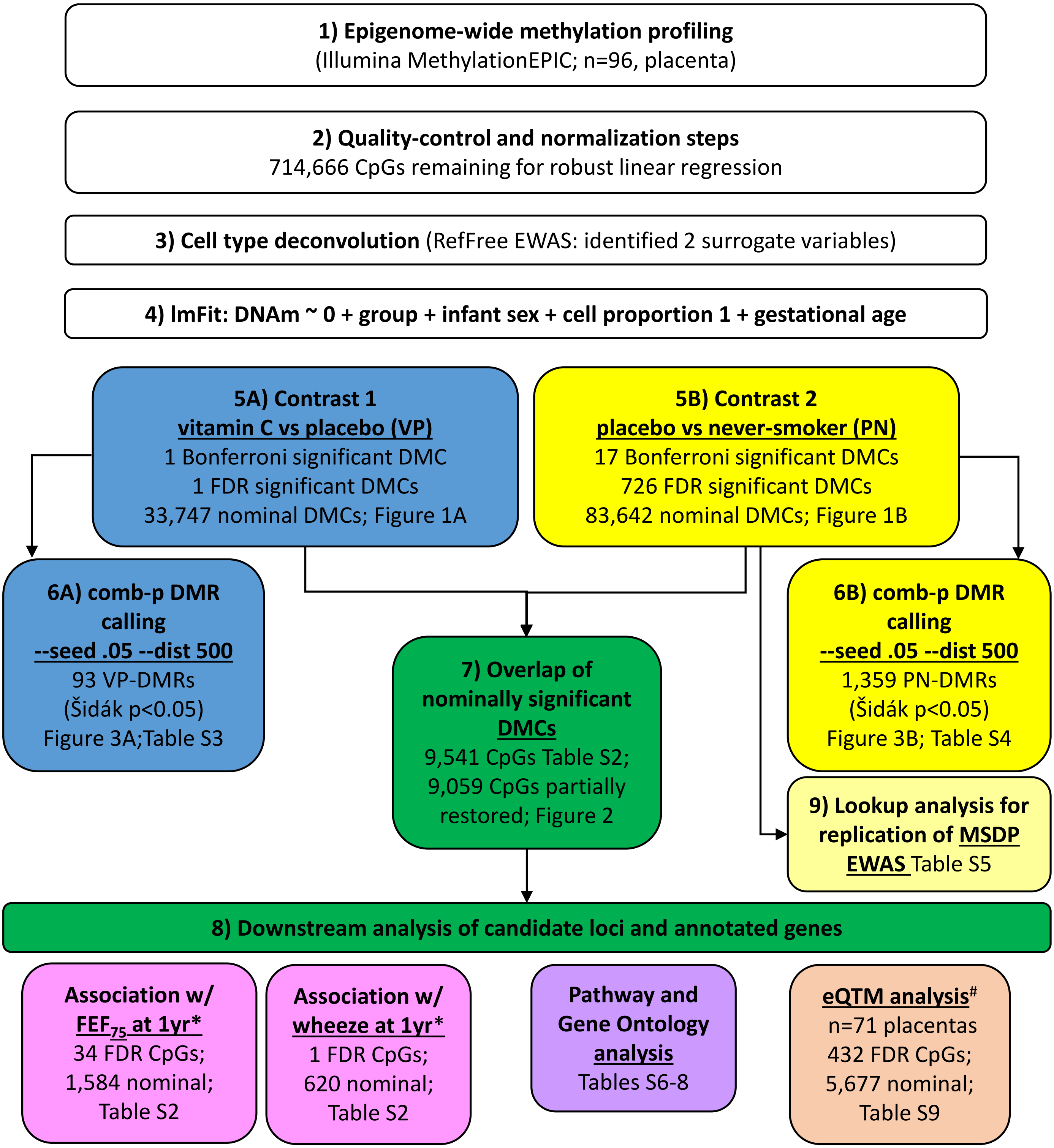

*adjusted for infant length at PFT, infant sex, and GA at delivery

#adjusted for infant sex, cell composition, and GA at delivery

Figure S2. Consort diagram of samples used in placental methylation and gene expression analysis.

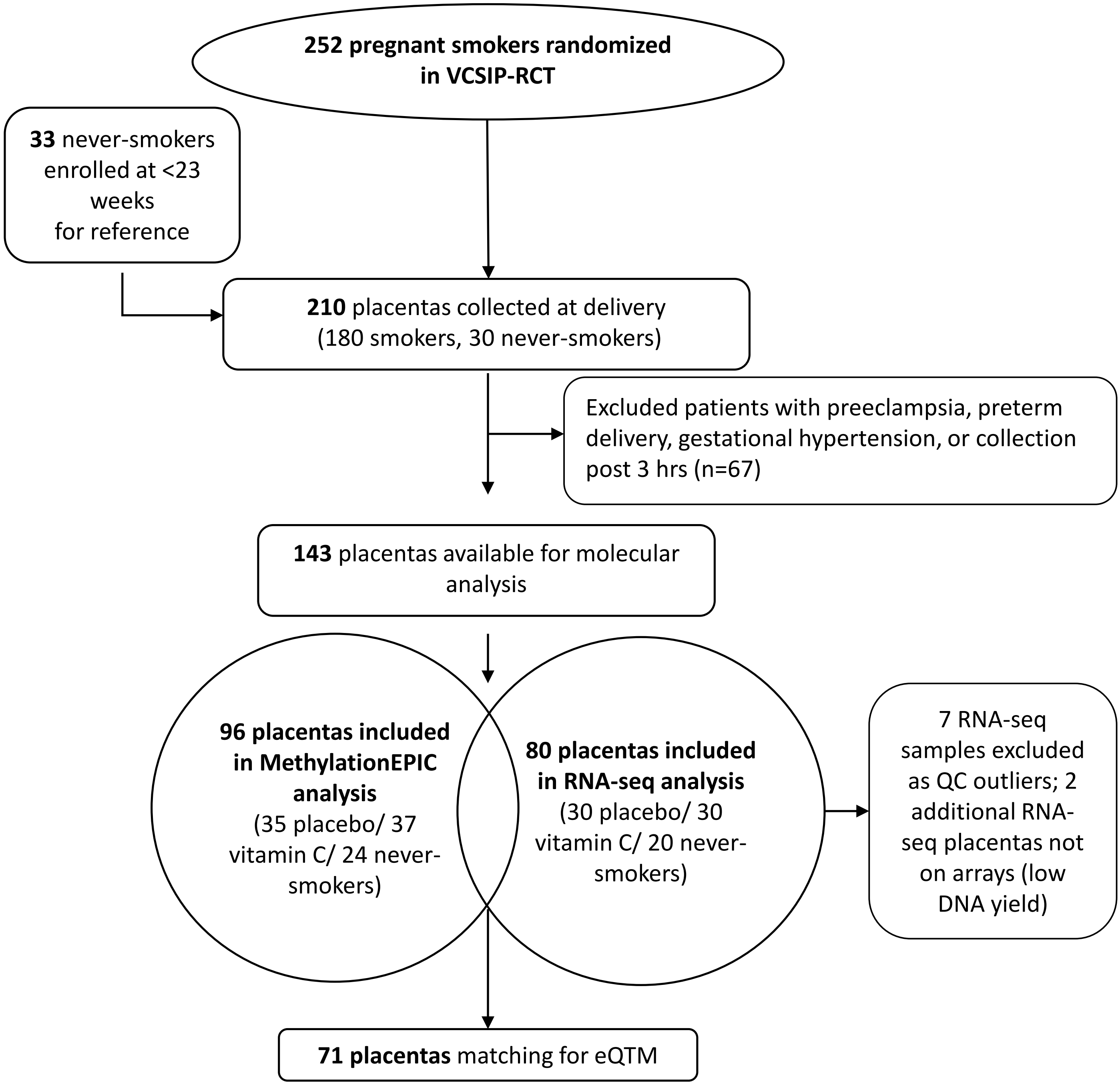

Figure S3. QQ-plots of expected vs actual p-values for associations in placental DNAm in A) Vitamin C vs Placebo and B) Never-smokers vs Placebo in unadjusted vs adjusted models.

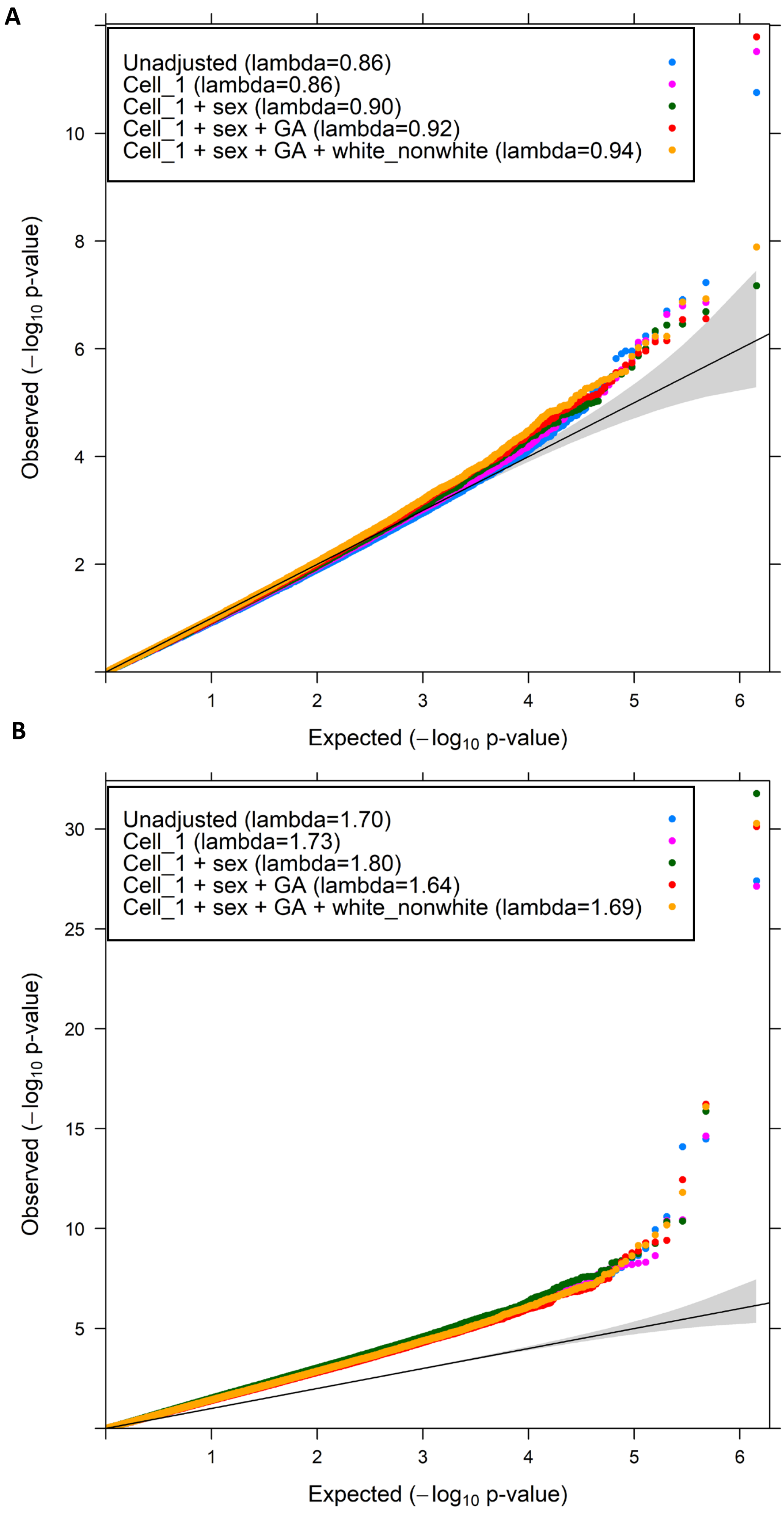

Figure S4. Comparison of FDR significant smoking DMCs with results from **a)** the PACE consortium meta-analysis using the Illumina450K array and **b)** with results from the Gen3G cohort using the same IlluminaEPIC array as in this study.

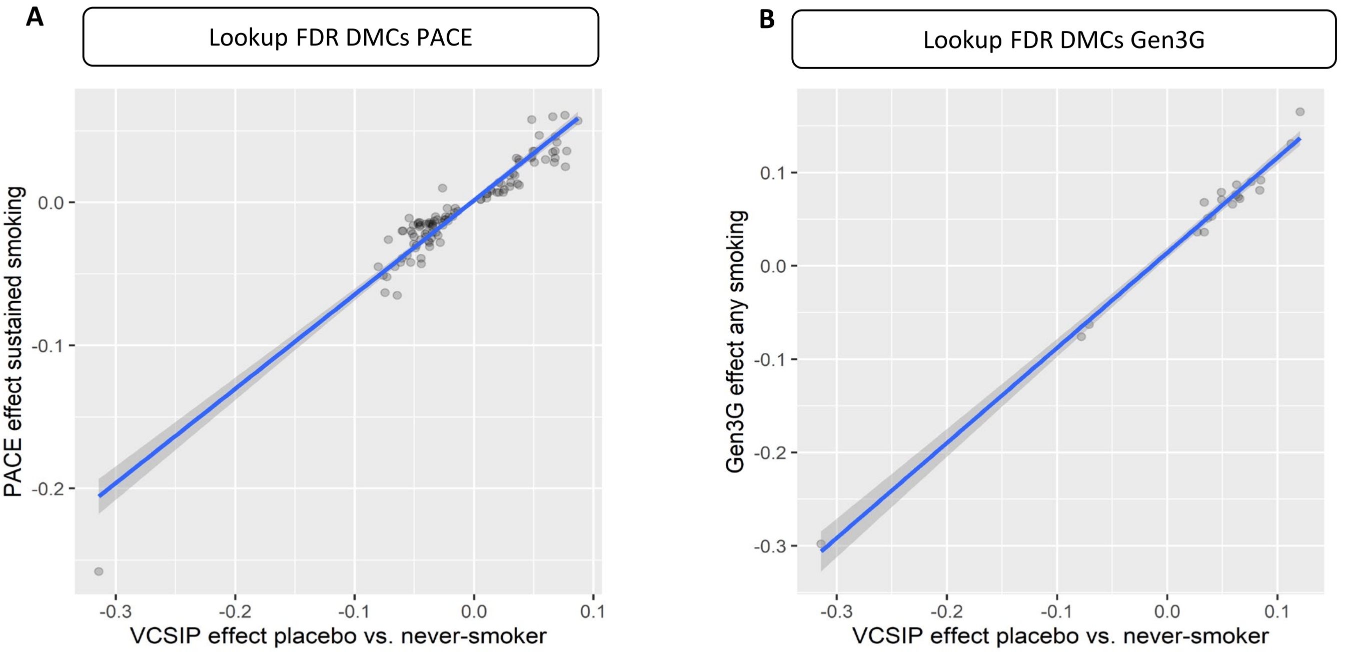

Figure S5. Top eQTMs associated with vitamin C vs placebo DMRs **(a,b)** and smoking status **(c,d)**. Key: green – never smoker; red – vitamin C; black – placebo; mRNA level = log counts per million.

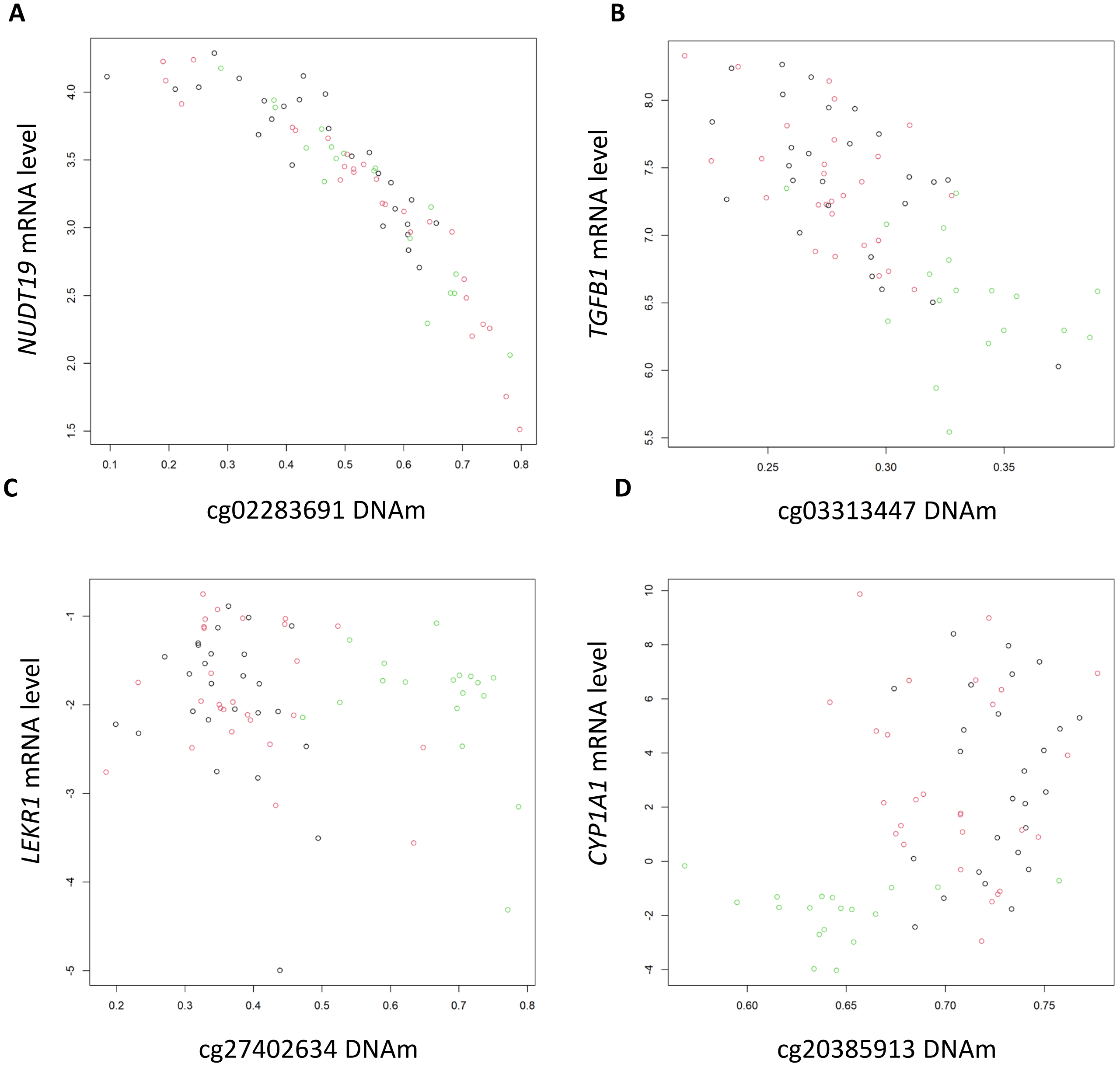

Figure S6. Candidate DMCs associated with m12 FEF_{75} after adjustment for infant length at 12 months of age, infant sex, and GA at delivery. **A-C:** Top 3 candidate CpGs associated with FEF75. **D:** Rows of the heatmap include all candidate restored CpGs (n=9,059). The right column represents the coefficient from the linear regression comparing vitamin C smokers to placebo and the left column is the R value for Pearson correlation with FEF75 at each candidate CpG.

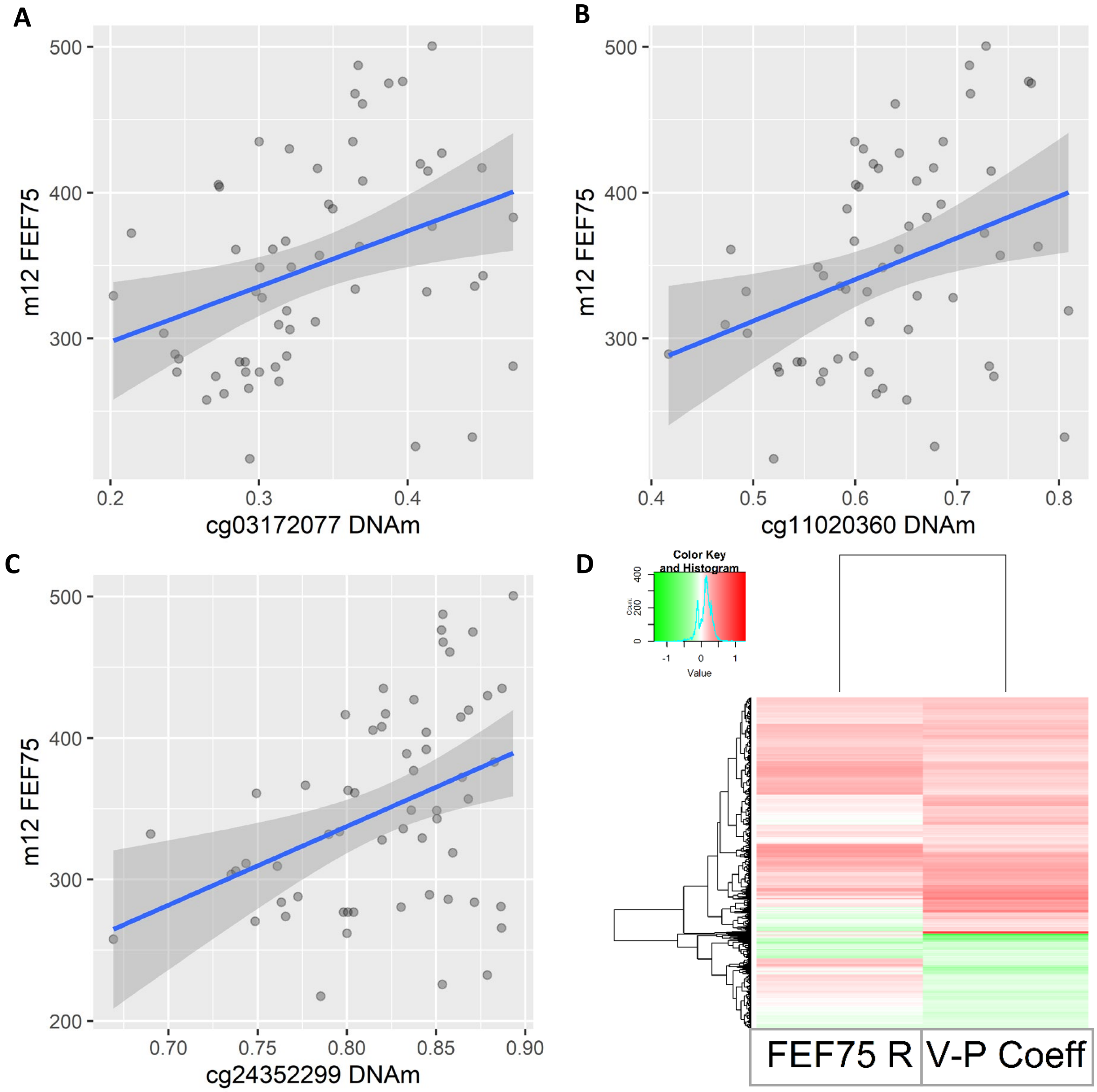

Figure S7. Heatmap of DMCs associated with m12 FEF_{75}. Columns represent individual subjects and are arranged from lowest m12 FEF_{75} to highest. Rows represent FDR significant CpGs after adjustment for infant length at 12 months of age, infant sex, and GA at delivery. Values for each row are mean centered - red indicates higher methylation and green indicates lower methylation.

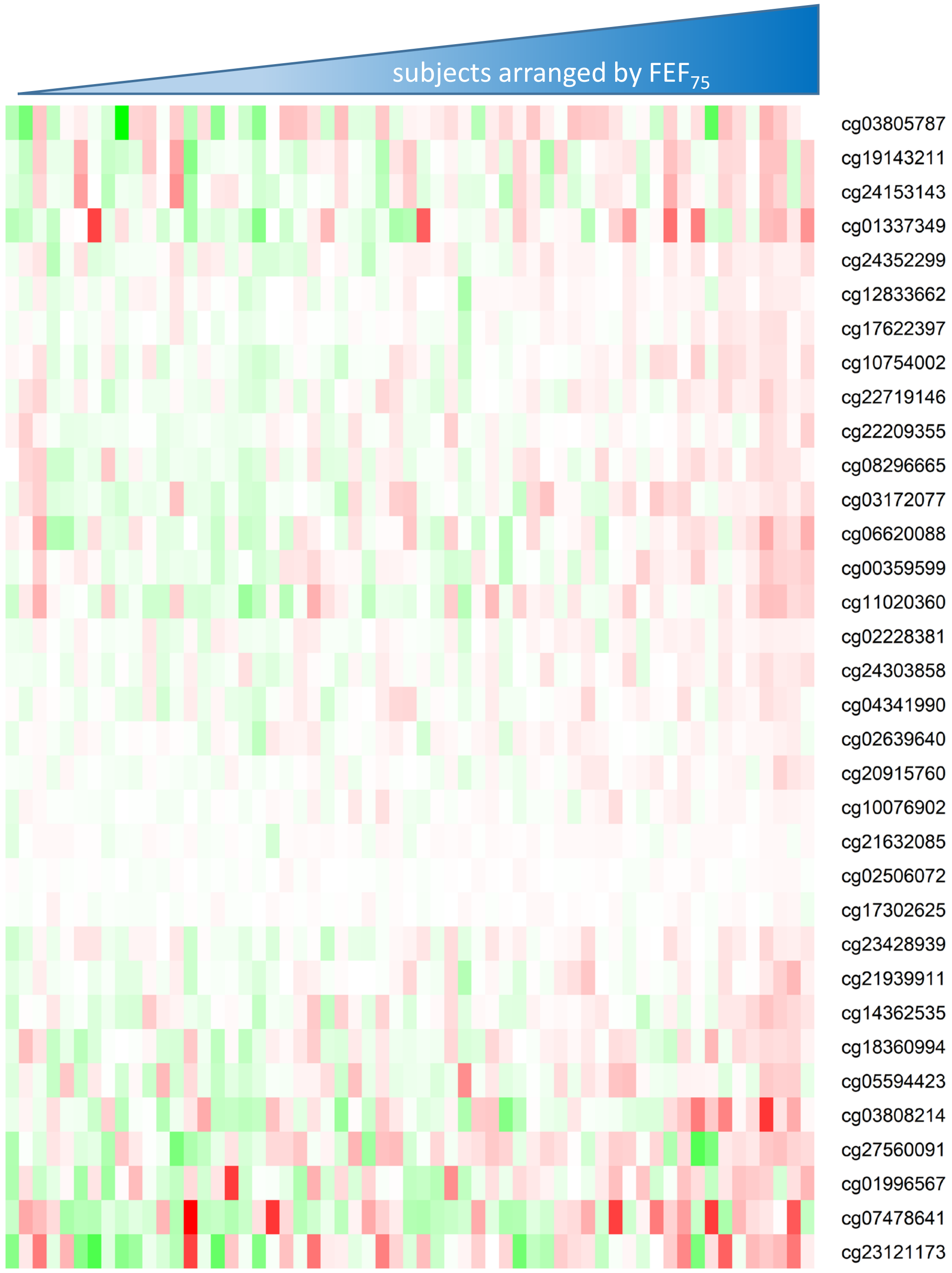

Figure S8. Visual summary of *DIP2C* findings.

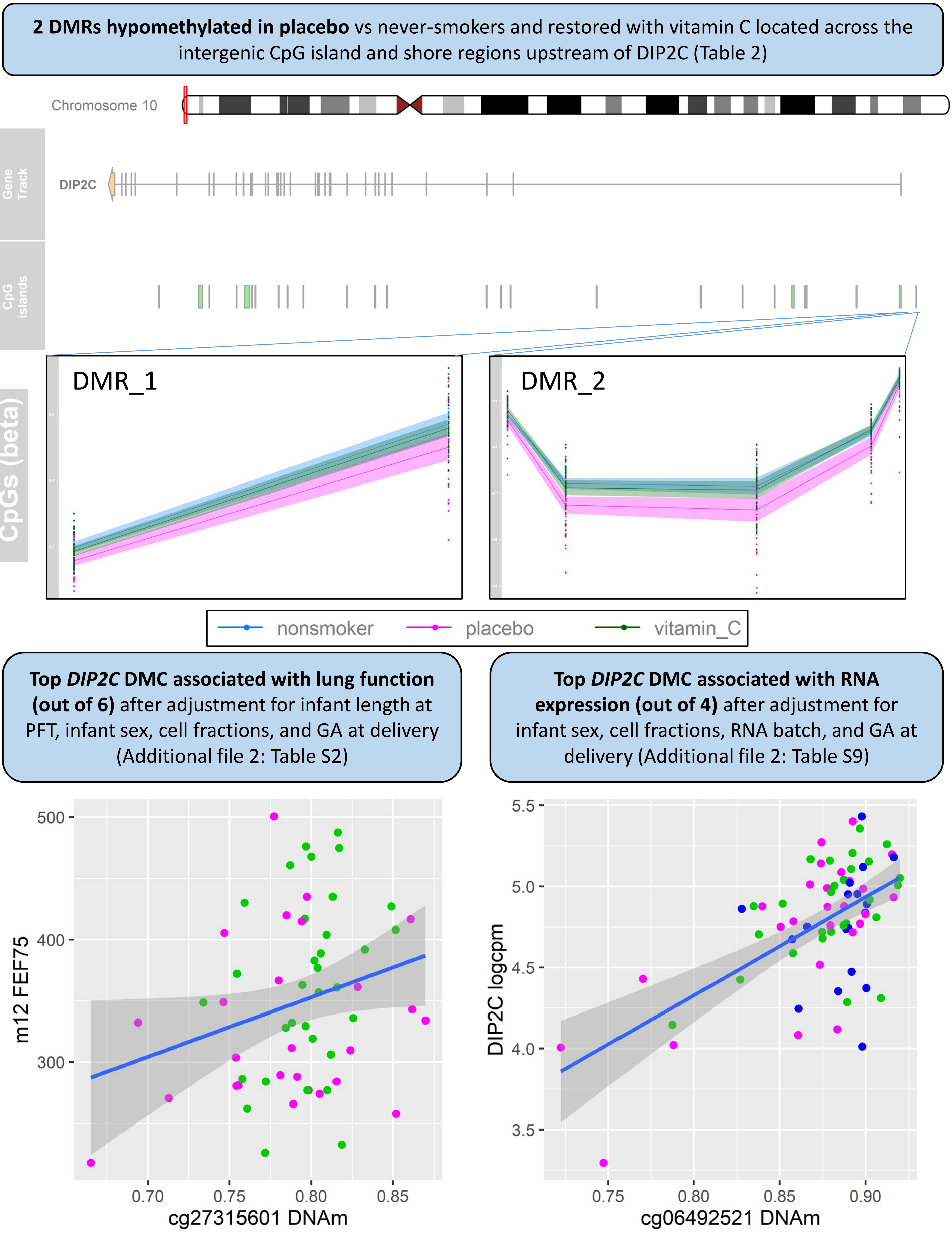

Supplemental Methods:

Collection of placental samples and DNA

Utilizing the biobank of placentas available from the RCT, we sought to identify DNA methylation (DNAm) changes in the placenta linked to functional effects of smoking during pregnancy. We excluded placentas from subjects with gestational hypertension, preeclampsia, and preterm delivery (<37 weeks), and placentas sampled outside the 3 hour collection window (Figure S2). Placentas were collected and processed by trained research staff using a standardized protocol, as described previously [58]. DNA, RNA, and miRNA were simultaneously extracted from placenta, powdered under liquid $N_{2,}$ using the AllPrep miRNA kit (Qiagen) and the QIAcube for automated nucleic acid extraction (Qiagen, USA).

Quantification of DNAm

Placental DNAM was assessed on the MethylationEPIC BeadChips, measuring over 850,000 CpGs at a nucleotide resolution, at the Fred Hutchinson Cancer Research Center Genomics Resource. In brief, 500ng of DNA was bisulfite converted using the EZ DNA Methylation Kit (Zymo Research) following the Illumina-specified instructions. Converted DNAs were applied to Illumina Infinium Methylation EPIC 8-Sample Beadchips following the Infinium HD Methylation 15019521v01 protocol. Processed BeadChips were scanned using the Illumina iScan+ with ICS v3.3.28 and intensity data was extracted with Illumina GenomeStudio software (GenomeStudio v2011.1 with Methylation Analysis Module v1.9.0).

RNA-sequencing and Data Preprocessing/QC

RNA was extracted from one placenta sample per subject, preserved in RNAlater (ThermoFisher, MA, USA), using the Qiagen AllPrep Universal Kit and the QIAcube for automated nucleic acid extraction (Qiagen Inc., MD, USA). Total RNA was sent to MedGenome Inc. (Foster City, CA, USA) for RNA QC, library preparation, and sequencing. cDNA libraries were generated using the Illumina Stranded Total RNA kit (Illumina, CA, USA) and sequenced using 100bp paired-end reads on the HiSeq 2500 platform (Illumina, CA, USA). Each sample was sequenced with >= 60 million reads and quality control of raw sequence performed by FastQC. FASTQ sequence data was preprocessed with Trimmomatic and aligned to GRCh38_Ensembl using STAR stranded alignment to generate a table of counts per transcript. After trimming and alignment additional QC was performed using RNA-SeQC. Raw count data was transformed to log counts per million (logcpm) and transcripts with low read counts removed by requiring a minimum of 0.2 logcpm in >=18 samples, based on the smallest sample group (never-smokers).
