# Supplementary material for: Impact of vitamin C supplementation on placental DNA methylation changes related to maternal smoking: association with gene expression and respiratory outcomes
Source: Clin Epigenetics. 2021 Sep 19;13:177. doi: 10.1186/s13148-021-01161-y (PMC8451157; doi:10.1186/s13148-021-01161-y)
Supplement: Supplementary file 3 — Additional file 3. Gviz plots of significant DMRs between vitamin-C supplemented smokers and placebo. All 93 DMRs are plotted and arranged in alphabetical order based on the nearest annotated gene. Each page represents a single DMR and is organized into multiple tracks showing genomic ranges, CpG island locations, DMR location, and CpG level data tracks for each comparison (placebo vs non-smoker and vitamin C vs placebo). [file 13148_2021_1161_MOESM3_ESM.pdf]

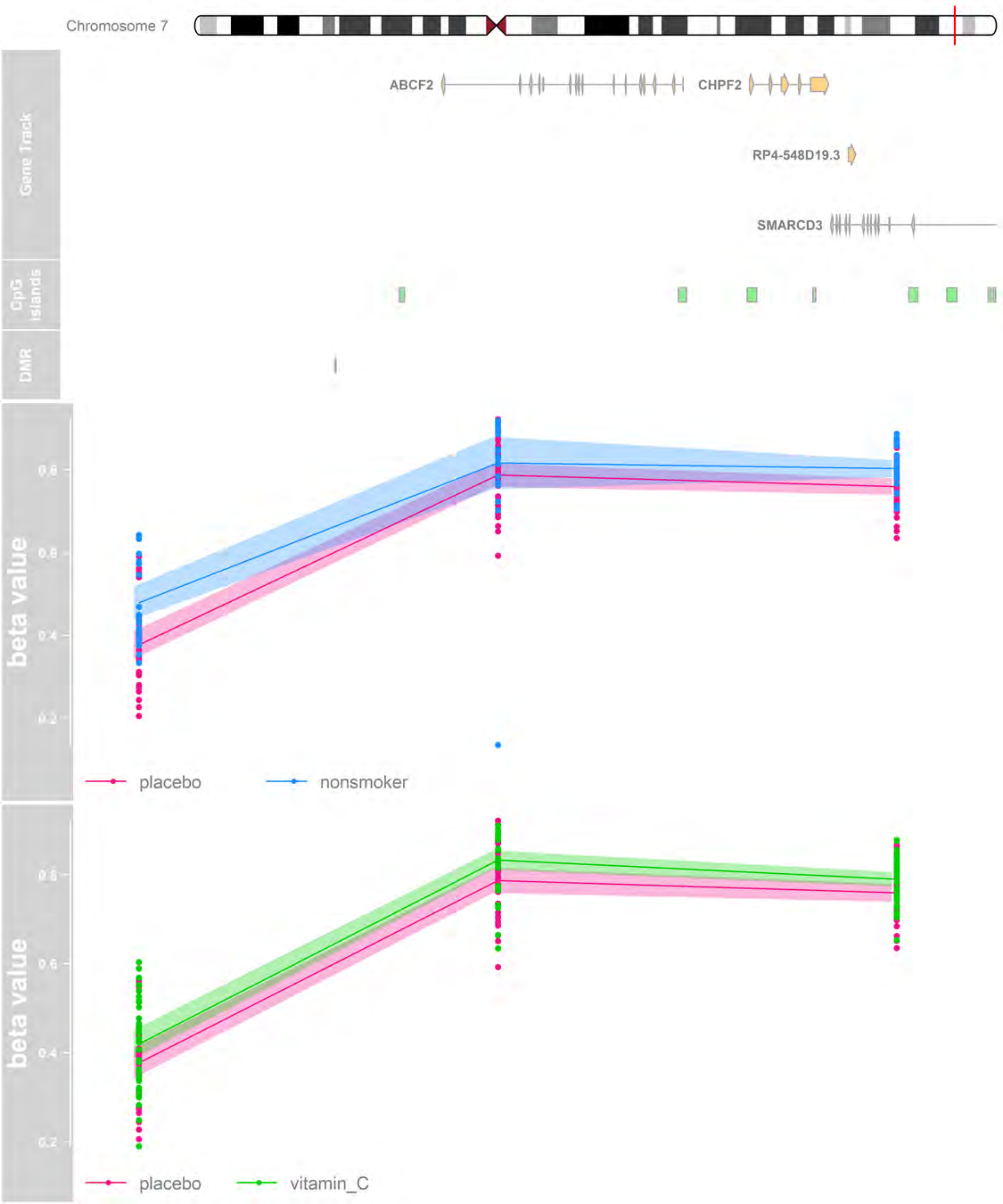

Chromosome 7
Gene Track
ABCF2
CHPF2
RP4-548D19.3
SMARCD3
CpG Islands
DMR
beta value
placebo
nonsmoker
beta value
placebo
vitamin_C

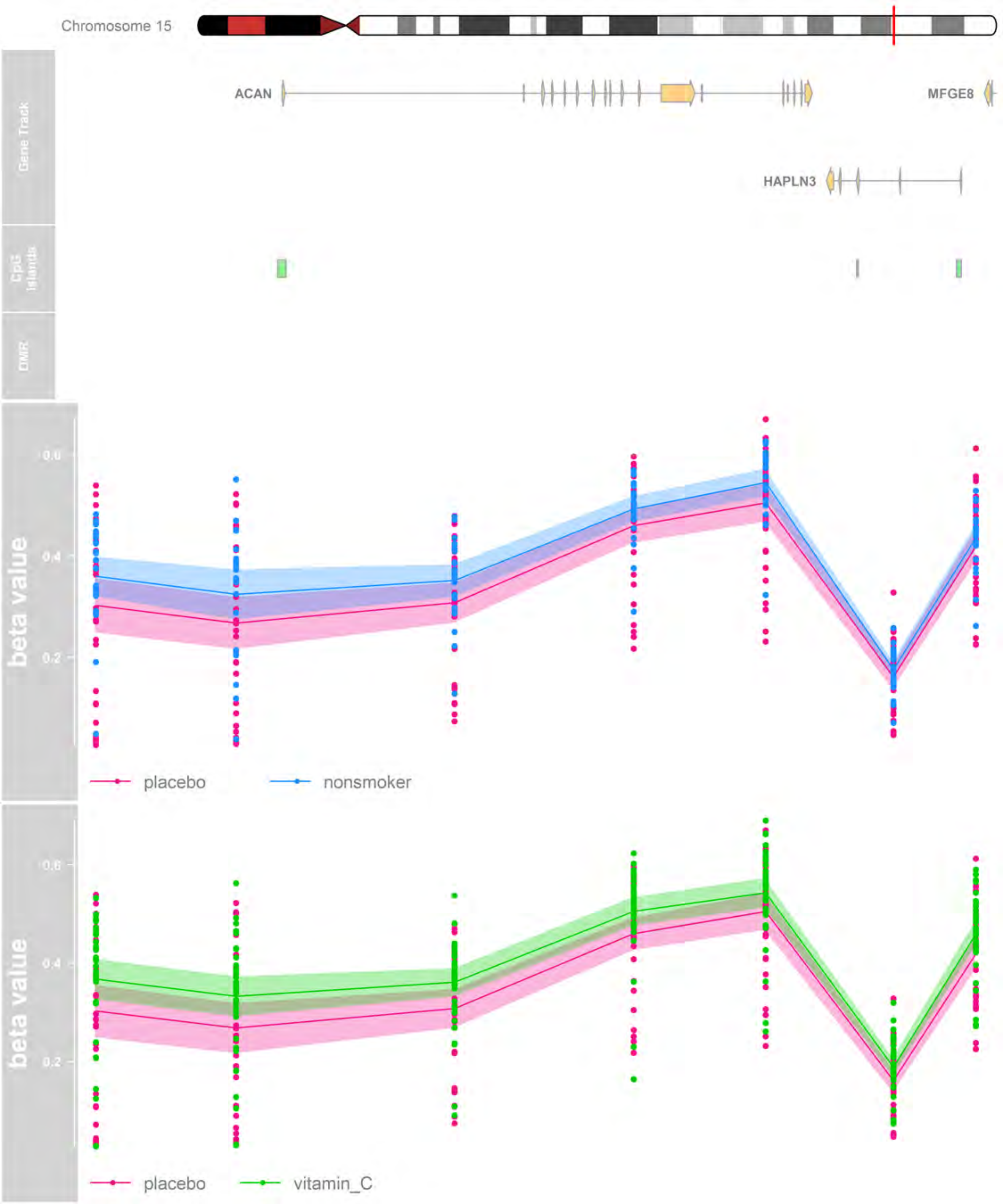

Chromosome 15
Gene Track
ACAN
MFGE8
HAPLN3
CpG Islands
DMR
beta value
0.6
0.4
0.2
placebo
nonsmoker
beta value
0.6
0.4
0.2
placebo
vitamin_C

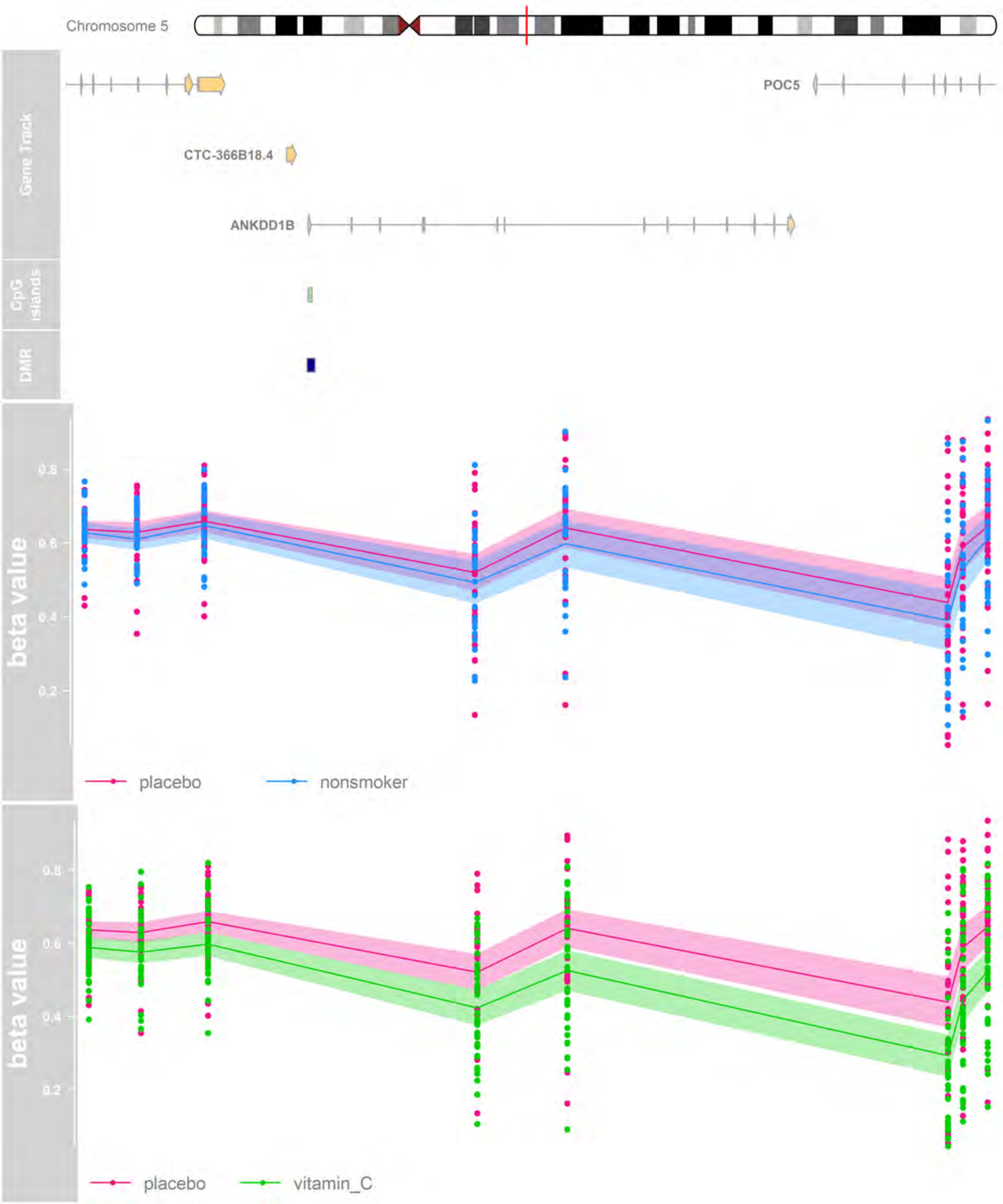

Chromosome 5
Gene Track
POC5
CTC-366B18.4
ANKDD1B
CpG islands
DMR
beta value
0.8
0.6
0.4
0.2
placebo
nonsmoker
beta value
0.8
0.6
0.4
0.2
placebo
vitamin_C

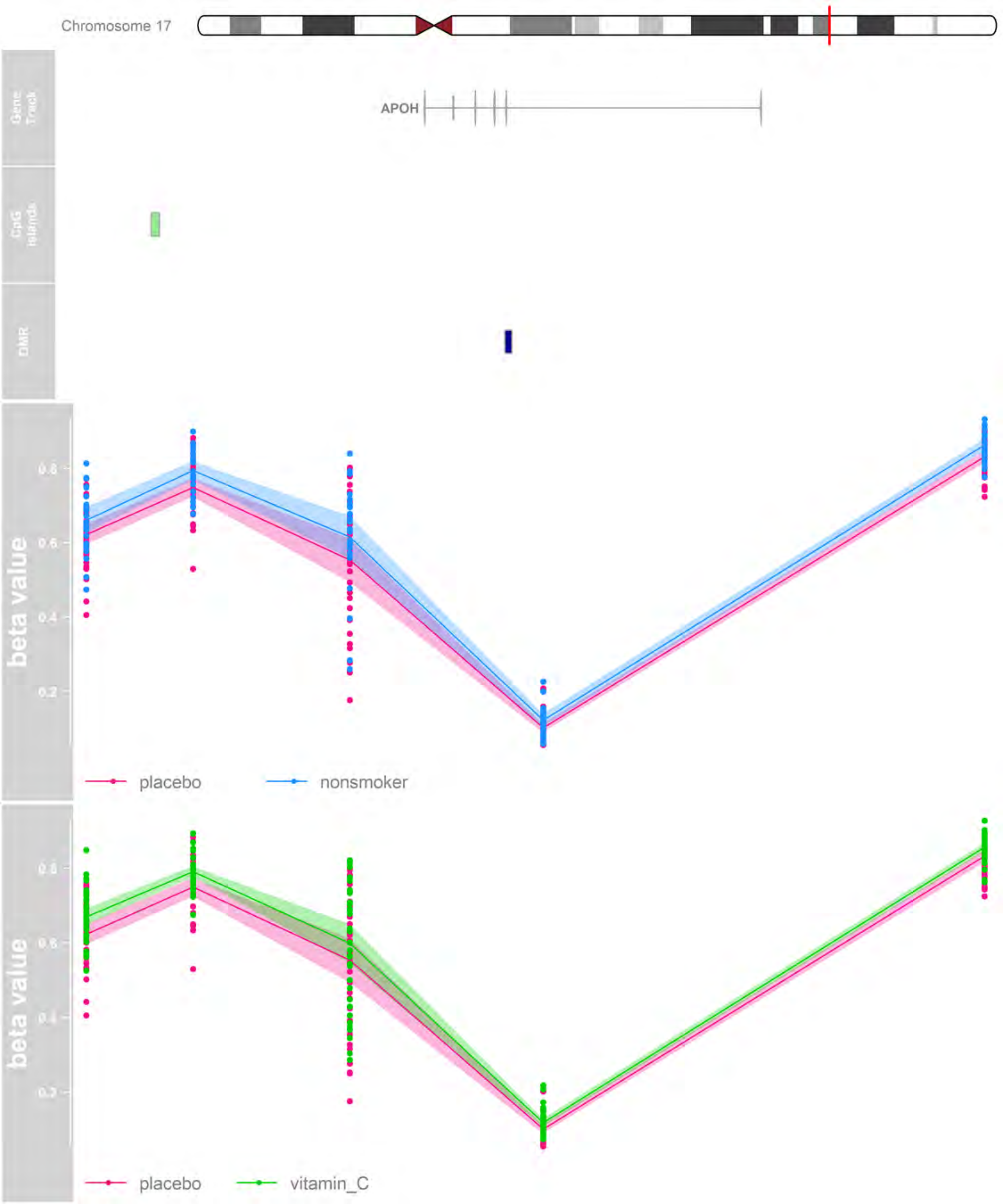

Chromosome 17
Gene Track
APOH
CpG islands
DMR
beta value
0.8
0.6
0.4
0.2
placebo
nonsmoker
beta value
0.8
0.6
0.4
0.2
placebo
vitamin_C

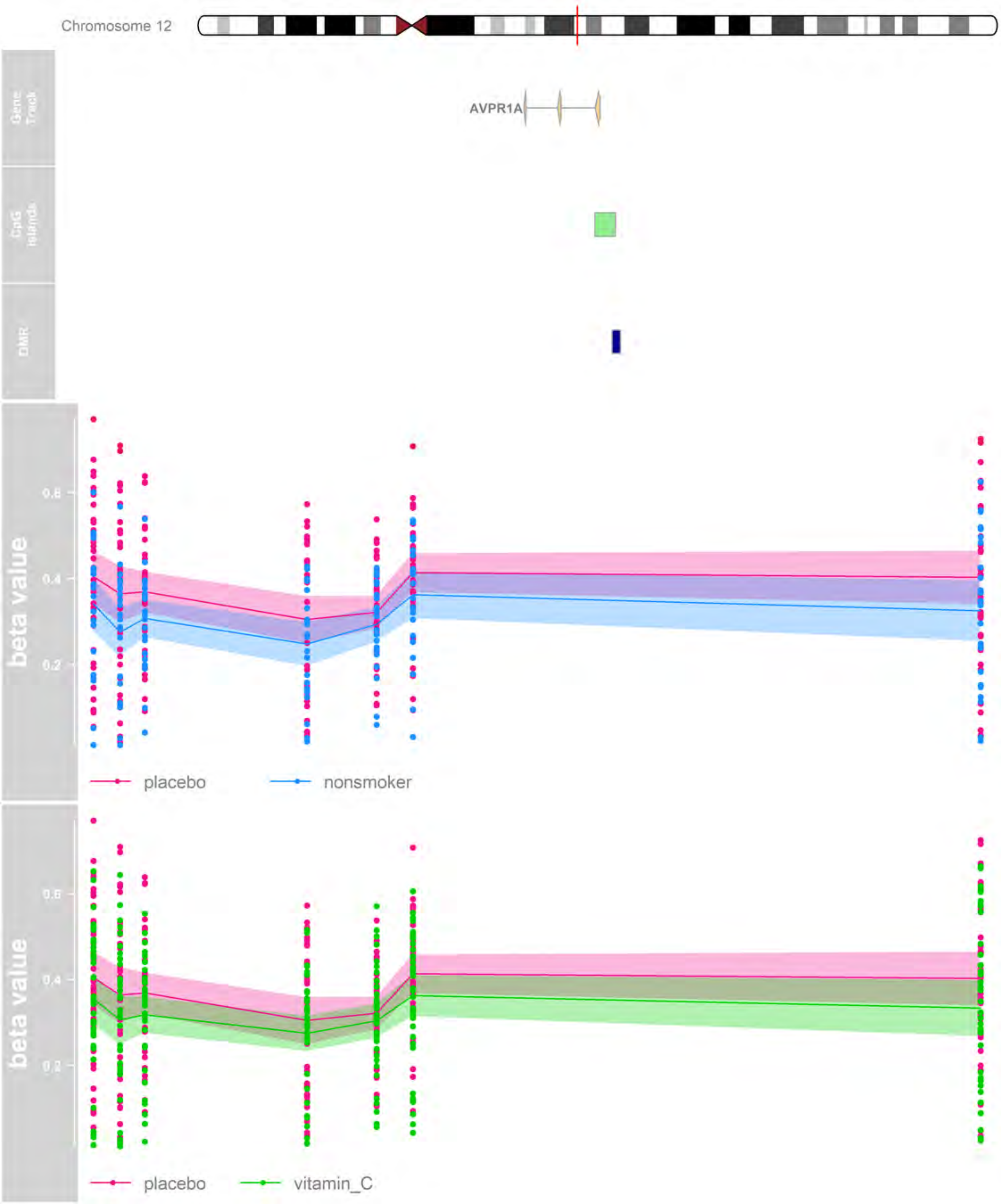

Chromosome 12
Gene Track
AVPR1A
CpG islands
DMR
beta value
0.6
0.4
0.2
placebo
nonsmoker
beta value
0.6
0.4
0.2
placebo
vitamin_C

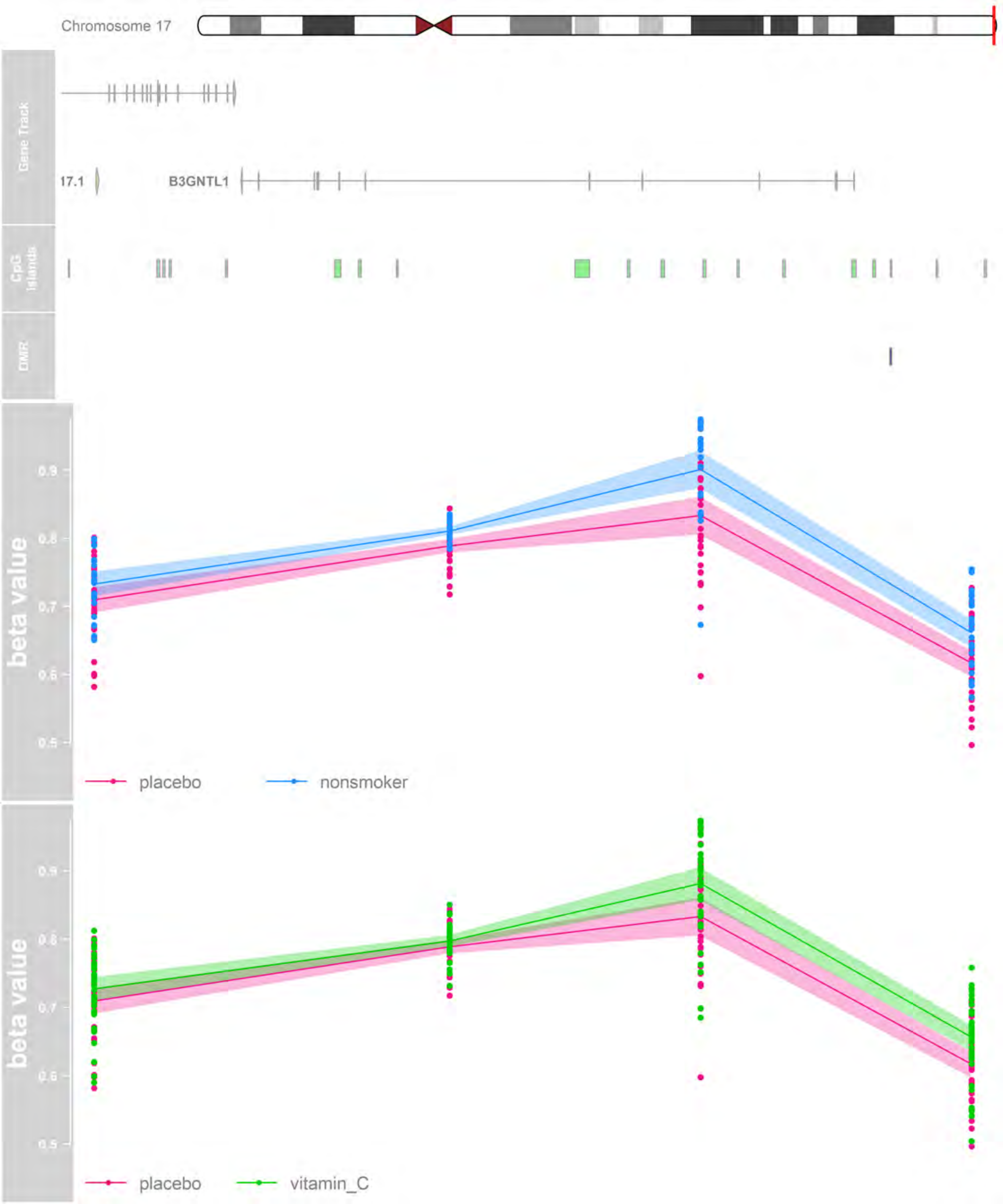

Chromosome 17
Gene Track
17.1
B3GNTL1
CpG Islands
DMR
beta value
0.9
0.8
0.7
0.6
0.5
placebo
nonsmoker
beta value
0.9
0.8
0.7
0.6
0.5
placebo
vitamin_C

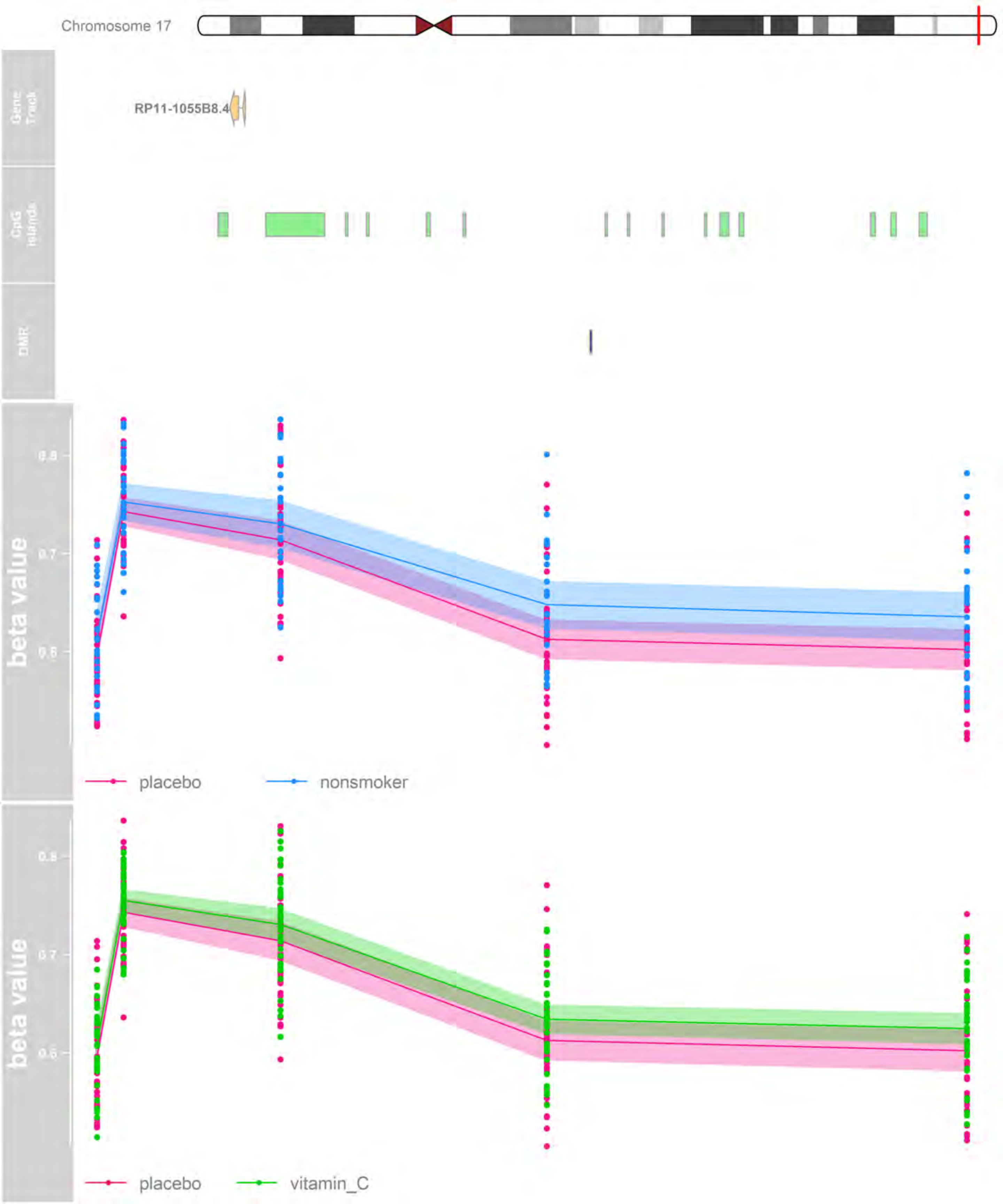

Chromosome 17
Gene Track
RP11-1055B8.4
CpG islands
DMR
beta value
0.8
0.7
0.6
placebo
nonsmoker
beta value
0.8
0.7
0.6
placebo
vitamin_C

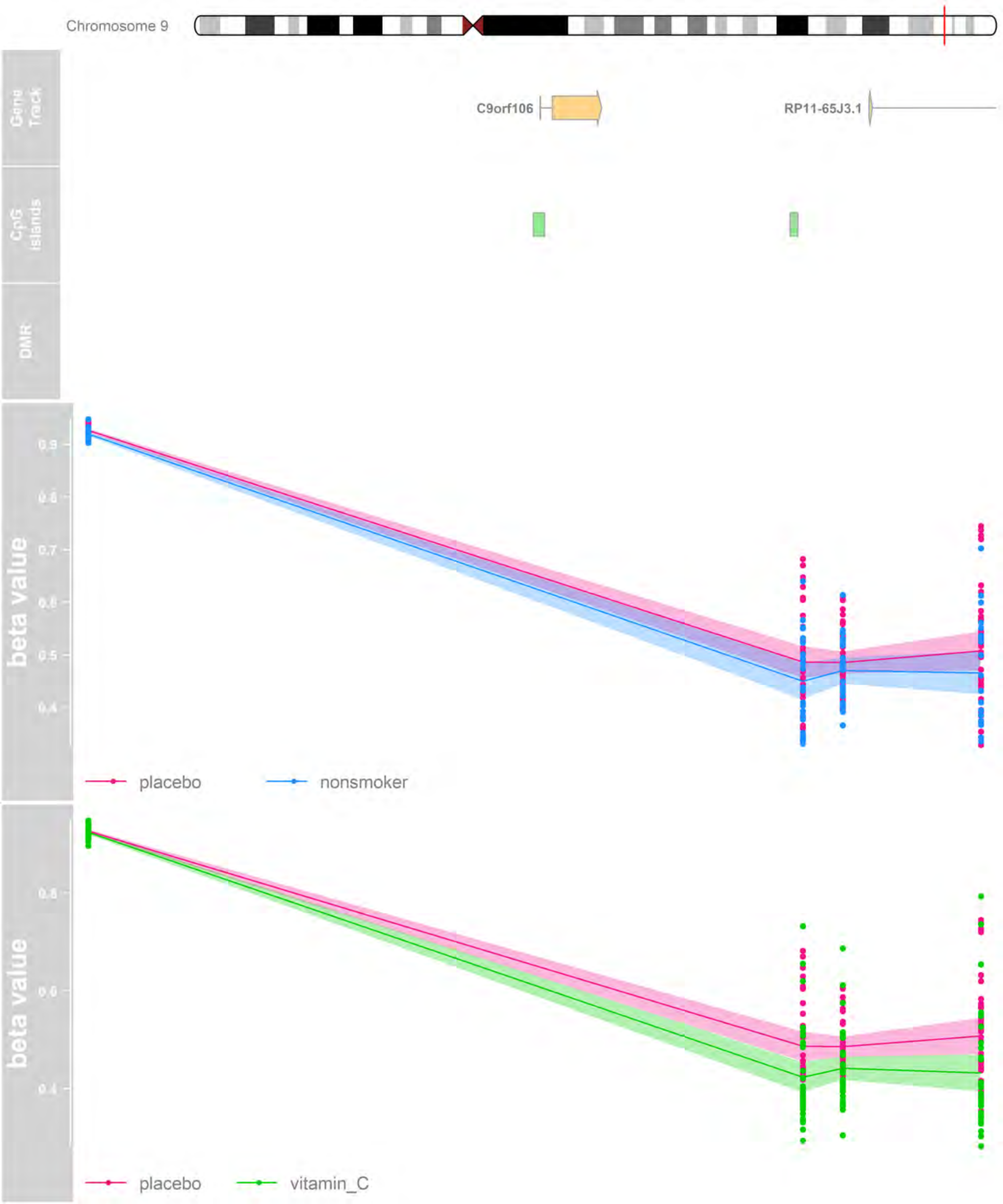
Chromosome 9
Gene Track
C9orf106
RP11-65J3.1
CpG Islands
DMR
beta value
0.9
0.8
0.7
0.6
0.5
0.4
placebo
nonsmoker
beta value
0.8
0.6
0.4
placebo
vitamin_C

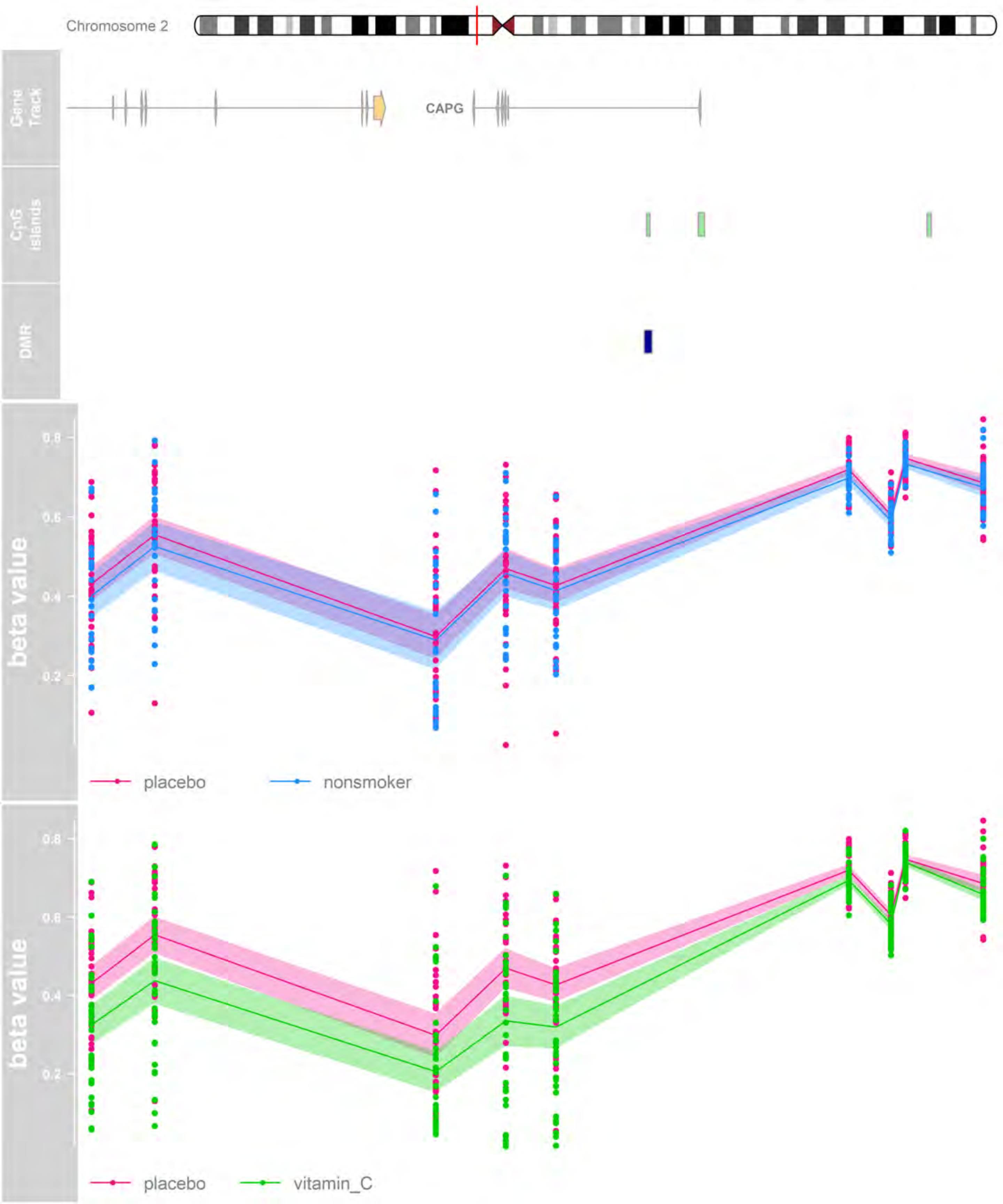

Chromosome 2
Gene Track
CAPG
CpG Islands
DMR
beta value
0.8
0.6
0.4
0.2
placebo
nonsmoker
beta value
0.8
0.6
0.4
0.2
placebo
vitamin_C

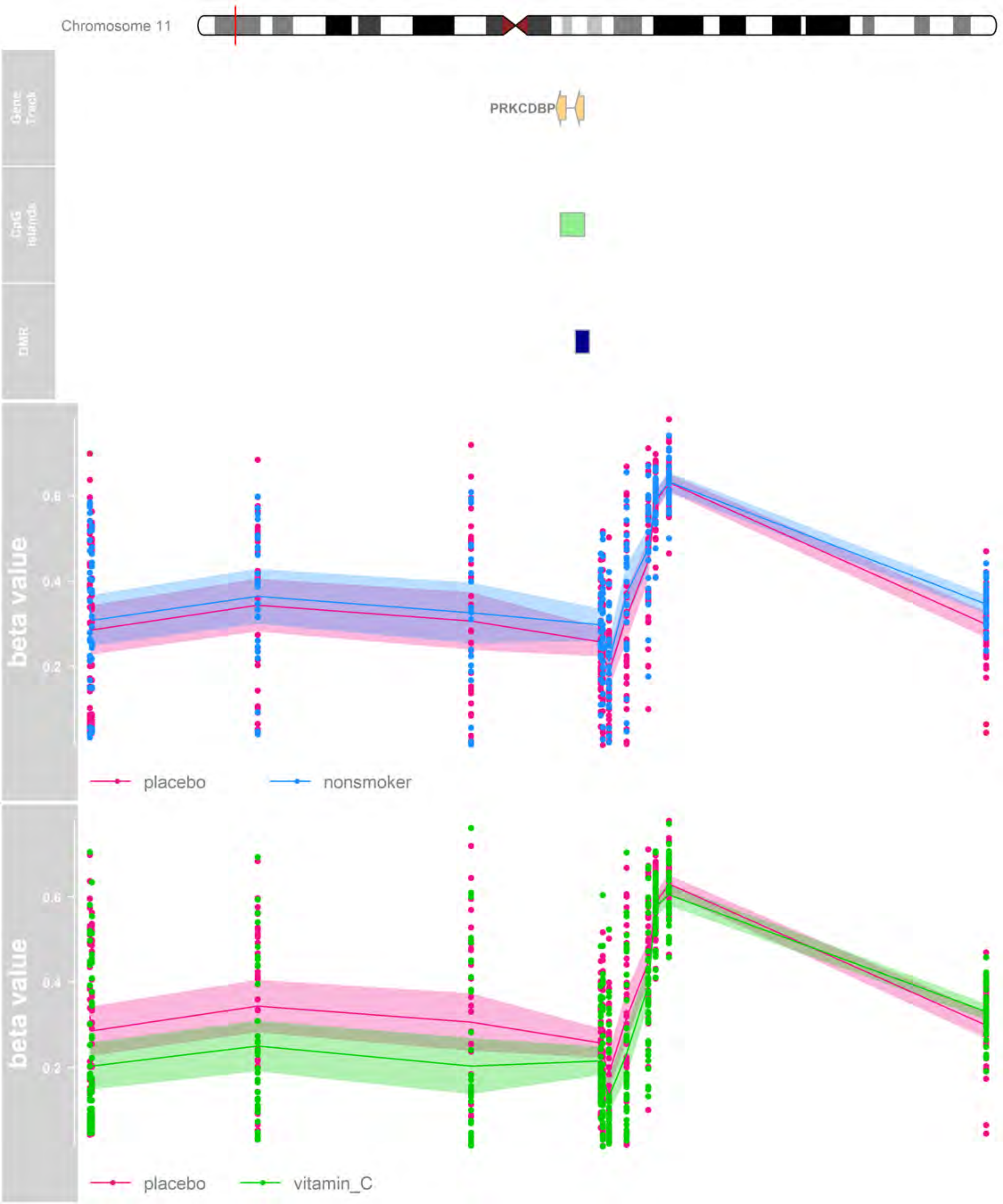

Chromosome 11
Gene Track
PRKCDBP
CpG islands
DMR
beta value
0.6
0.4
0.2
placebo
nonsmoker
beta value
0.6
0.4
0.2
placebo
vitamin_C

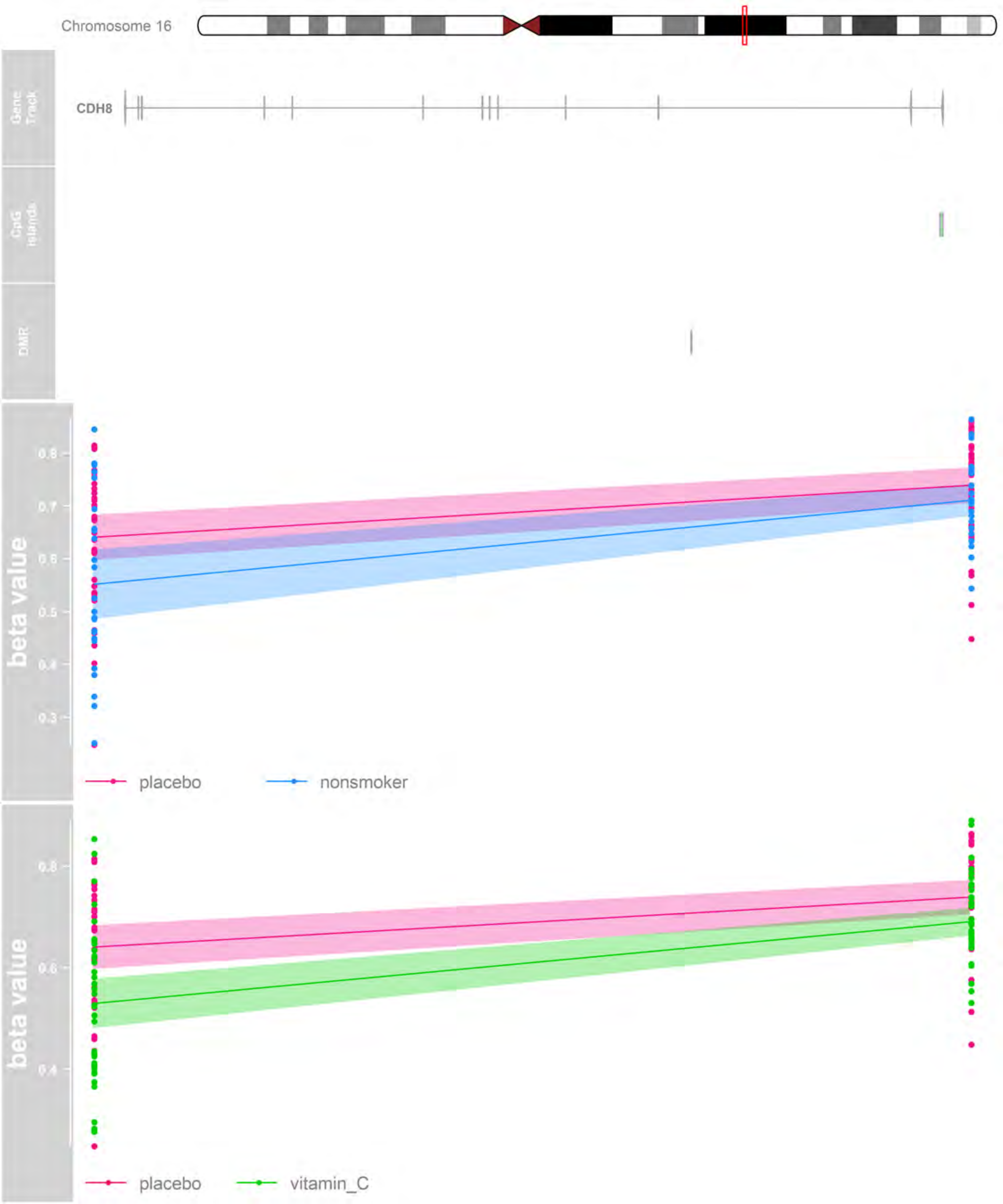

Chromosome 16
Gene Track
CDH8
CpG Islands
DMR
beta value
0.8
0.7
0.6
0.5
0.4
0.3
placebo
nonsmoker
beta value
0.8
0.6
0.4
placebo
vitamin_C

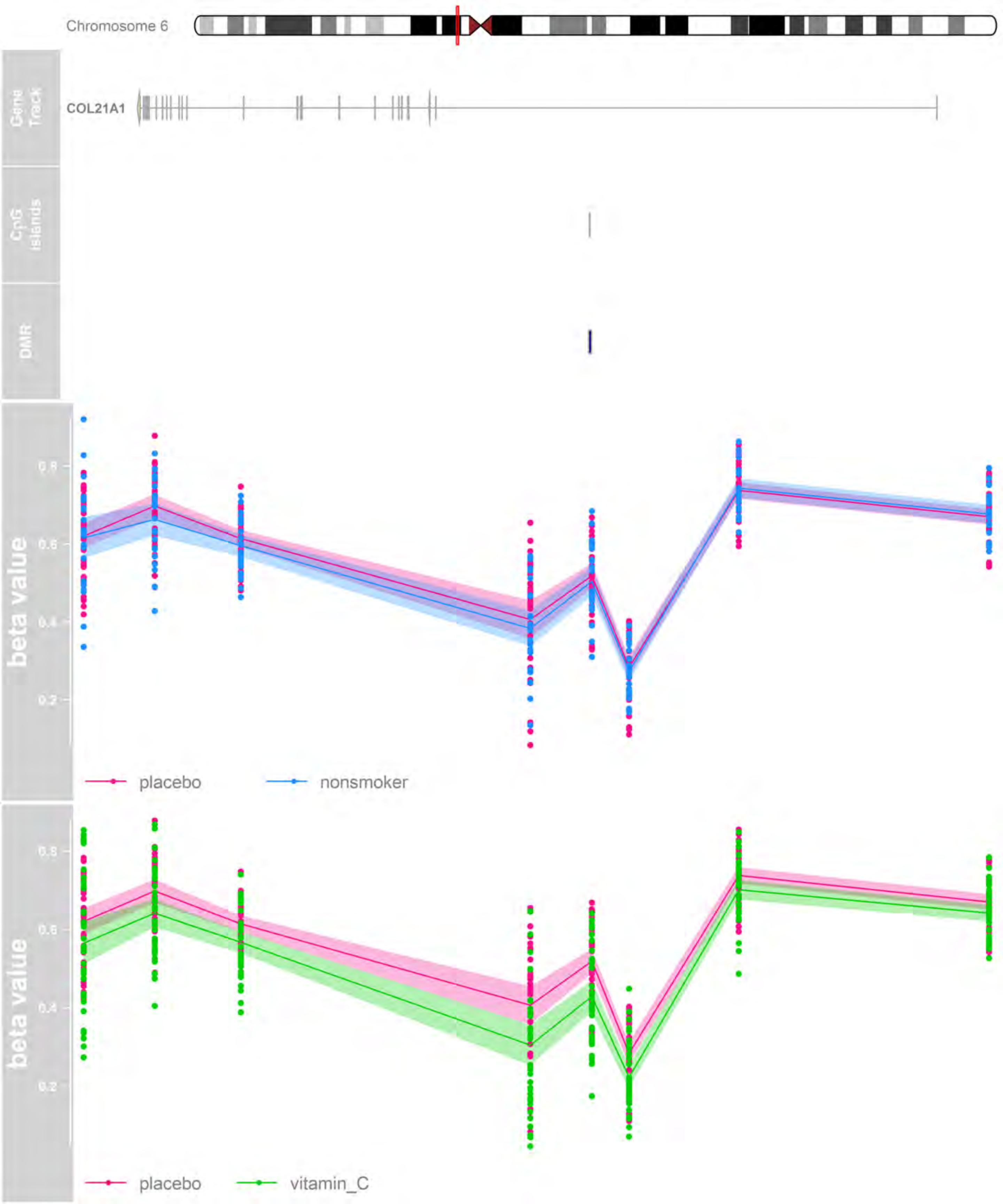
Chromosome 6
Gene Track
COL21A1
CpG islands
DMR
beta value
0.8
0.6
0.4
0.2
placebo
nonsmoker
beta value
0.8
0.6
0.4
0.2
placebo
vitamin_C

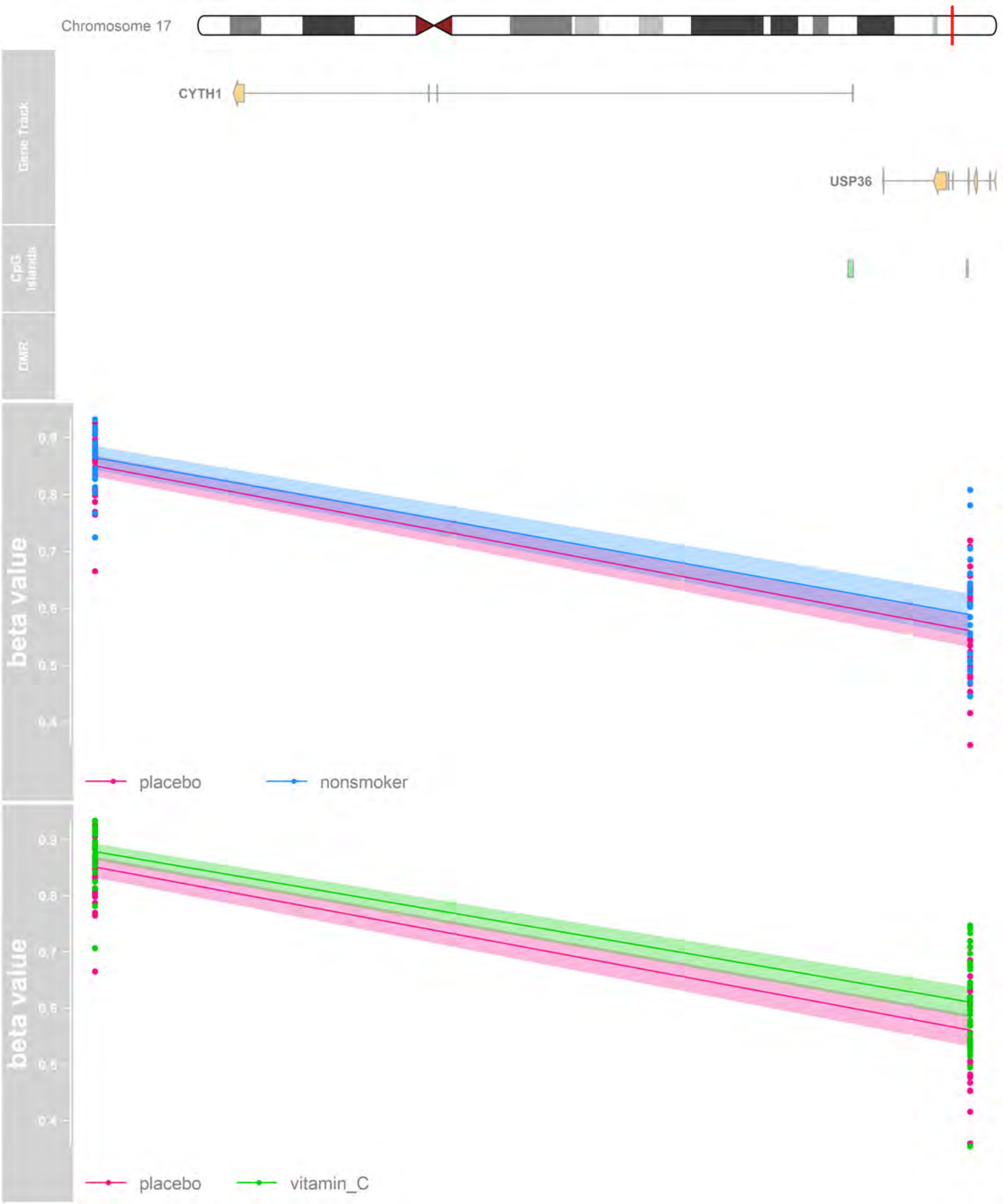

Chromosome 17
Gene Track
CYTH1
USP36
CpG Islands
DMR
beta value
0.9
0.8
0.7
0.6
0.5
0.4
placebo
nonsmoker
beta value
0.9
0.8
0.7
0.6
0.5
0.4
placebo
vitamin_C

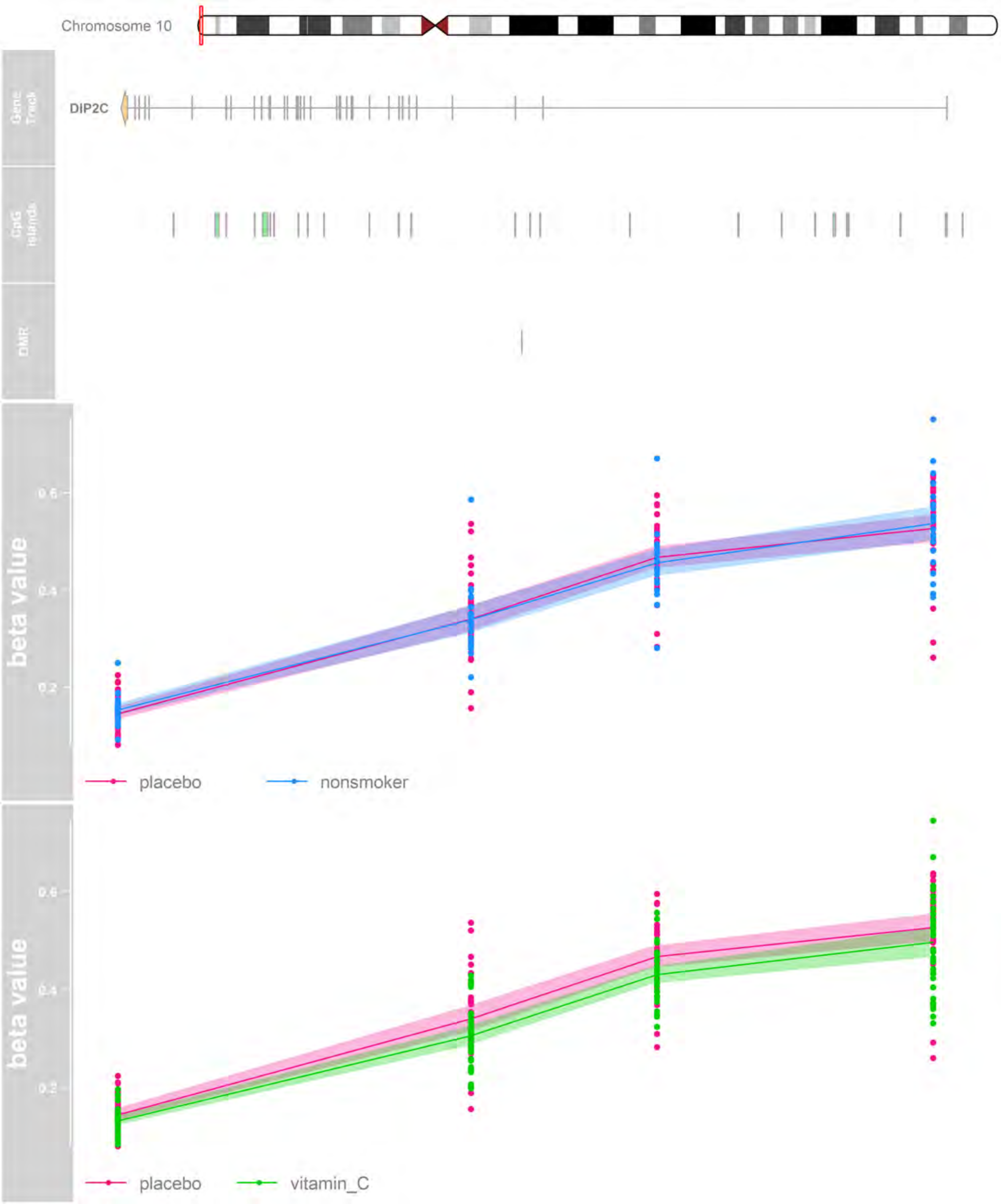
Chromosome 10
Gene Track
DIP2C
CpG islands
DMR
beta value
0.6
0.4
0.2
placebo
nonsmoker
beta value
0.6
0.4
0.2
placebo
vitamin_C

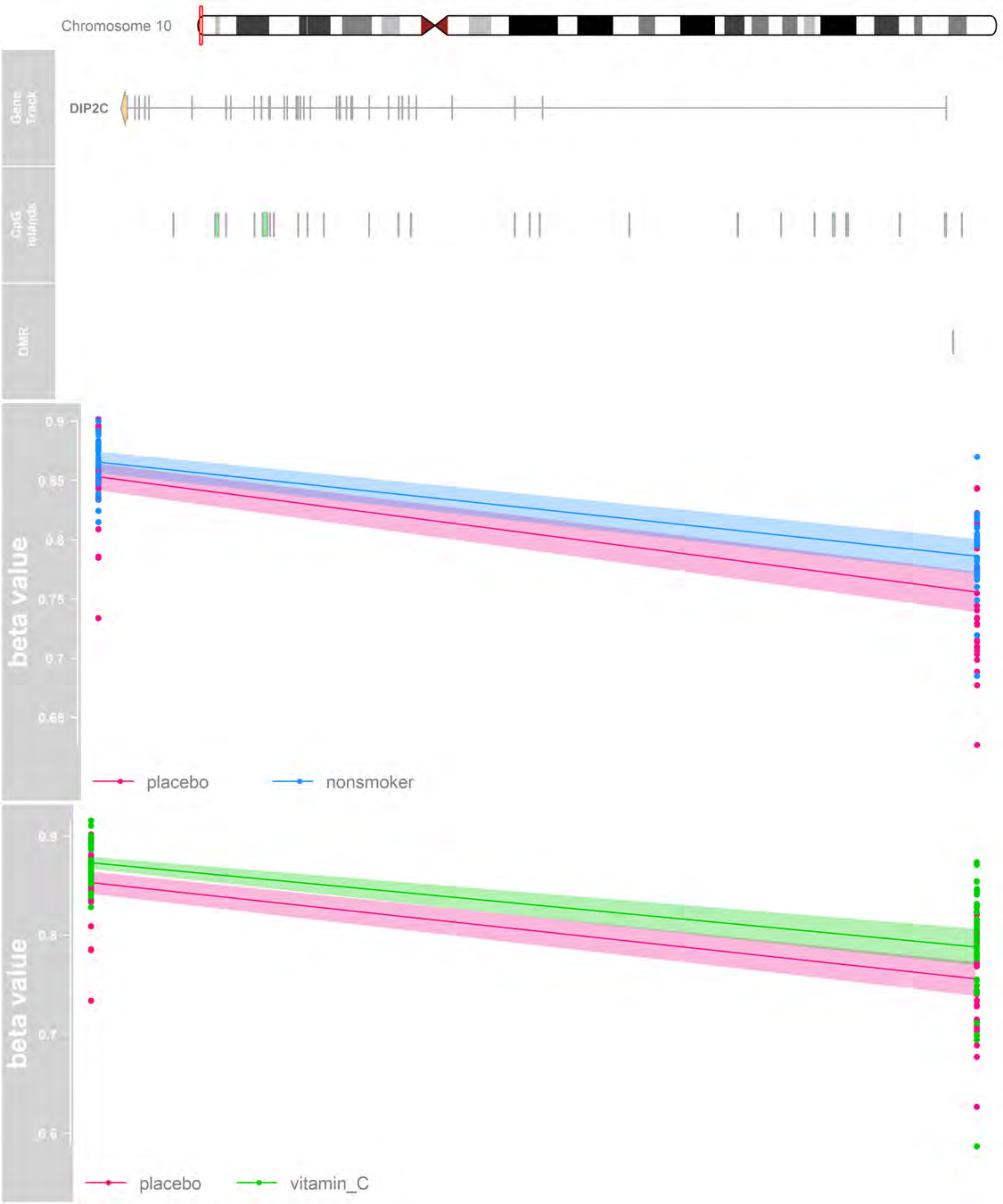
Chromosome 10
Gene Track
DIP2C
CpG islands
DMR
beta value
0.9
0.85
0.8
0.75
0.7
0.65
placebo
nonsmoker
beta value
0.9
0.8
0.7
0.6
placebo
vitamin_C

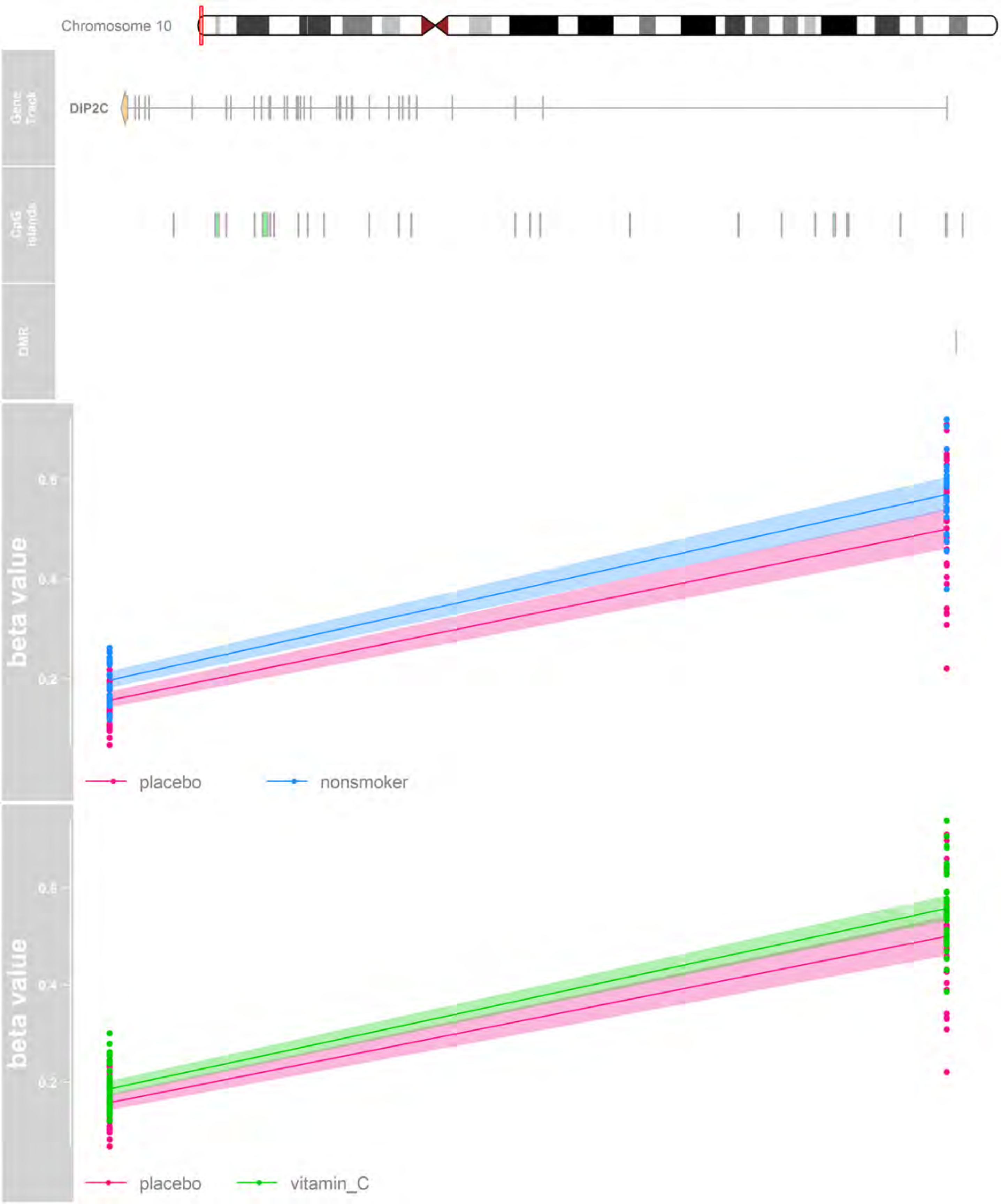

Chromosome 10
Gene Track
DIP2C
CpG islands
DMR
beta value
0.6
0.4
0.2
placebo
nonsmoker
beta value
0.6
0.4
0.2
placebo
vitamin_C

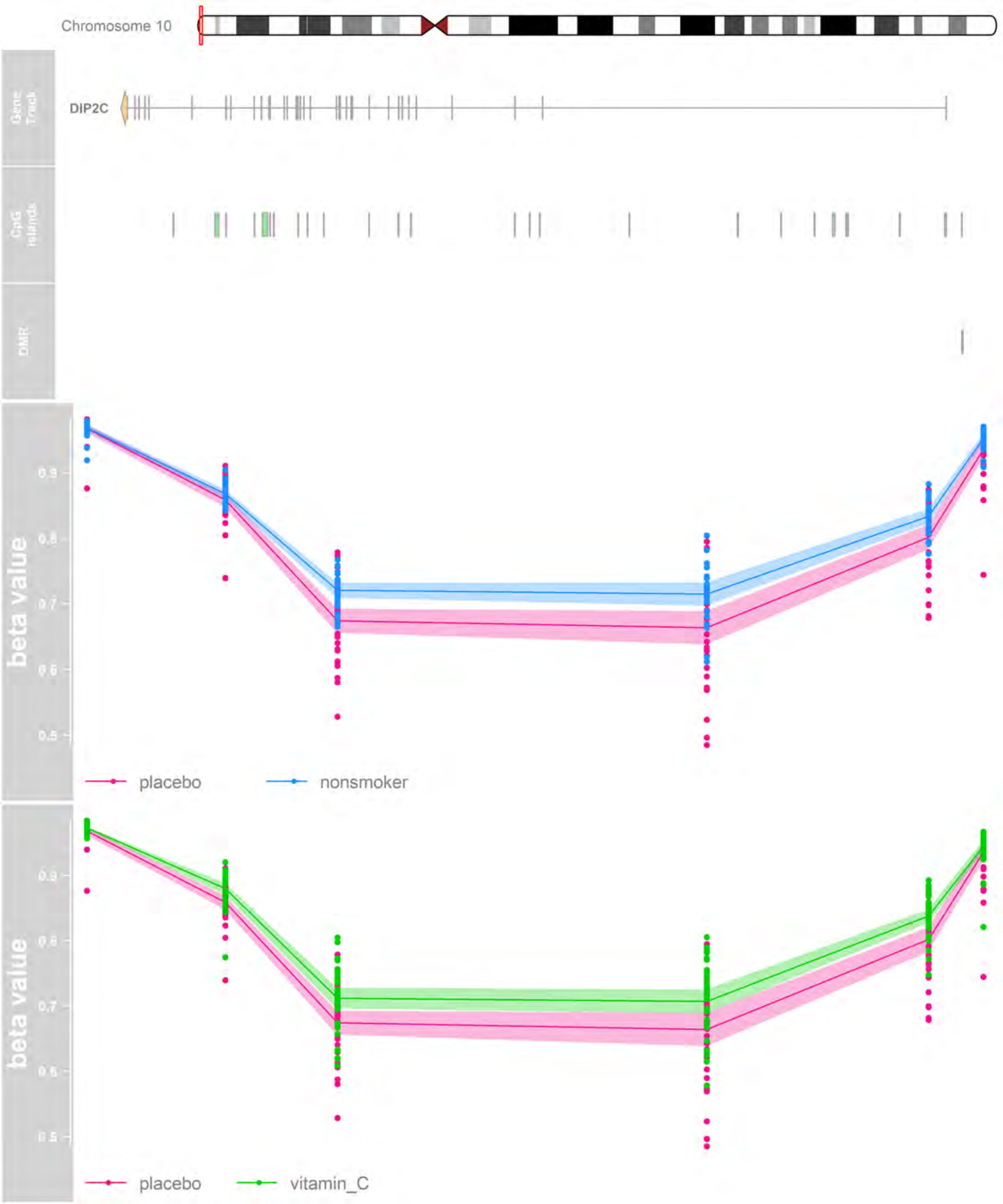

Chromosome 10
Gene Track
DIP2C
CpG islands
DMR
beta value
0.9
0.8
0.7
0.6
0.5
placebo
nonsmoker
beta value
0.9
0.8
0.7
0.6
0.5
placebo
vitamin_C

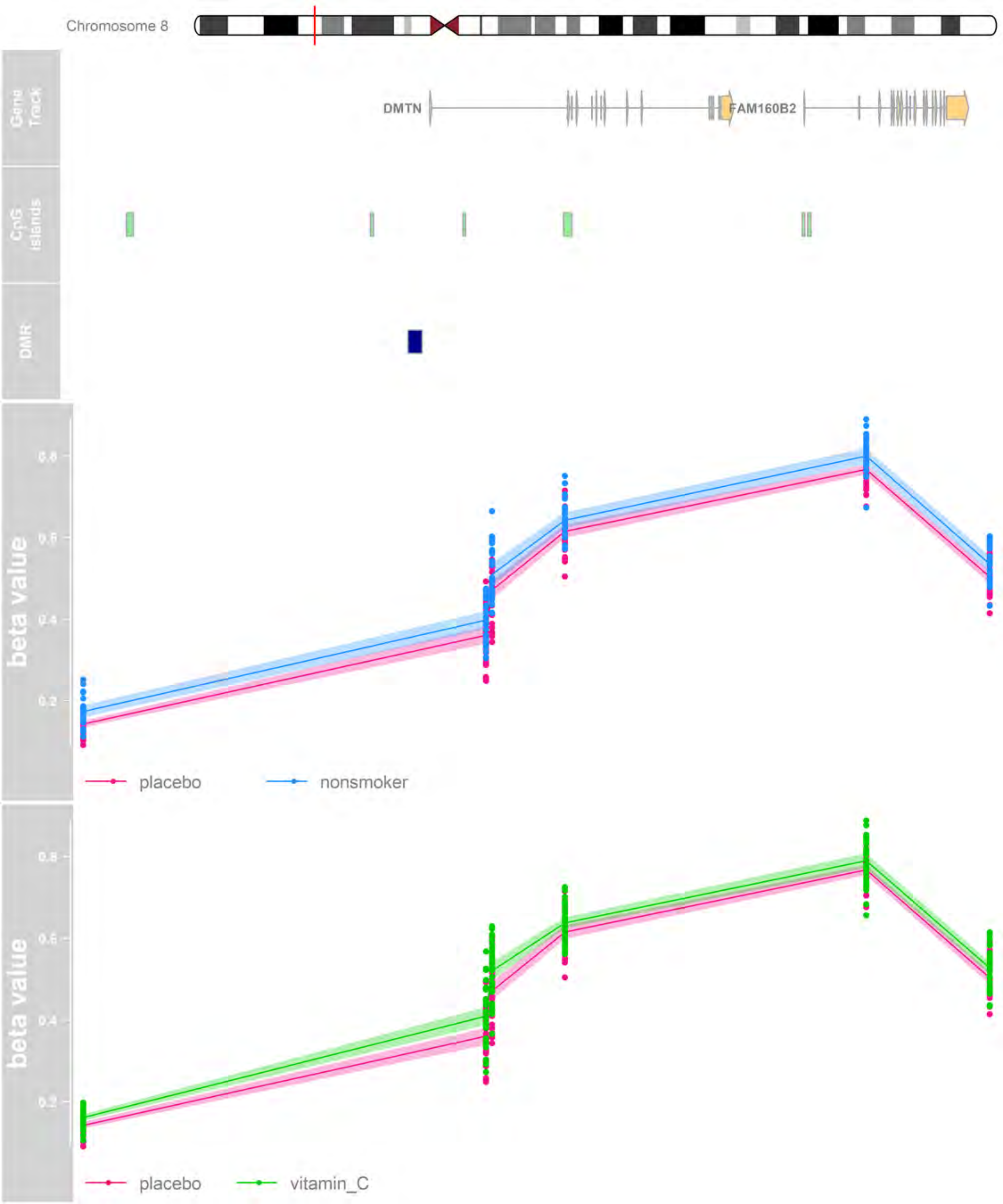
Chromosome 8
Gene Track
DMTN
FAM160B2
CpG islands
DMR
beta value
0.8
0.6
0.4
0.2
placebo
nonsmoker
beta value
0.8
0.6
0.4
0.2
placebo
vitamin_C

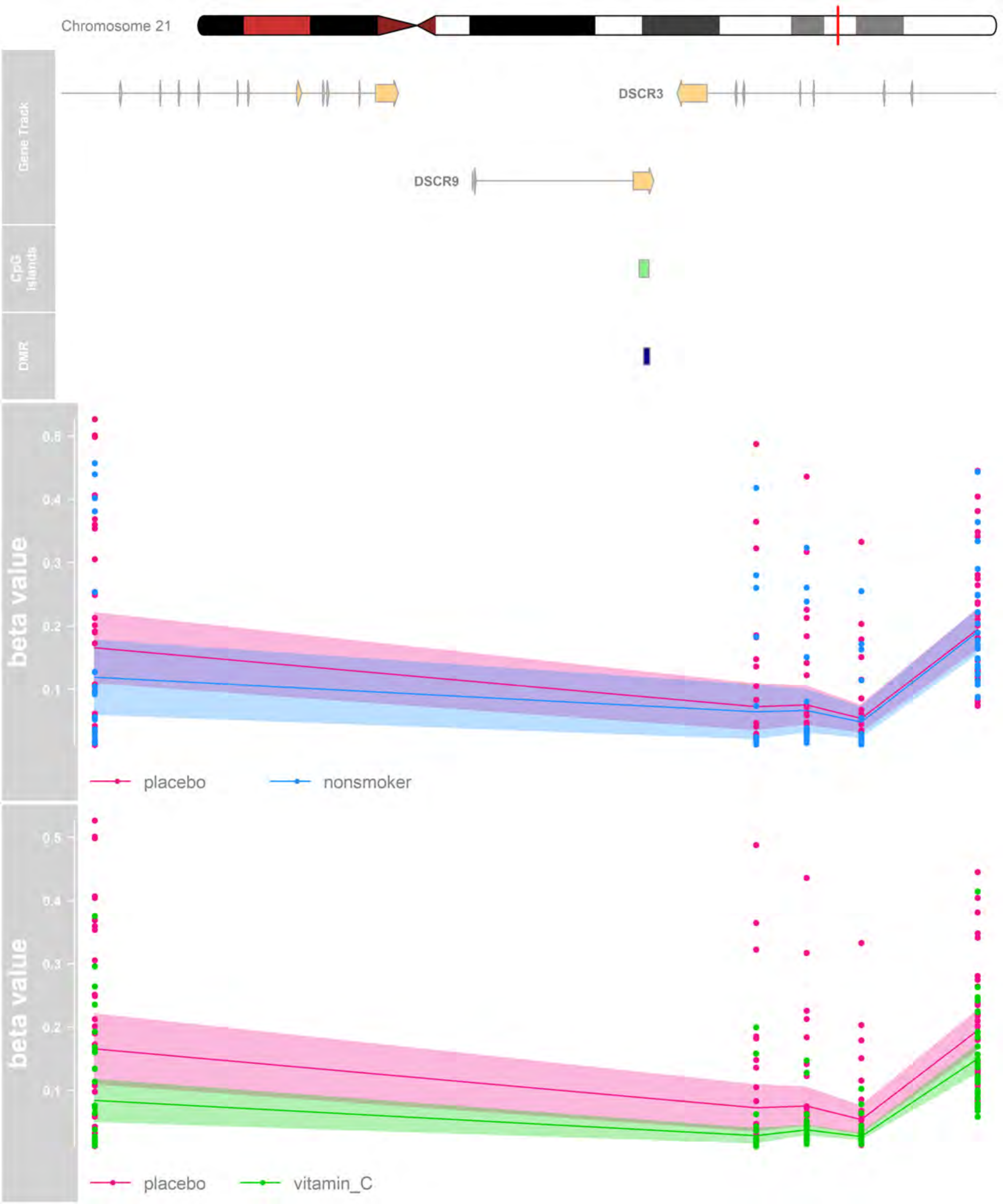
Chromosome 21
Gene Track
DSCR3
DSCR9
CpG Islands
DMR
beta value
0.5
0.4
0.3
0.2
0.1
placebo
nonsmoker
beta value
0.5
0.4
0.3
0.2
0.1
placebo
vitamin_C

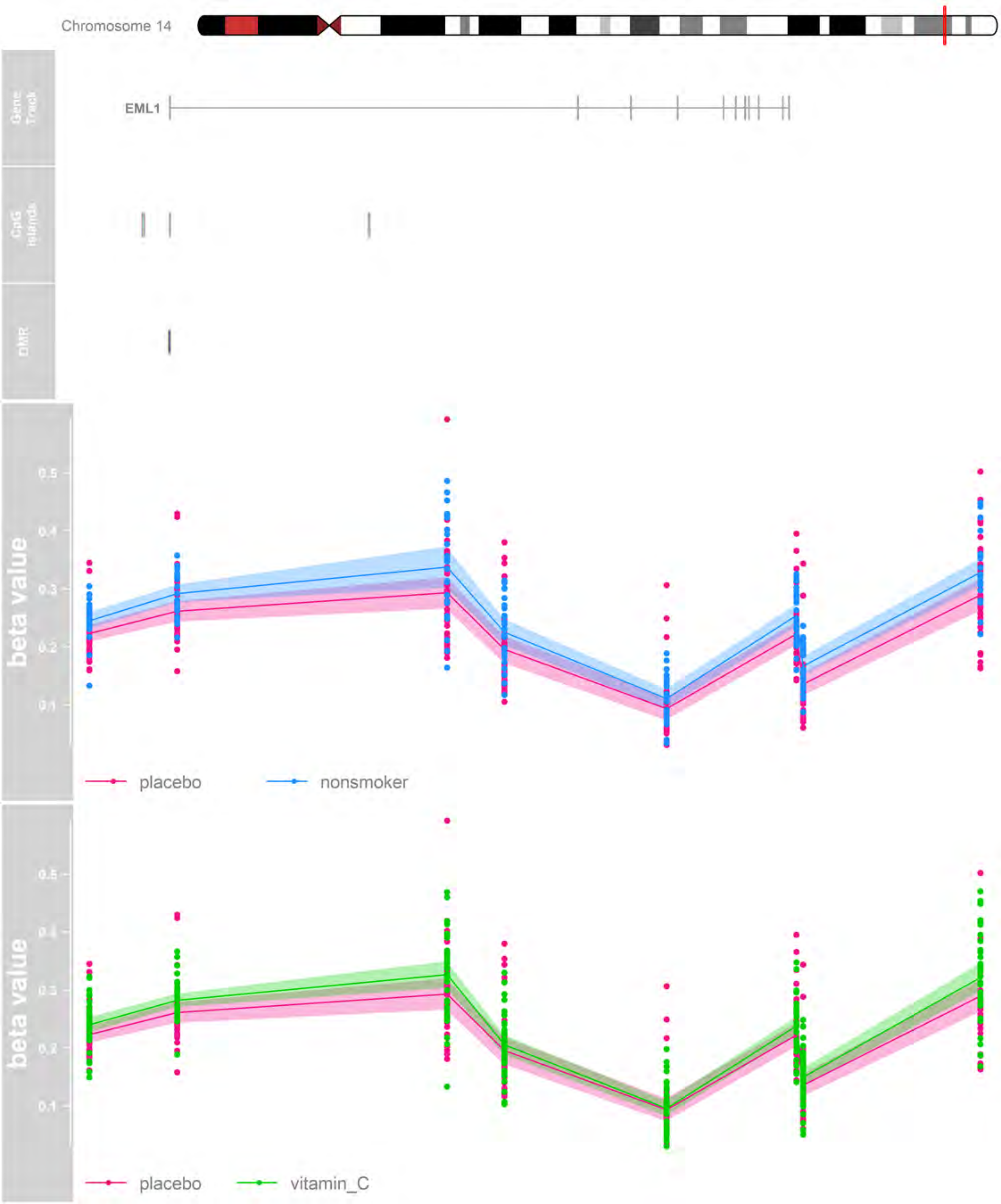

Chromosome 14
Gene Track
EML1
CpG Islands
DMR
beta value
0.5
0.4
0.3
0.2
0.1
placebo
nonsmoker
beta value
0.5
0.4
0.3
0.2
0.1
placebo
vitamin_C

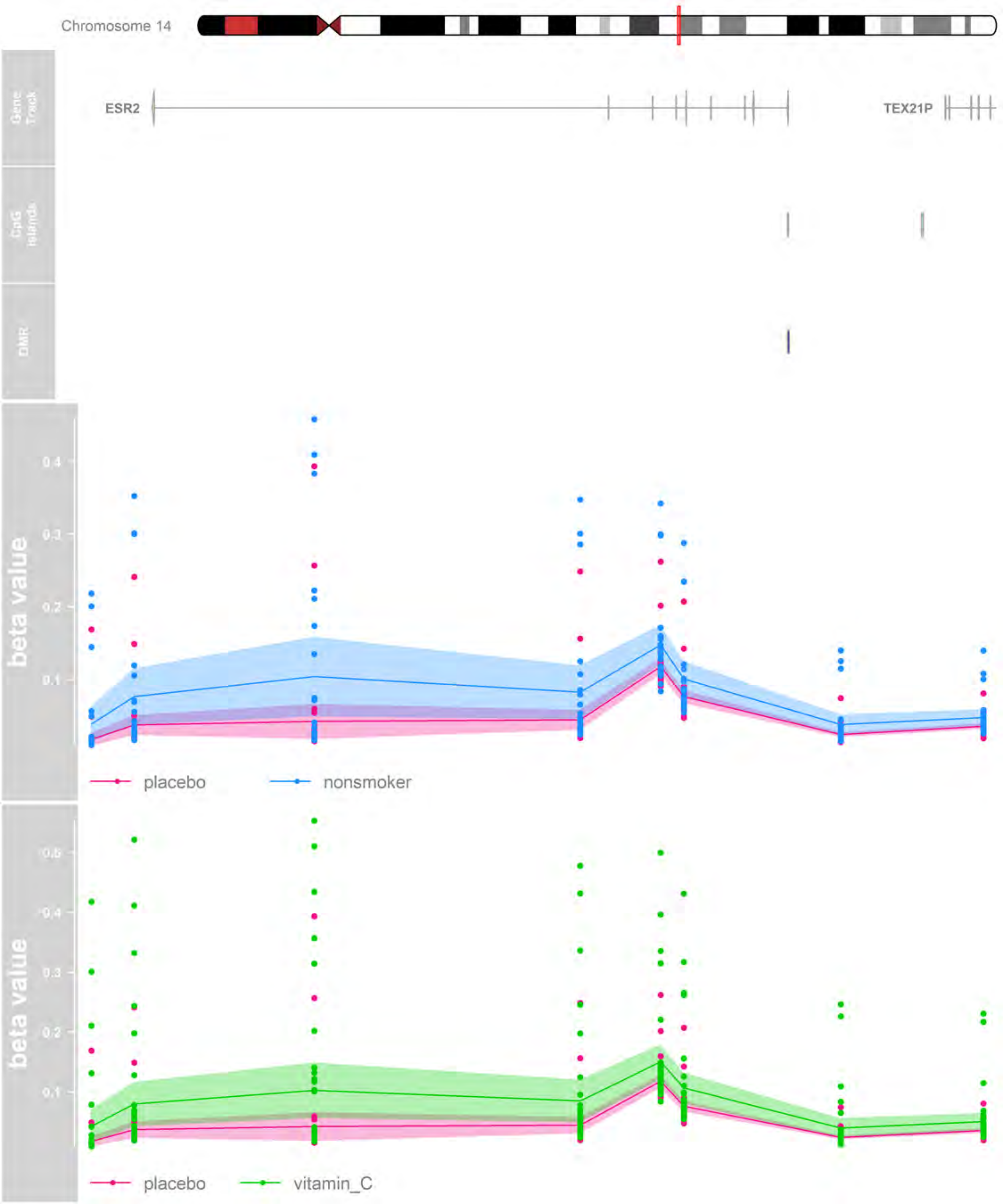

Chromosome 14
Gene Track
ESR2
TEX21P
CpG islands
DMR
beta value
0.4
0.3
0.2
0.1
placebo
nonsmoker
beta value
0.5
0.4
0.3
0.2
0.1
placebo
vitamin_C

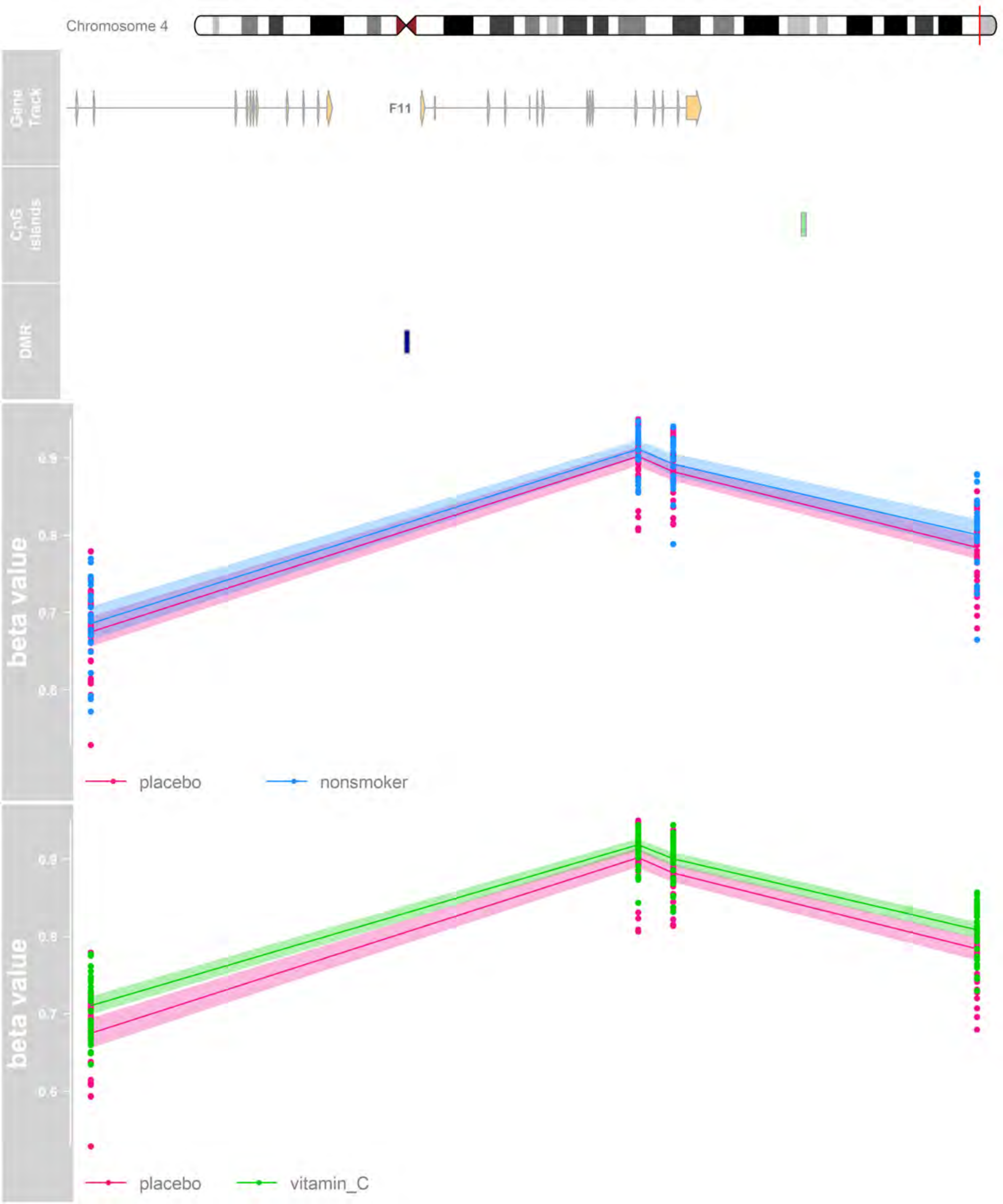

Chromosome 4
Gene Track
F11
CpG Islands
DMR
beta value
0.9
0.8
0.7
0.6
placebo
nonsmoker
beta value
0.9
0.8
0.7
0.6
placebo
vitamin_C

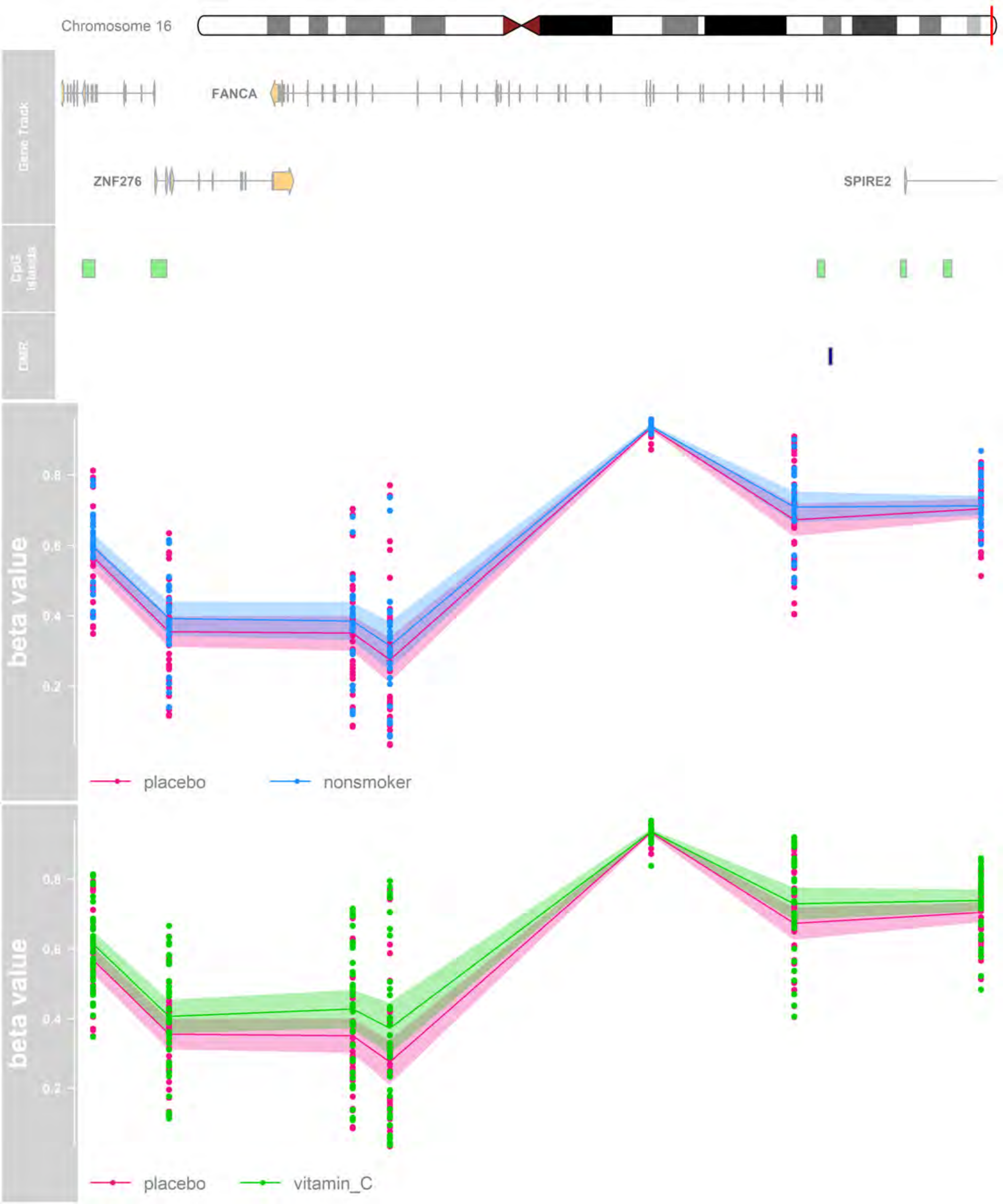

Chromosome 16
Gene Track
FANCA
ZNF276
SPIRE2
CpG Islands
DMR
beta value
0.8
0.6
0.4
0.2
placebo
nonsmoker
beta value
0.8
0.6
0.4
0.2
placebo
vitamin_C

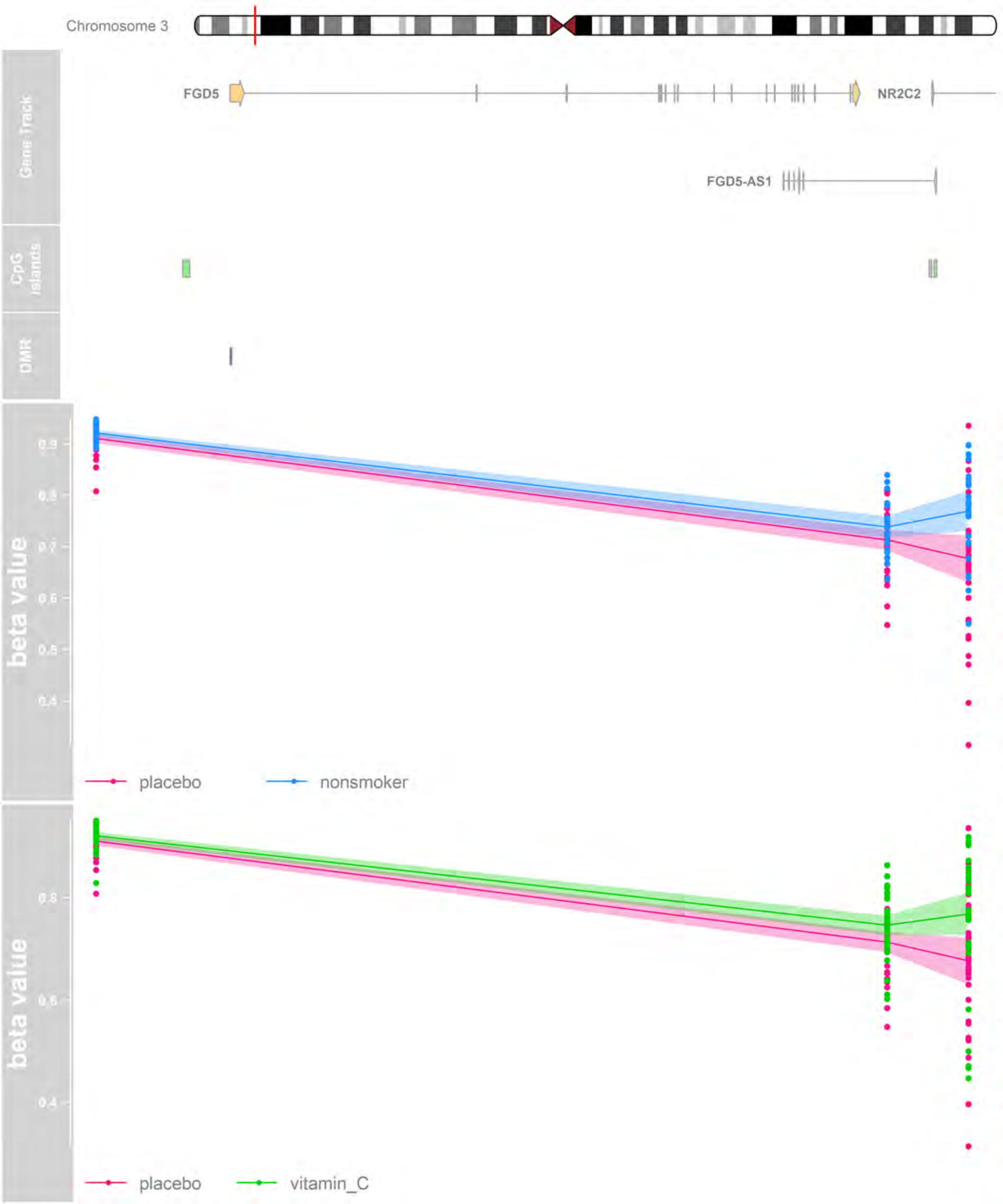

Chromosome 3
Gene Track
FGD5
NR2C2
FGD5-AS1
CpG islands
DMR
beta value
0.9
0.8
0.7
0.6
0.5
0.4
placebo
nonsmoker
beta value
0.8
0.6
0.4
placebo
vitamin_C

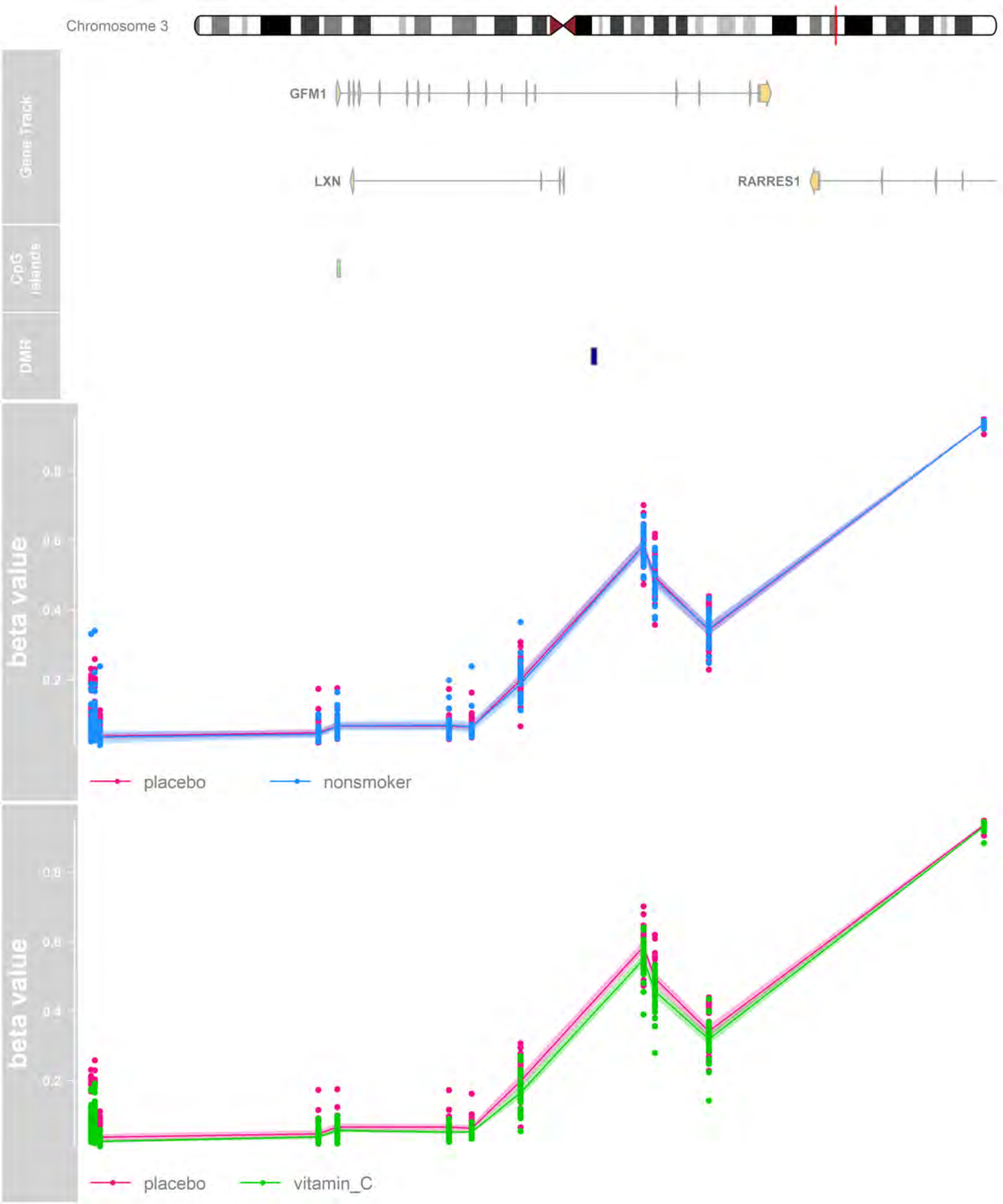

Chromosome 3
Gene Track
GFM1
LXN
RARRES1
CpG islands
DMR
beta value
0.8
0.6
0.4
0.2
placebo
nonsmoker
beta value
0.8
0.6
0.4
0.2
placebo
vitamin_C

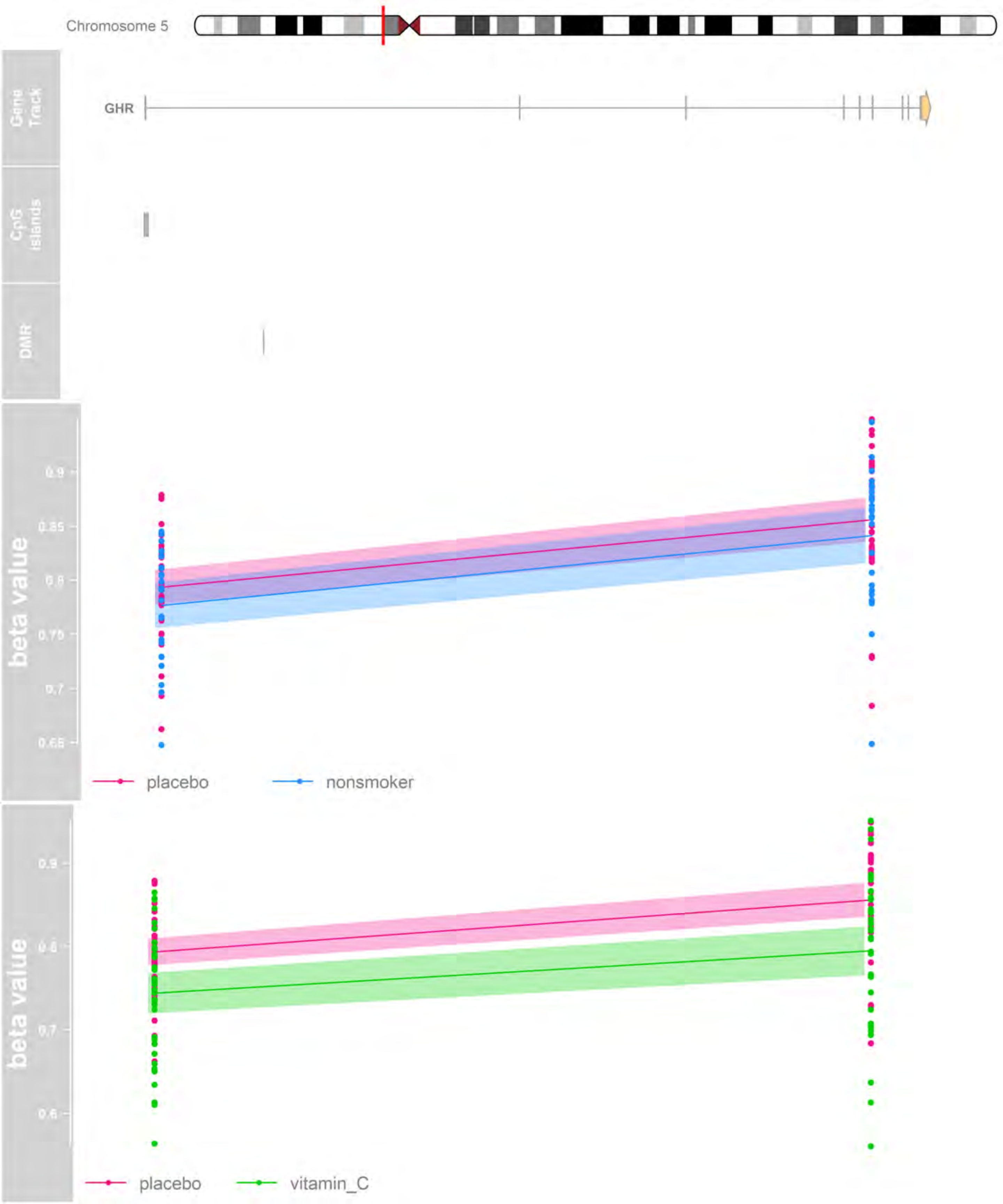
Chromosome 5
Gene Track
GHR
CpG Islands
DMR
beta value
0.9
0.85
0.8
0.75
0.7
0.65
placebo
nonsmoker
beta value
0.9
0.8
0.7
0.6
placebo
vitamin_C

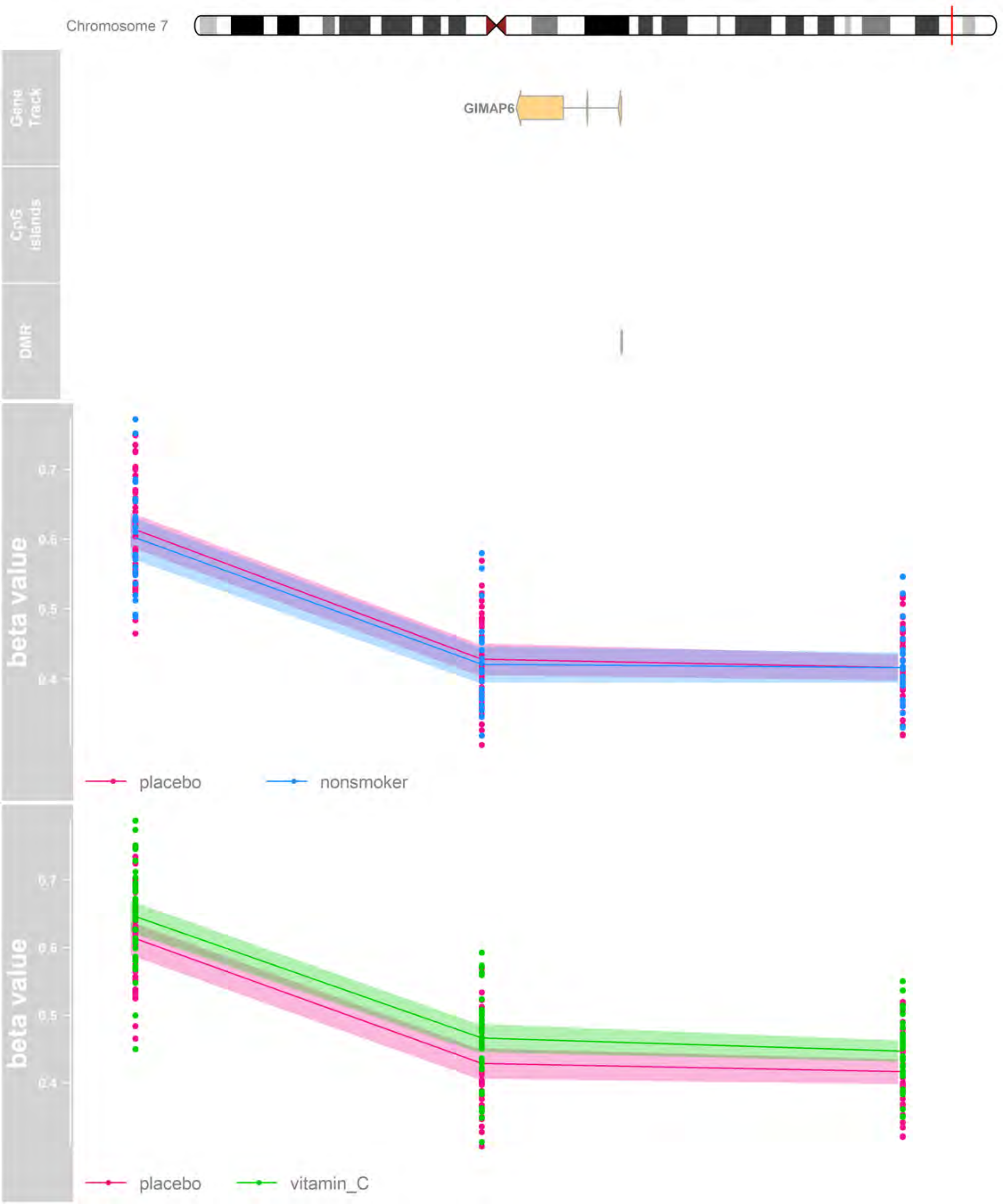

Chromosome 7
Gene Track
GIMAP6
CpG Islands
DMR
beta value
0.7
0.6
0.5
0.4
placebo
nonsmoker
beta value
0.7
0.6
0.5
0.4
placebo
vitamin_C

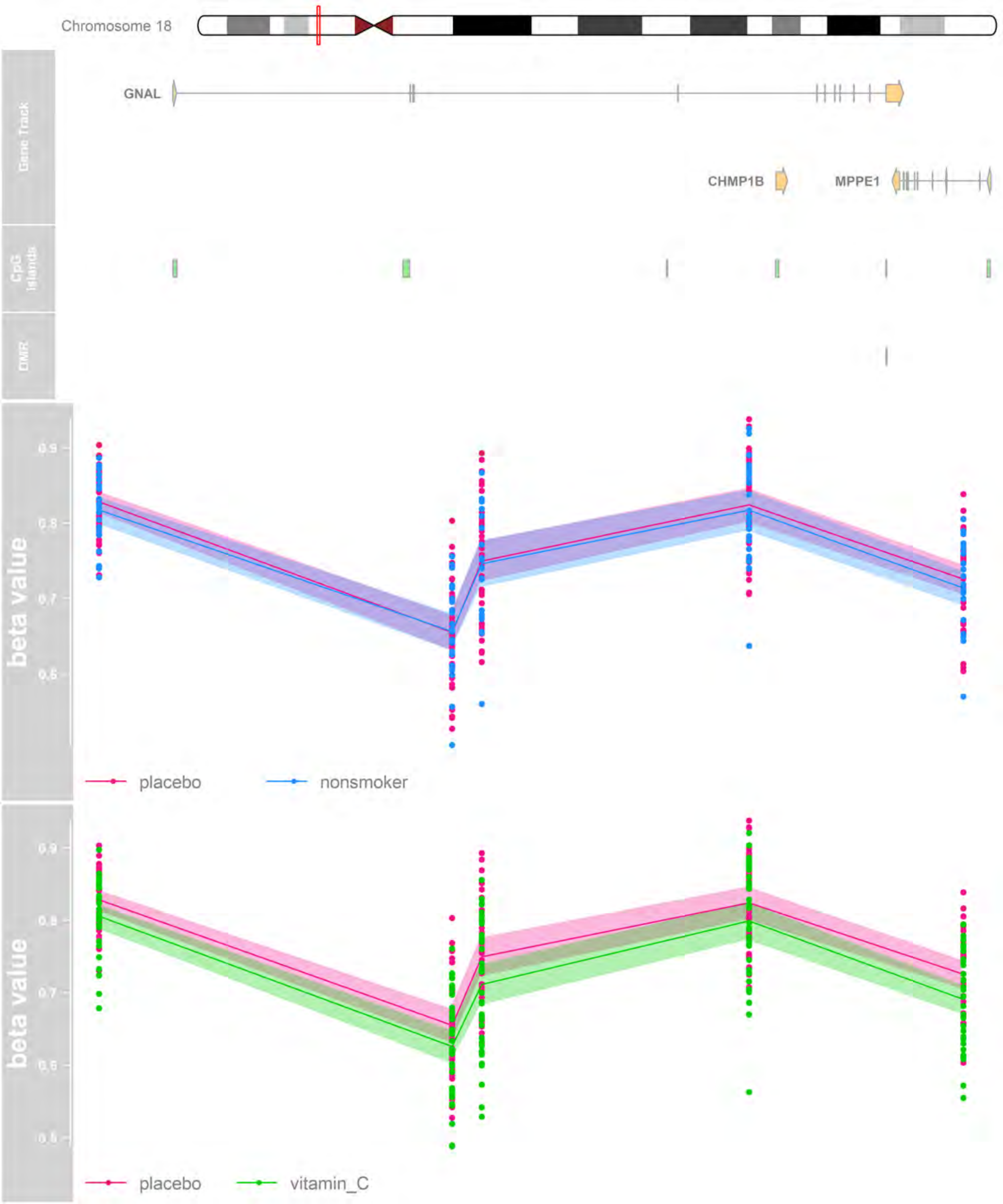

Chromosome 18
Gene Track
GNAL
CHMP1B
MPPE1
CpG Islands
DMR
beta value
0.9
0.8
0.7
0.6
placebo
nonsmoker
beta value
0.9
0.8
0.7
0.6
0.5
placebo
vitamin_C

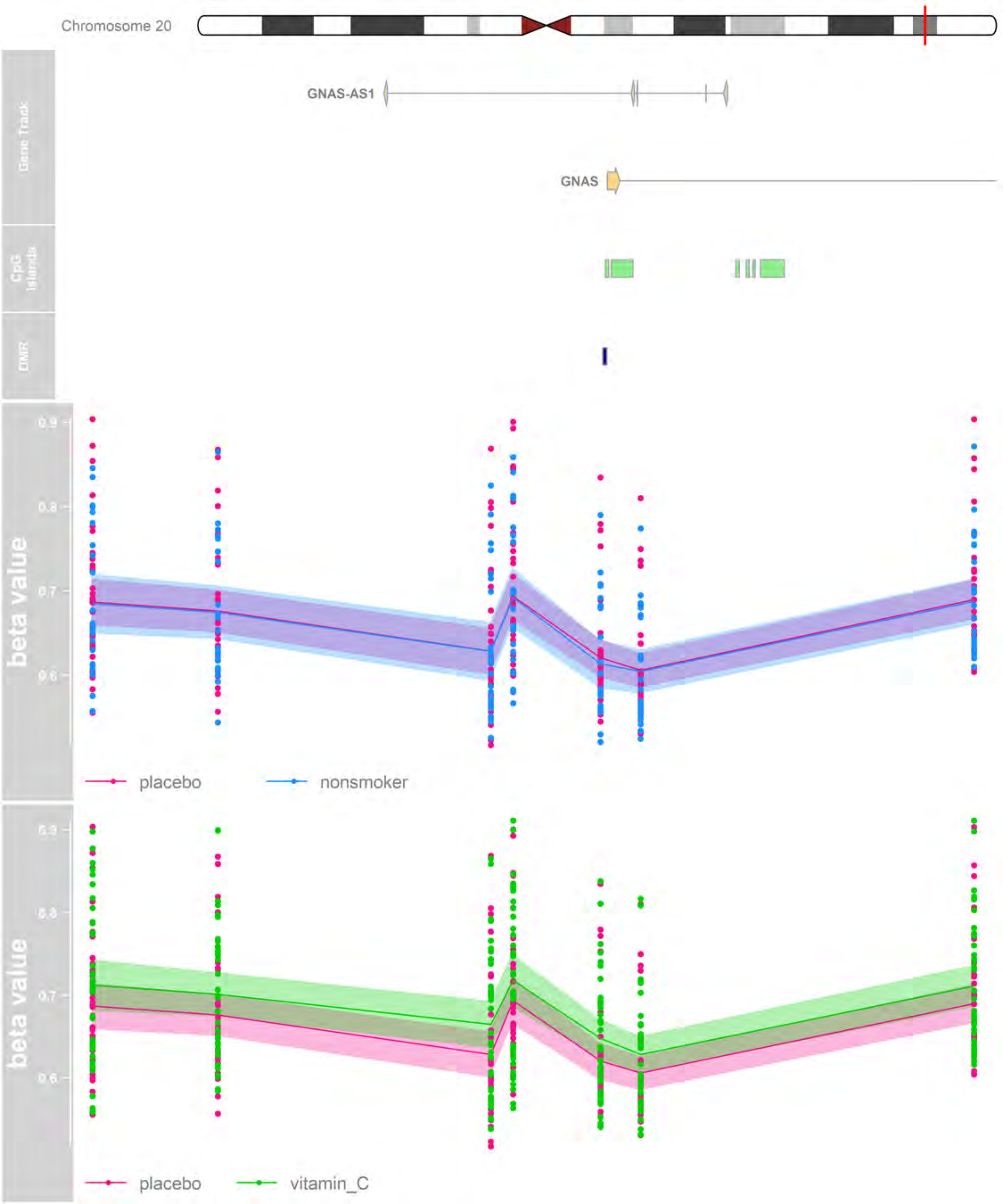

Chromosome 20
Gene Track
GNAS-AS1
GNAS
CpG Islands
DMR
beta value
0.9
0.8
0.7
0.6
placebo
nonsmoker
beta value
0.9
0.8
0.7
0.6
placebo
vitamin_C

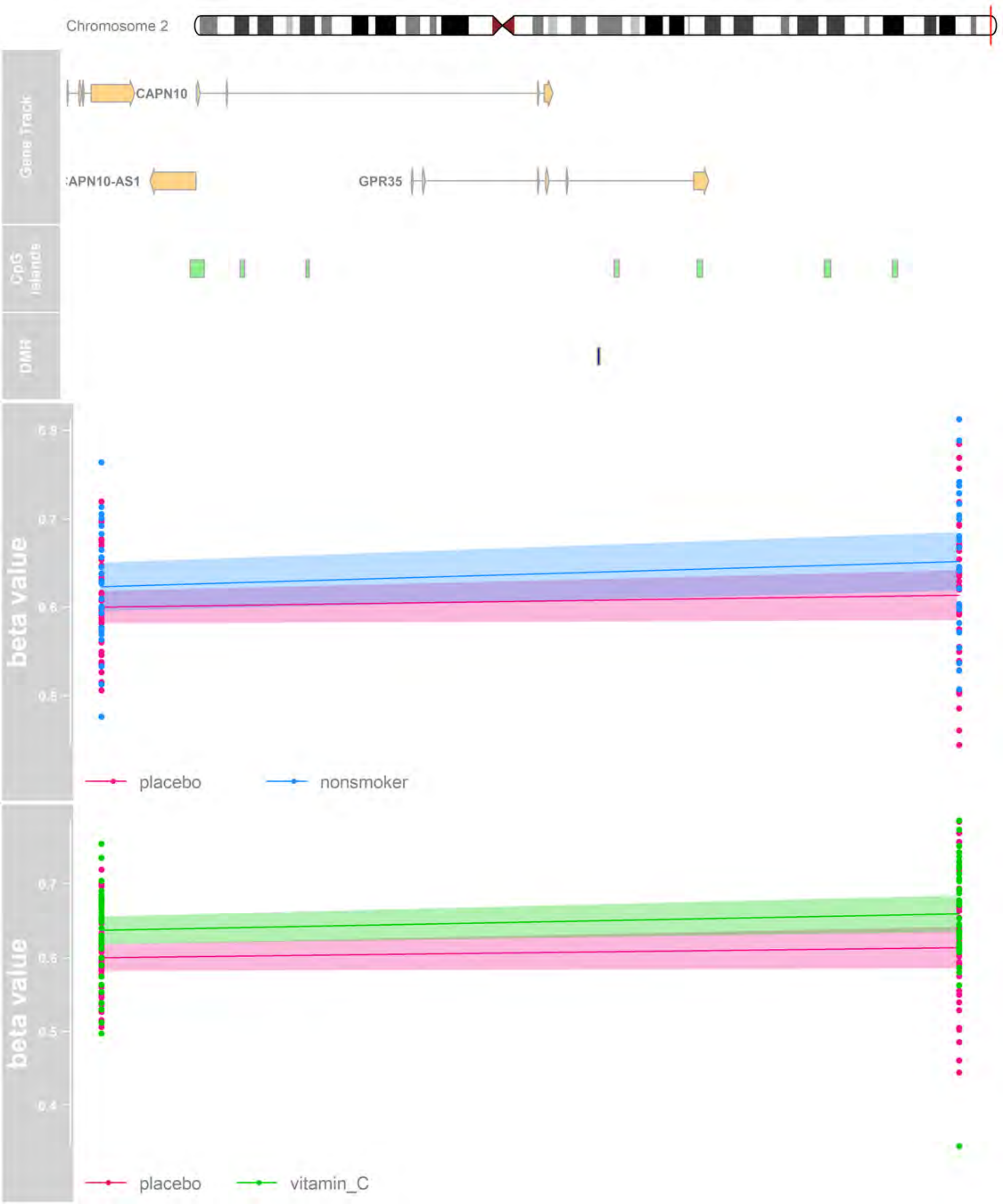

Chromosome 2
Gene Track
CAPN10
:APN10-AS1
GPR35
CpG islands
DMR
beta value
0.8
0.7
0.6
0.5
placebo
nonsmoker
beta value
0.7
0.6
0.5
0.4
placebo
vitamin_C

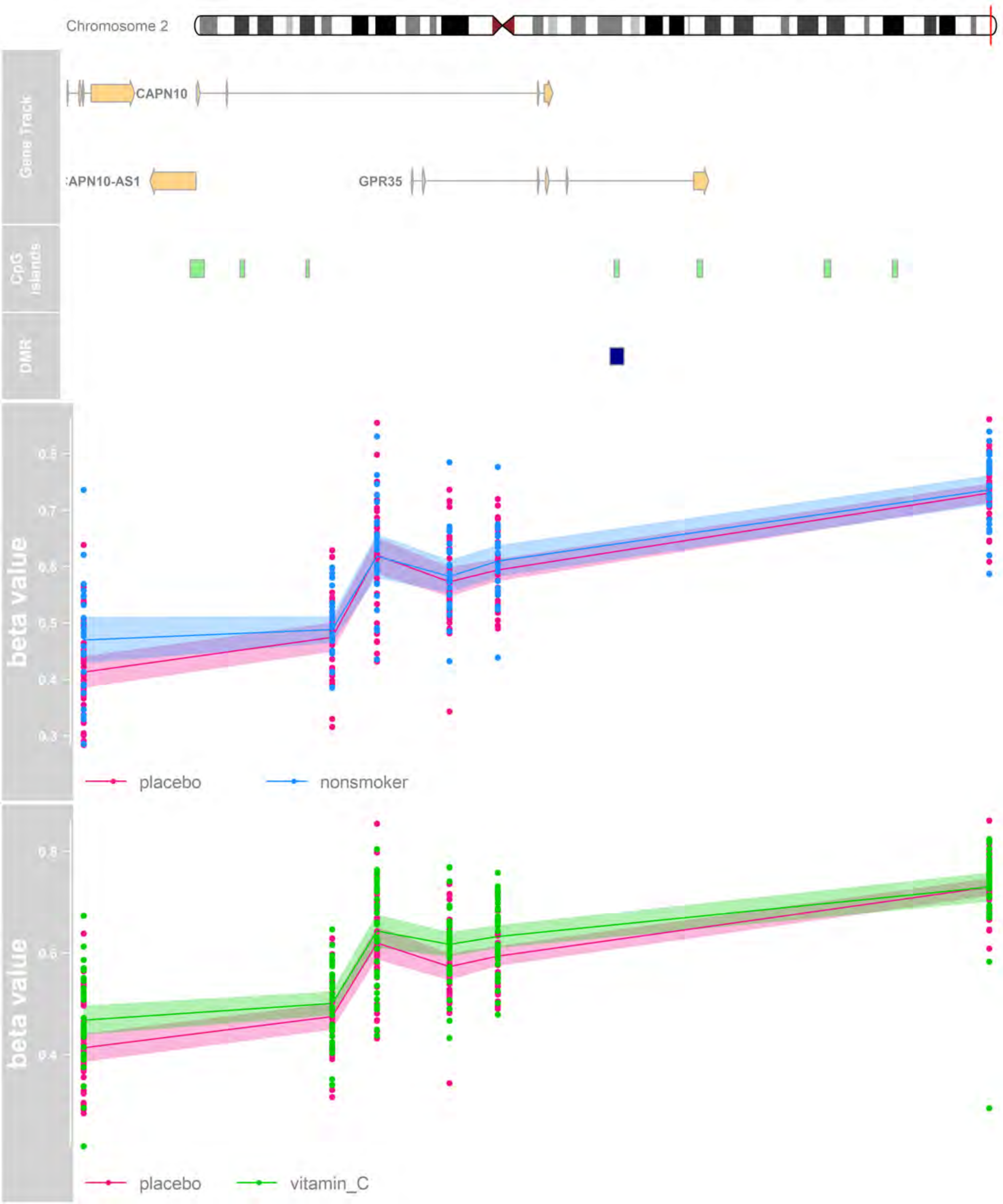

Chromosome 2
Gene Track
CAPN10
CAPN10-AS1
GPR35
CpG Islands
DMR
beta value
placebo
nonsmoker
beta value
placebo
vitamin_C

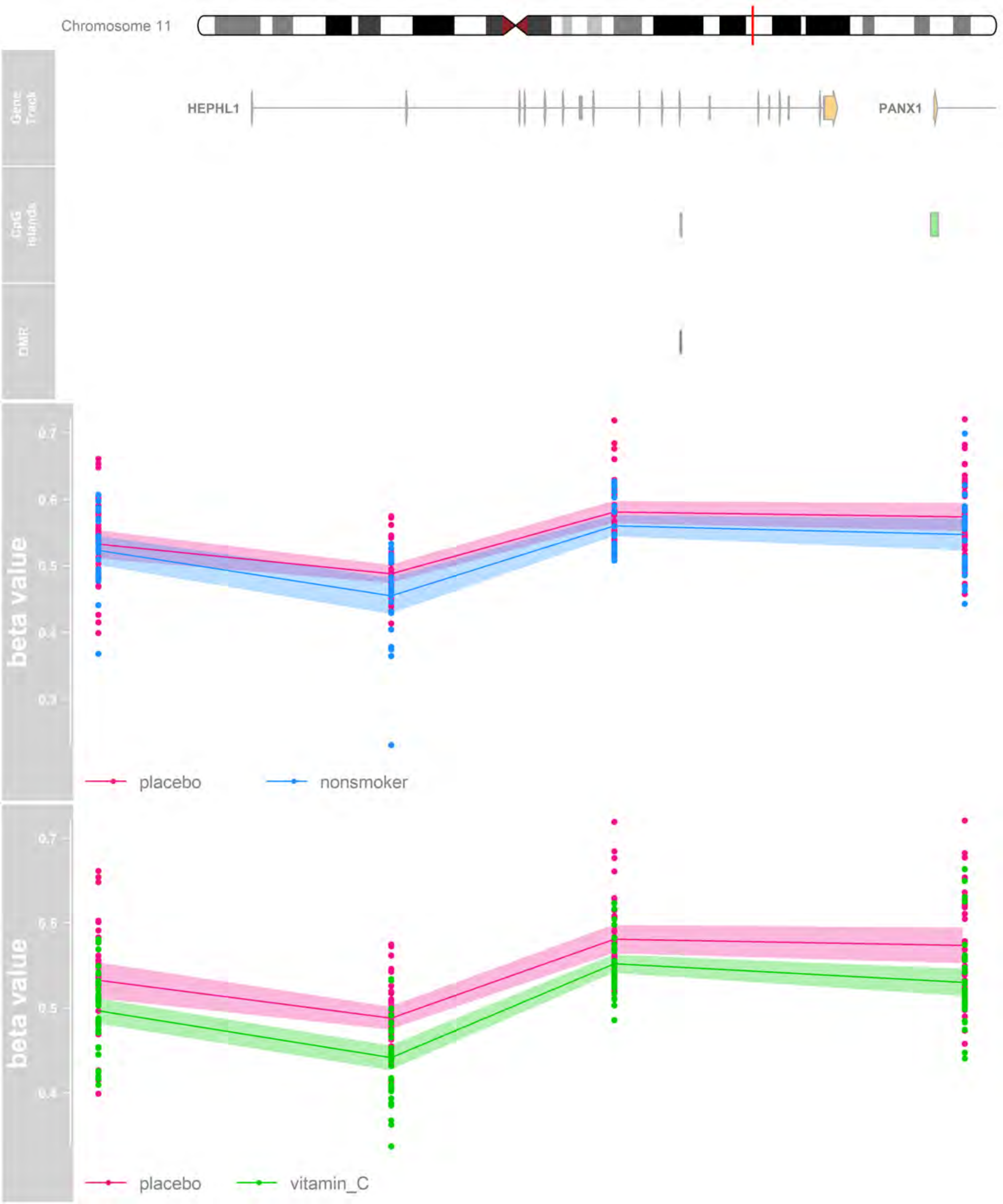

Chromosome 11
Gene Track
HEPHL1
PANX1
CpG islands
DMR
beta value
0.7
0.6
0.5
0.4
0.3
placebo
nonsmoker
beta value
0.7
0.6
0.5
0.4
placebo
vitamin_C

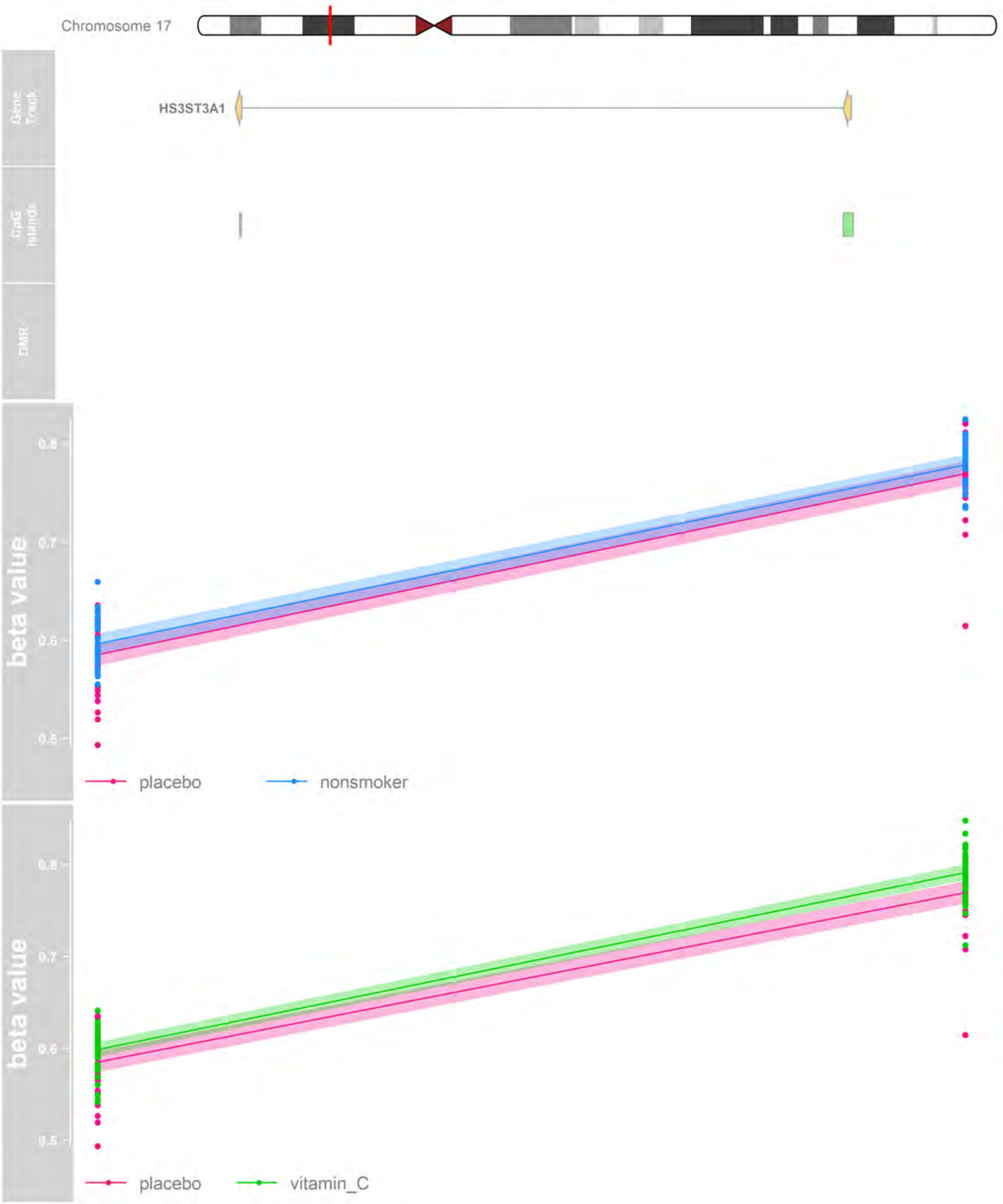

Chromosome 17
Gene Track
HS3ST3A1
CpG islands
DMR
beta value
0.8
0.7
0.6
0.5
placebo
nonsmoker
beta value
0.8
0.7
0.6
0.5
placebo
vitamin_C

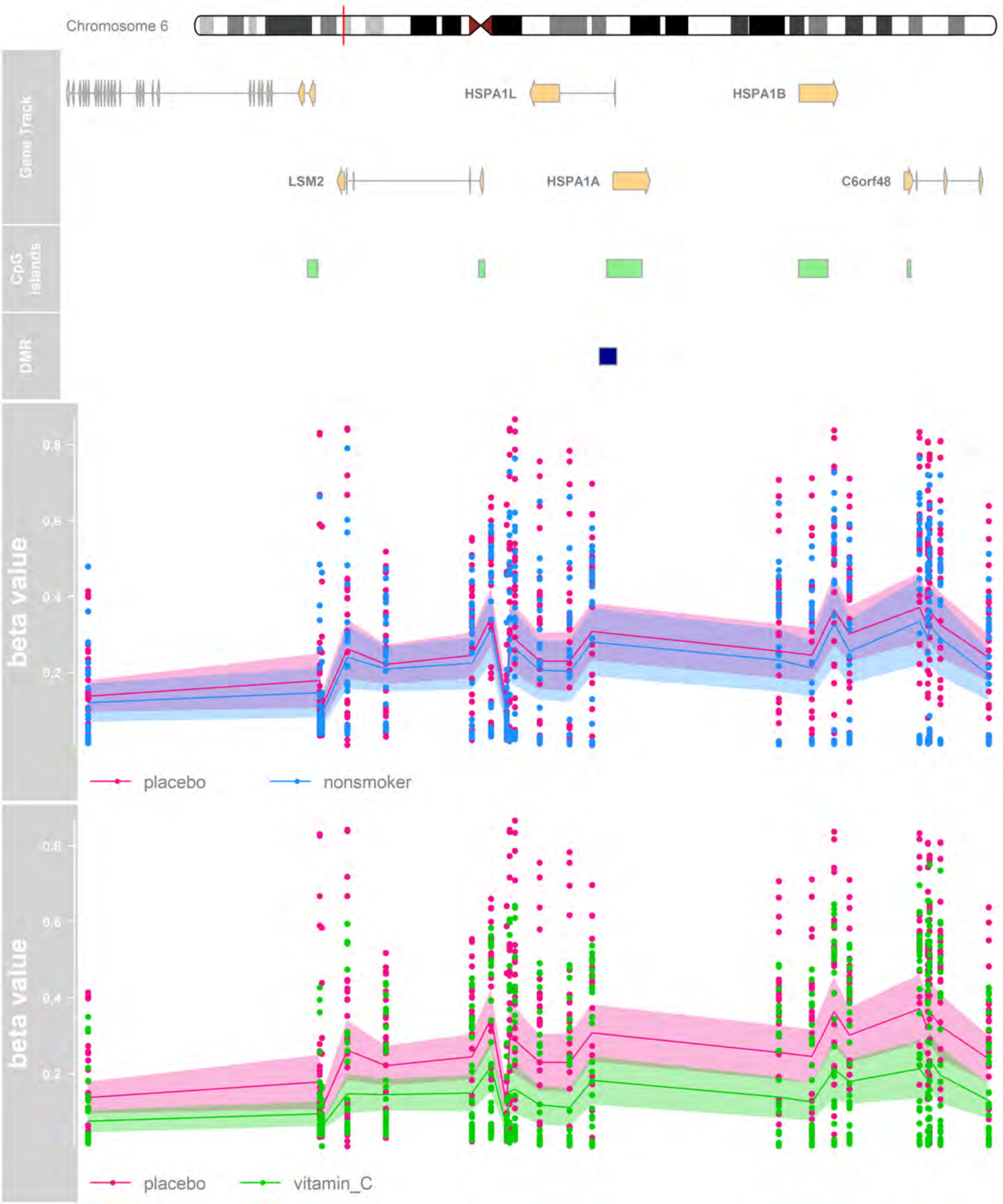

Chromosome 6
Gene Track
HSPA1L
HSPA1B
LSM2
HSPA1A
C6orf48
CpG islands
DMR
beta value
0.8
0.6
0.4
0.2
placebo
nonsmoker
beta value
0.8
0.6
0.4
0.2
placebo
vitamin_C

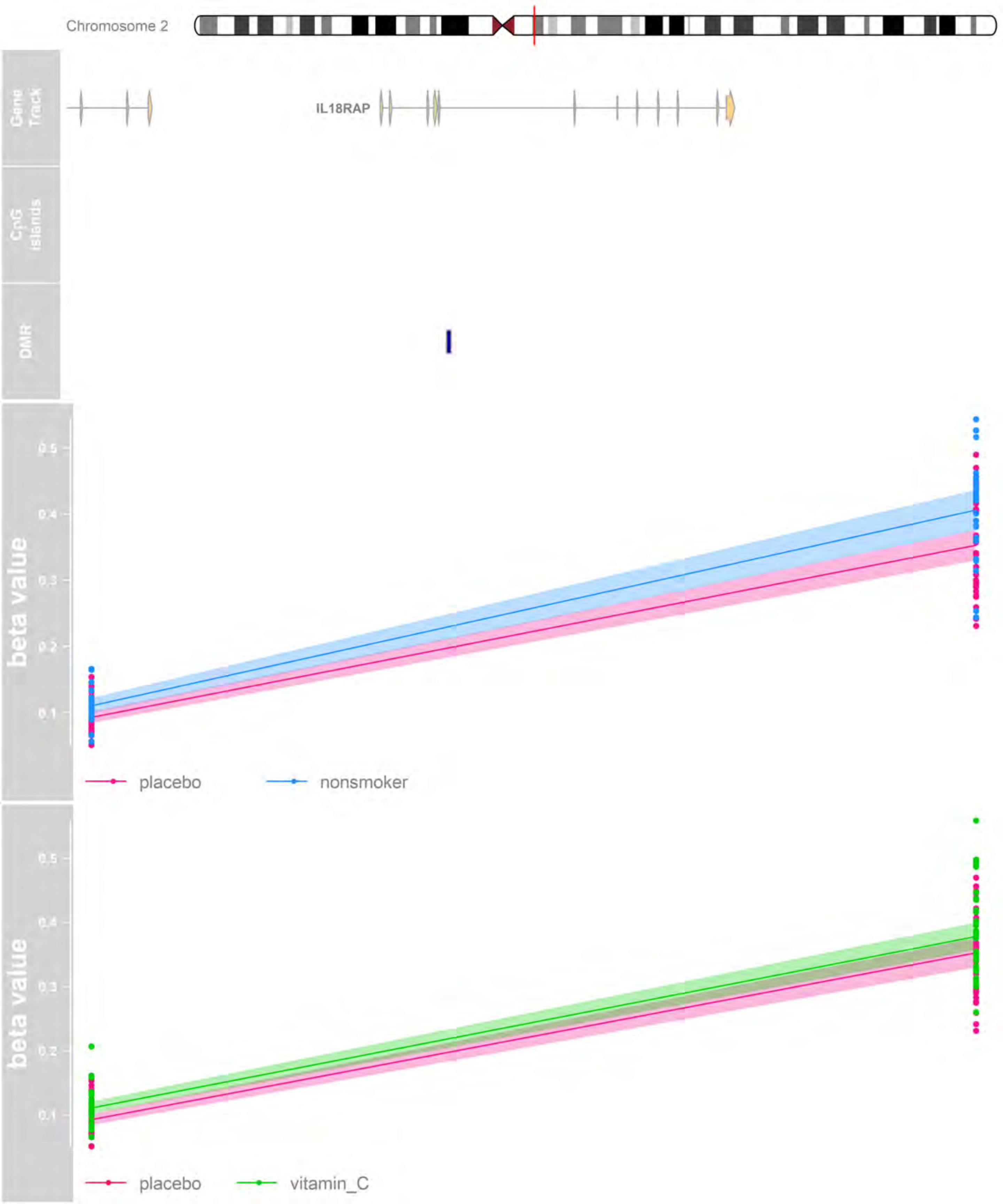

Chromosome 2
Gene Track
IL18RAP
CpG islands
DMR
beta value
0.5
0.4
0.3
0.2
0.1
placebo
nonsmoker
beta value
0.5
0.4
0.3
0.2
0.1
placebo
vitamin_C

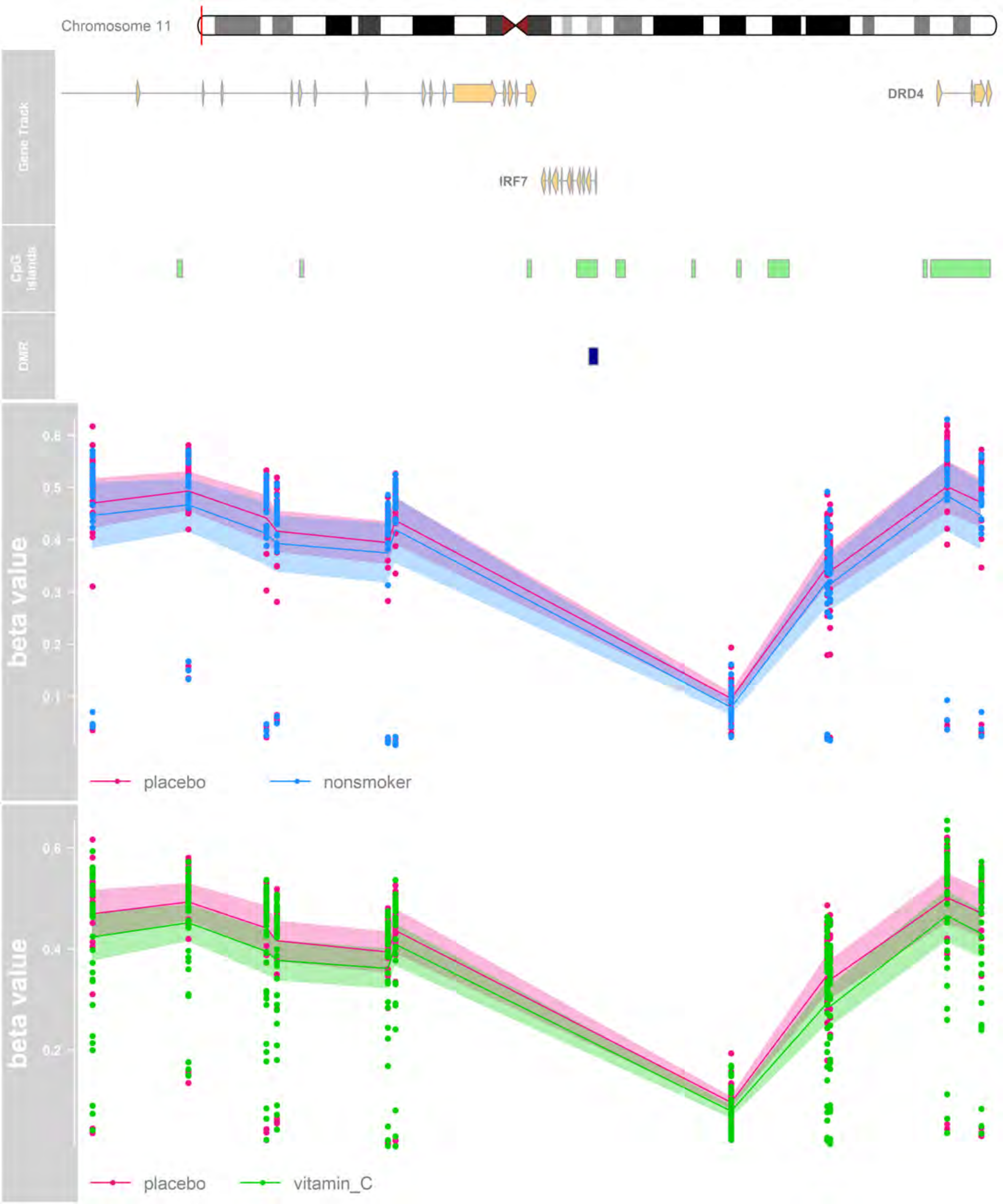

Chromosome 11
Gene Track
DRD4
IRF7
CpG Islands
DMR
beta value
0.6
0.5
0.4
0.3
0.2
0.1
placebo
nonsmoker
beta value
0.6
0.4
0.2
placebo
vitamin_C

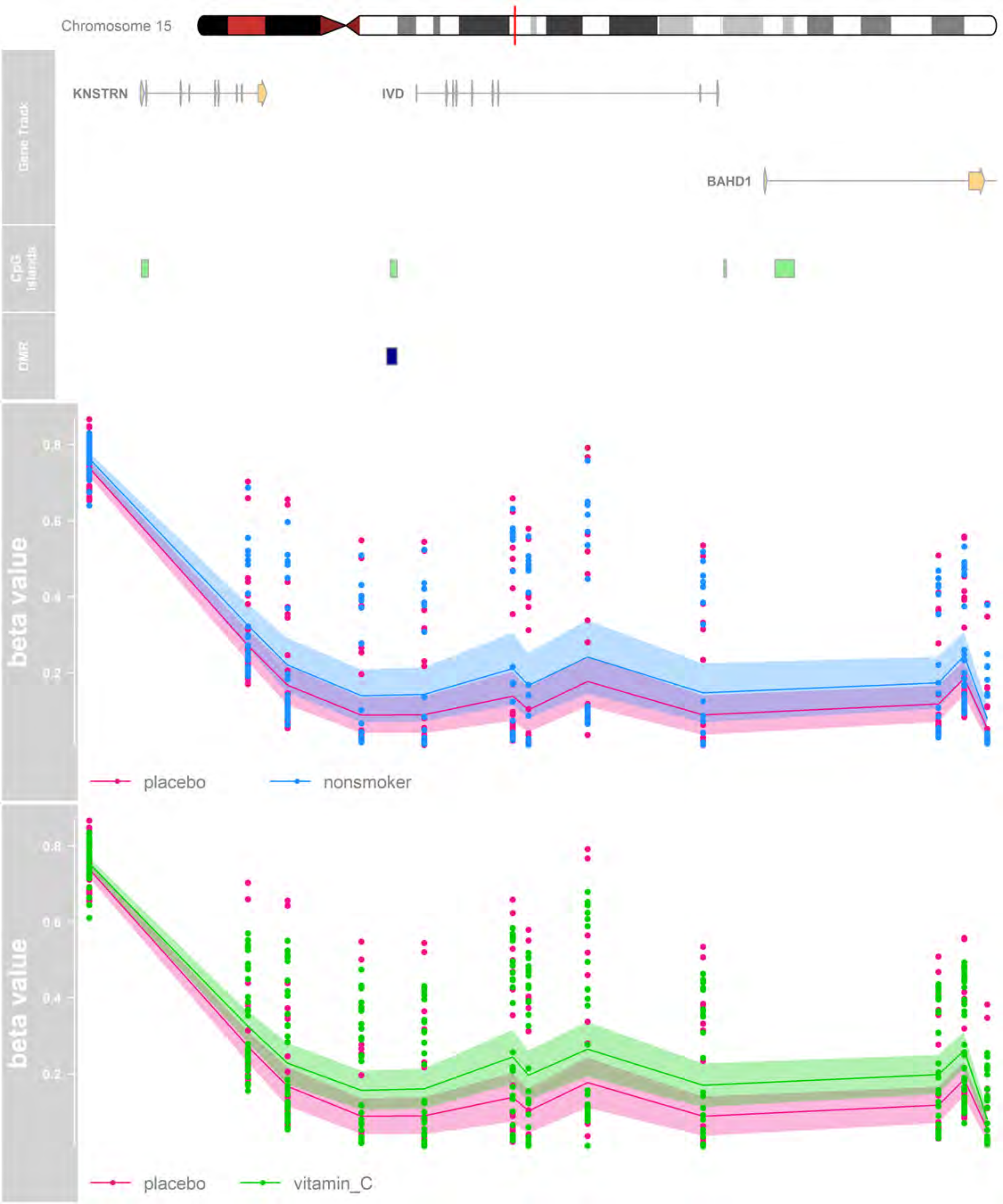

Chromosome 15
Gene Track
KNSTRN
IVD
BAHD1
CpG Islands
DMR
beta value
0.8
0.6
0.4
0.2
placebo
nonsmoker
beta value
0.8
0.6
0.4
0.2
placebo
vitamin_C

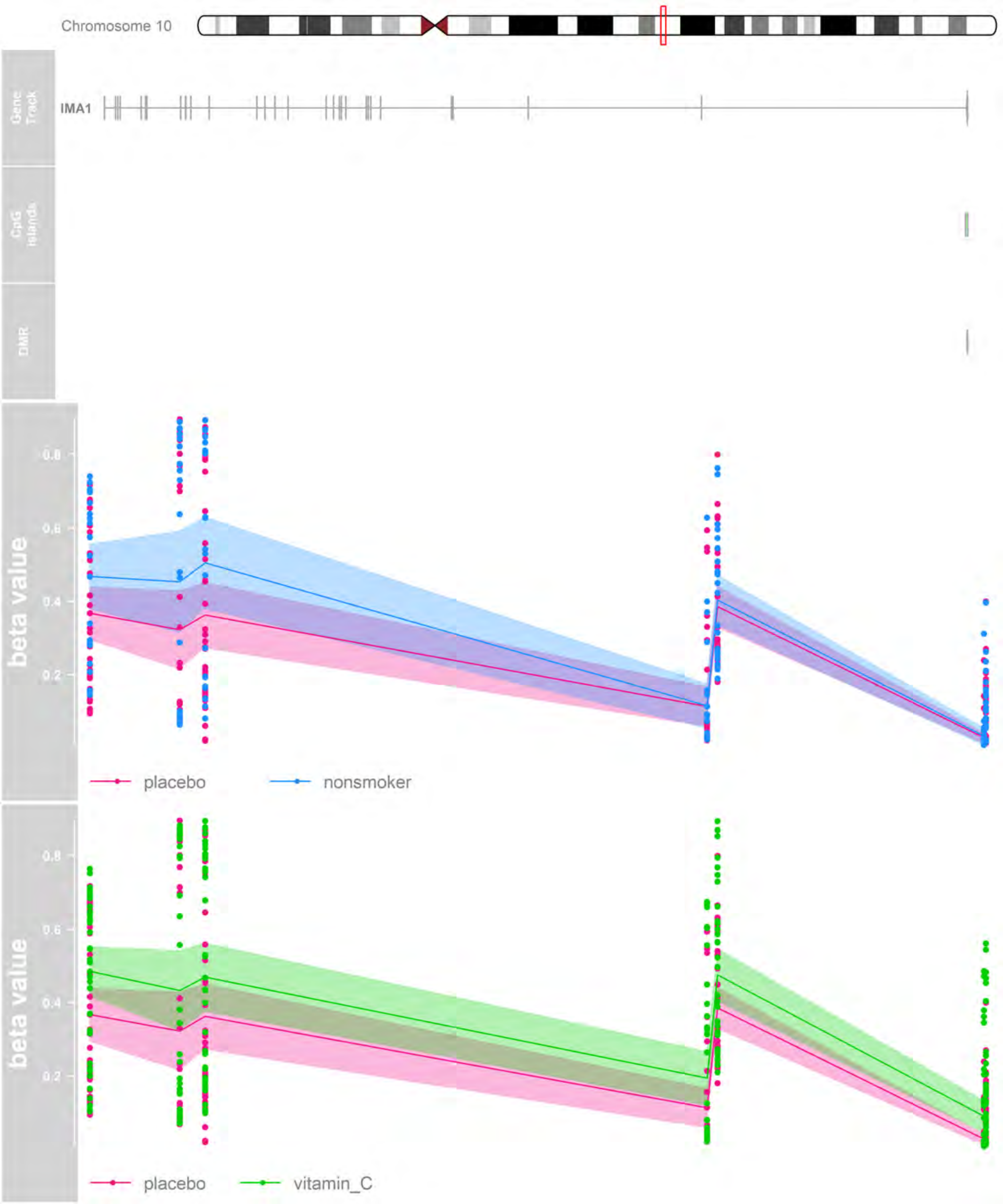

Chromosome 10
Gene Track
IMA1
CpG islands
DMR
beta value
0.8
0.6
0.4
0.2
placebo
nonsmoker
beta value
0.8
0.6
0.4
0.2
placebo
vitamin_C

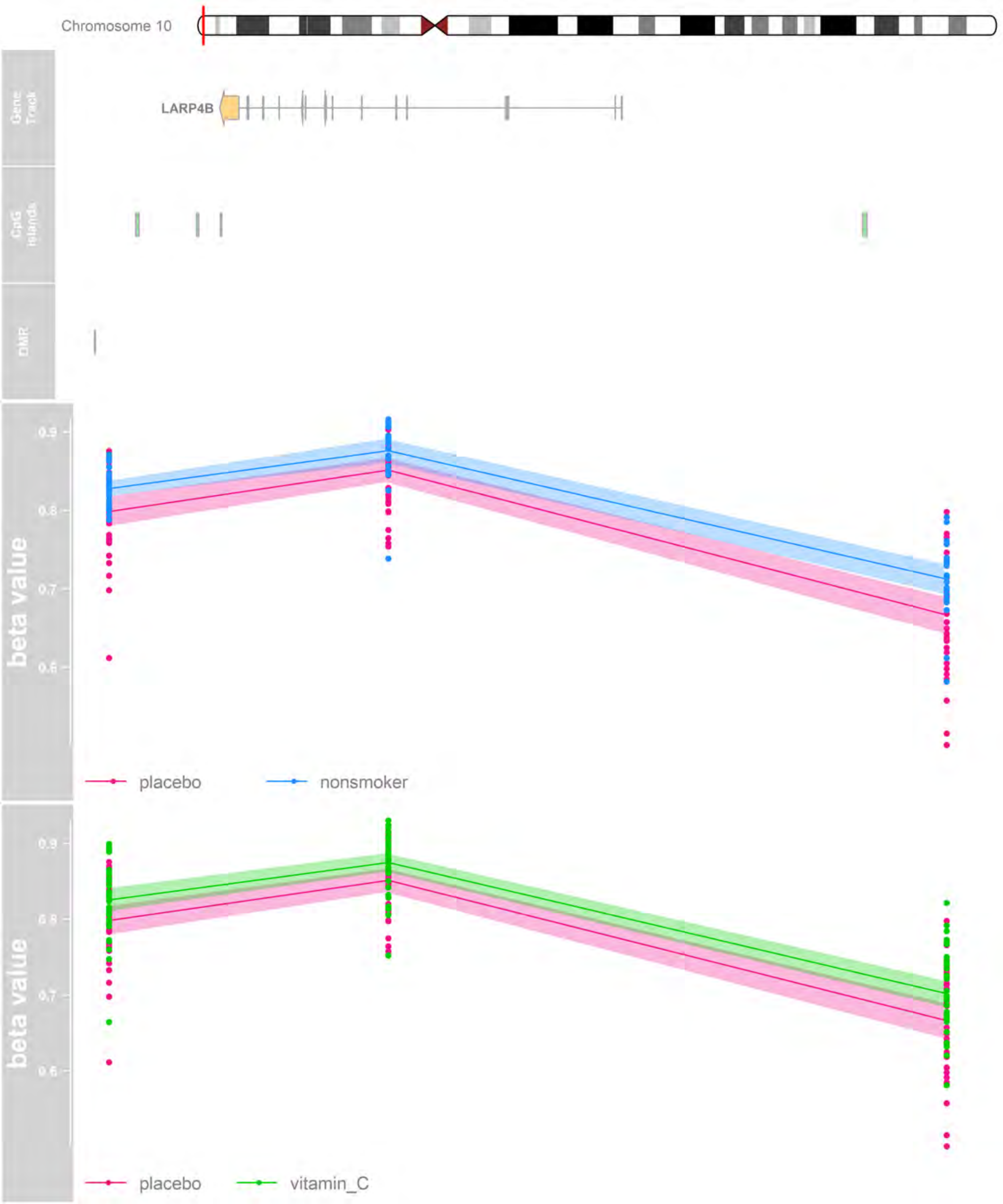

Chromosome 10
Gene Track
LARP4B
CpG islands
DMR
beta value
0.9
0.8
0.7
0.6
placebo
nonsmoker
beta value
0.9
0.8
0.7
0.6
placebo
vitamin_C

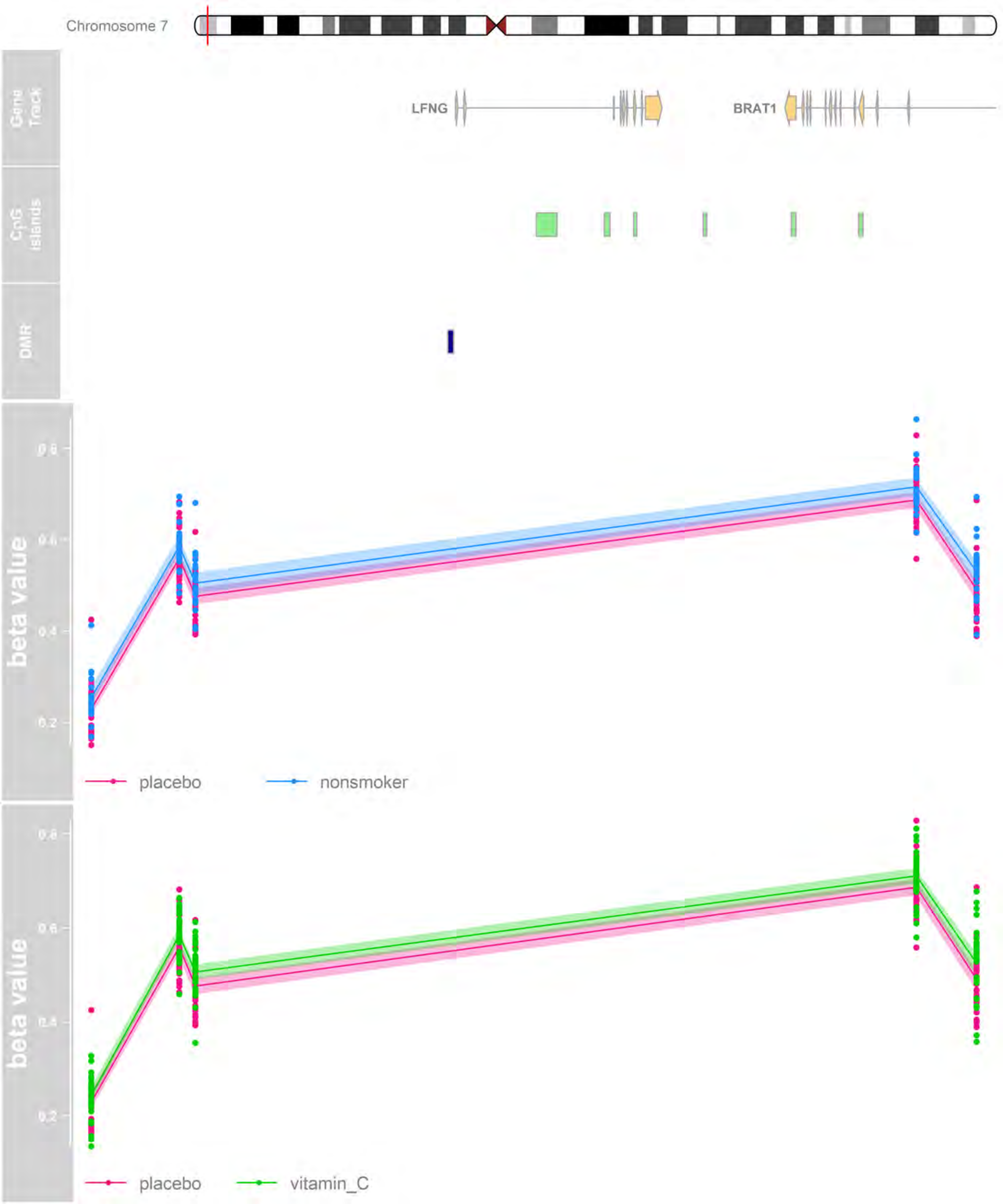

Chromosome 7
Gene Track
LFNG
BRAT1
CpG islands
DMR
beta value
0.8
0.6
0.4
0.2
placebo
nonsmoker
beta value
0.8
0.6
0.4
0.2
placebo
vitamin_C

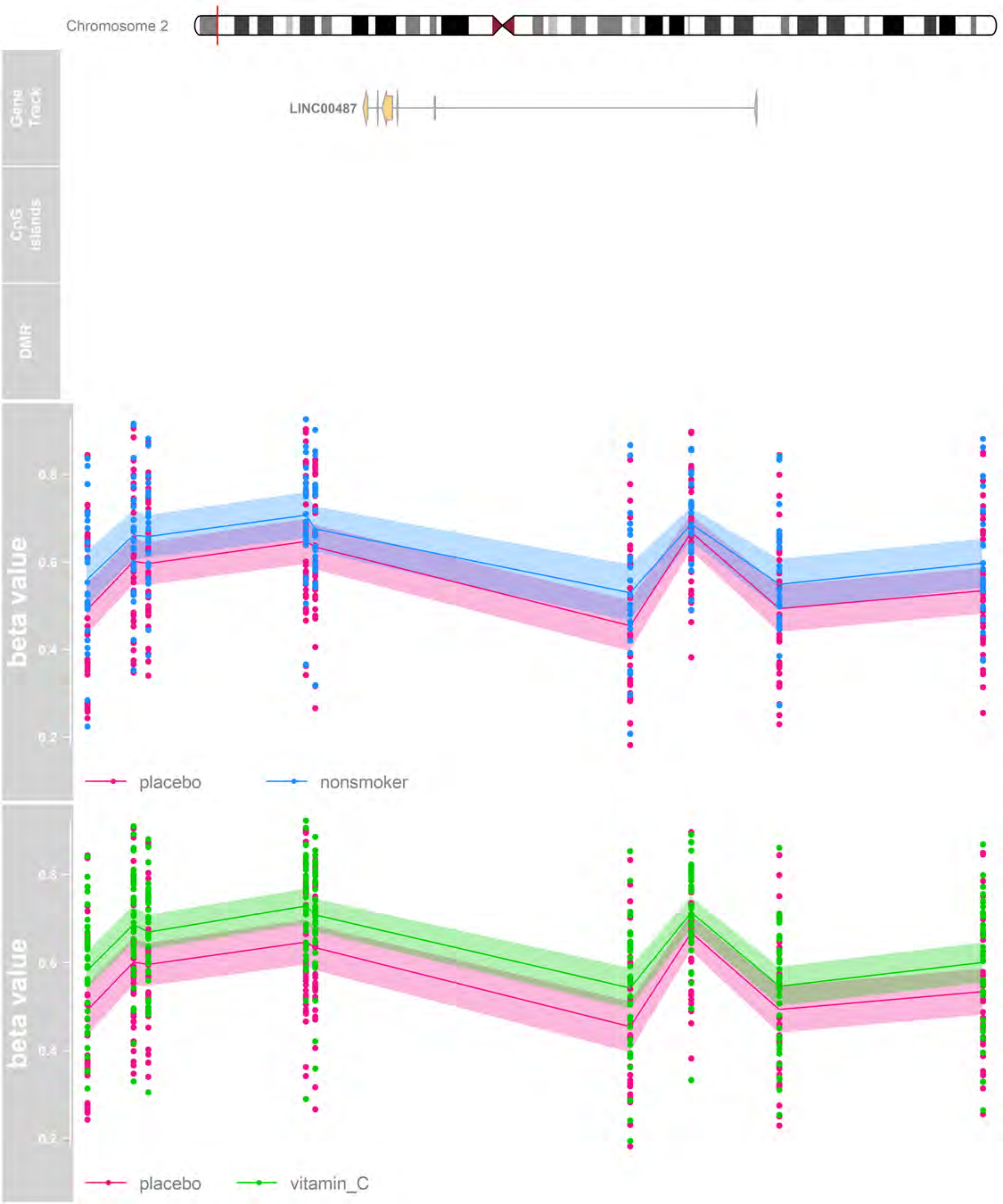

Chromosome 2
Gene Track
LINC00487
CpG Islands
DMR
beta value
0.8
0.6
0.4
0.2
placebo
nonsmoker
beta value
0.8
0.6
0.4
0.2
placebo
vitamin_C

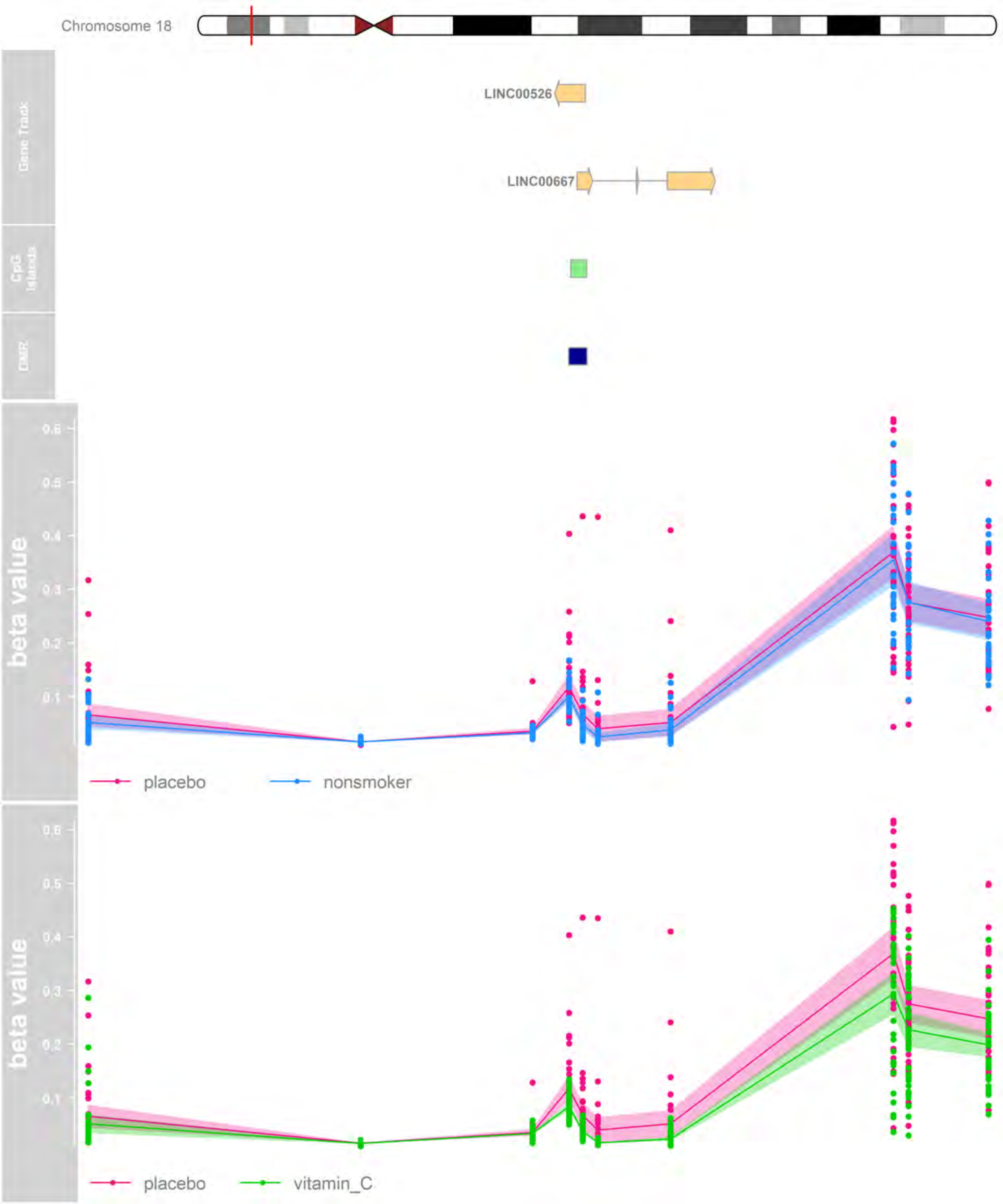

Chromosome 18
Gene Track
LINC00526
LINC00667
CpG Islands
DMR
beta value
0.6
0.5
0.4
0.3
0.2
0.1
placebo
nonsmoker
beta value
0.6
0.5
0.4
0.3
0.2
0.1
placebo
vitamin_C

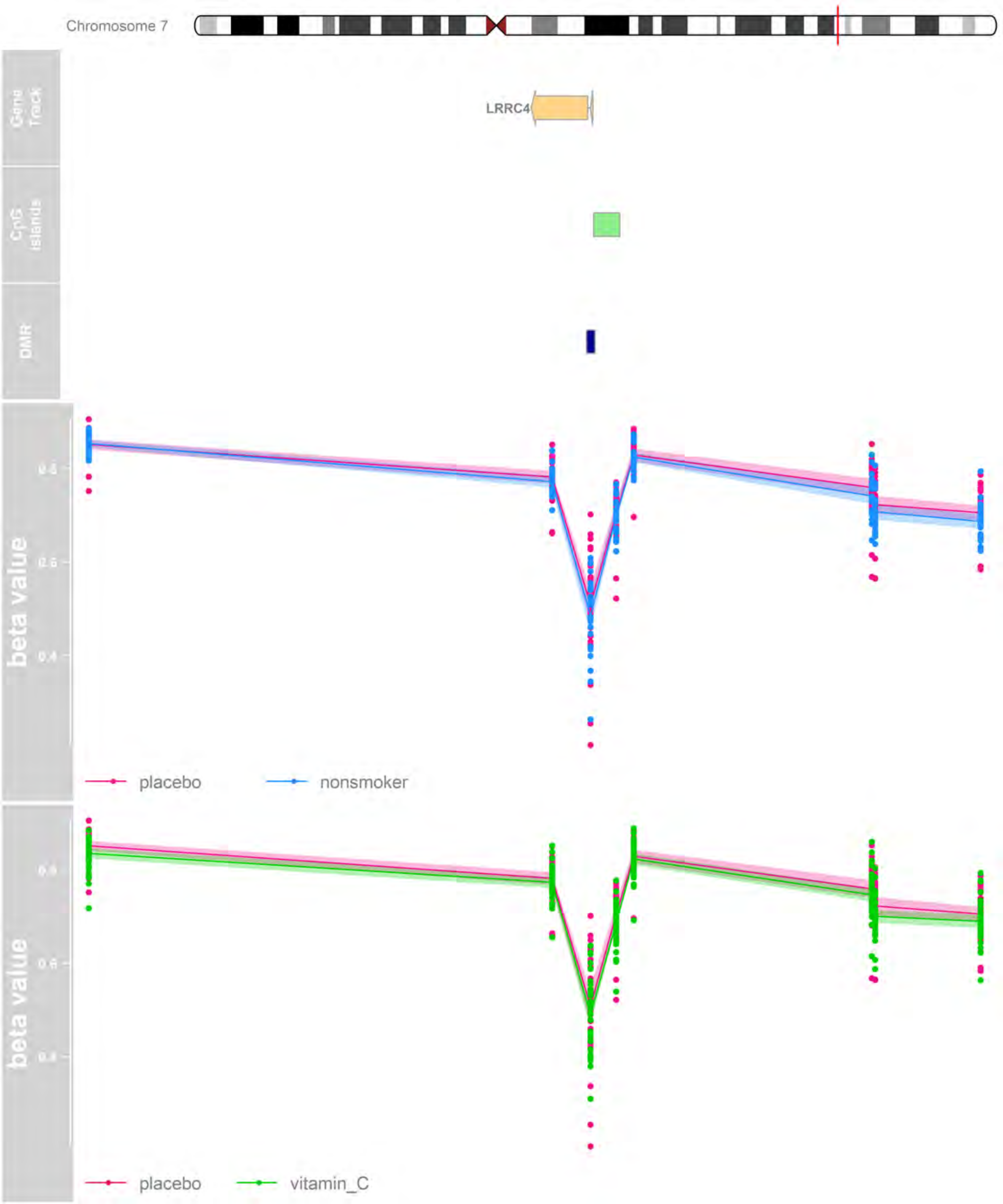
Chromosome 7
Gene Track
LRRC4
CpG islands
DMR
beta value
placebo
nonsmoker
beta value
placebo
vitamin_C

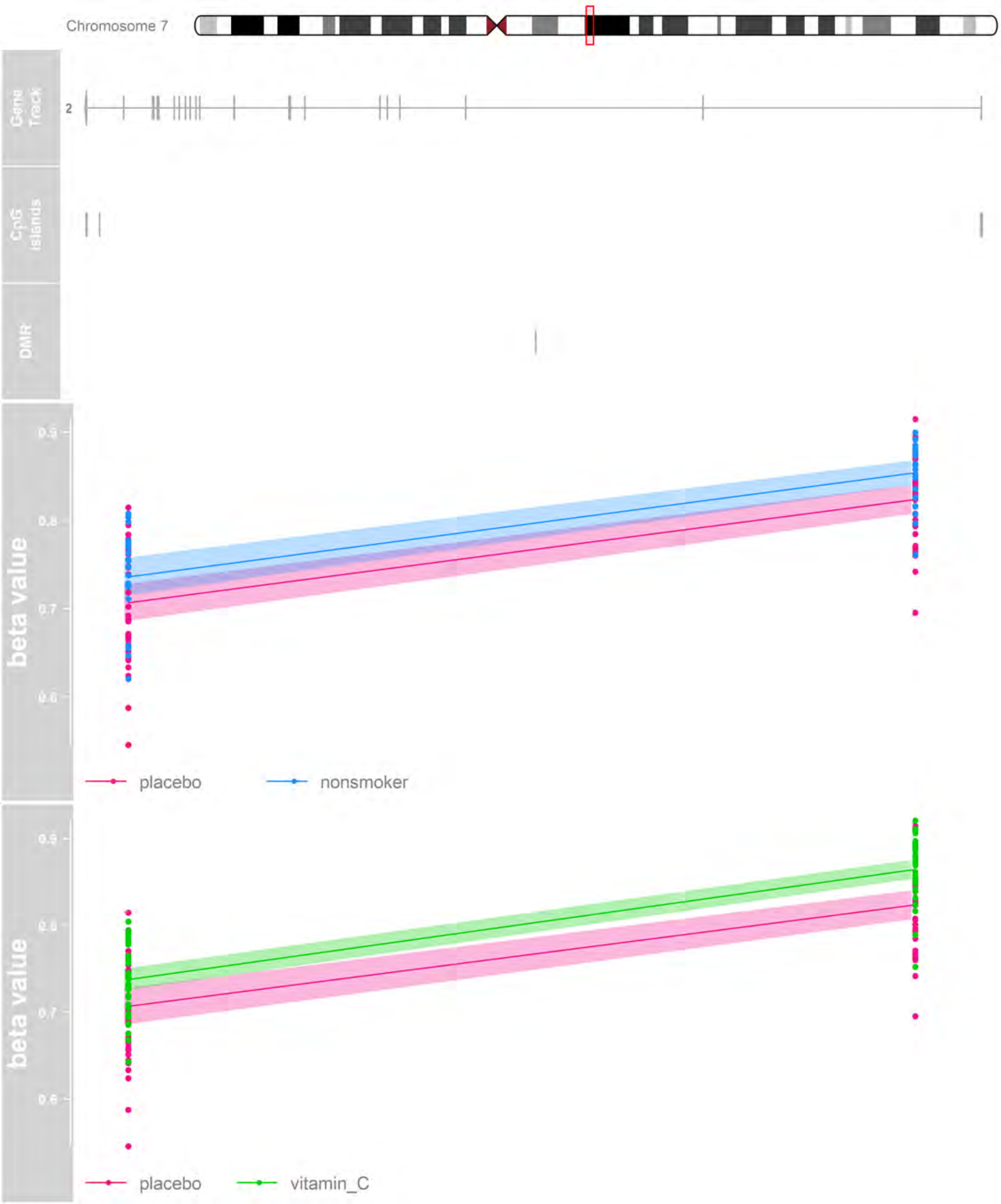
Chromosome 7
Gene Track
2
CpG Islands
DMR
beta value
0.9
0.8
0.7
0.6
placebo
nonsmoker
beta value
0.9
0.8
0.7
0.6
placebo
vitamin_C

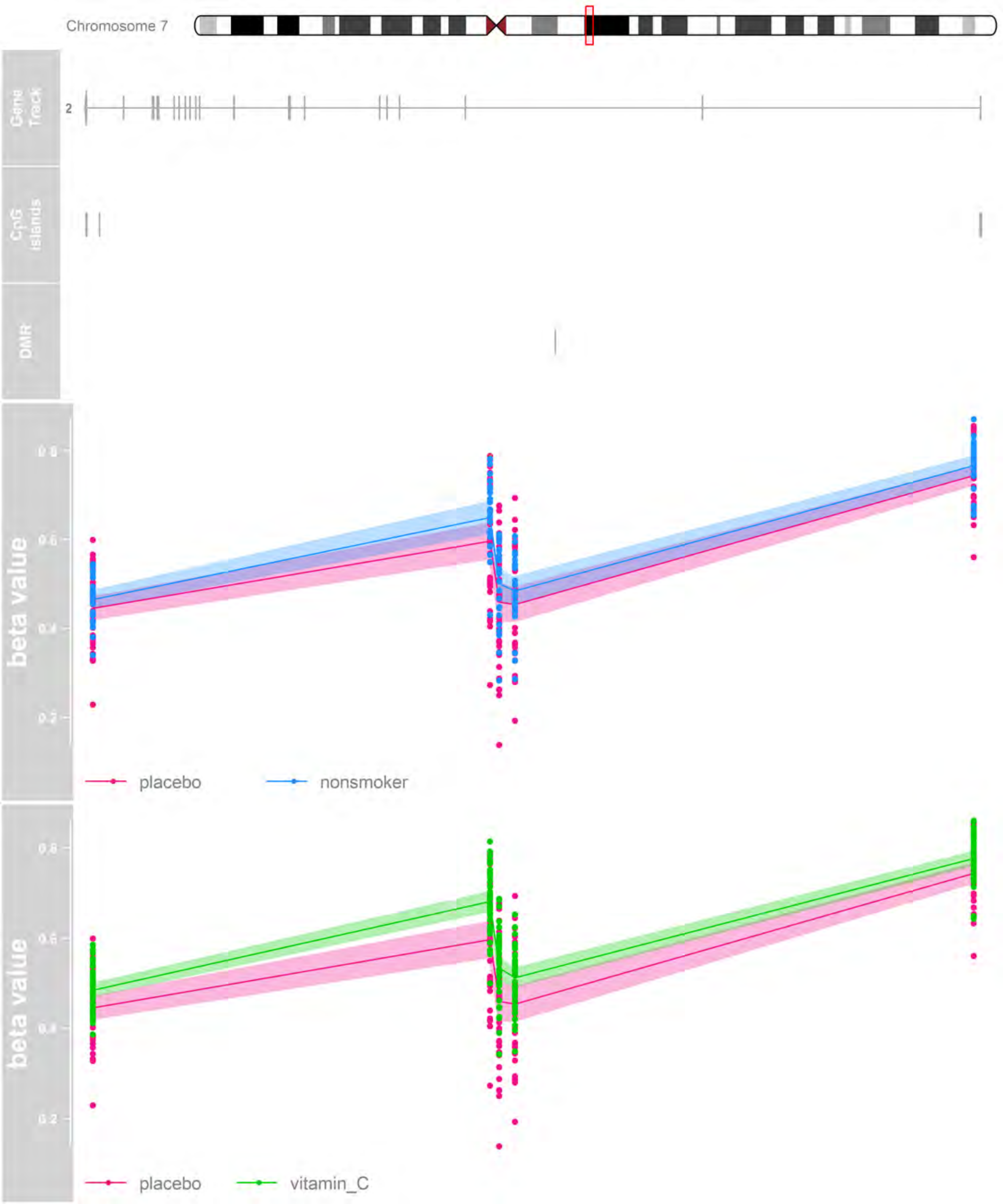

Chromosome 7
Gene Track
2
CpG islands
DMR
beta value
0.8
0.6
0.4
0.2
placebo
nonsmoker
beta value
0.8
0.6
0.4
0.2
placebo
vitamin_C

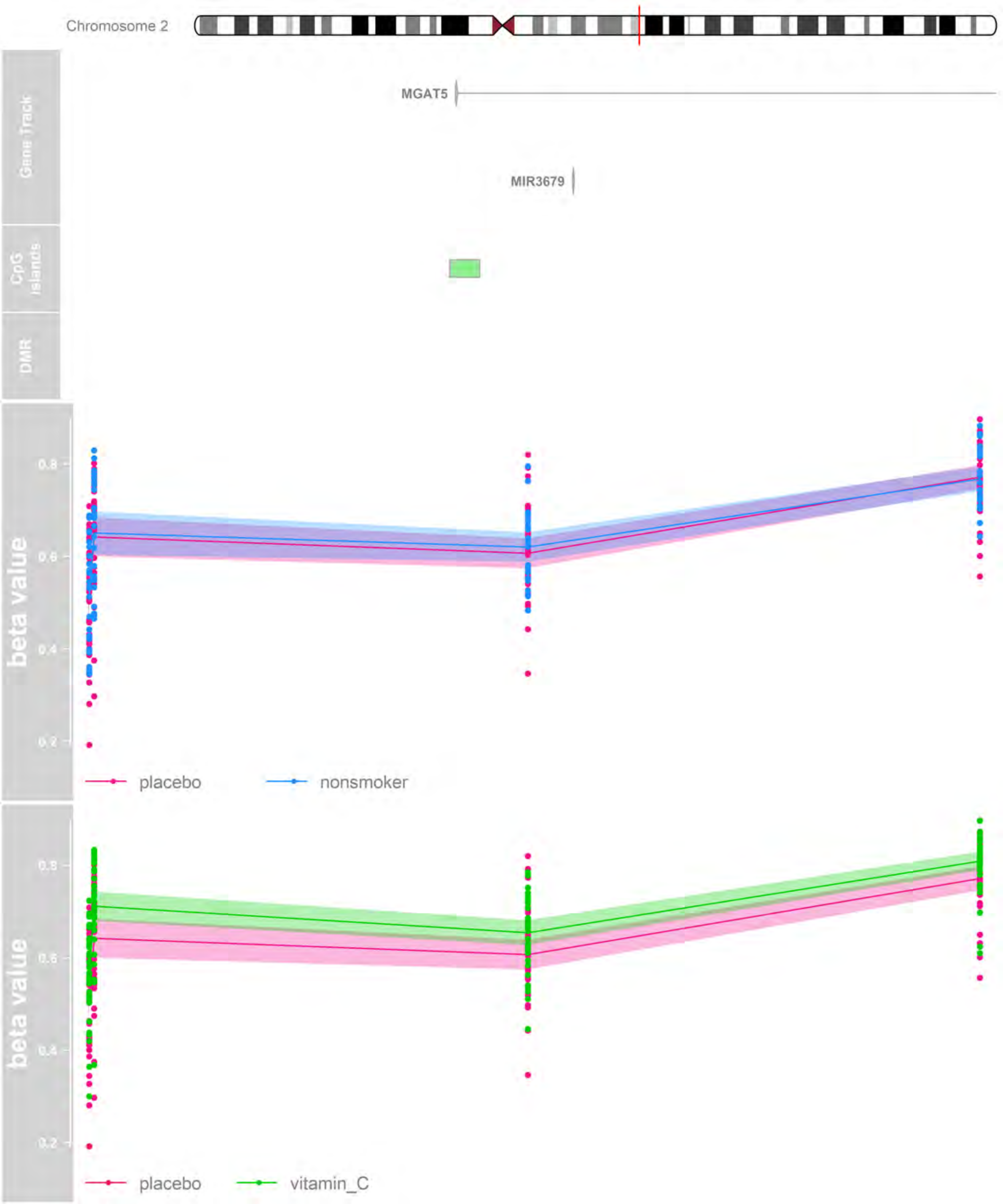
Chromosome 2
Gene Track
MGAT5
MIR3679
CpG islands
DMR
beta value
0.8
0.6
0.4
0.2
placebo
nonsmoker
beta value
0.8
0.6
0.4
0.2
placebo
vitamin_C

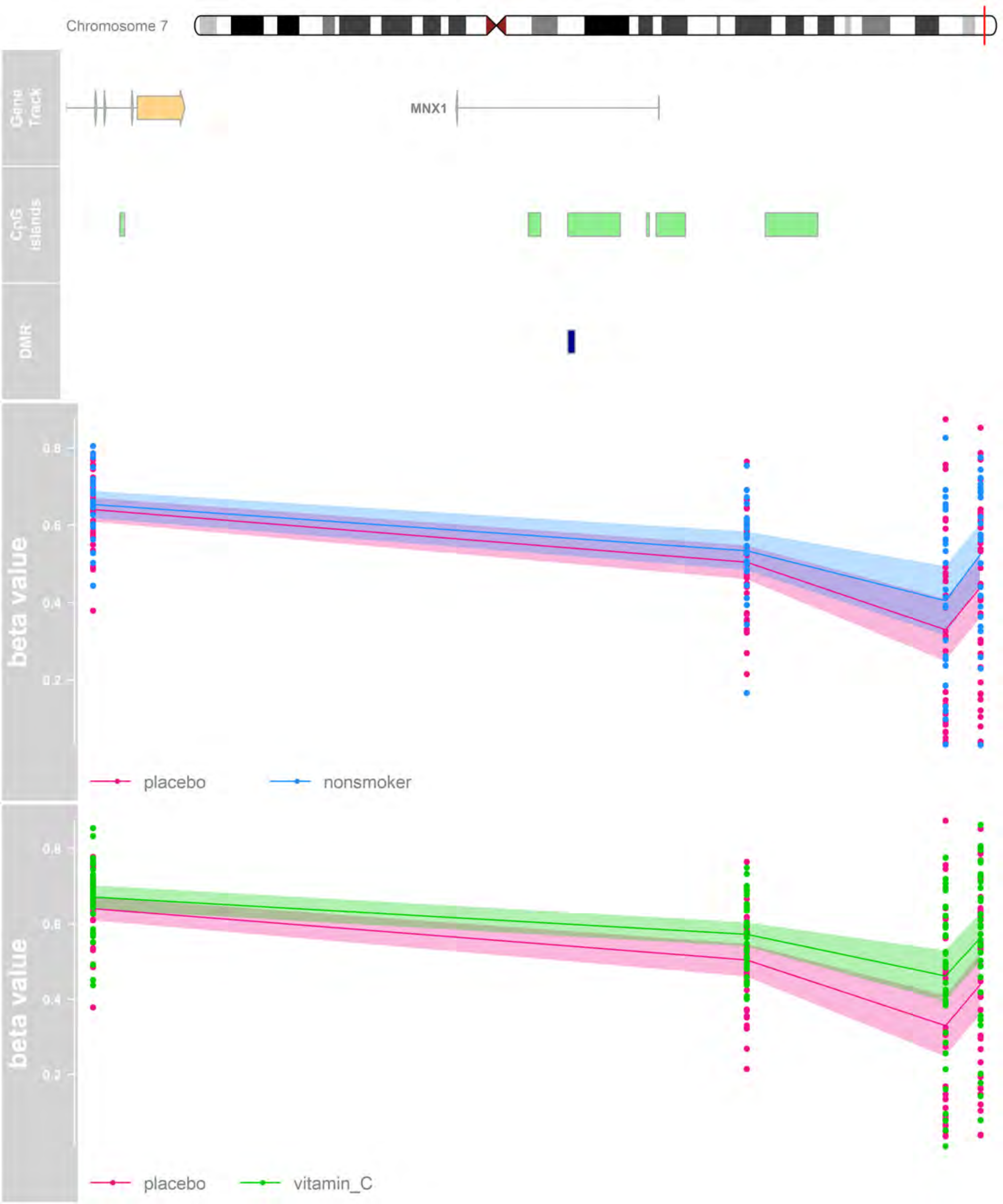
Chromosome 7
Gene Track
MNX1
CpG Islands
DMR
beta value
0.8
0.6
0.4
0.2
placebo
nonsmoker
beta value
0.8
0.6
0.4
0.2
placebo
vitamin_C

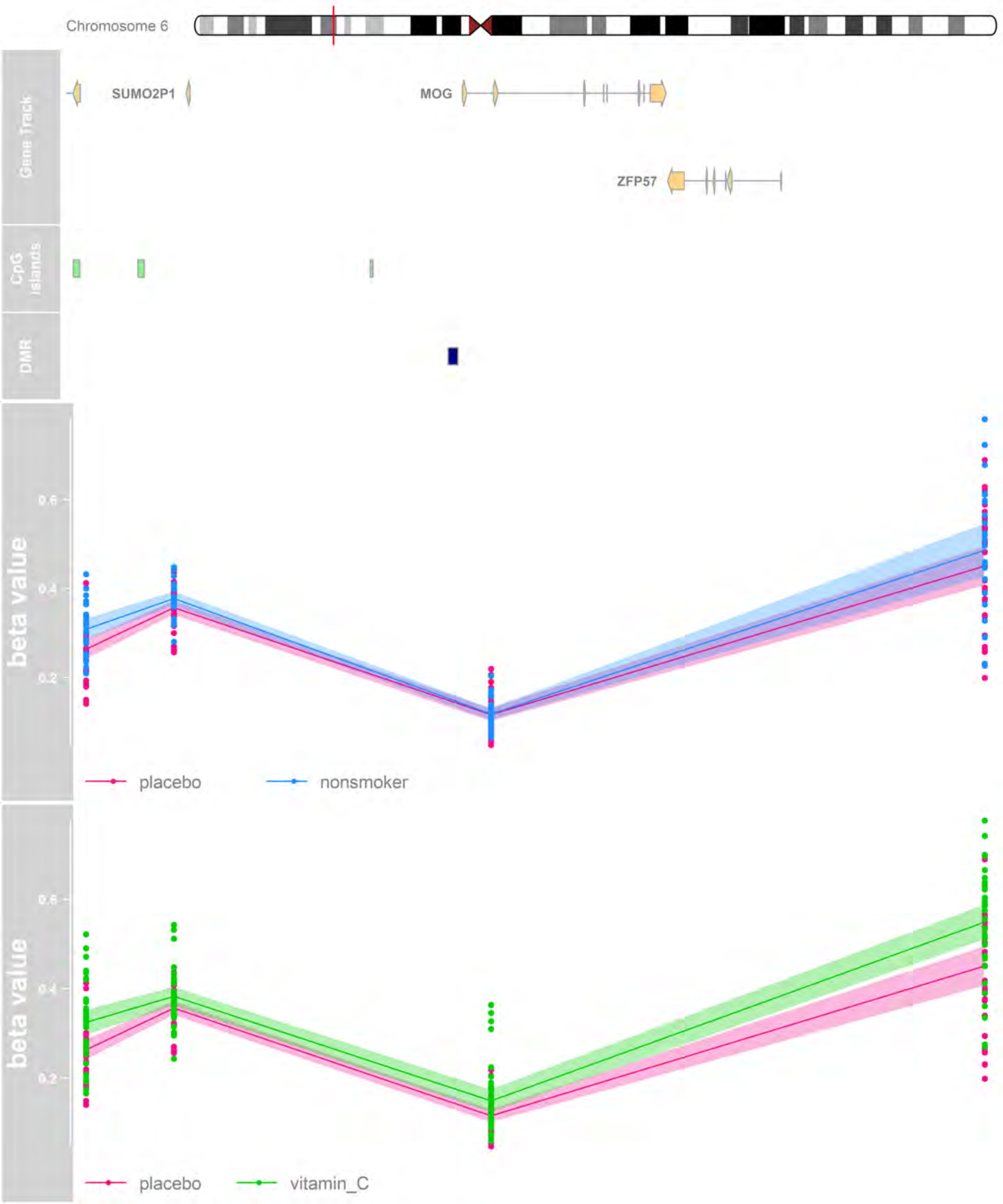

Chromosome 6
Gene Track
SUMO2P1
MOG
ZFP57
CpG islands
DMR
beta value
0.6
0.4
0.2
placebo
nonsmoker
beta value
0.6
0.4
0.2
placebo
vitamin_C

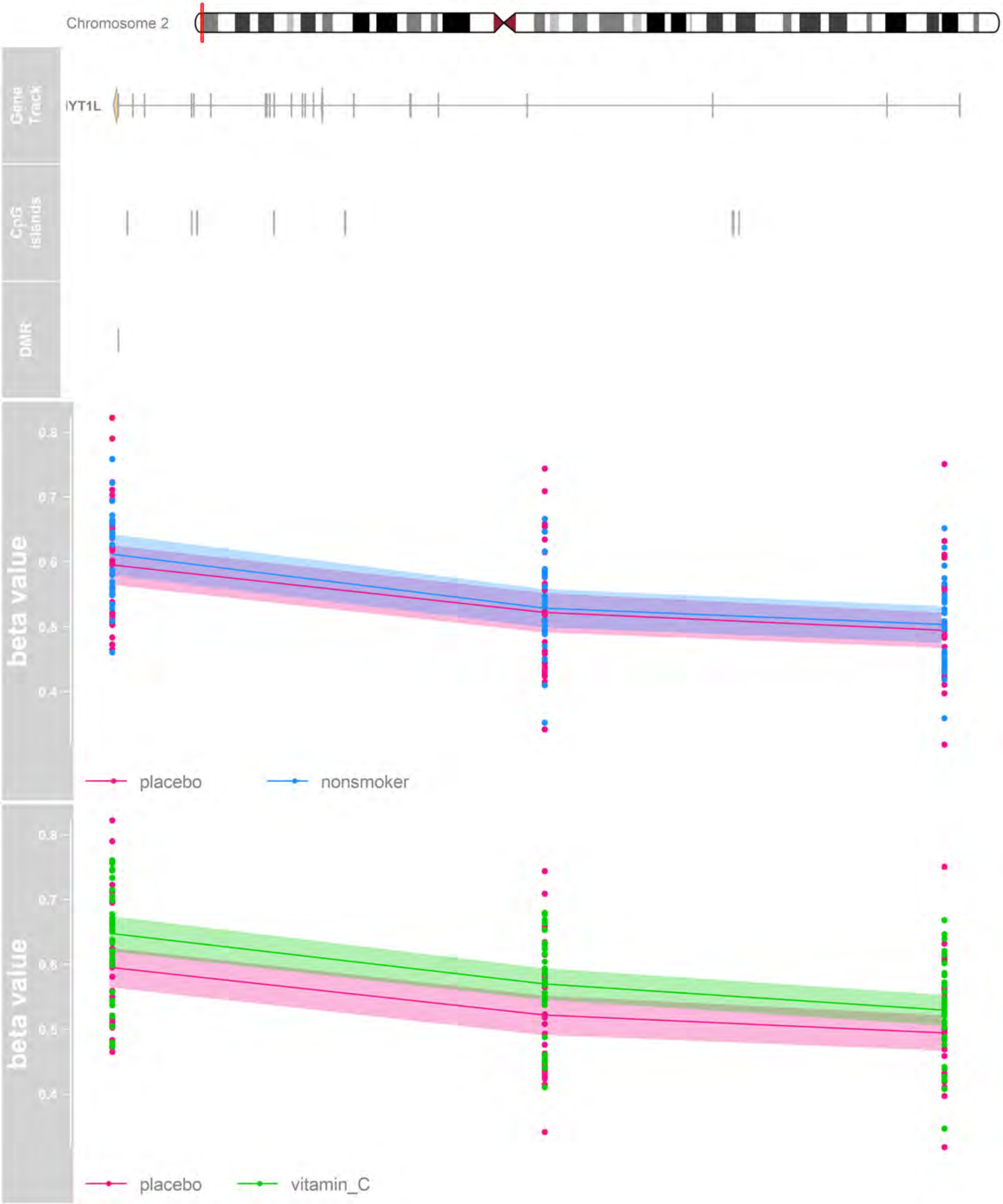

Chromosome 2
Gene Track
CpG islands
DMR
beta value
placebo
nonsmoker
beta value
placebo
vitamin_C

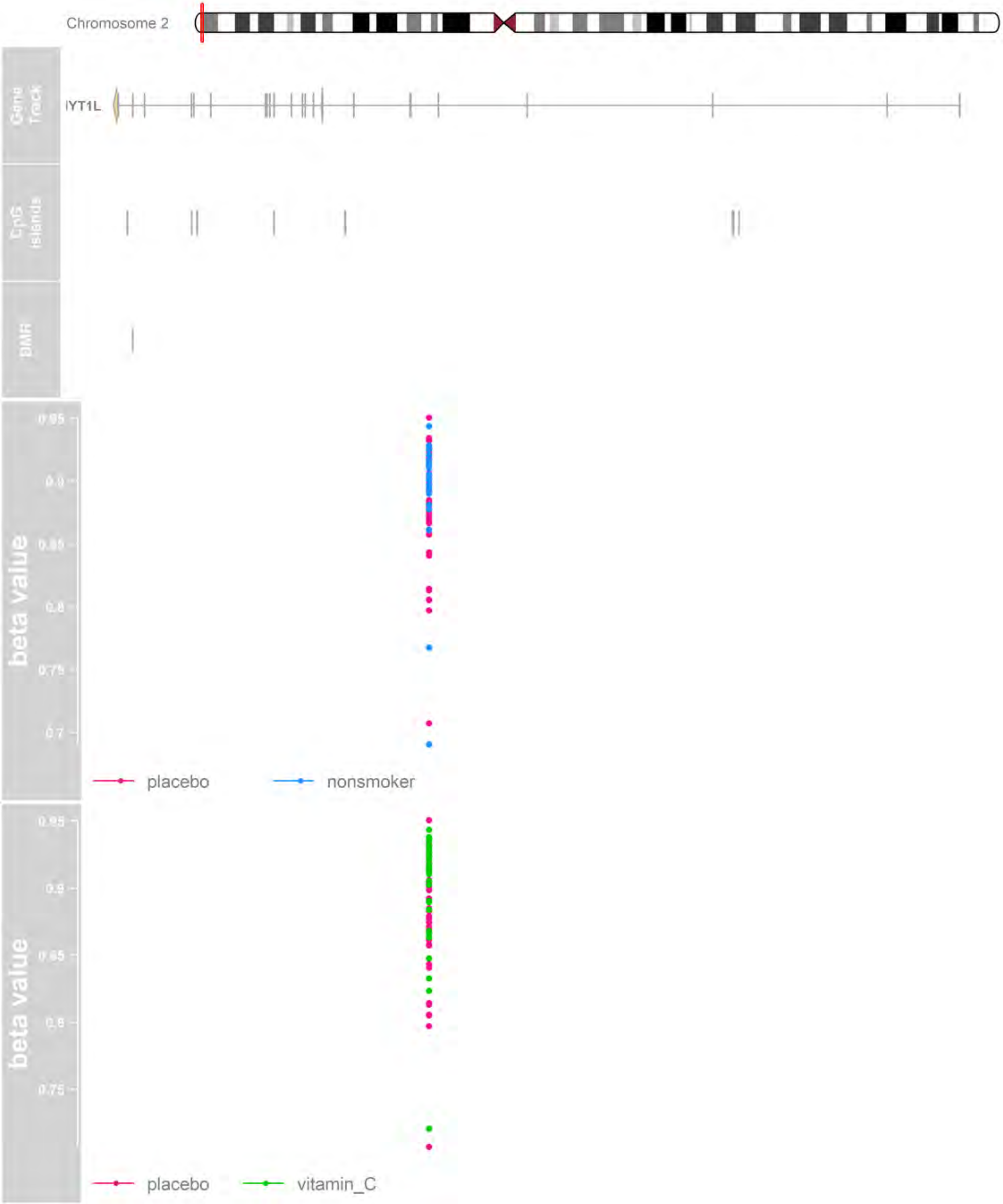

Chromosome 2
Gene Track
IYT1L
CpG islands
DMR
beta value
0.95
0.9
0.85
0.8
0.75
0.7
placebo
nonsmoker
beta value
0.95
0.9
0.85
0.8
0.75
placebo
vitamin_C

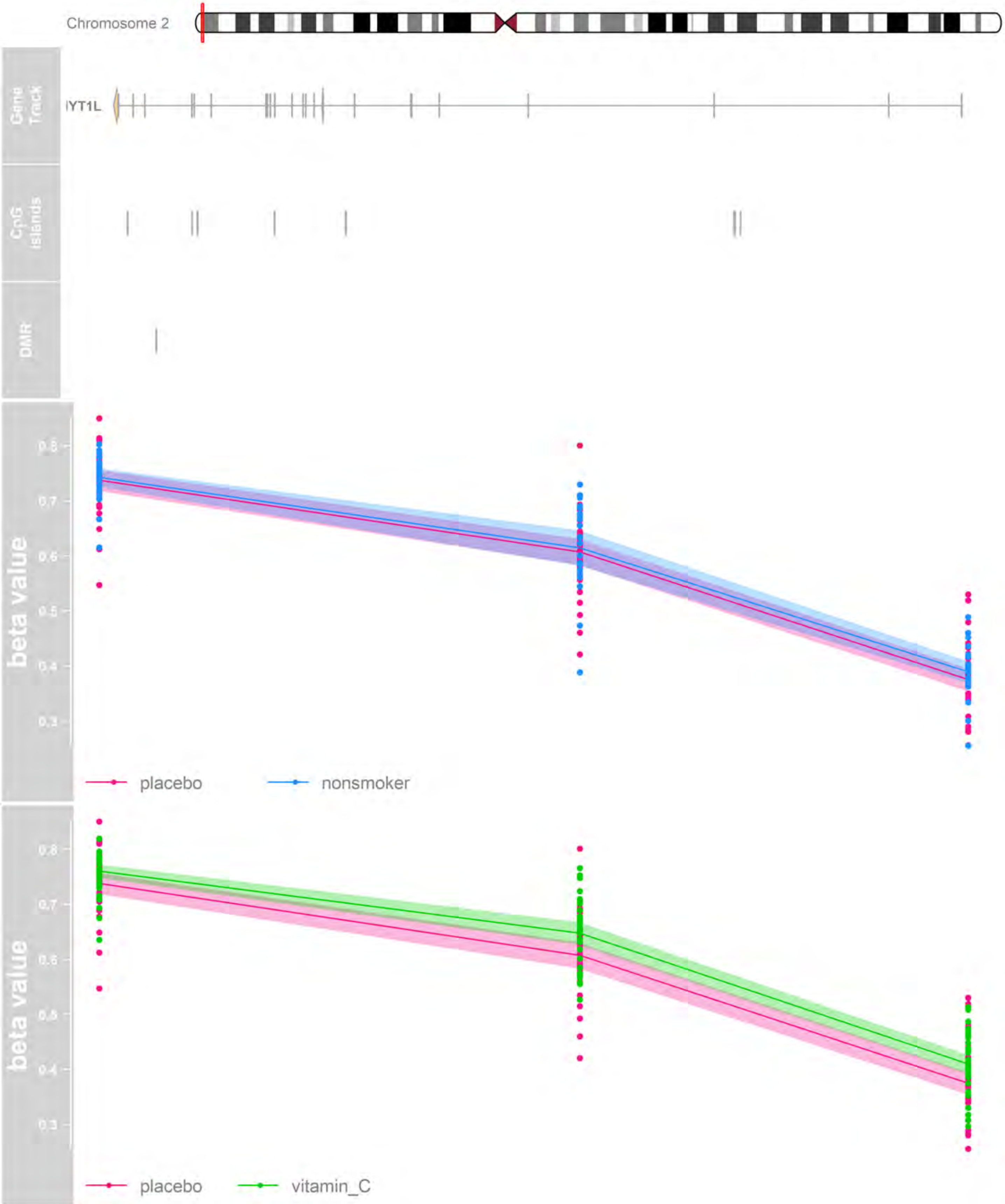
Chromosome 2
Gene Track
YT1L
CpG Islands
DMR
beta value
0.8
0.7
0.6
0.5
0.4
0.3
placebo
nonsmoker
beta value
0.8
0.7
0.6
0.5
0.4
0.3
placebo
vitamin_C

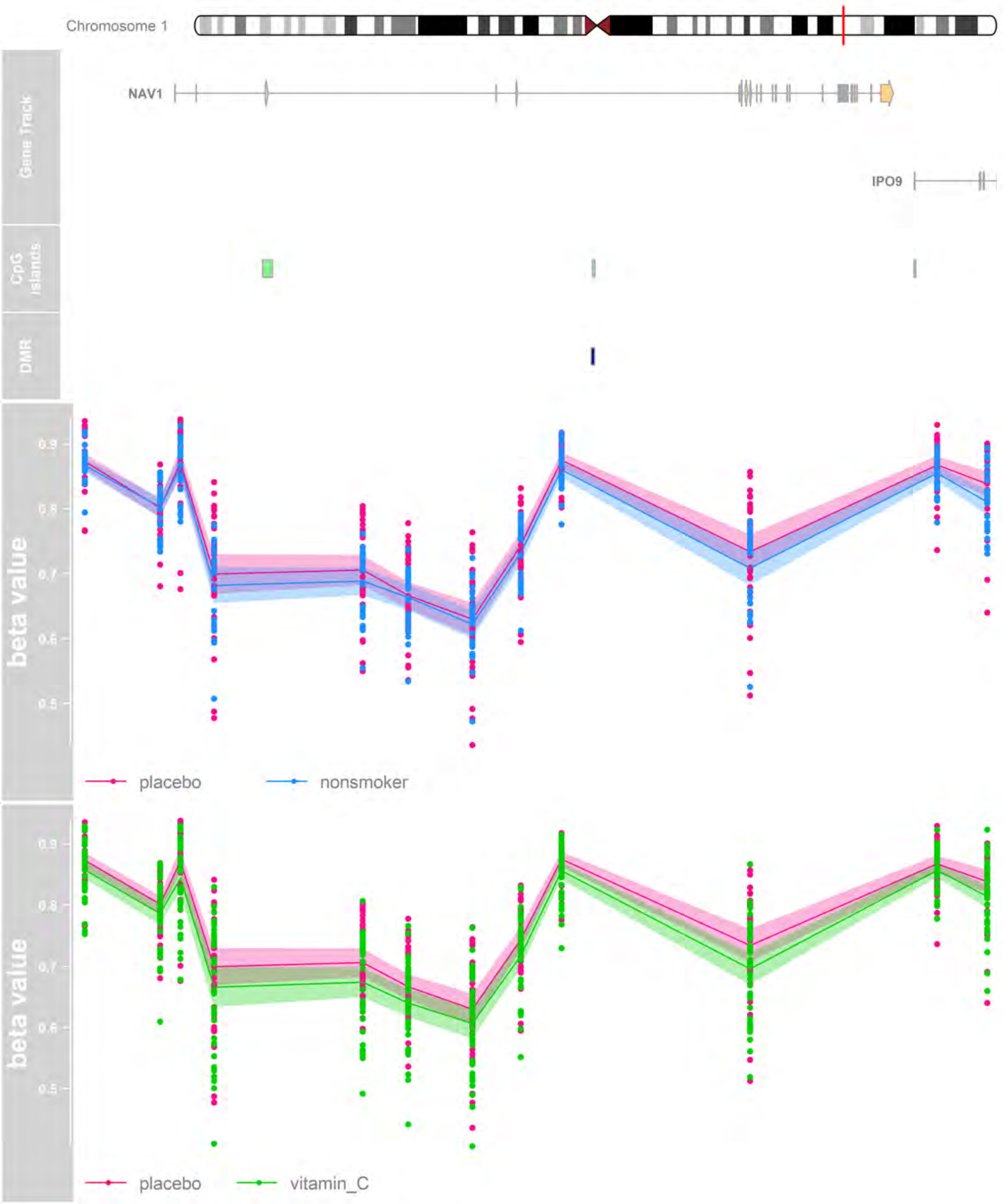

Chromosome 1
Gene Track
NAV1
IPO9
CpG islands
DMR
beta value
0.9
0.8
0.7
0.6
0.5
placebo
nonsmoker
beta value
0.9
0.8
0.7
0.6
0.5
placebo
vitamin_C

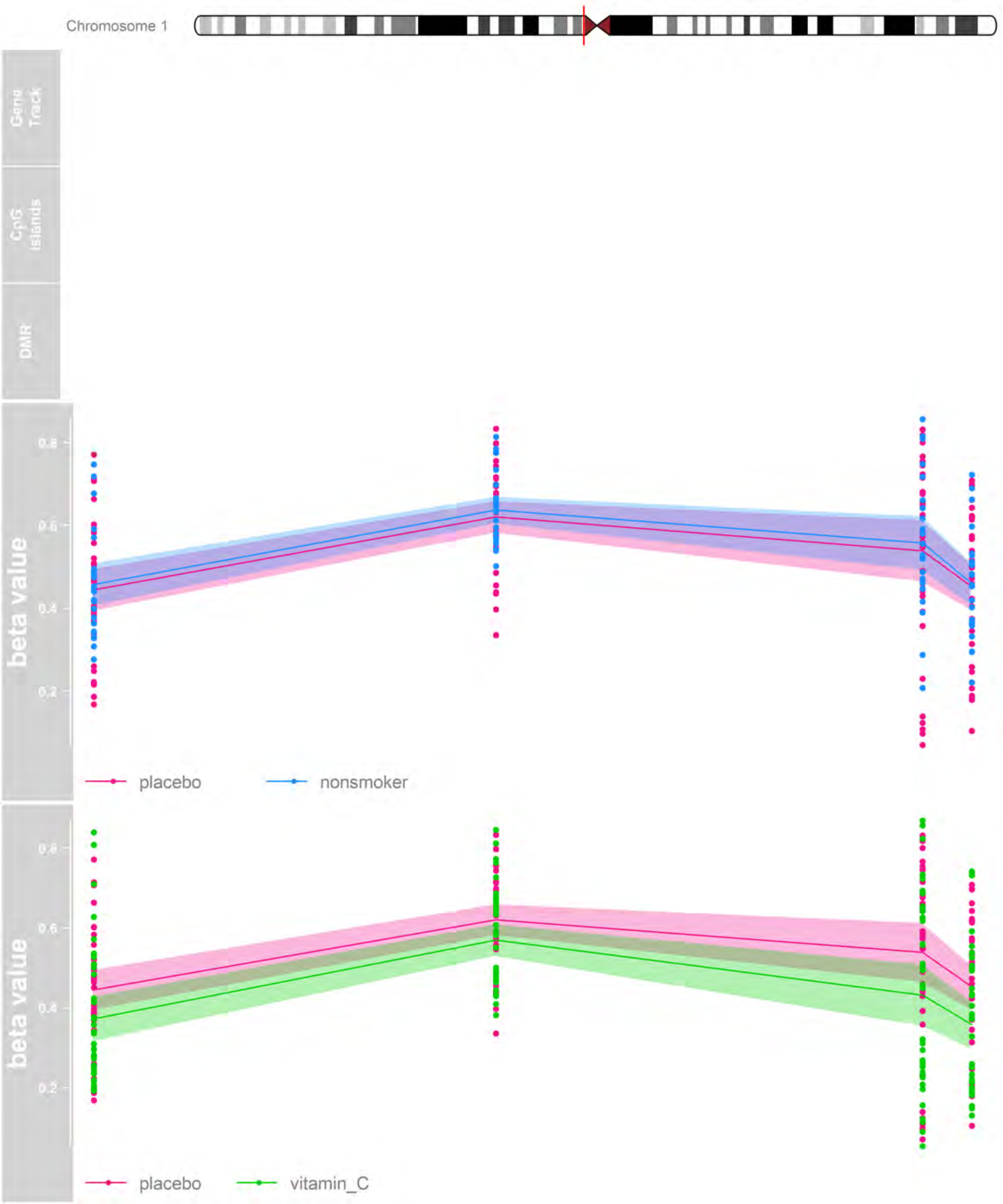

Chromosome 1
Gene Track
CpG Islands
DMR
beta value
0.8
0.6
0.4
0.2
placebo
nonsmoker
beta value
0.8
0.6
0.4
0.2
placebo
vitamin_C

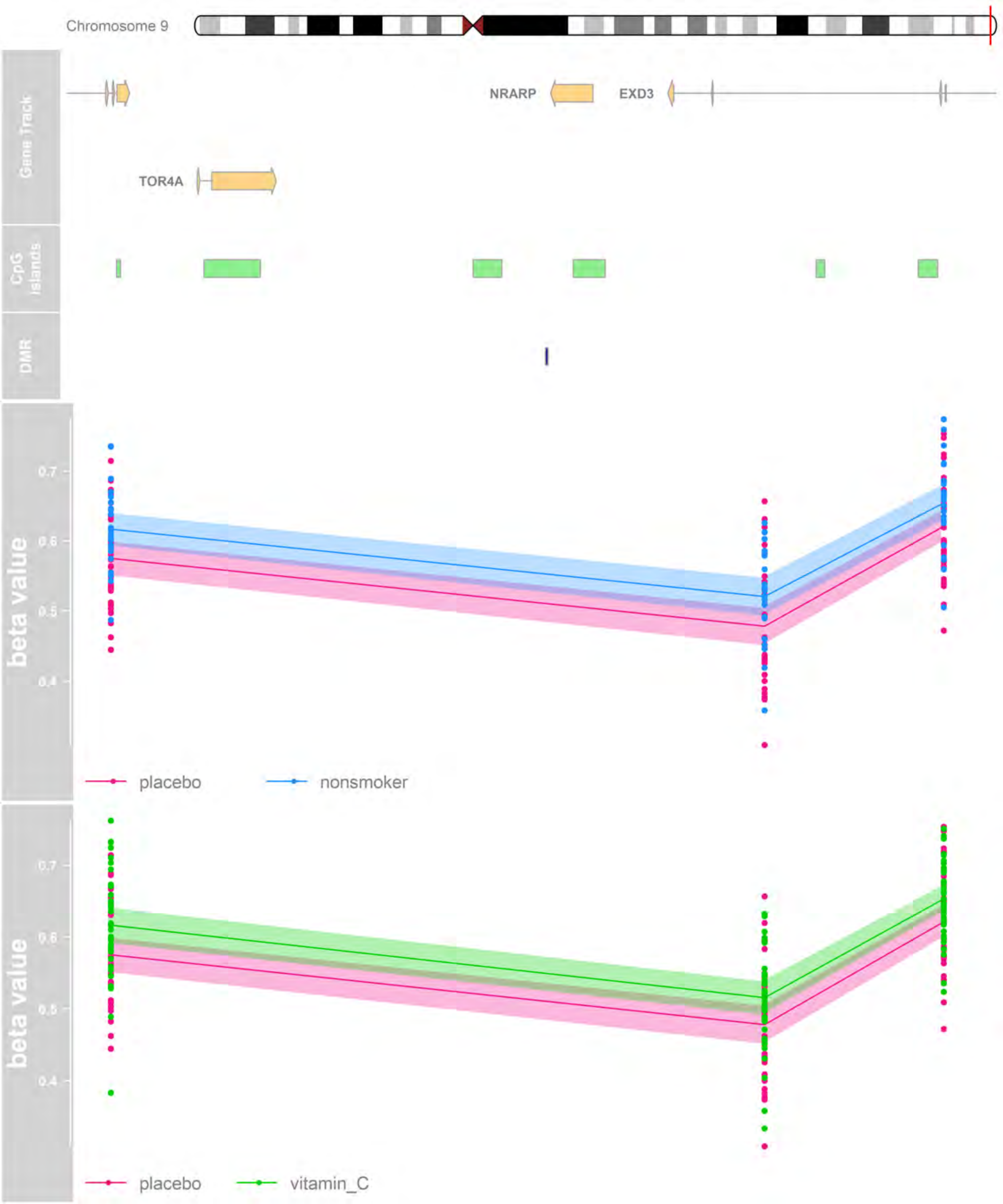
Chromosome 9
Gene Track
NRARP
EXD3
TOR4A
CpG islands
DMR
beta value
0.7
0.6
0.5
0.4
placebo
nonsmoker
beta value
0.7
0.6
0.5
0.4
placebo
vitamin_C

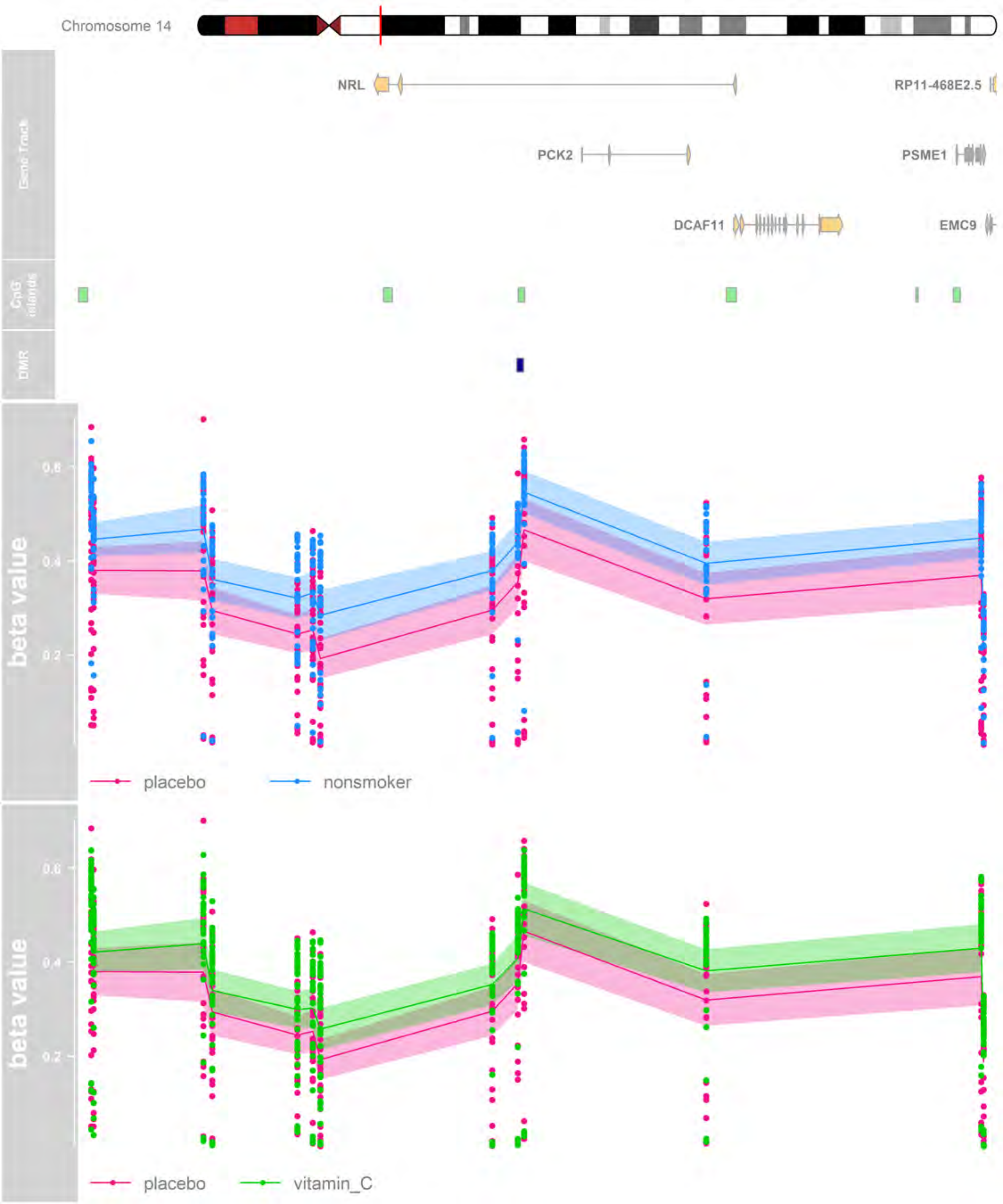

Chromosome 14
Gene Track
NRL
RP11-468E2.5
PCK2
PSME1
DCAF11
EMC9
CpG islands
DMR
beta value
0.6
0.4
0.2
placebo
nonsmoker
beta value
0.6
0.4
0.2
placebo
vitamin_C

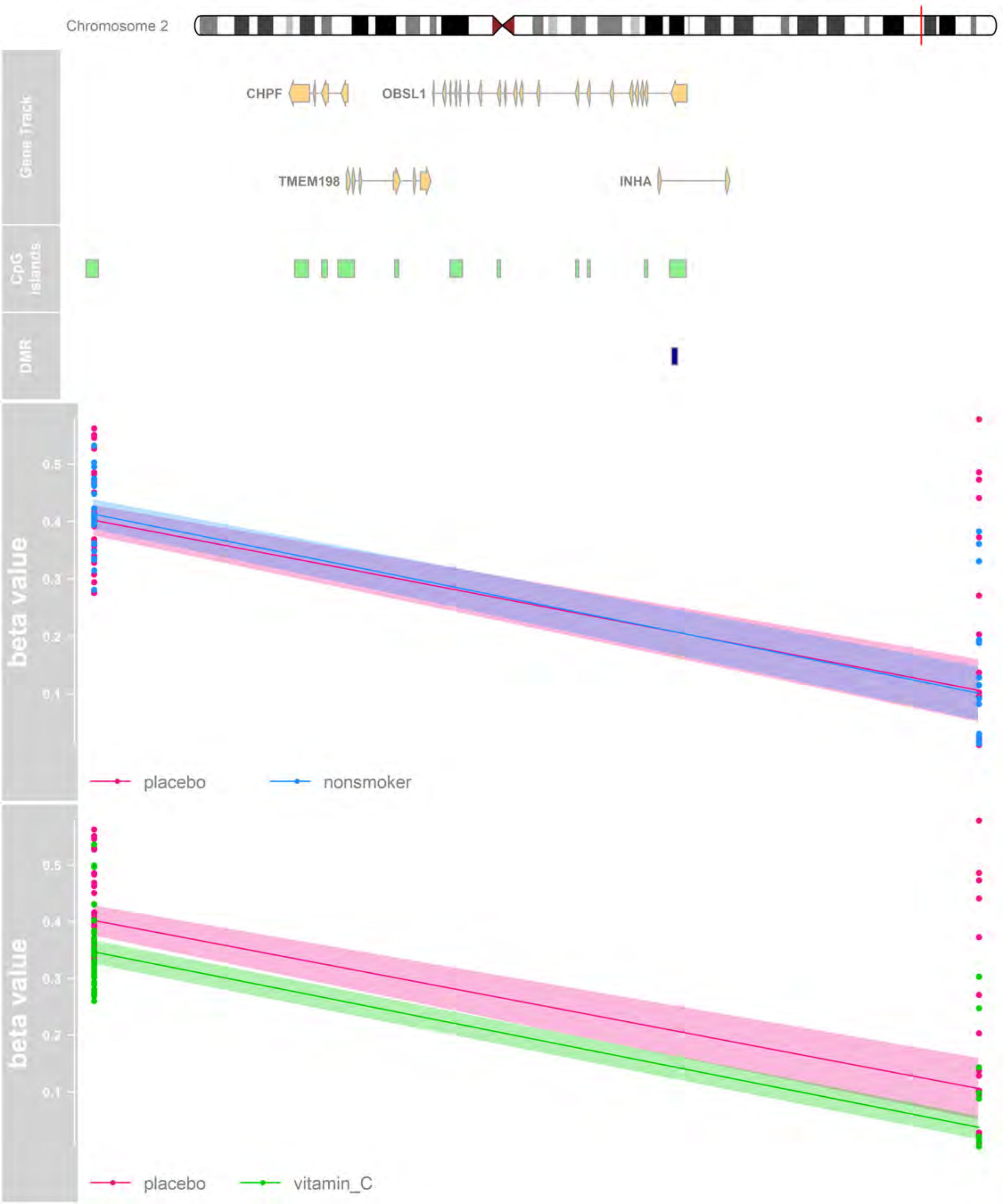

Chromosome 2
Gene Track
CHPF
OBSL1
TMEM198
INHA
CpG islands
DMR
beta value
0.5
0.4
0.3
0.2
0.1
placebo
nonsmoker
beta value
0.5
0.4
0.3
0.2
0.1
placebo
vitamin_C

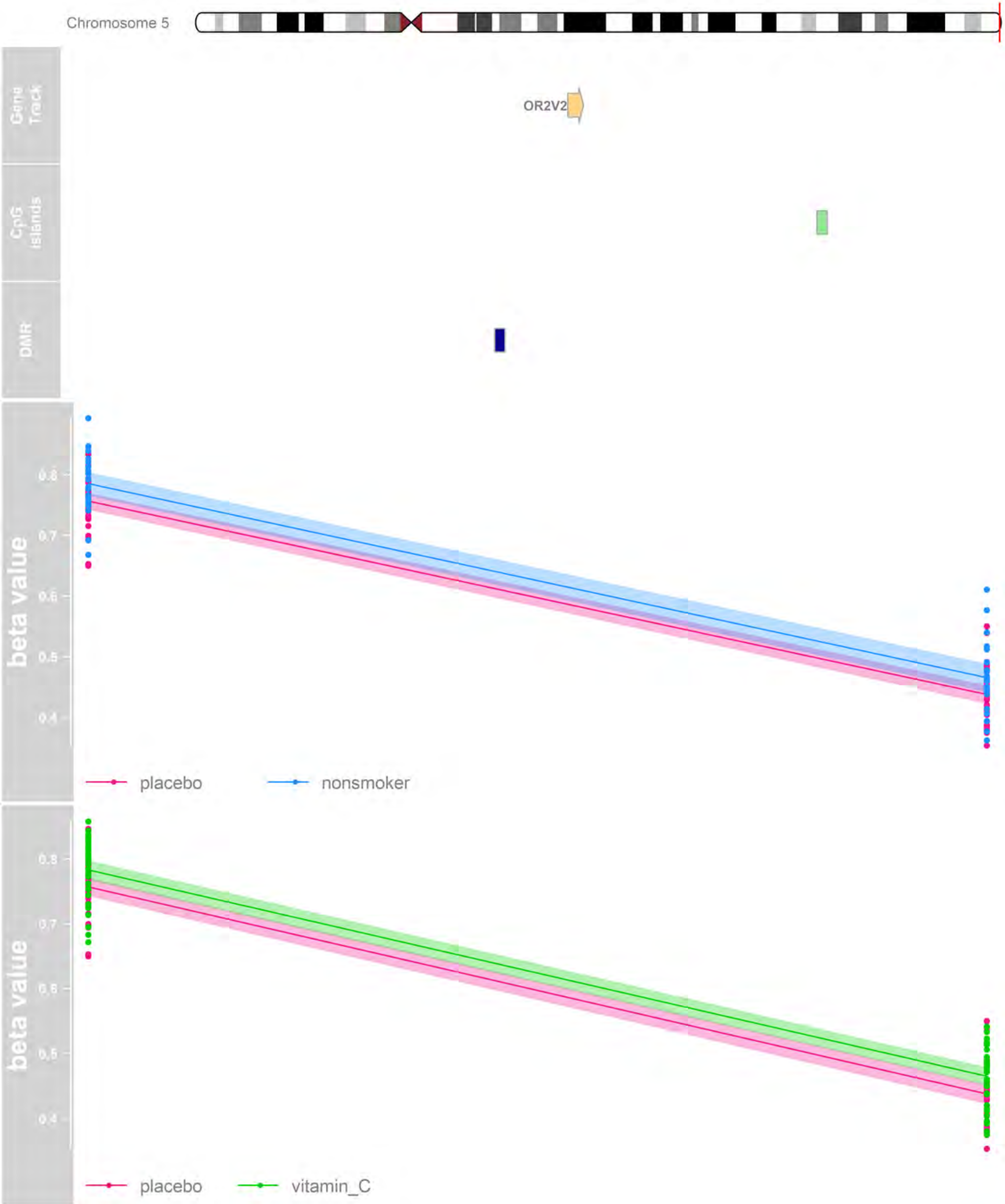

Chromosome 5
Gene Track
OR2V2
CpG islands
DMR
beta value
0.8
0.7
0.6
0.5
0.4
placebo
nonsmoker
beta value
0.8
0.7
0.6
0.5
0.4
placebo
vitamin_C

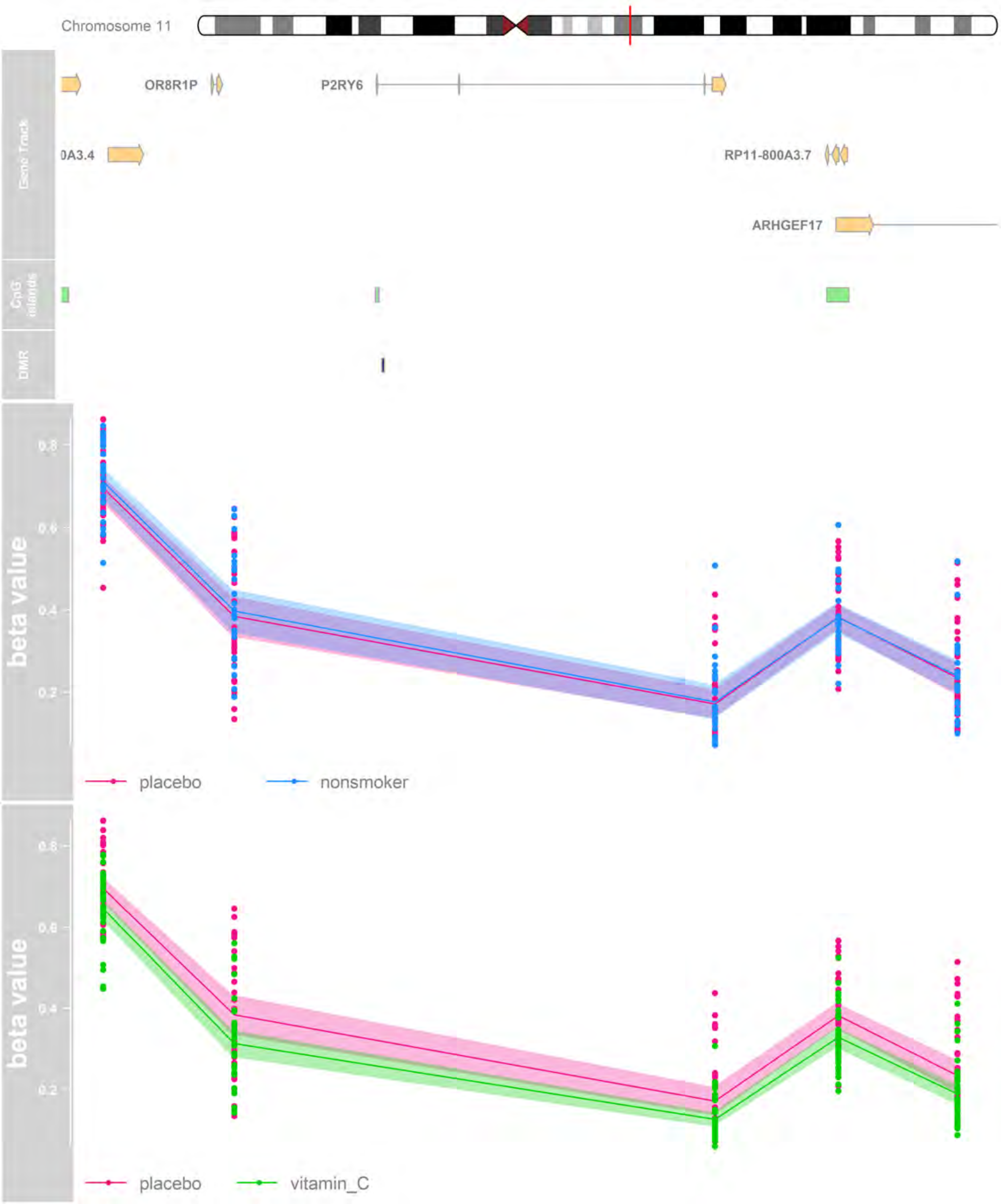

Chromosome 11
Gene Track
OR8R1P
P2RY6
0A3.4
RP11-800A3.7
ARHGEF17
CpG islands
DMR
beta value
0.8
0.6
0.4
0.2
placebo
nonsmoker
beta value
0.8
0.6
0.4
0.2
placebo
vitamin_C

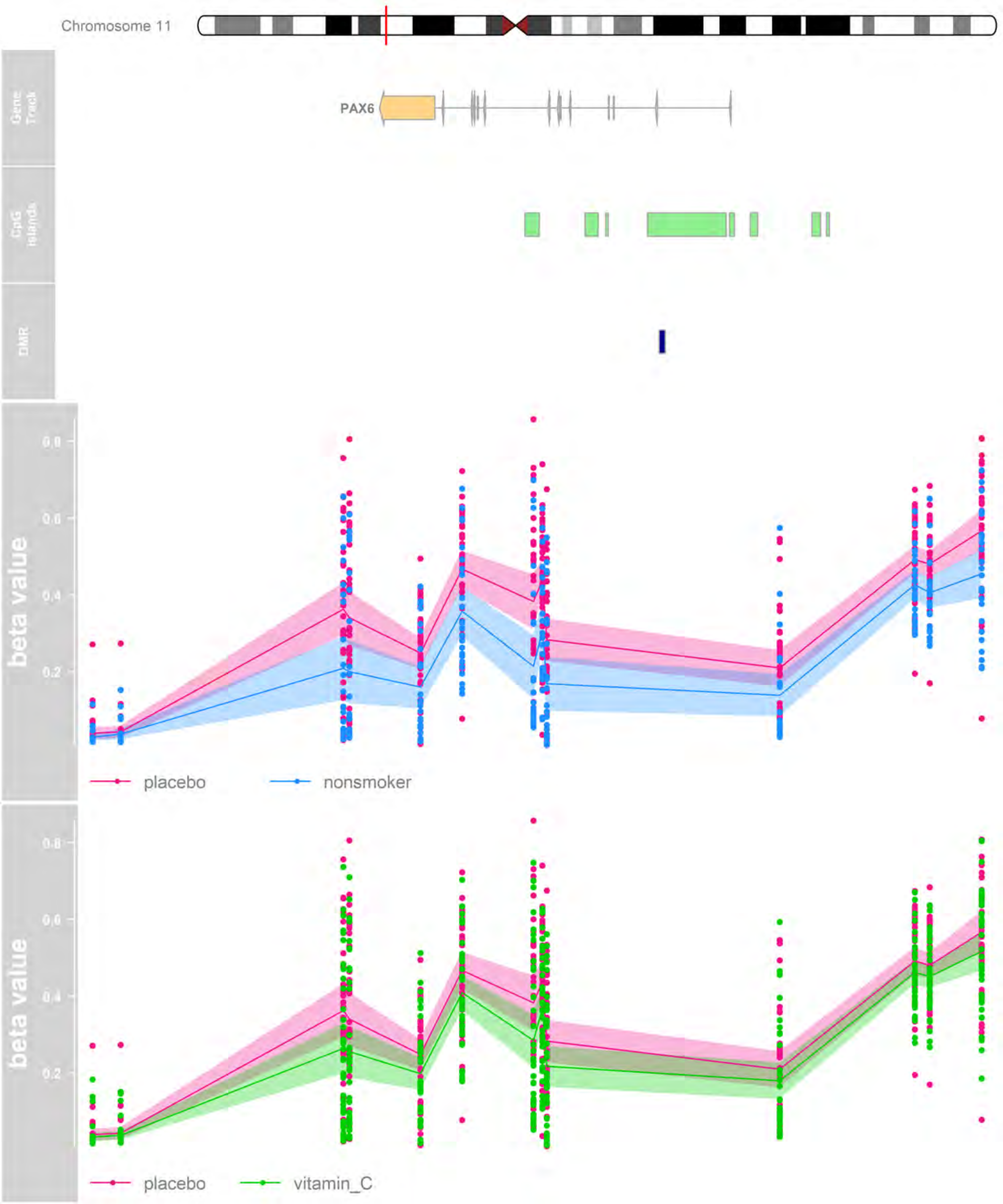

Chromosome 11
Gene Track
PAX6
CpG Islands
DMR
beta value
0.8
0.6
0.4
0.2
placebo
nonsmoker
beta value
0.8
0.6
0.4
0.2
placebo
vitamin_C

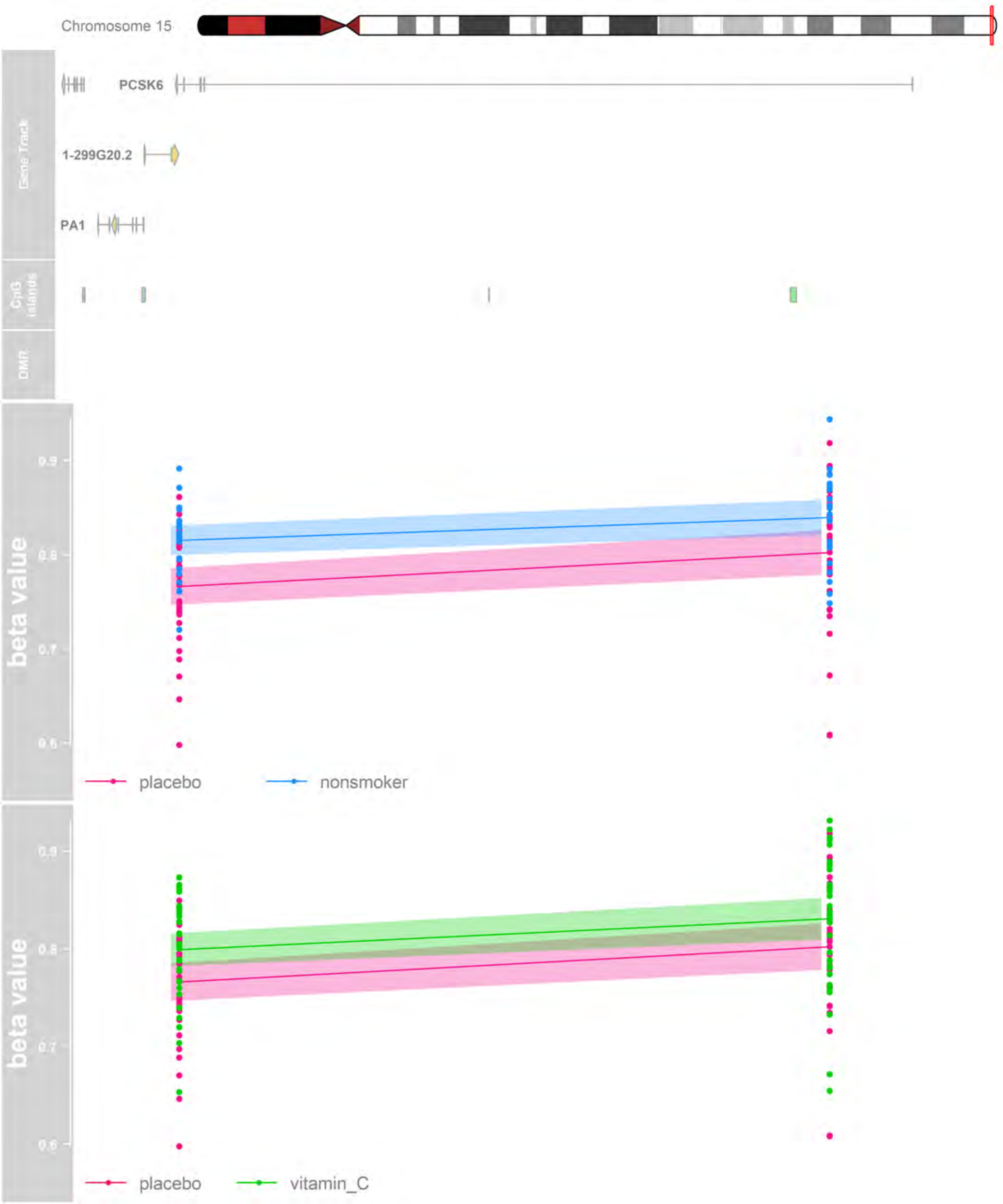

Chromosome 15
Gene Track
PCSK6
1-299G20.2
PA1
CpG islands
DMR
beta value
0.9
0.8
0.7
0.6
placebo
nonsmoker
beta value
0.9
0.8
0.7
0.6
placebo
vitamin_C

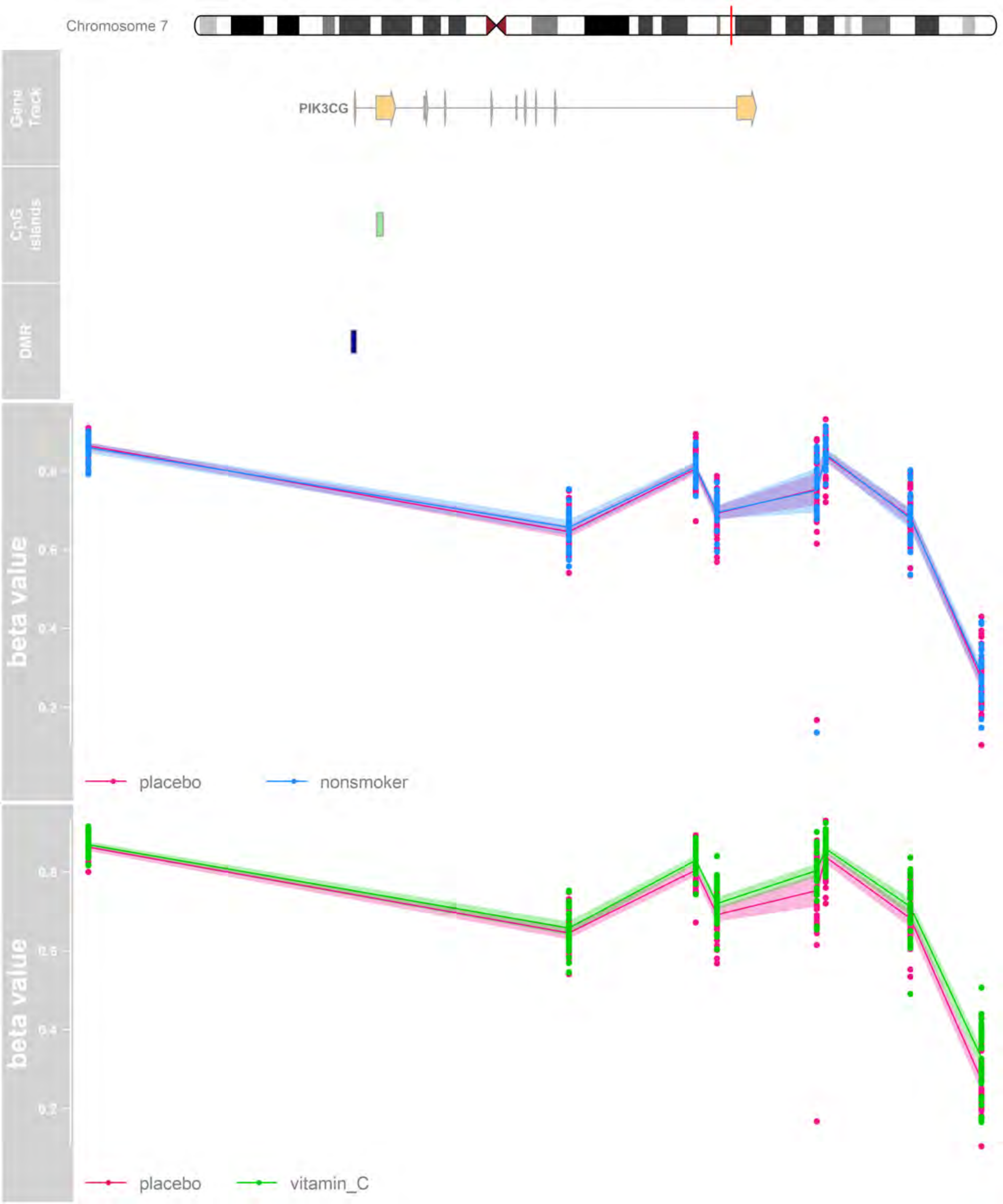

Chromosome 7
Gene Track
PIK3CG
CpG islands
DMR
beta value
0.8
0.6
0.4
0.2
placebo
nonsmoker
beta value
0.8
0.6
0.4
0.2
placebo
vitamin_C

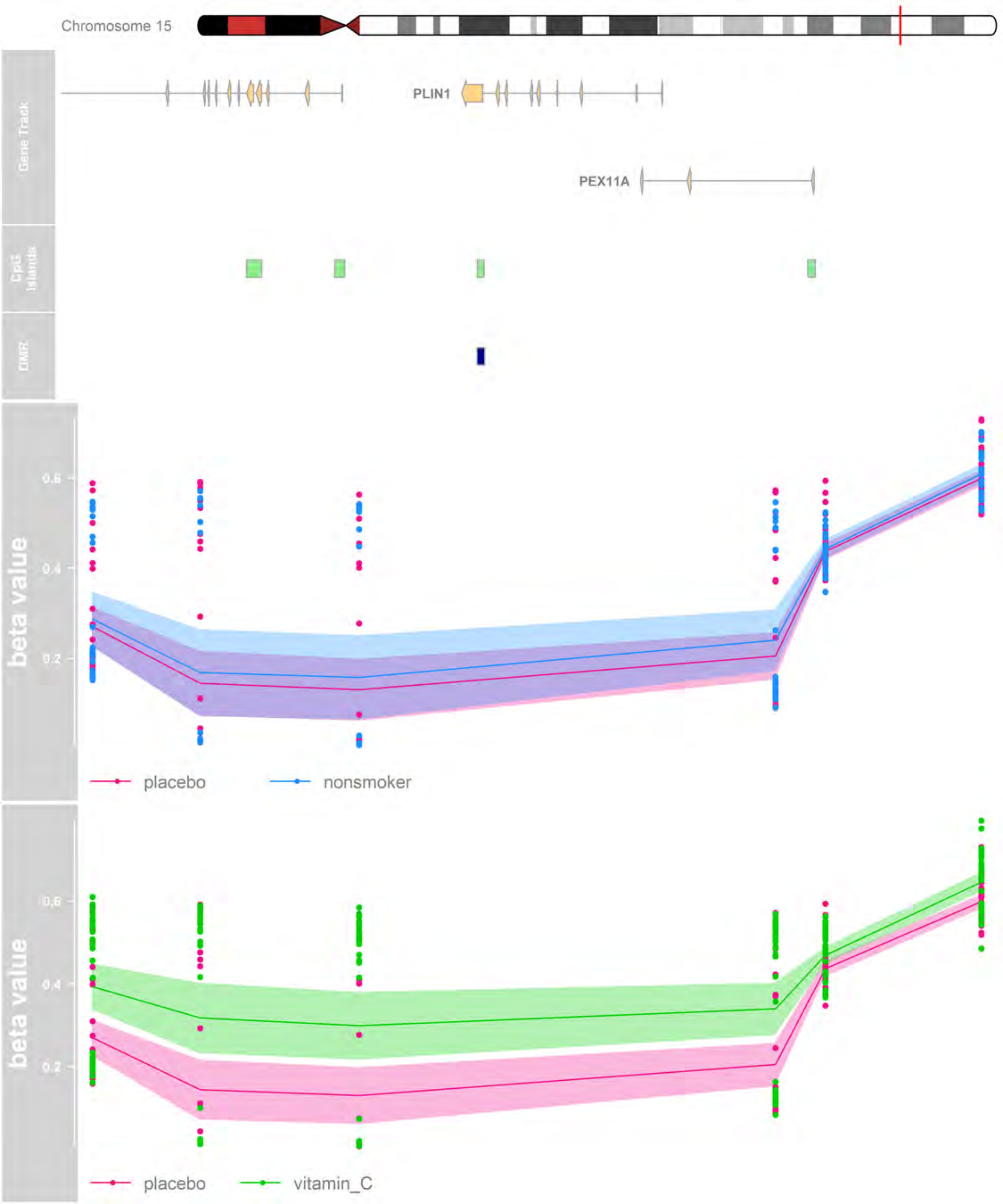

Chromosome 15
Gene Track
PLIN1
PEX11A
CpG Islands
DMR
beta value
0.6
0.4
0.2
placebo
nonsmoker
beta value
0.6
0.4
0.2
placebo
vitamin_C

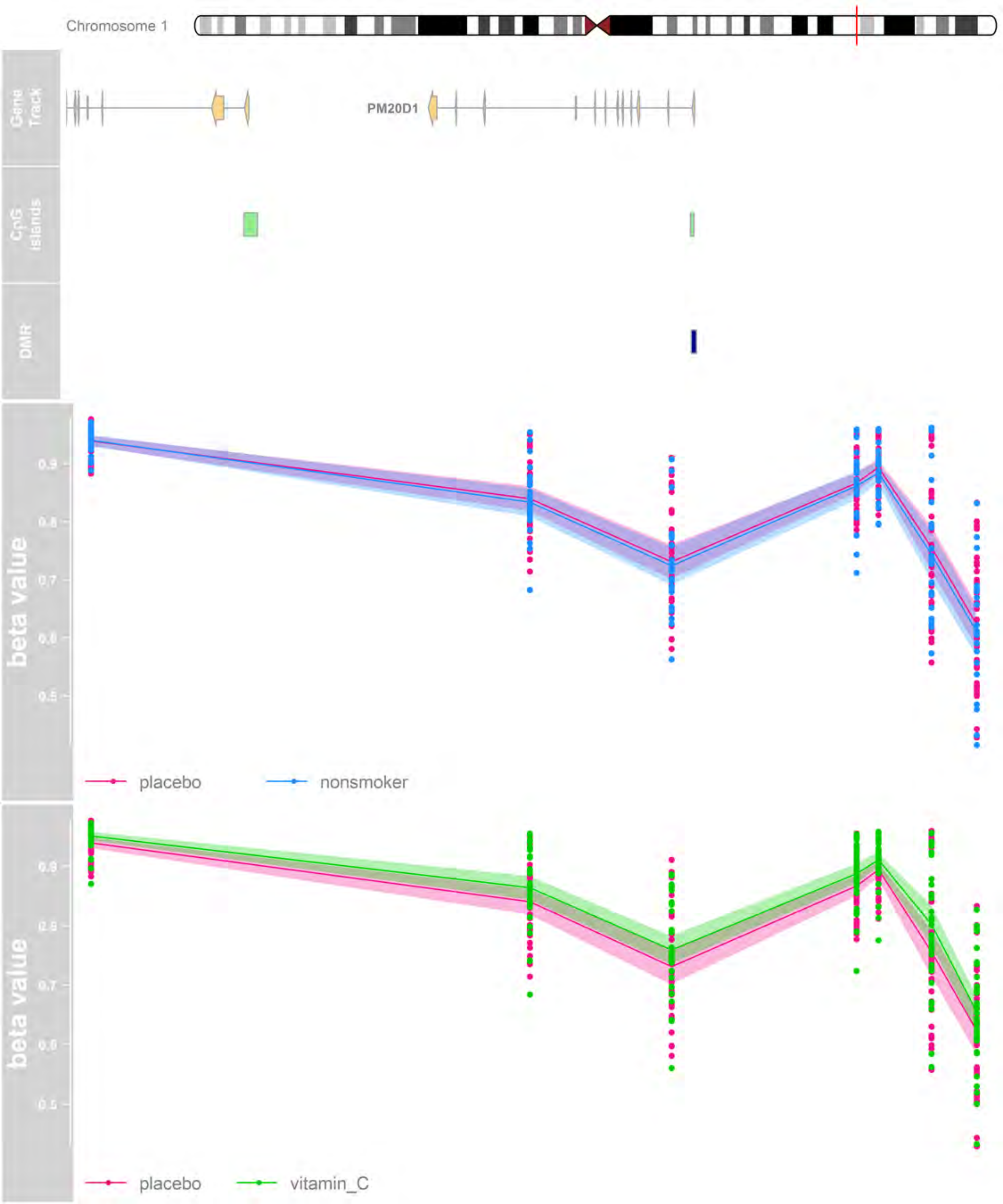

Chromosome 1
Gene Track
PM20D1
CpG Islands
DMR
beta value
0.9
0.8
0.7
0.6
0.5
placebo
nonsmoker
beta value
0.9
0.8
0.7
0.6
0.5
placebo
vitamin_C

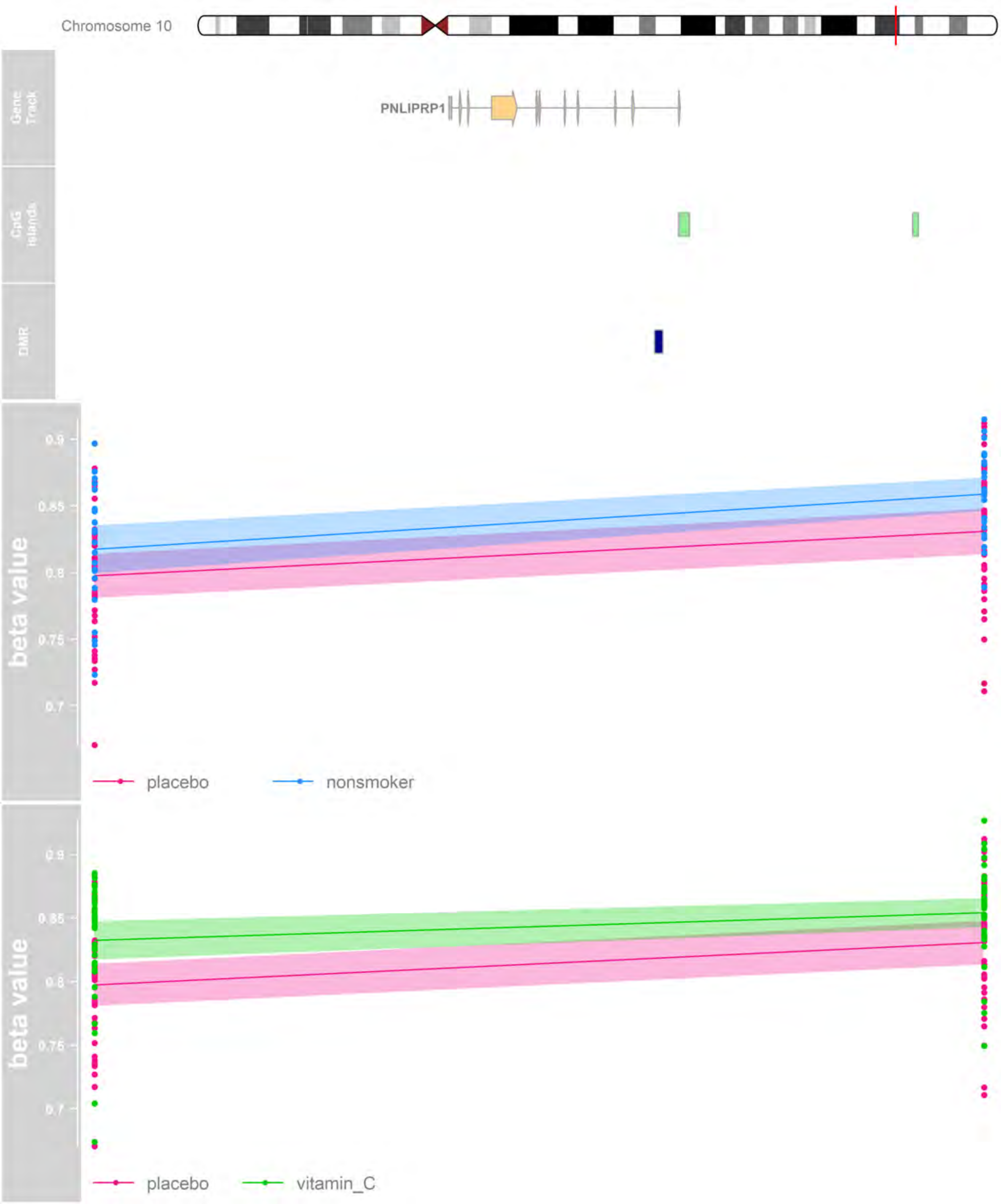

Chromosome 10
Gene Track
PNLIPRP1
CpG islands
DMR
beta value
0.9
0.85
0.8
0.75
0.7
placebo
nonsmoker
beta value
0.9
0.85
0.8
0.75
0.7
placebo
vitamin_C

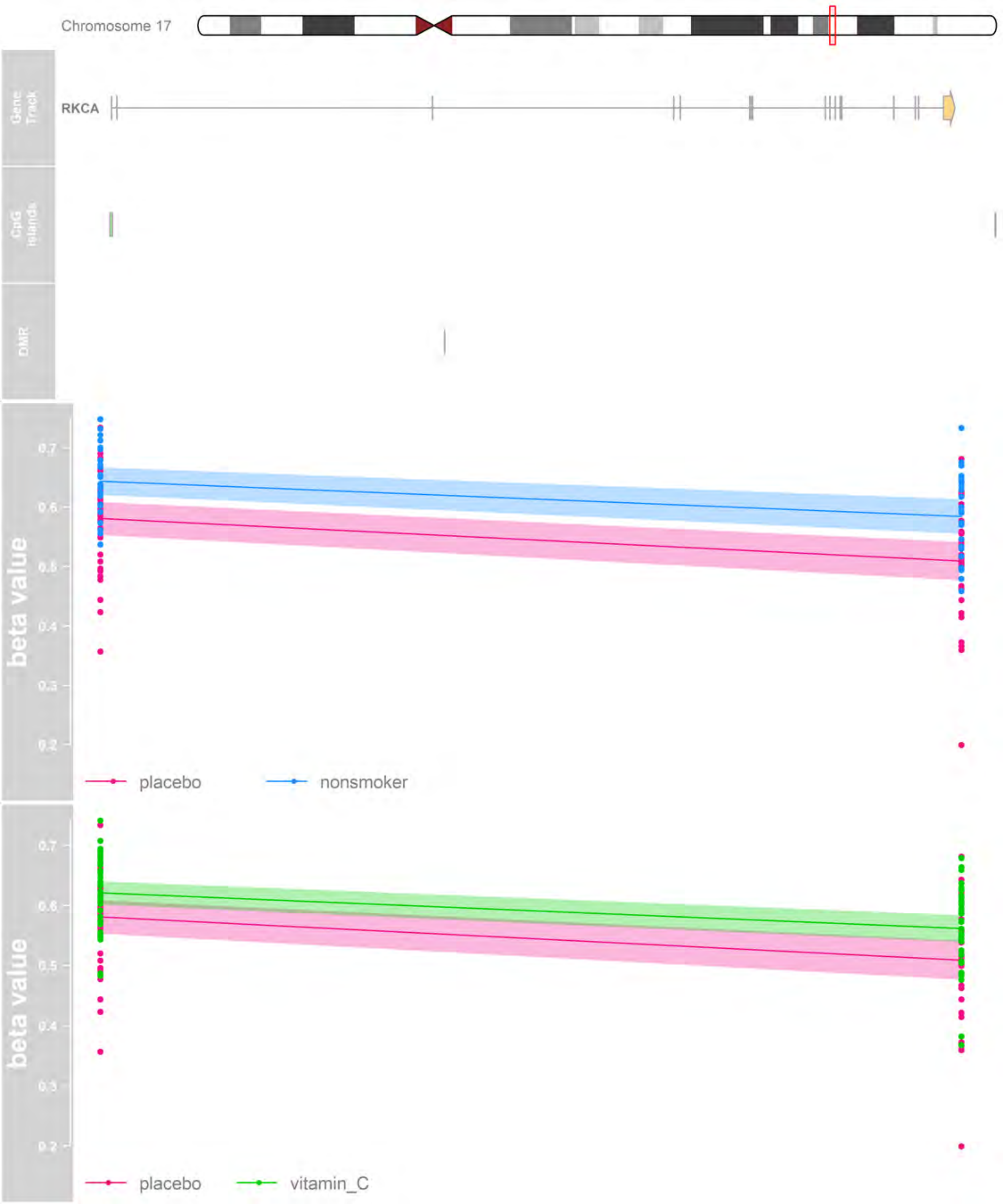
Chromosome 17
Gene Track
RKCA
CpG islands
DMR
beta value
0.7
0.6
0.5
0.4
0.3
0.2
placebo
nonsmoker
beta value
0.7
0.6
0.5
0.4
0.3
0.2
placebo
vitamin_C

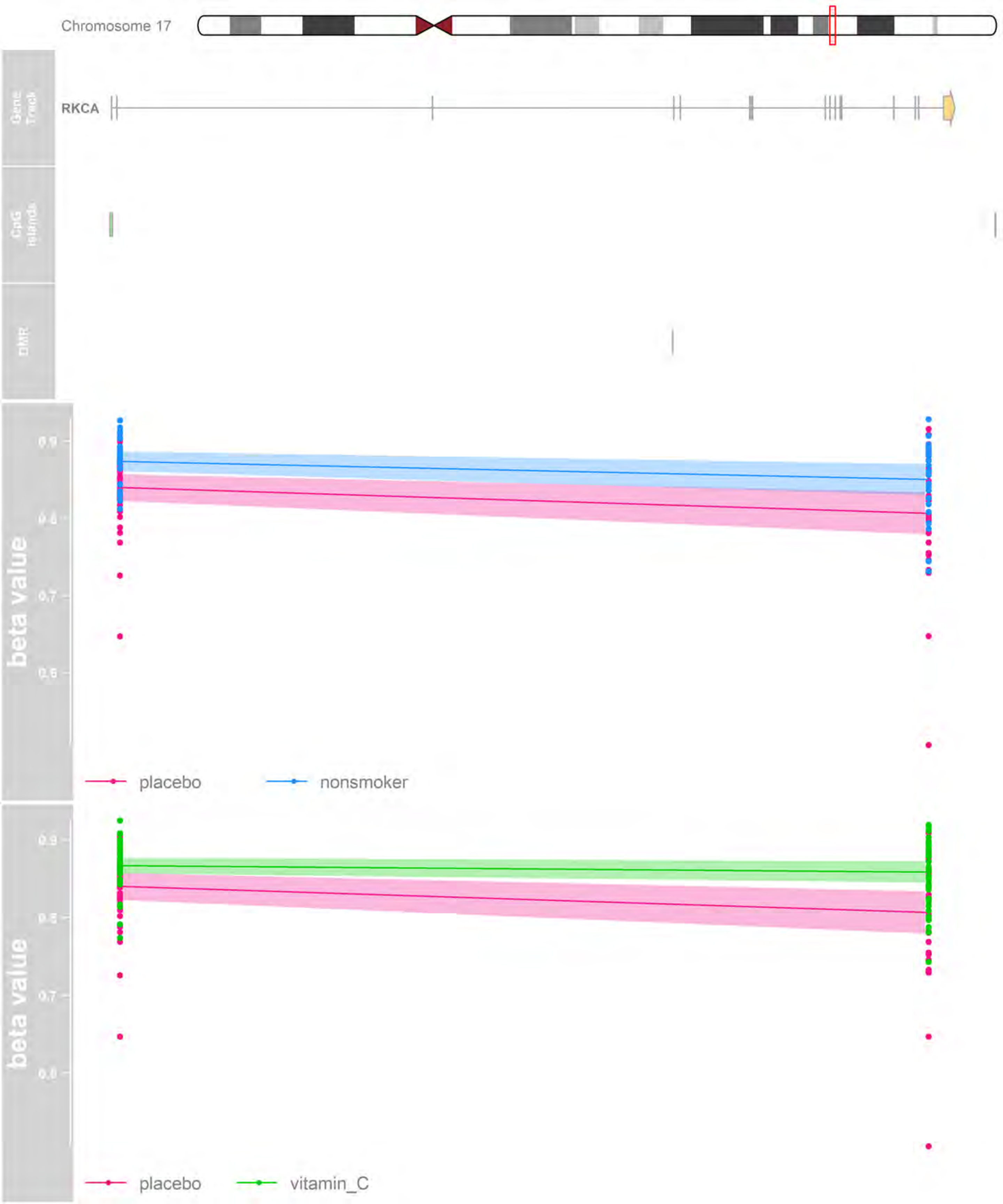

Chromosome 17
Gene Track
RKCA
CpG islands
DMR
beta value
0.9
0.8
0.7
0.6
placebo
nonsmoker
beta value
0.9
0.8
0.7
0.6
placebo
vitamin_C

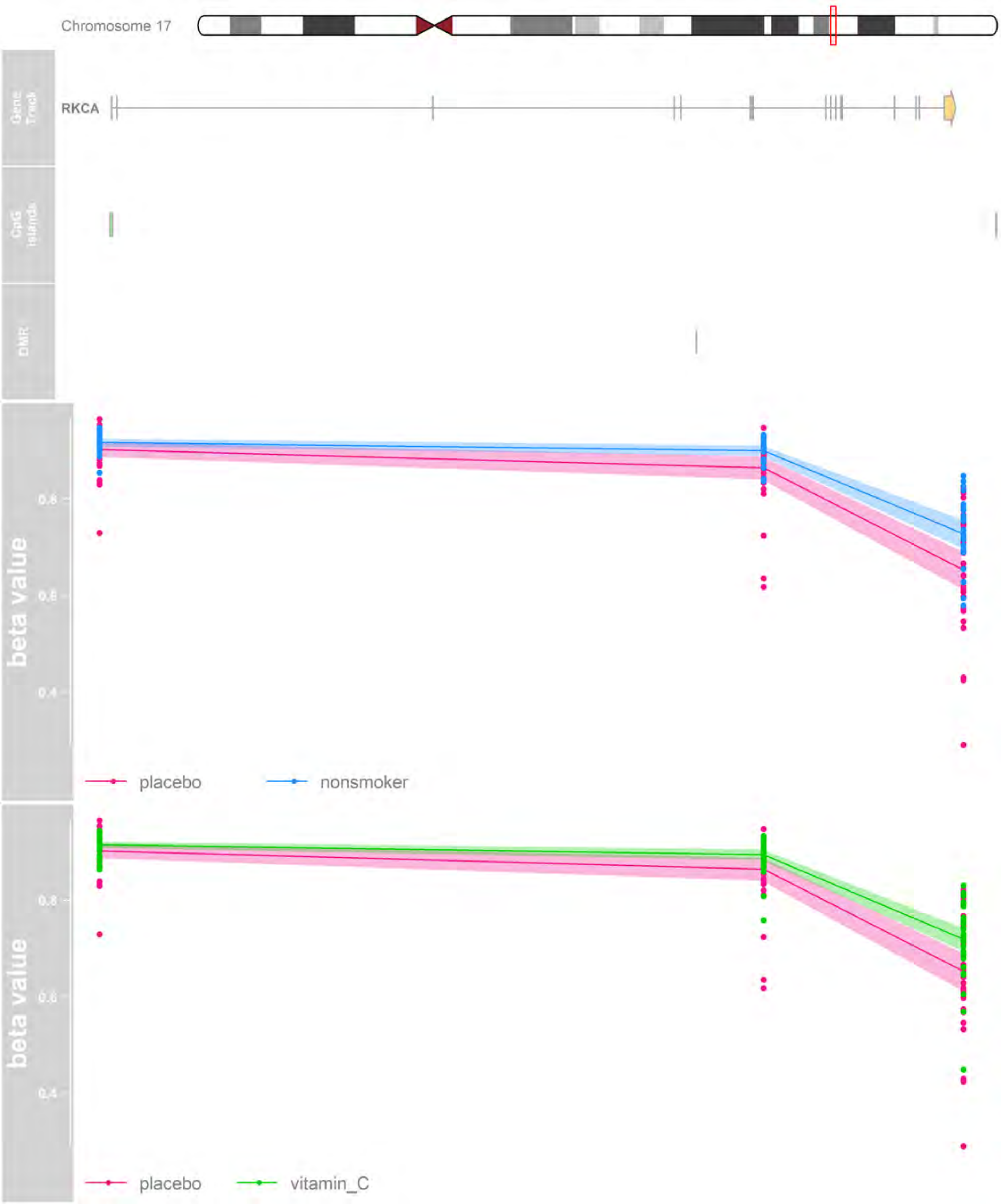

Chromosome 17
Gene Track
RKCA
CpG islands
DMR
beta value
0.8
0.6
0.4
placebo
nonsmoker
beta value
0.8
0.6
0.4
placebo
vitamin_C

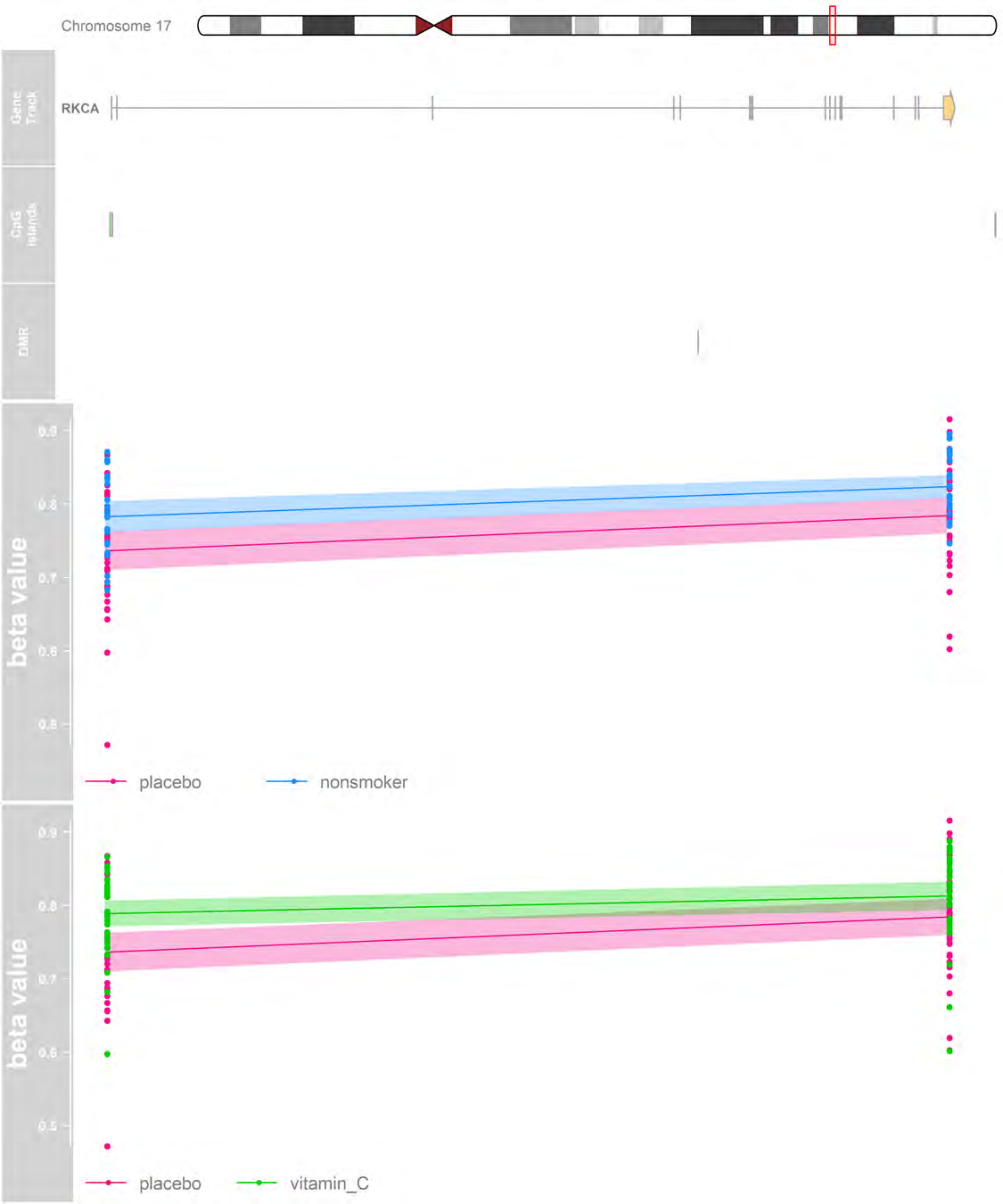

Chromosome 17
Gene Track
RKCA
CpG islands
DMR
beta value
placebo
nonsmoker
beta value
placebo
vitamin_C

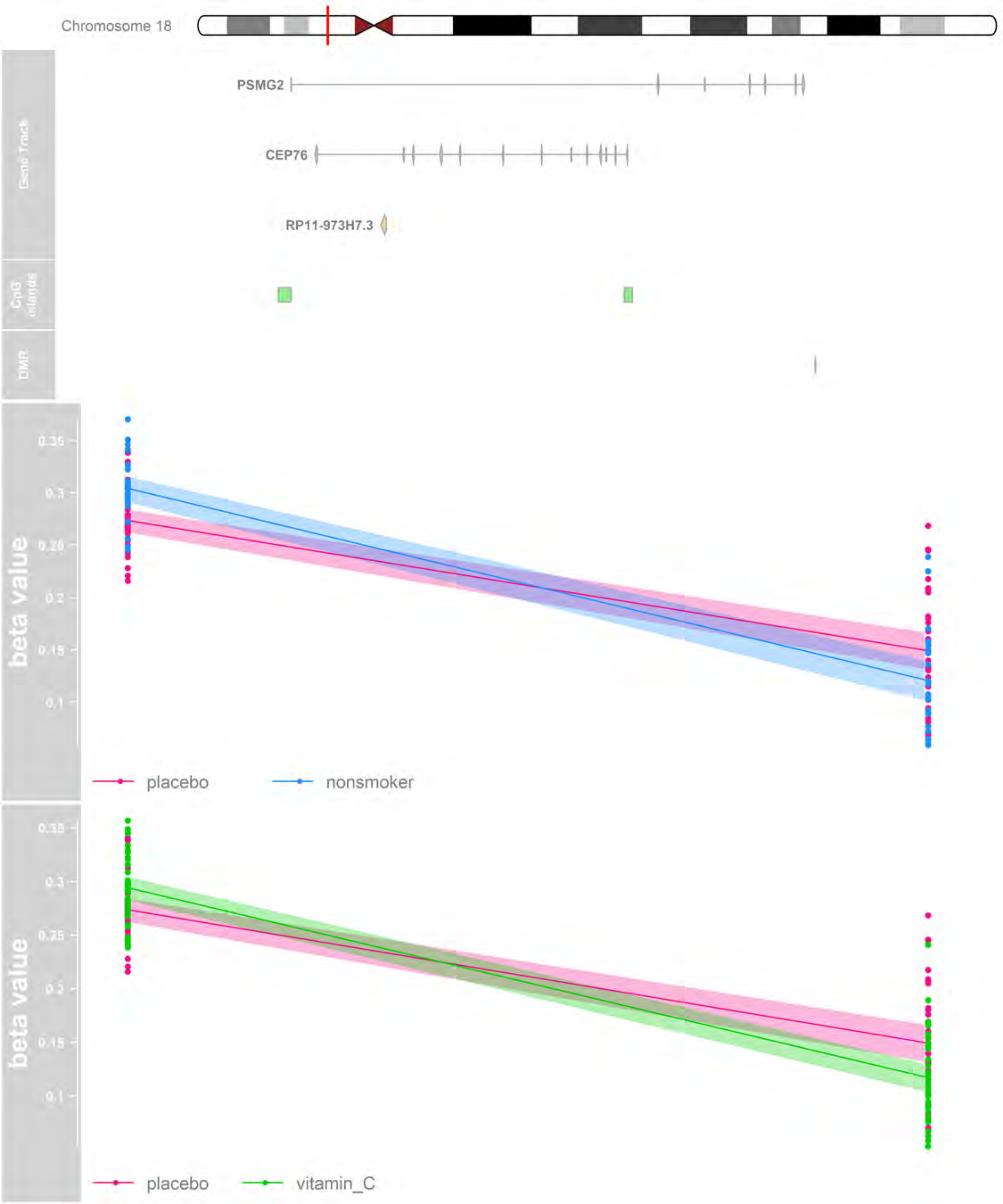

Chromosome 18
PSMG2
CEP76
RP11-973H7.3
Gene Track
CpG islands
DMR
beta value
0.35
0.3
0.25
0.2
0.15
0.1
placebo
nonsmoker
beta value
0.35
0.3
0.25
0.2
0.15
0.1
placebo
vitamin_C

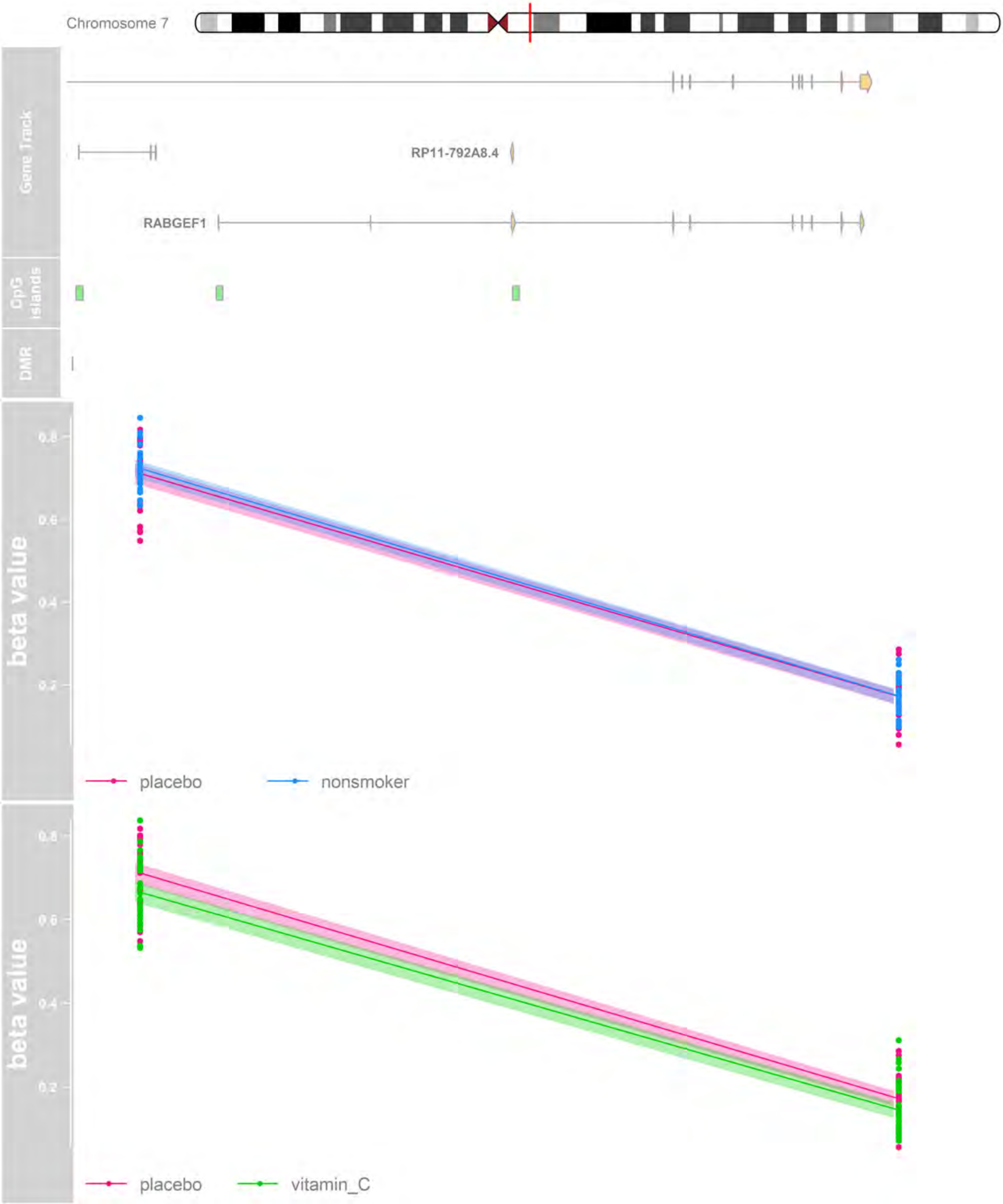

Chromosome 7
Gene Track
RP11-792A8.4
RABGEF1
CpG islands
DMR
beta value
0.8
0.6
0.4
0.2
placebo
nonsmoker
beta value
0.8
0.6
0.4
0.2
placebo
vitamin_C

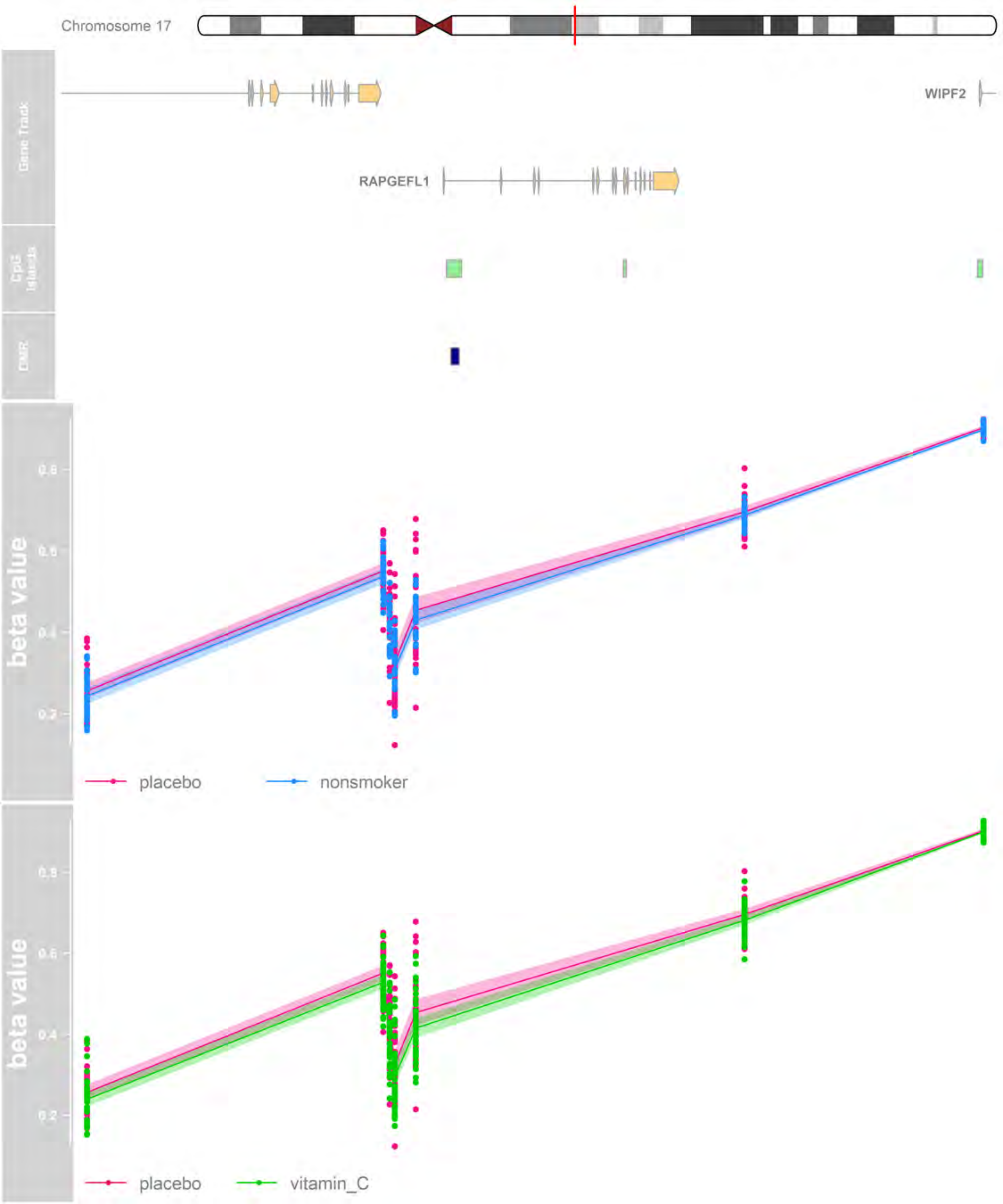

Chromosome 17
Gene Track
WIPF2
RAPGEFL1
CpG Islands
DMR
beta value
0.8
0.6
0.4
0.2
placebo
nonsmoker
beta value
0.8
0.6
0.4
0.2
placebo
vitamin_C

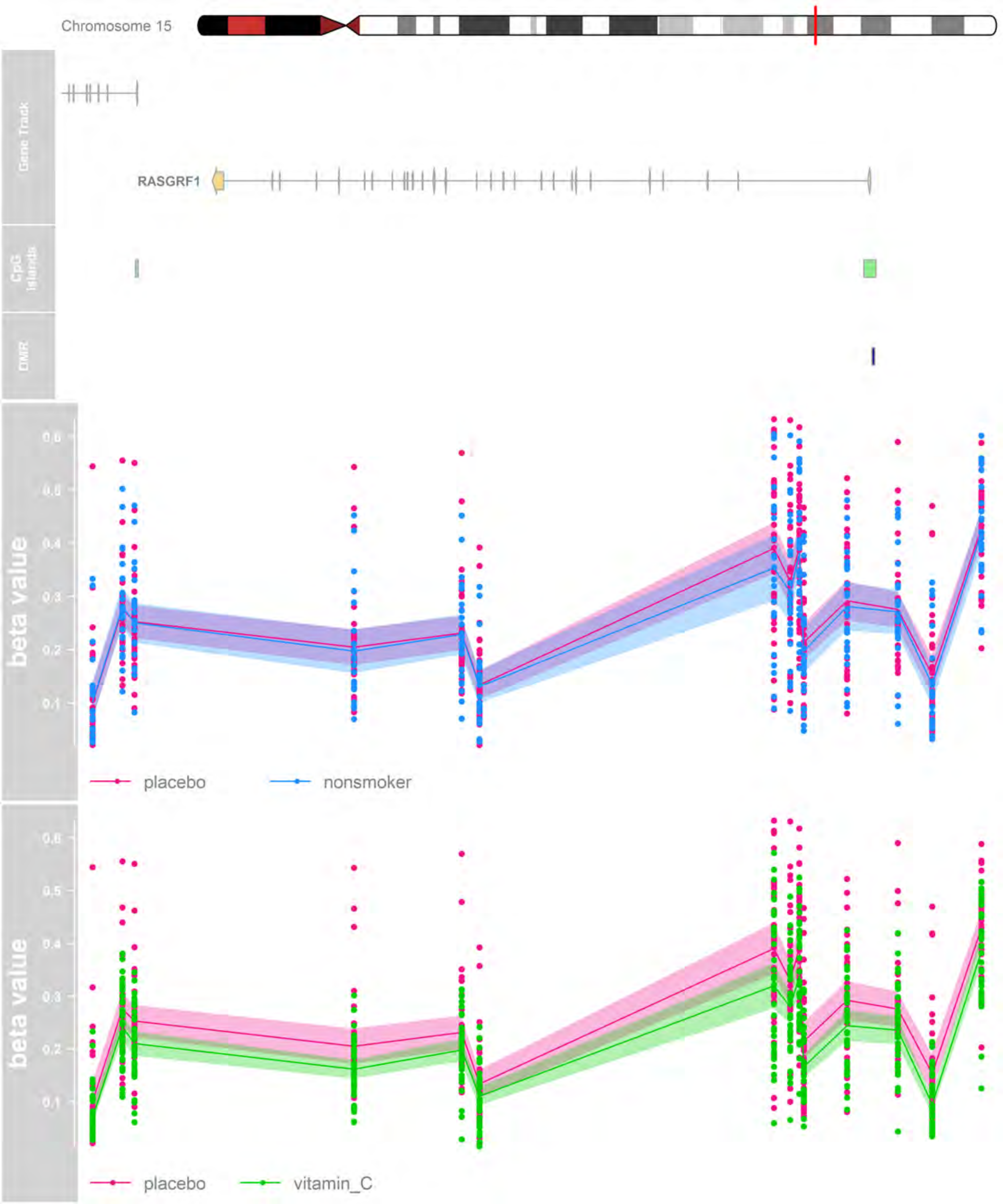

Chromosome 15
Gene Track
RASGRF1
CpG Islands
DMR
beta value
0.6
0.5
0.4
0.3
0.2
0.1
placebo
nonsmoker
beta value
0.6
0.5
0.4
0.3
0.2
0.1
placebo
vitamin_C

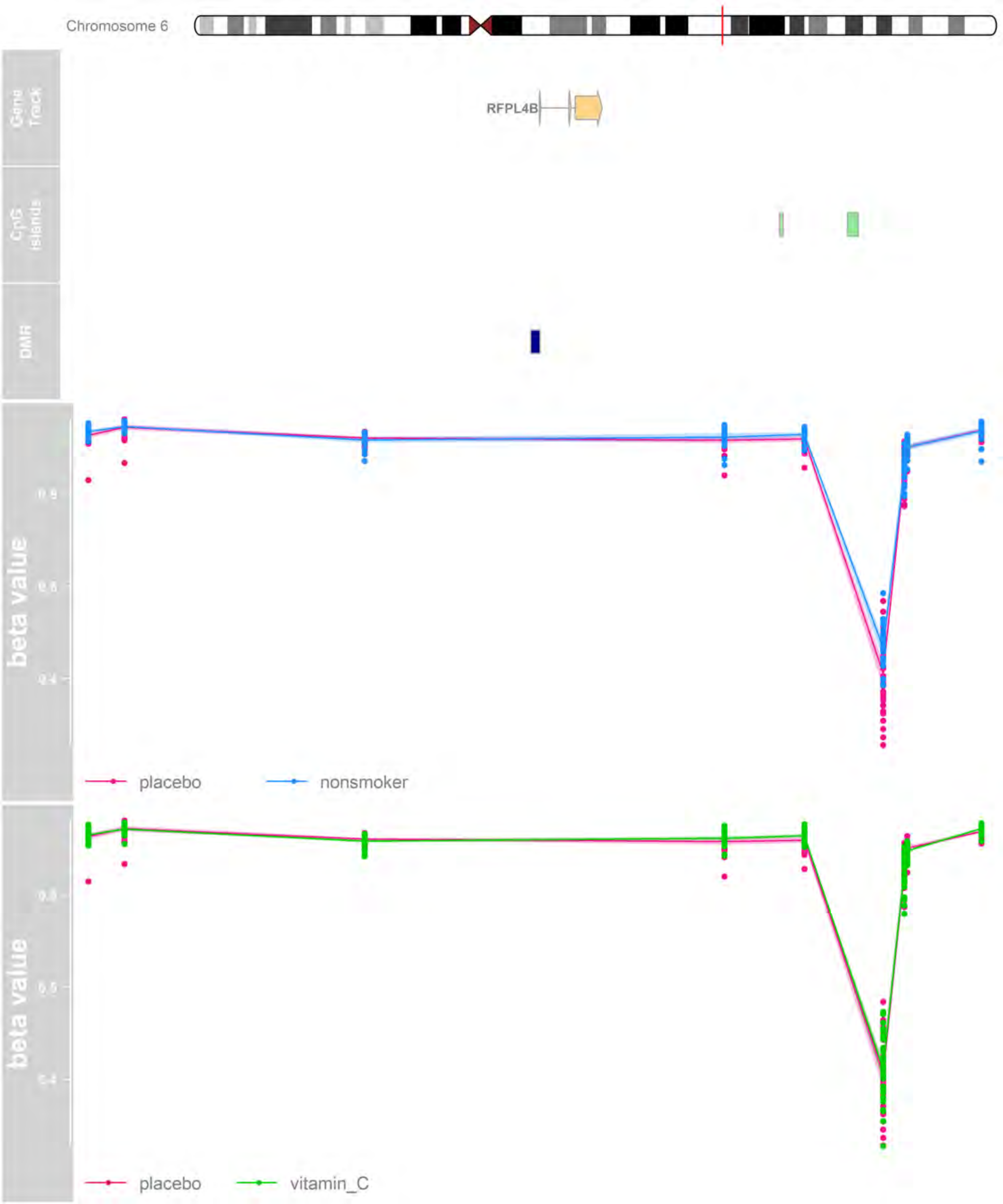

Chromosome 6
Gene Track
RFPL4B
CpG islands
DMR
beta value
placebo
nonsmoker
beta value
placebo
vitamin_C

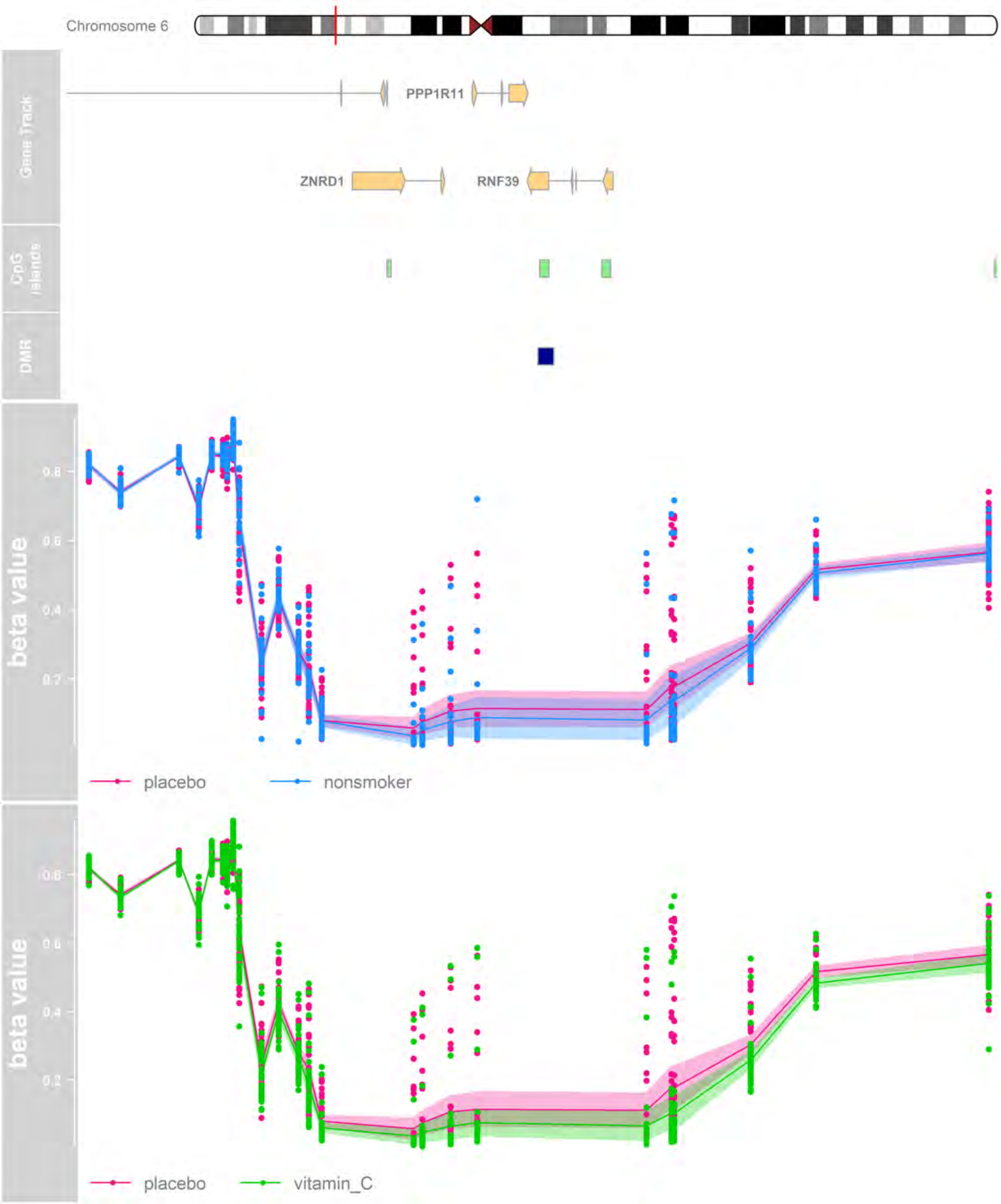

Chromosome 6
Gene Track
PPP1R11
ZNRD1
RNF39
CpG islands
DMR
beta value
0.8
0.6
0.4
0.2
placebo
nonsmoker
beta value
0.8
0.6
0.4
0.2
placebo
vitamin_C

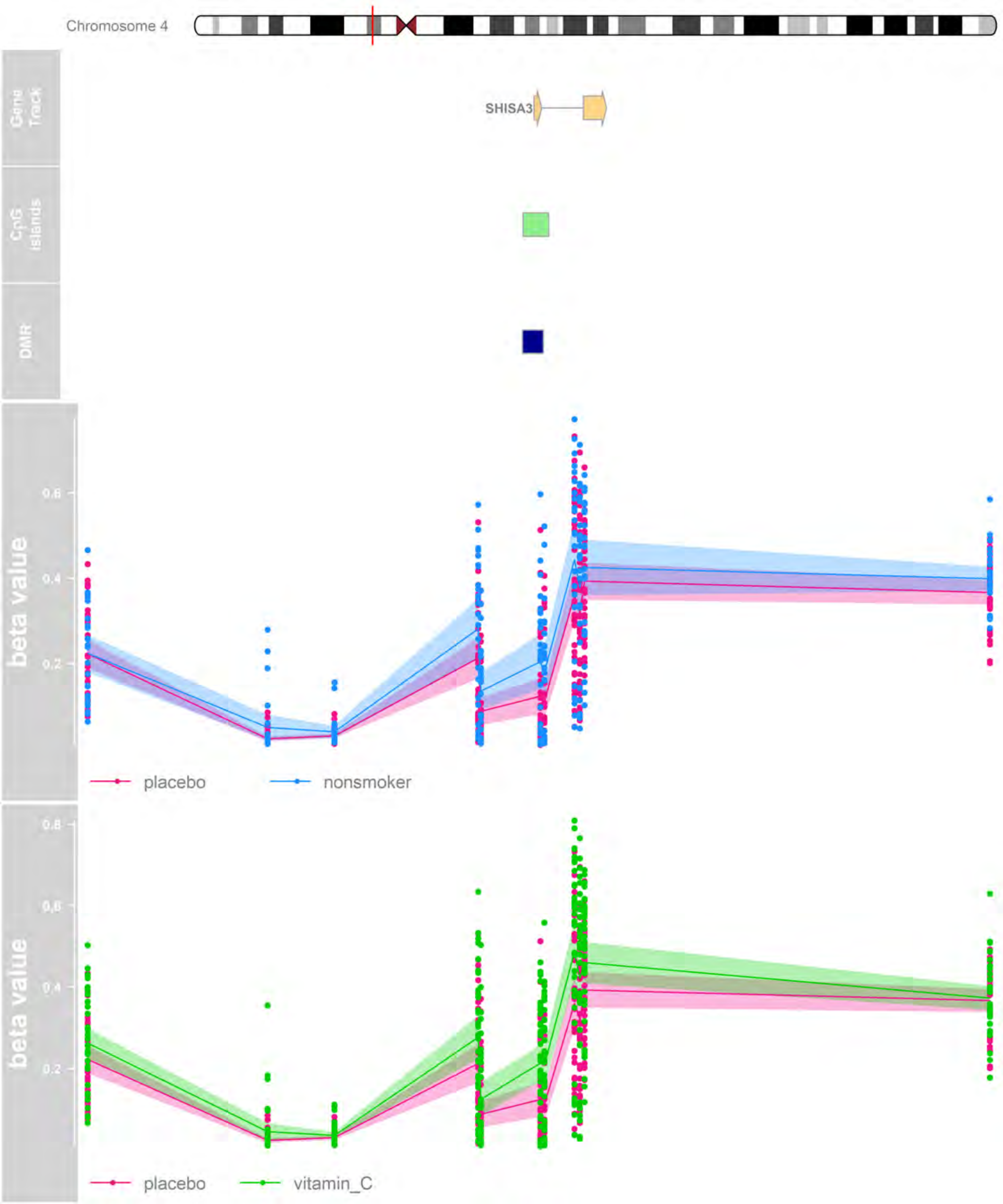

Chromosome 4
Gene Track
SHISA3
CpG islands
DMR
beta value
0.6
0.4
0.2
placebo
nonsmoker
beta value
0.8
0.6
0.4
0.2
placebo
vitamin_C

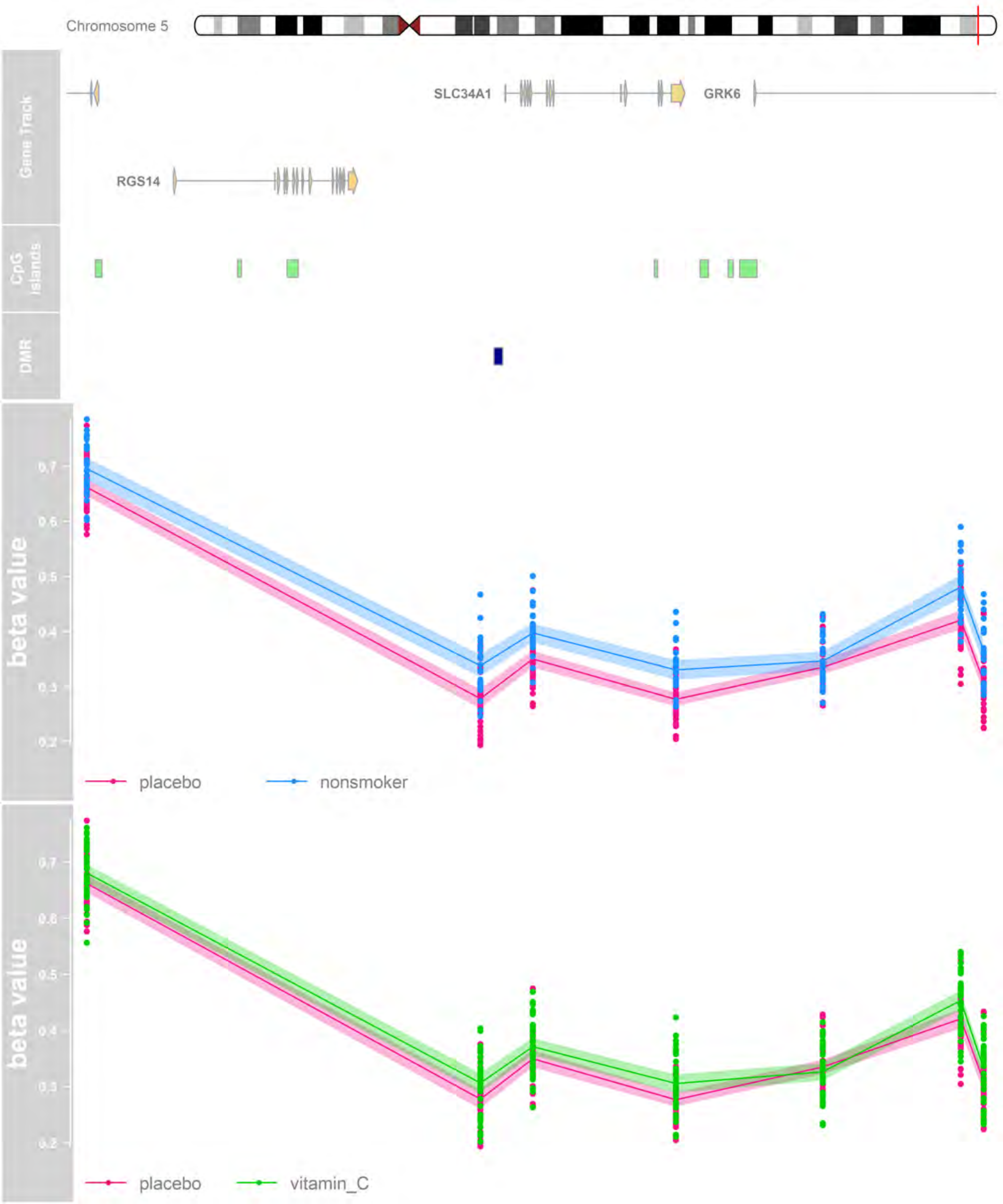

Chromosome 5
Gene Track
SLC34A1
GRK6
RGS14
CpG Islands
DMR
beta value
0.7
0.6
0.5
0.4
0.3
0.2
placebo
nonsmoker
beta value
0.7
0.6
0.5
0.4
0.3
0.2
placebo
vitamin_C

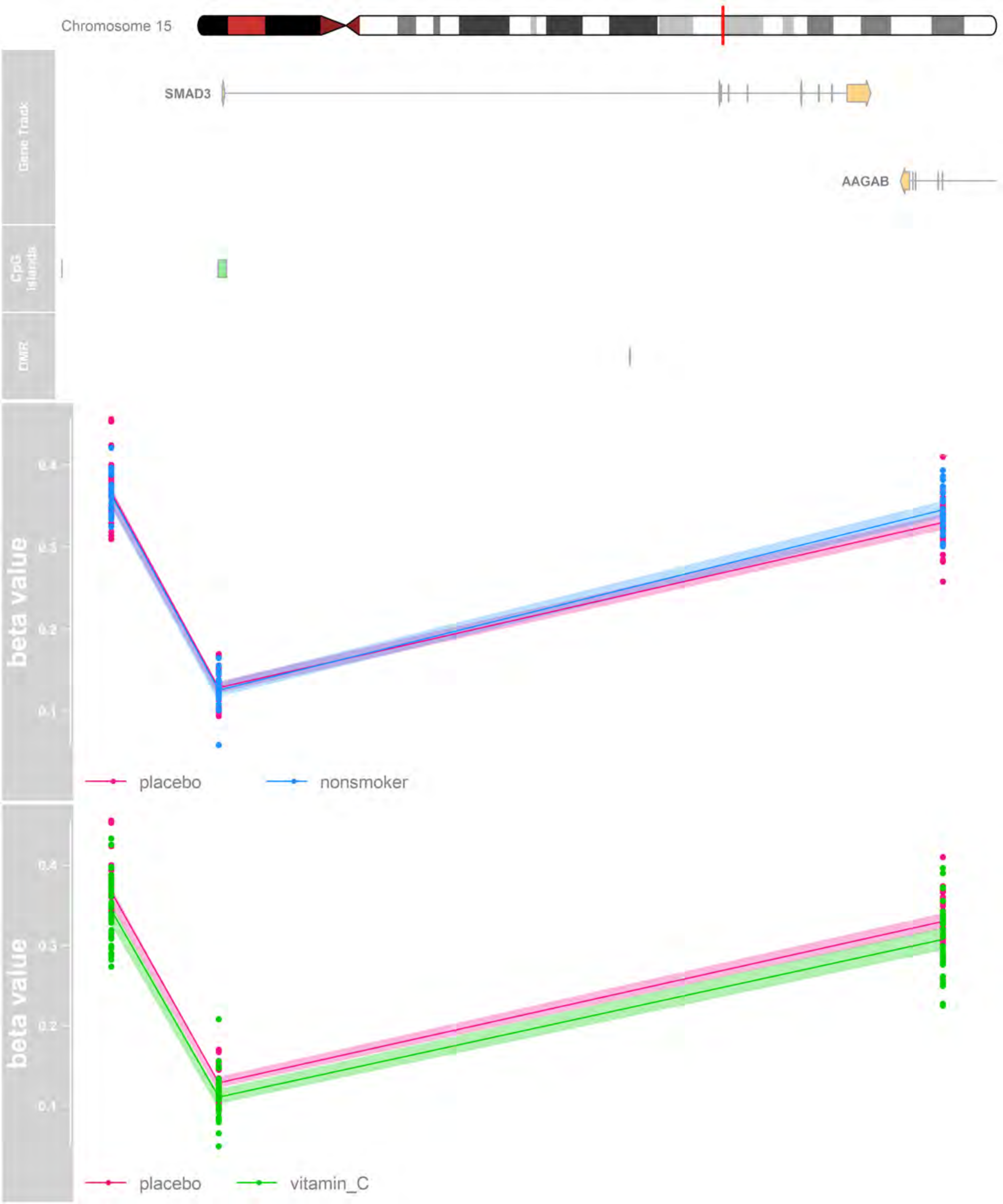

Chromosome 15
Gene Track
SMAD3
AAGAB
CpG Islands
DMR
beta value
0.4
0.3
0.2
0.1
placebo
nonsmoker
beta value
0.4
0.3
0.2
0.1
placebo
vitamin_C

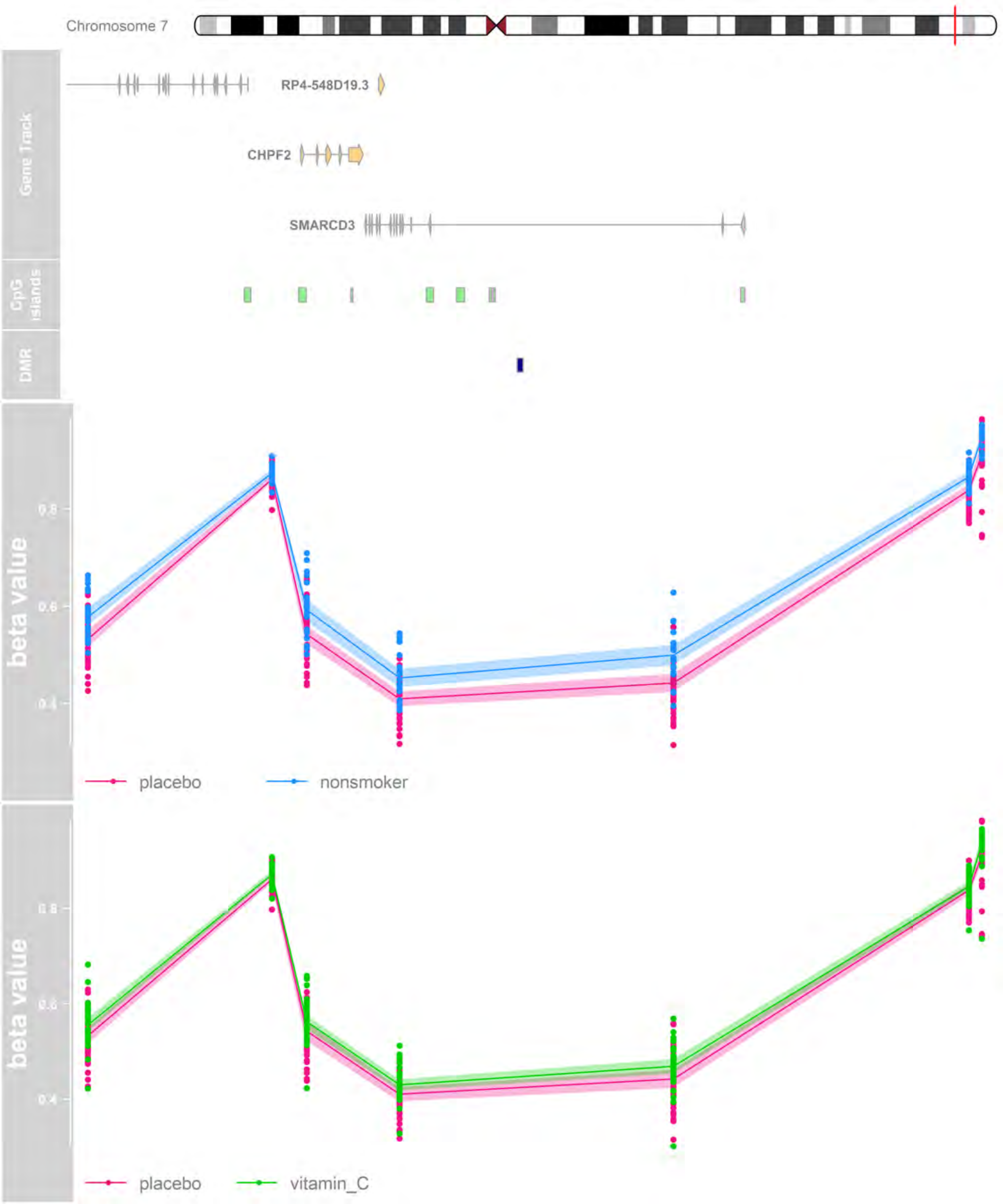

Chromosome 7
Gene Track
RP4-548D19.3
CHPF2
SMARCD3
CpG islands
DMR
beta value
0.8
0.6
0.4
placebo
nonsmoker
beta value
0.8
0.6
0.4
placebo
vitamin_C

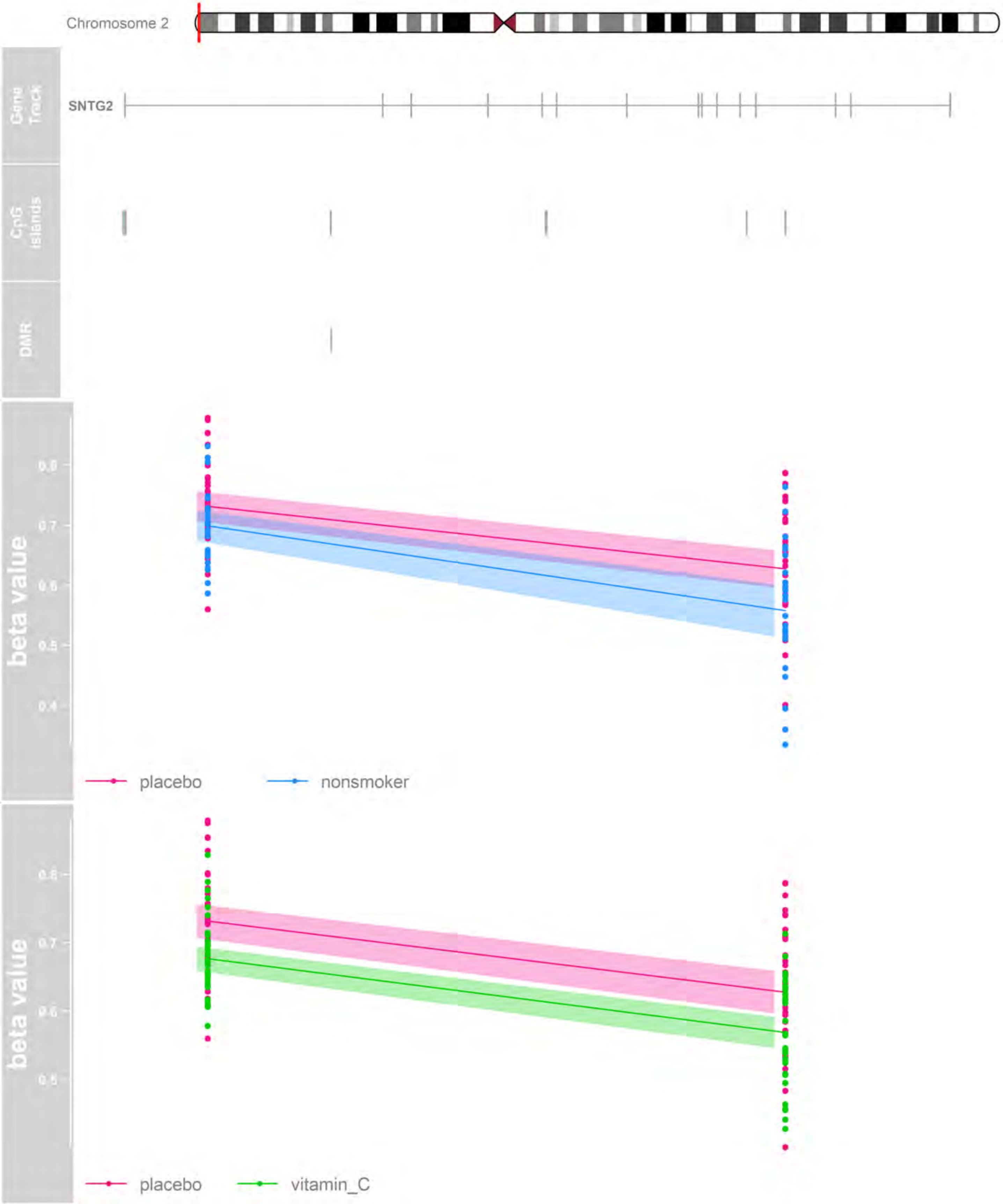

Chromosome 2
Gene Track
SNTG2
CpG Islands
DMR
beta value
0.8
0.7
0.6
0.5
0.4
placebo
nonsmoker
beta value
0.8
0.7
0.6
0.5
placebo
vitamin_C

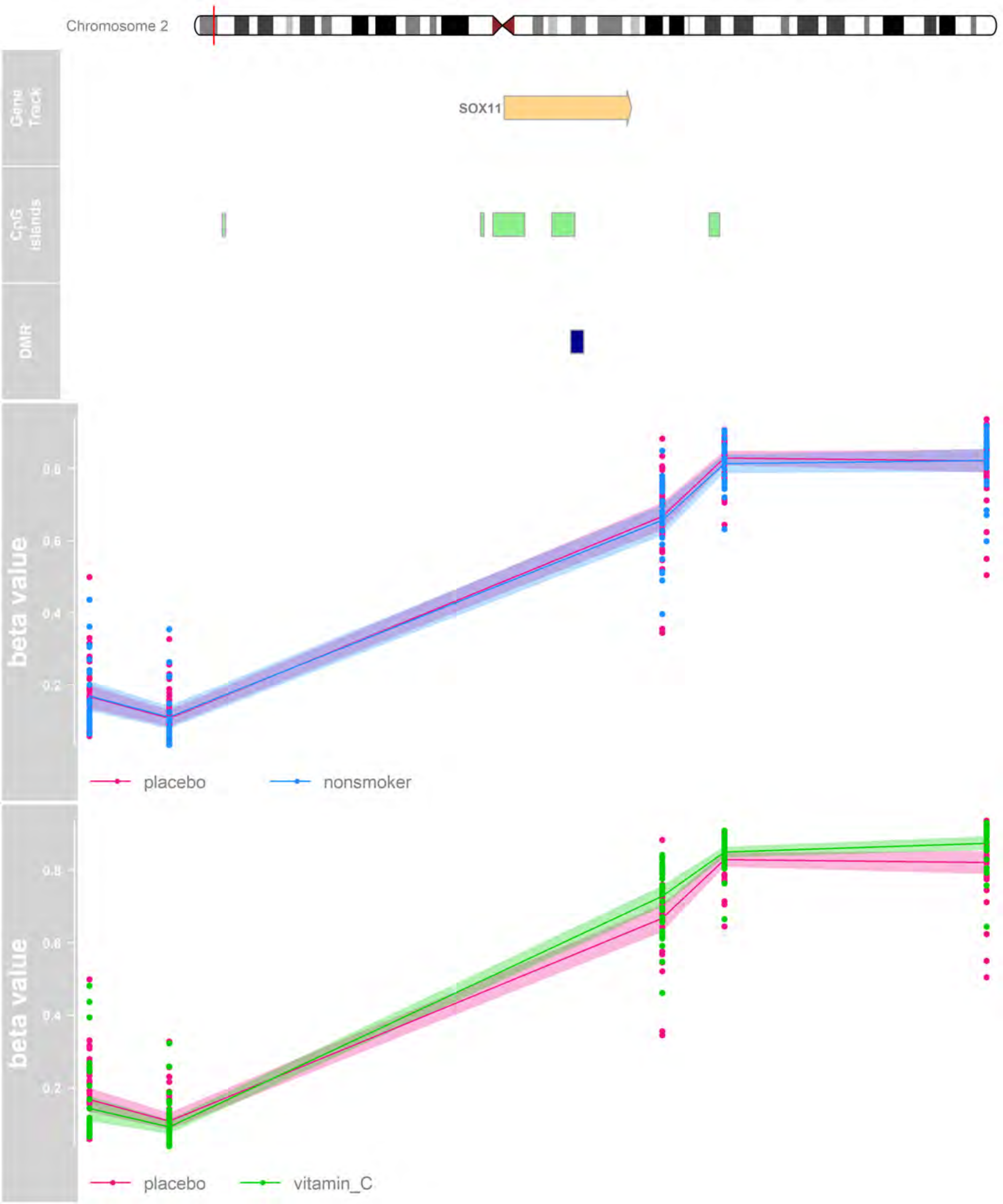

Chromosome 2
Gene Track
SOX11
CpG islands
DMR
beta value
0.8
0.6
0.4
0.2
placebo
nonsmoker
beta value
0.8
0.6
0.4
0.2
placebo
vitamin_C

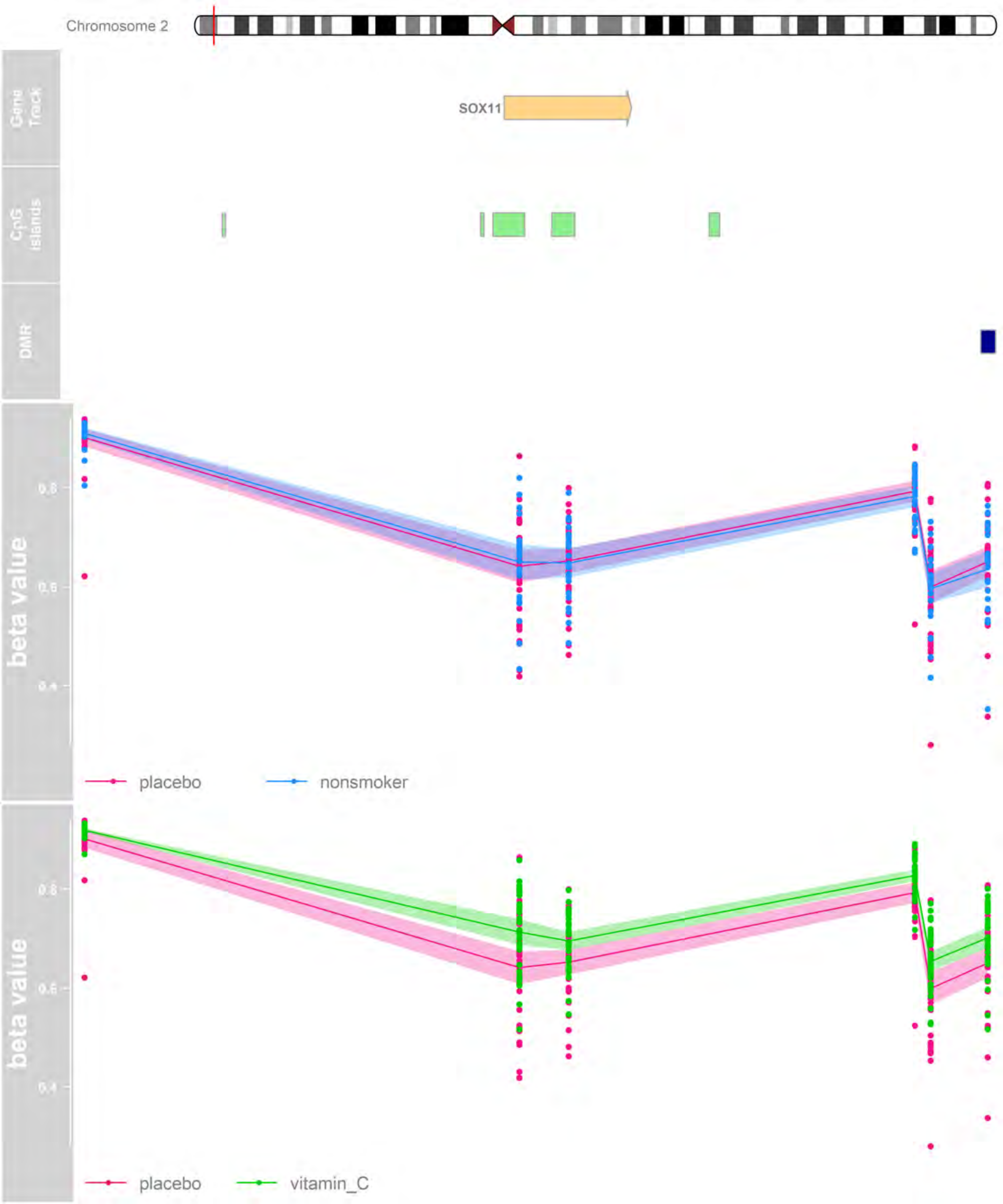

Chromosome 2
Gene Track
SOX11
CpG Islands
DMR
beta value
0.8
0.6
0.4
placebo
nonsmoker
beta value
0.8
0.6
0.4
placebo
vitamin_C

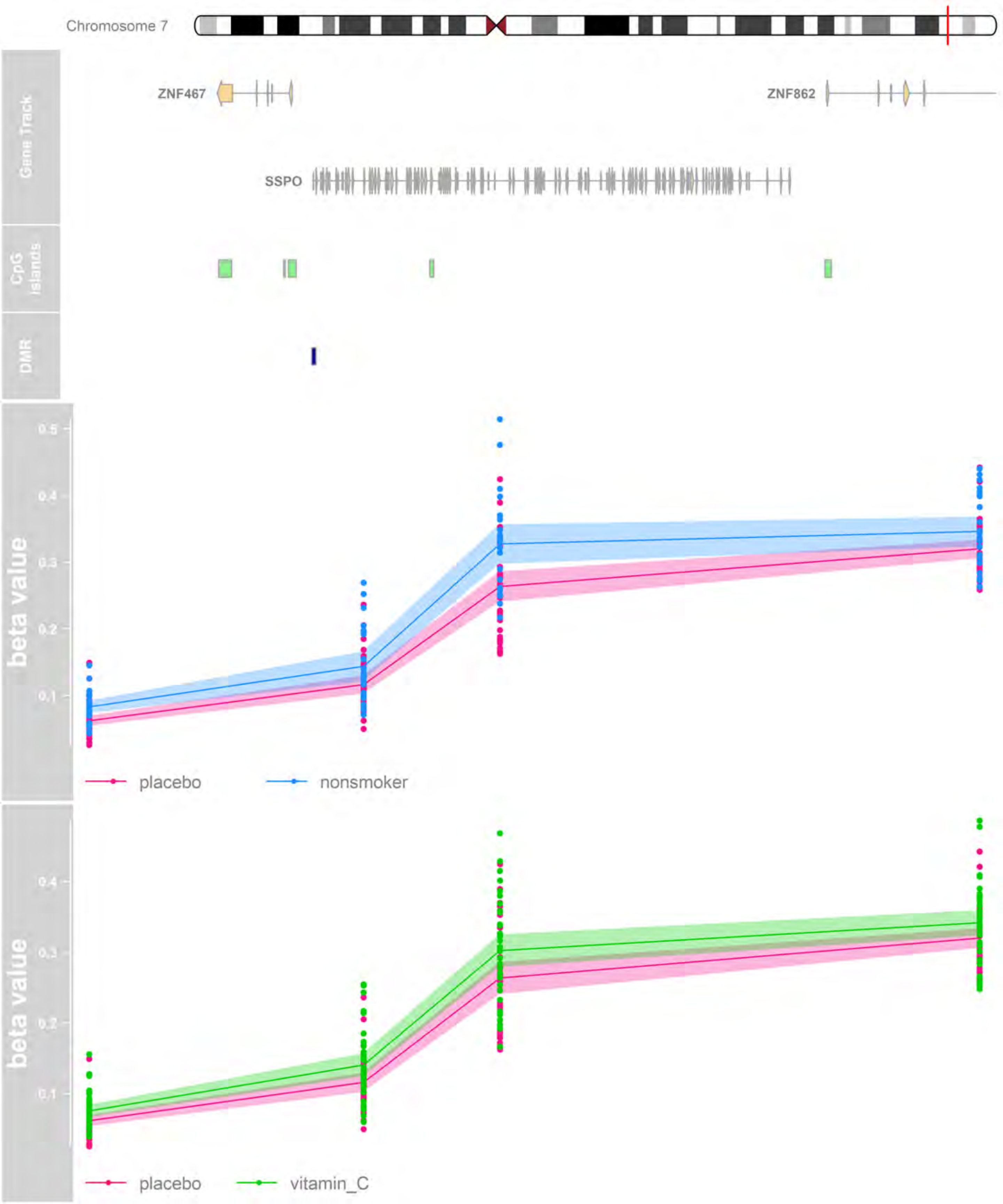

Chromosome 7
Gene Track
ZNF467
ZNF862
SSPO
CpG islands
DMR
beta value
0.5
0.4
0.3
0.2
0.1
placebo
nonsmoker
beta value
0.4
0.3
0.2
0.1
placebo
vitamin_C

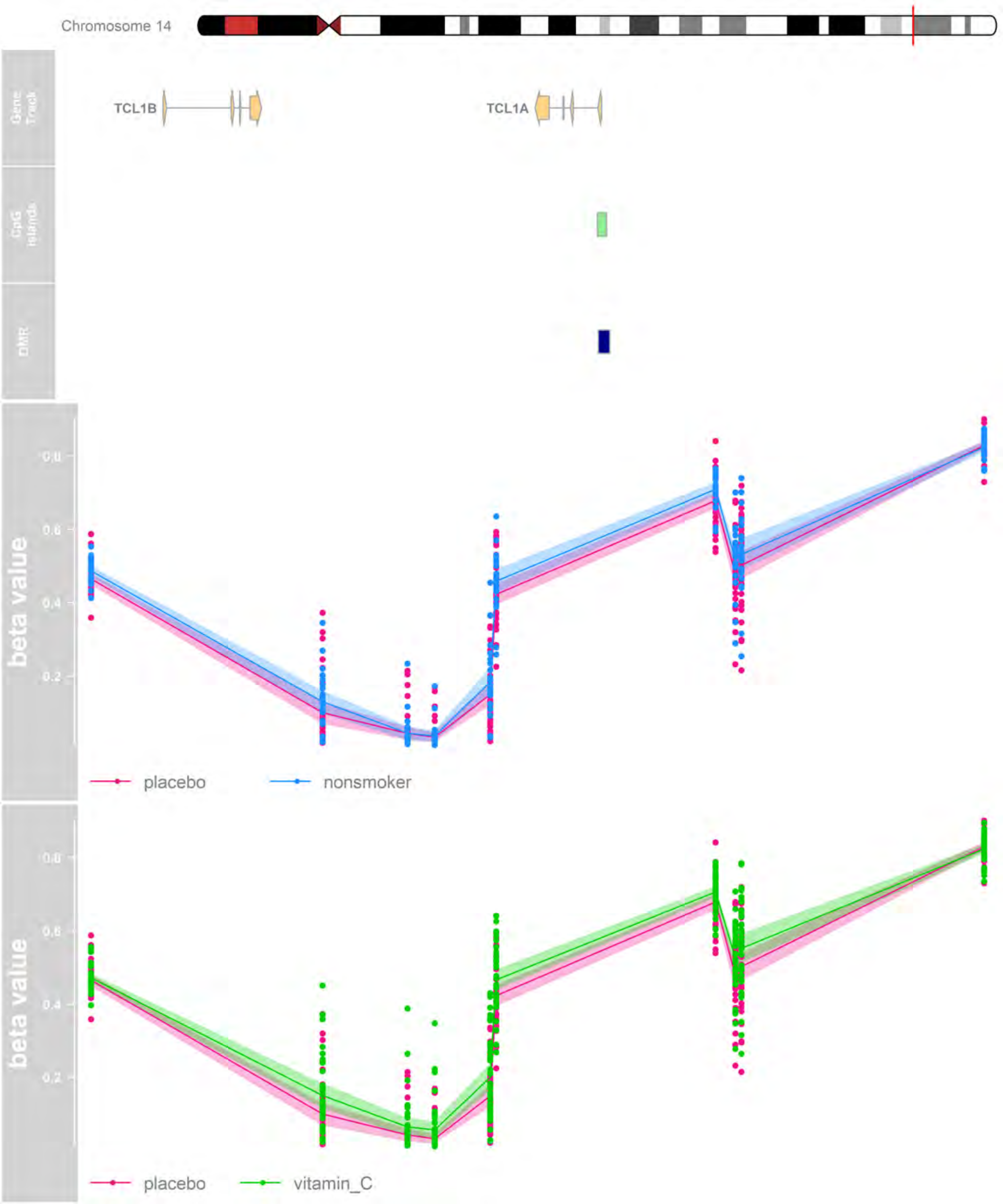
Chromosome 14
Gene Track
CpG islands
DMR
TCL1B
TCL1A
beta value
0.8
0.6
0.4
0.2
placebo
nonsmoker
beta value
0.8
0.6
0.4
0.2
placebo
vitamin_C

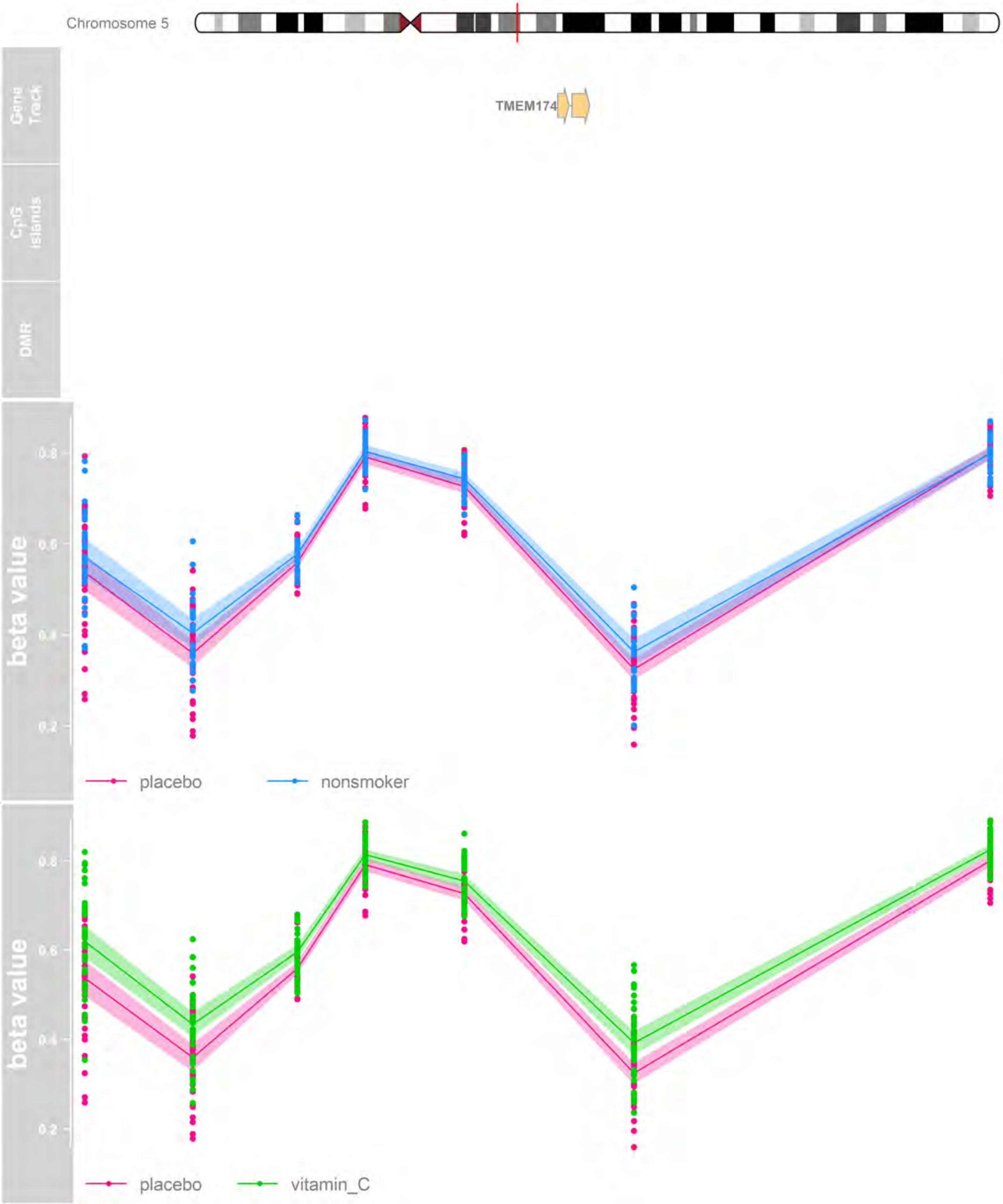

Chromosome 5
Gene Track
TMEM174
CpG islands
DMR
beta value
0.8
0.6
0.4
0.2
placebo
nonsmoker
beta value
0.8
0.6
0.4
0.2
placebo
vitamin_C

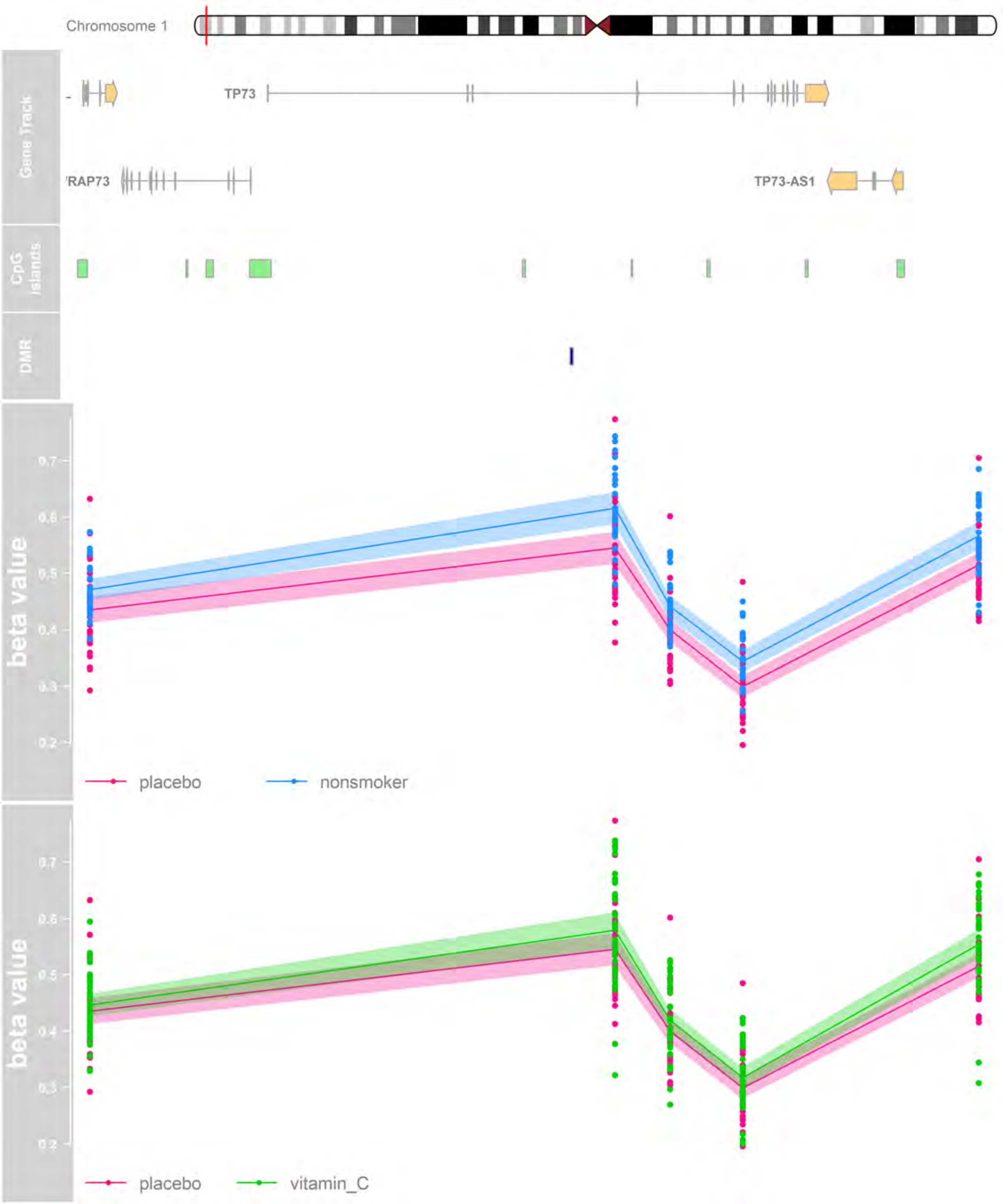

Chromosome 1
Gene Track
TP73
RAP73
TP73-AS1
CpG islands
DMR
beta value
0.7
0.6
0.5
0.4
0.3
0.2
placebo
nonsmoker
beta value
0.7
0.6
0.5
0.4
0.3
0.2
placebo
vitamin_C

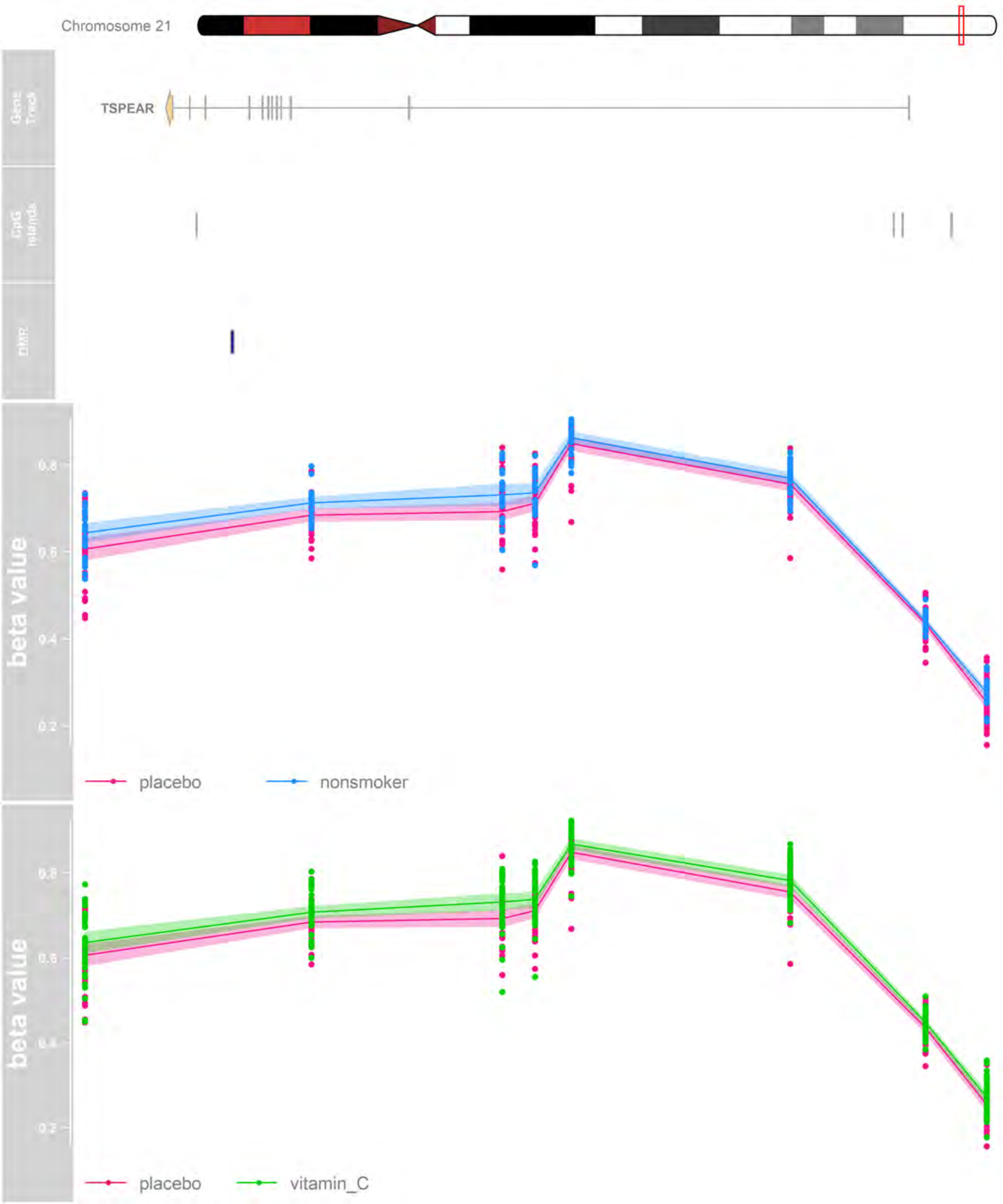

Chromosome 21
TSPEAR
beta value
0.8
0.6
0.4
0.2
placebo
nonsmoker
beta value
0.8
0.6
0.4
0.2
placebo
vitamin_C

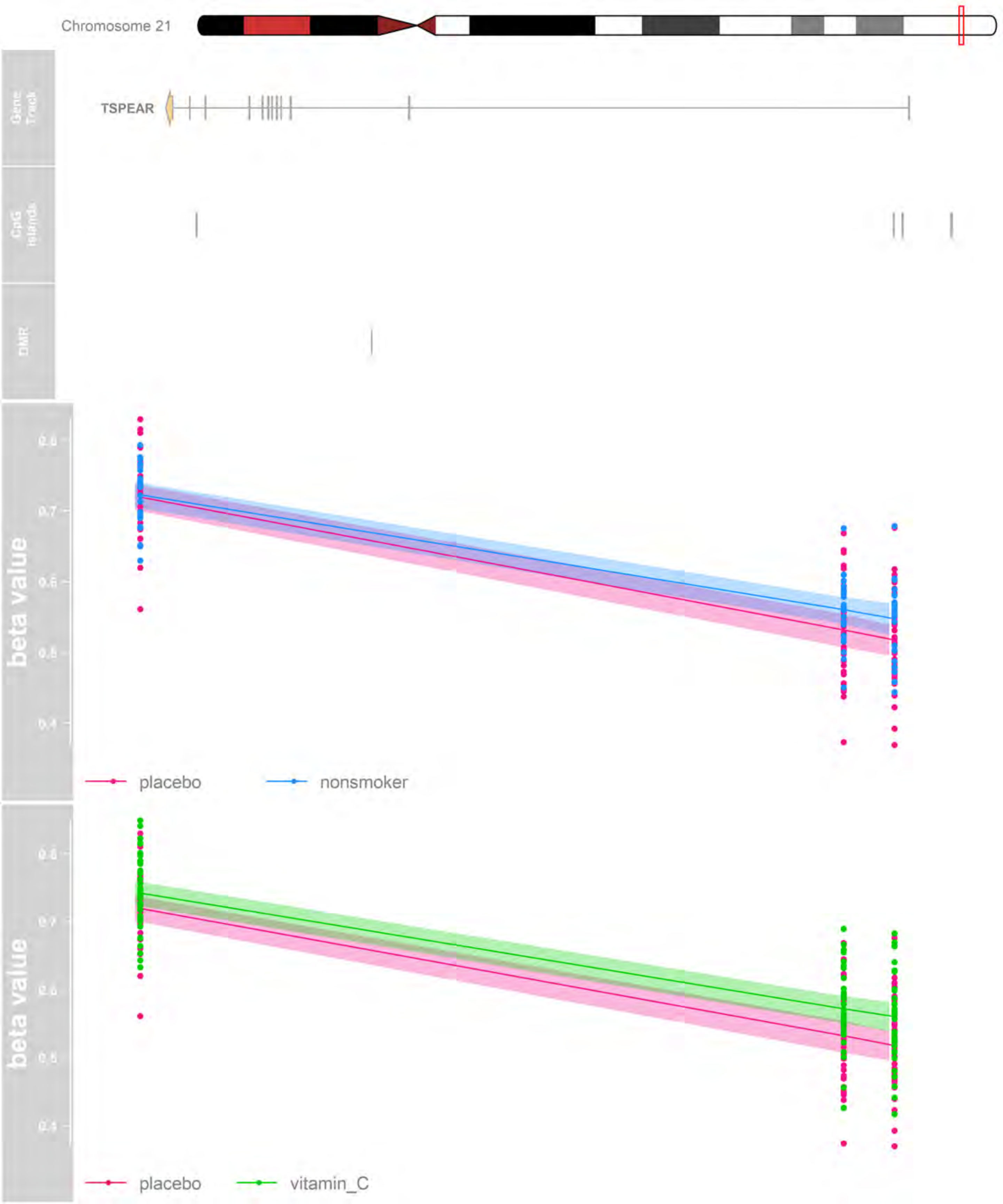

Chromosome 21
Gene Track
TSPEAR
CpG islands
DMR
beta value
0.8
0.7
0.6
0.5
0.4
placebo
nonsmoker
beta value
0.8
0.7
0.6
0.5
0.4
placebo
vitamin_C

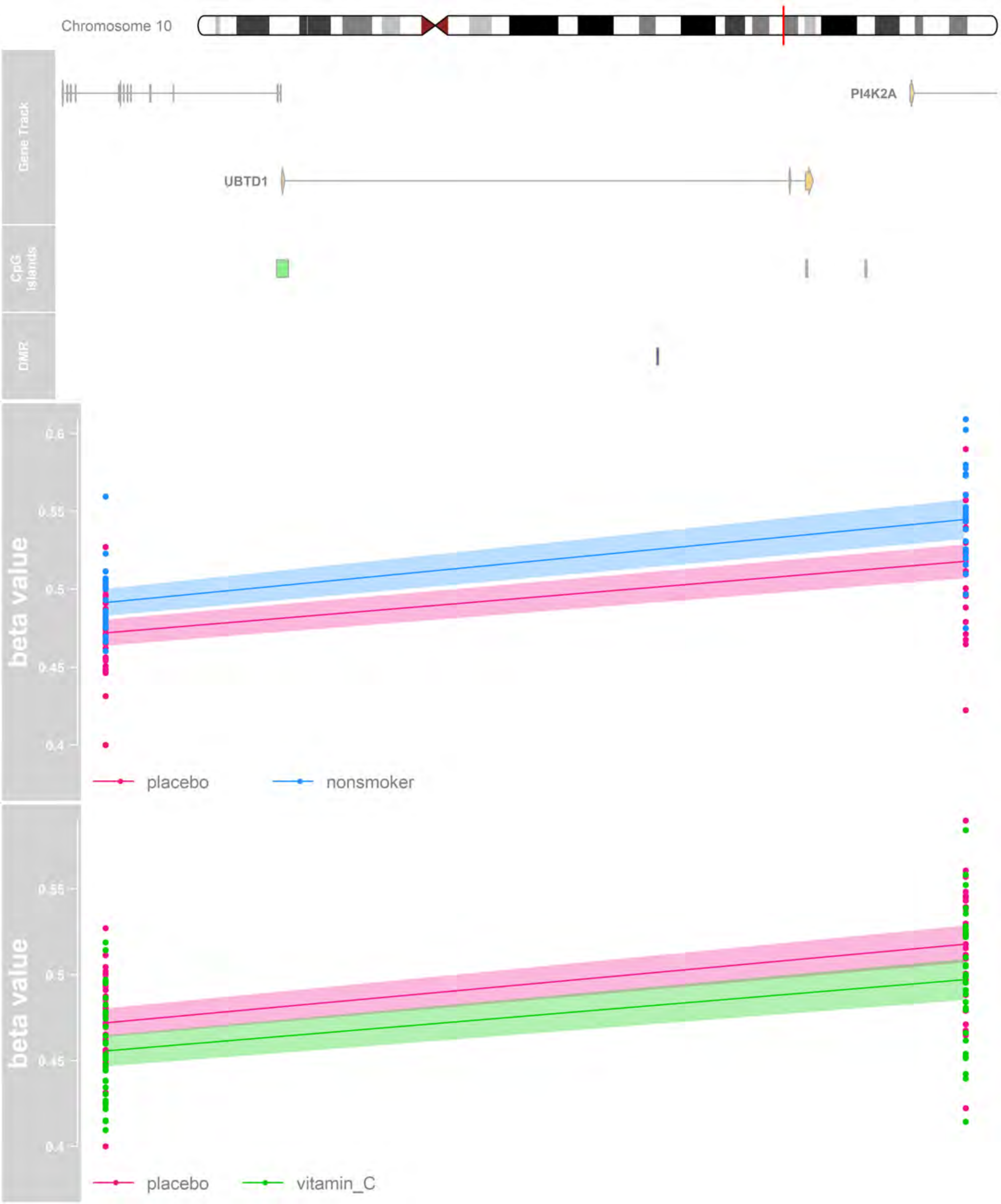

Chromosome 10
Gene Track
PI4K2A
UBTD1
CpG Islands
DMR
beta value
0.6
0.55
0.5
0.45
0.4
placebo
nonsmoker
beta value
0.55
0.5
0.45
0.4
placebo
vitamin_C

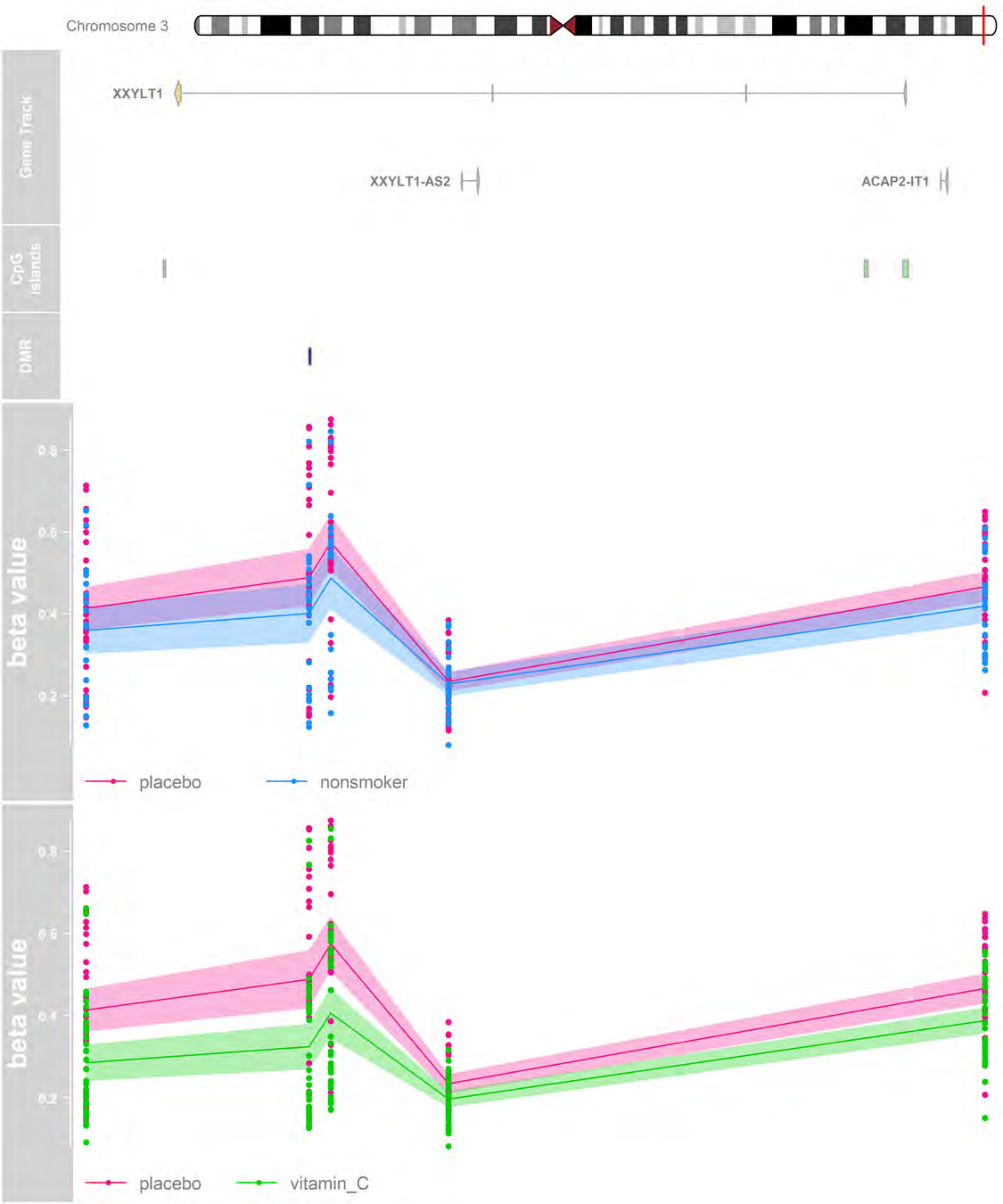

Chromosome 3
Gene Track
XXYLT1
XXYLT1-AS2
ACAP2-IT1
CpG islands
DMR
beta value
0.8
0.6
0.4
0.2
placebo
nonsmoker
beta value
0.8
0.6
0.4
0.2
placebo
vitamin_C

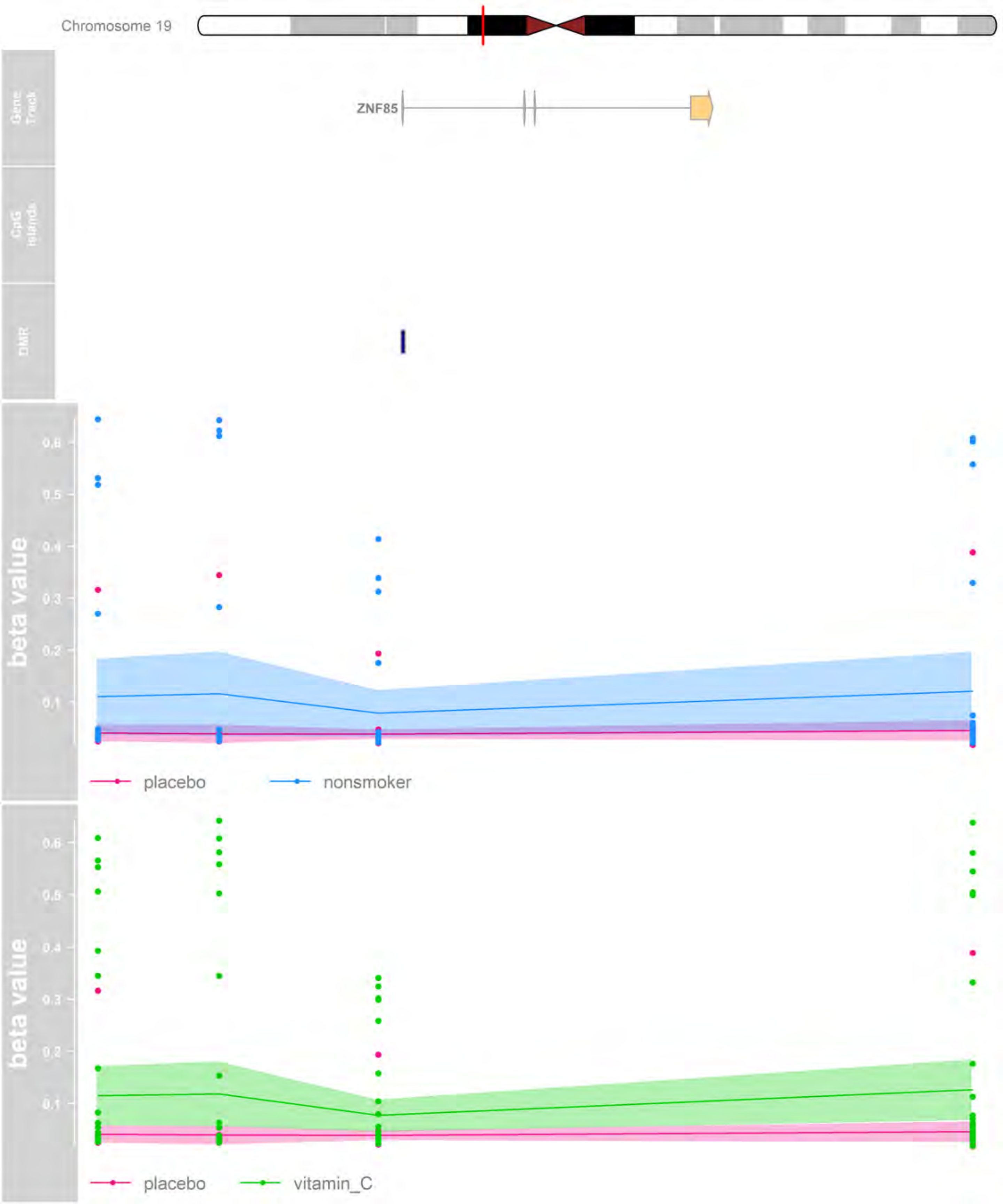

Chromosome 19
Gene Track
CpG islands
DMR
ZNF85
beta value
0.6
0.5
0.4
0.3
0.2
0.1
placebo
nonsmoker
beta value
0.6
0.5
0.4
0.3
0.2
0.1
placebo
vitamin_C

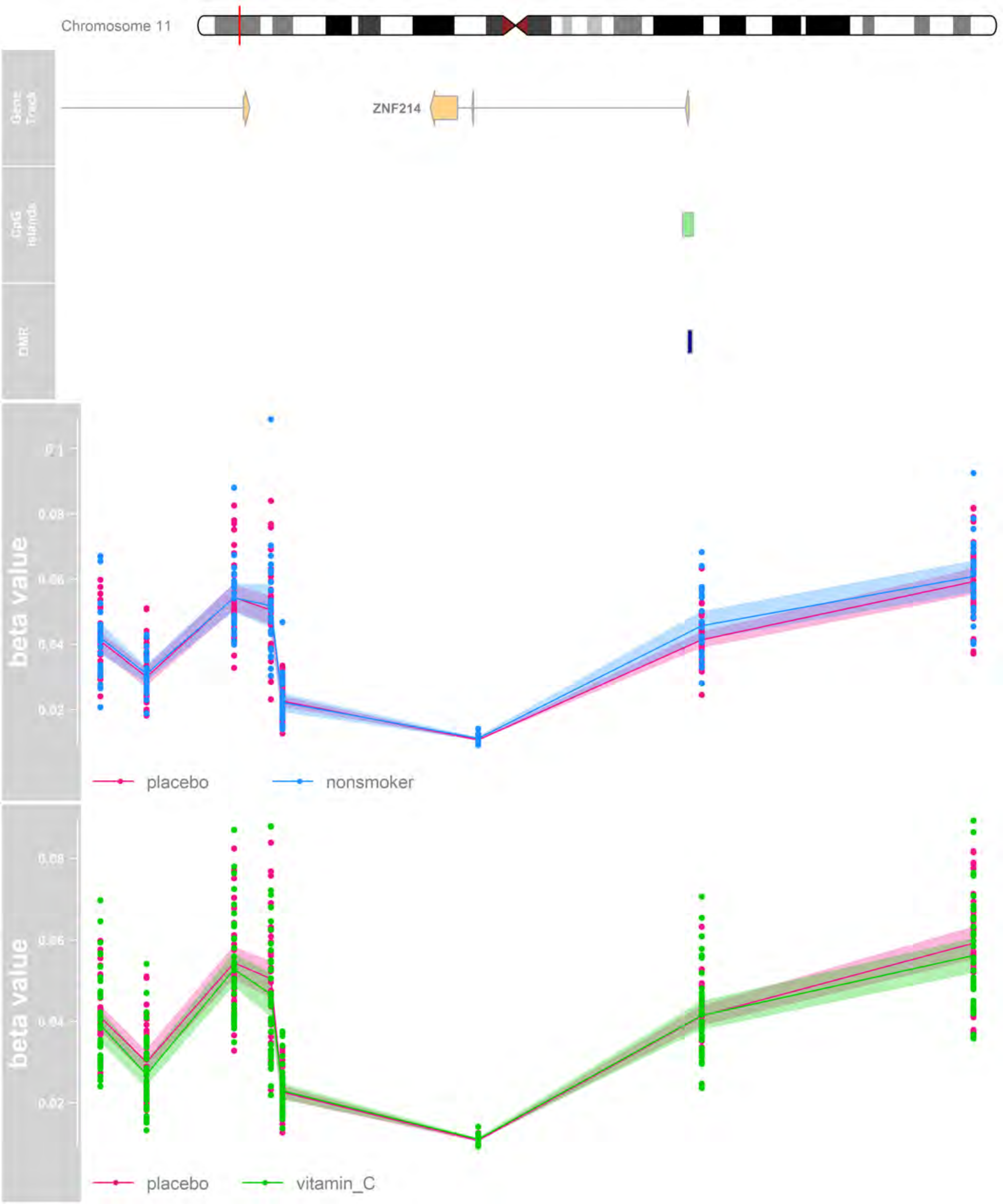

Chromosome 11
Gene Track
ZNF214
CpG islands
DMR
beta value
0.1
0.08
0.06
0.04
0.02
placebo
nonsmoker
beta value
0.08
0.06
0.04
0.02
placebo
vitamin_C

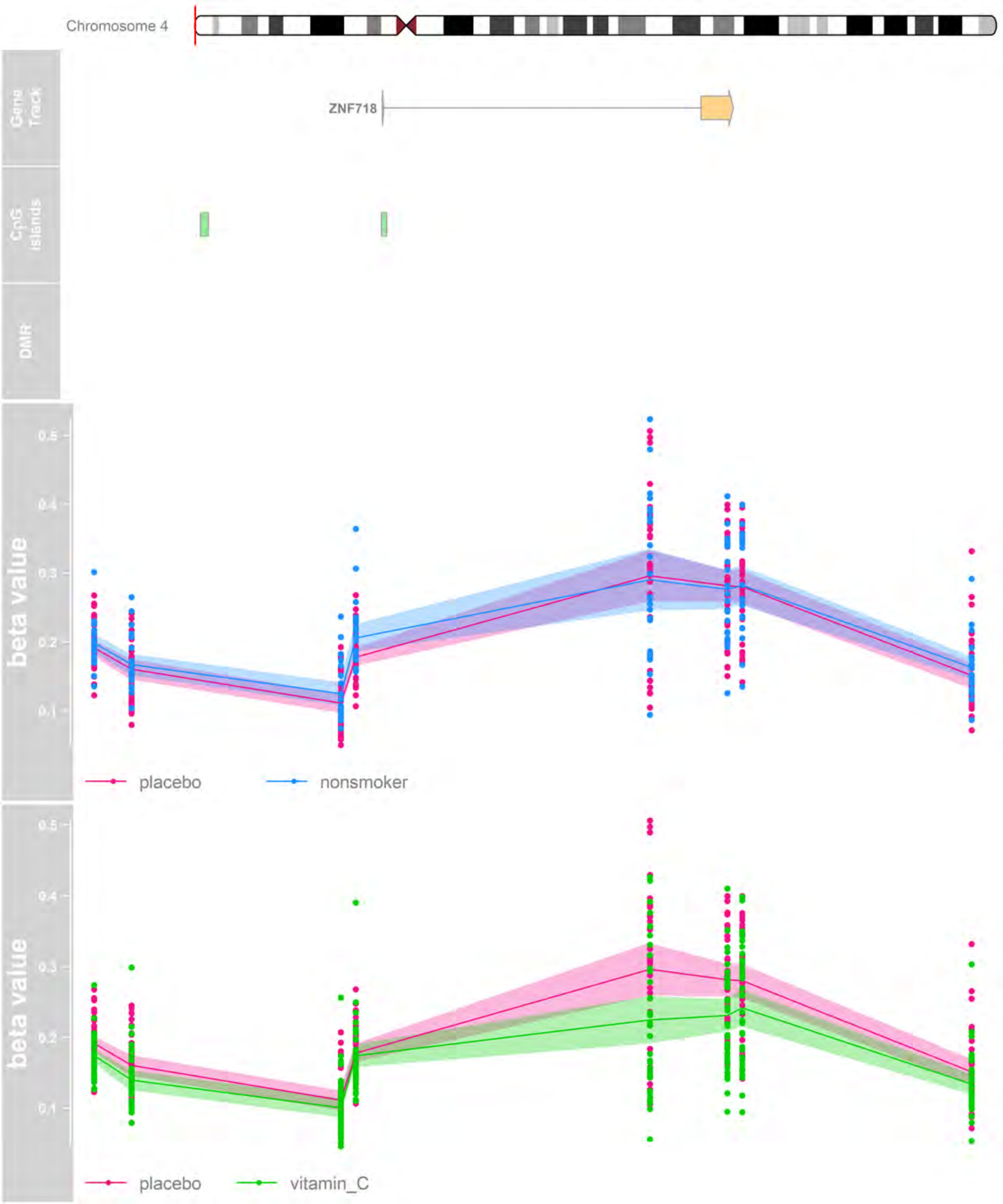

Chromosome 4
Gene Track
ZNF718
CpG islands
DMR
beta value
0.5
0.4
0.3
0.2
0.1
placebo
nonsmoker
beta value
0.5
0.4
0.3
0.2
0.1
placebo
vitamin_C
